# PCET-mediated deconstructive cross-coupling of aliphatic alcohols†

**DOI:** 10.1039/d5sc00737b

**Published:** 2025-03-27

**Authors:** Yeersen Patehebieke, Rima Charaf, Kumar Bhaskar Pal, Beatriz Meana Baamonde, Andjela Brnovic, Leif Hammarström, Carl-Johan Wallentin

**Affiliations:** a Department of Chemistry and Molecular Biology, University of Gothenburg Gothenburg SE 41390 Sweden carl.wallentin@chem.gu.se; b Department of Chemistry – Ångström Laboratory, Uppsala University Uppsala SE 75120 Sweden leif.hammarstrom@kemi.uu.se

## Abstract

A practical deconstructive arylation of aliphatic alcohols has been developed using a synergistic photoredox proton-coupled electron transfer (PCET) and nickel dual catalytic system. The method efficiently generates alkyl radicals *via* concerted PCET-mediated β-scission, enabling the formation of C(sp^3^)–C(sp^2^) bonds between alcohols and aryl halides. Optimization studies revealed a broad functional group tolerance and high chemoselectivity, with good yields even for challenging tertiary alcohol substrates. Mechanistic insights from transient absorption spectroscopy confirmed the dominance of a PCET pathway for radical generation. This strategy expands the utility of alcohols as alkyl radical precursors in cross-coupling reactions, offering a versatile tool for constructing complex molecular architectures.

## Abstract 2

A practical deconstructive arylation of aliphatic alcohols has been developed using a synergistic photoredox proton-coupled electron transfer (PCET) and nickel dual catalytic system.

## Graphical abstract

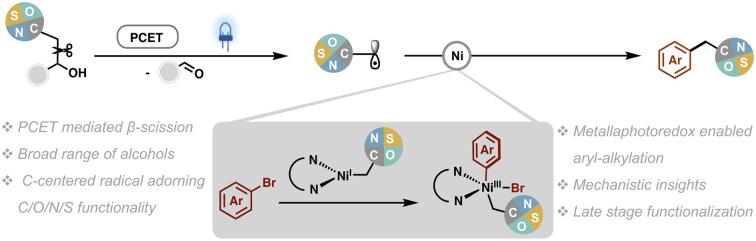

## Introduction

The rapid creation of molecular complexity and exploration of new 3D chemical space is crucial for efficient and successful drug discovery.^1^ Nickel photoredox dual catalysis has emerged as a promising approach to achieve this, enabling the fast introduction of C(sp^3^) centers under mild conditions.

Pioneering work by MacMillan, Doyle,^2^ and Molander^3^ has demonstrated that alkyl radicals can be viable partners in photoredox nickel dual catalytic cross-coupling to form new C(sp^3^)–C(sp^2^) bonds. This method has since become a powerful strategy for forming challenging C(sp^3^)–C(sp^2^) and C(sp^3^)–C(sp^3^) bonds under mild conditions, with broad functional group tolerance, providing convenient access to structurally diverse scaffolds in organic synthesis.^4^

**Fig. 1:**
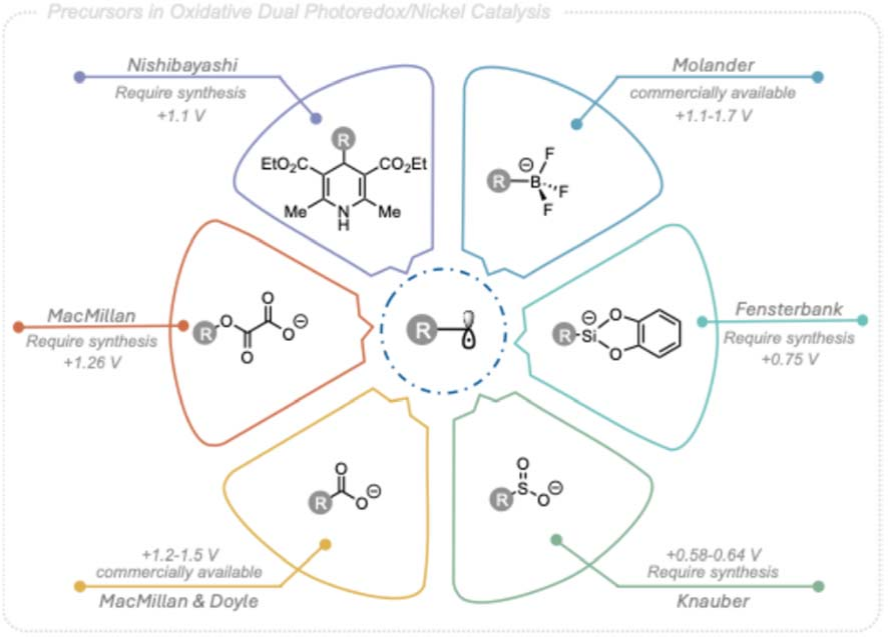
Alkyl radical precursors used in Ni-photoredox cross-coupling reactions.

The desire to expand the scope of coupling partners for this powerful tool to simpler, cheaper, and more abundant starting materials has led to a broad variety of alkyl radical precursors being applicable in photoredox nickel dual catalytic C(sp^3^)–C(sp^2^) cross-coupling. These include carboxylic acids,^2^ trifluoroborate salts,^3,5^ 4-alkyl-1,4-dihydropyridines (DHPs),^6^ oxalates,^7^ sulfinates,^8^ alkyl halides,^9^ silicates,^10^ and C(sp^3^)–H bonds,^11^ (Fig. 1). Although these precursors span a wide range of chemical diversity, many are not naturally occurring leading to limited commercial availability and frequently complex synthesis routes. In addition, the redox potentials and/or stability characteristics associated with these radical precursors are coupled to limitations in synthetic applicability. In contrast, alcohols are abundant in both natural and industrial sources and harbors stability features highly compatible with divers conditions,^12^ making them an appealing option as C(sp^3^)-centered radical precursors in photoredox nickel dual catalytic cross-coupling reactions targeting structurally diversity.

**Fig. 2:**
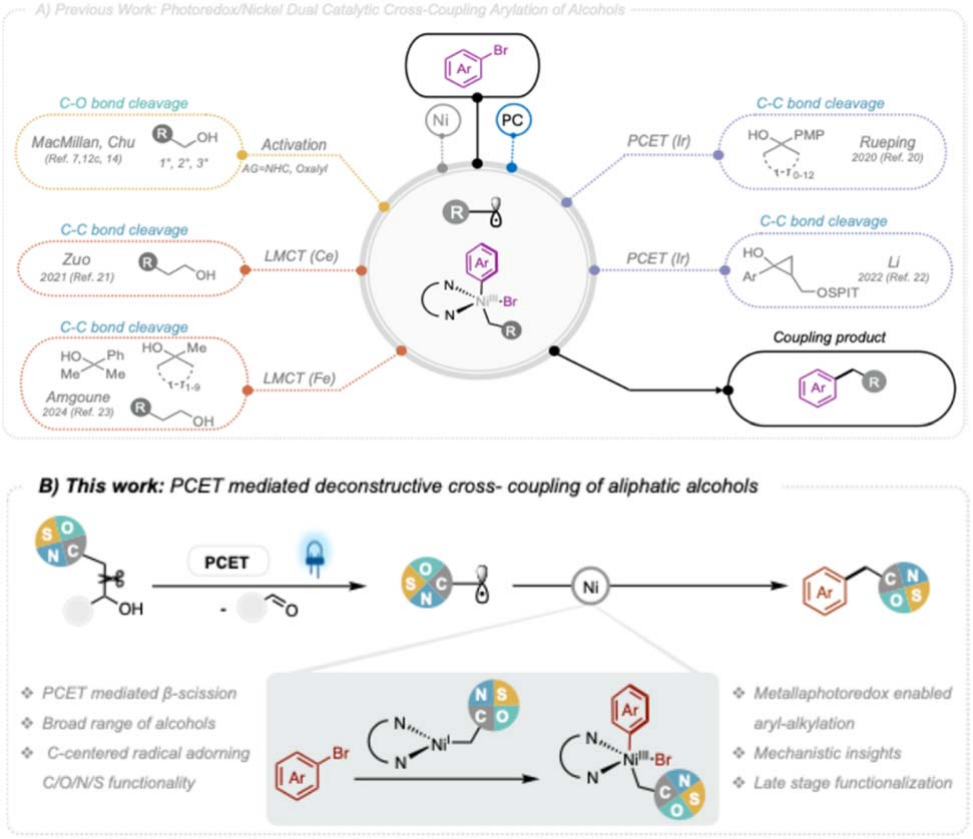
(A) Previous reported dual catalytic cross-coupling arylation of alcohols. (B) This work: PCET-mediated deconstructive cross-coupling of aliphatic alcohols.

Recently, various radical deoxyfunctionalisation strategies have been developed. These processes are able to efficiently engage hydroxyl groups in photoredox mediated mesolytic or fragmentative generation of C-centered radicals that consequentially partake in nickel catalyzed formation of C–C, C–H, or C–heteroatom bonds (Fig. 2A).^13^ Most of these methods facilitate the generation of alkyl radicals from alcohols through the use of various activation strategies. For instance, oxalates derived from primary, secondary, or tertiary alcohols have been widely employed as radical precursors, allowing the efficient formation of C(sp^3^)–C(sp^2^) bonds with aryl halides.^7*a*,14^ Recently, the MacMillan group reported an elegant photoredox Ni-mediated process for the arylation of free alcohols.^12*c*^ The main advantage of this strategy, compared to the previous use of oxalates, is that it eliminates the need for isolating preactivated starting materials. Instead, the corresponding alcohols are activated *in situ* by condensation with an NHC reagent, facilitating C–O bond cleavage and radical generation under visible light irradiation. This *in situ* NHC activation method have inspired other research groups to further advance the deoxyfunctionalization of alcohols, not only expanding the range of substrates but also enabling enantioconvergent cross-coupling reactions.^15^

Seminal work by Knowles and Zuo demonstrated the generation of alkyl radicals directly from free alcohols *via* photocatalytic proton-coupled electron transfer (PCET)^16^ or ligand-to-metal charge transfer (LMCT) mechanisms.^17^ These strategies mainly utilized the generated radicals in Giese-type addition reactions.^18^ In the context of photoredox nickel dual catalysis, the use of aliphatic alcohols as alkyl radical synthons is rare.^19^ To date, there are only four notable examples in the literature where C-centered radicals are generated directly from free alcohols through PCET or LMCT and used in photoredox nickel dual catalytic cross-coupling reactions (Fig. 2A).

In 2020, the Rueping group utilized the PCET activation mode in combination with a nickel-catalyzed cross-coupling reaction, successfully achieving C–C bond cleavage and arylation of cycloalcohols using aryl halides.^20^ Later, in 2021, the Zuo group employed a cerium-catalyzed LMCT activation mode to generate alkyl radicals, which were then combined with a nickel-catalyzed cross-coupling reaction to successfully achieve a dehydroxymethylative arylation of primary aliphatic alcohols with aryl halides.^21^ In 2022, the Li group developed an enantioselective β-arylation of cyclopropanols with aryl bromides, employing a dual catalyst system involving PCET-mediated photoredox and nickel catalysis.^22^ Similar to the strategy pursued by the Zuo group,^21^ Tran and Amgoune published a dual catalytic system that utilizes a photoinduced LMCT strategy based on iron in 2024.^23^ While these protocols showcase the flexibility of using alcohols as alkyl radical precursors, they come with some limitations associated with restriction to viable substrates, such as cycloalkanols or primary alcohols, and typically α-hetero C-centered radicals are not well tolerated.

Given the synthetic opportunities presented by direct generation of alkyl radicals from alcohols in photoredox nickel-dual catalysis and inspired by our recent work,^24^ alongside that of Huang and Chen,^25^ we here present a practical deconstructive arylation of aliphatic alcohols *via* synergistic photoredox PCET and nickel dual catalysis (Fig. 2B). Our approach involves generating alkyl radicals through concerted PCET mediated β-scission, these radicals are then captured by the nickel-catalyzed cross-coupling cycle, paving the way for constructing C(sp^3^)–C(sp^2^) bonds between aliphatic free alcohols and aryl halides. The process showcases an unmet compatibility with a broad scope of alcohols, including those harboring α-C, α-N, α-O, α-S hydroxyl groups.

The mechanistic complexity of this type of transformations remains challenging and is thus still elusive. Our previous study,^24^ using nanosecond transient absorption (ns-TA) experiments and DFT calculations, revealed that direct fragmentation (indirect non-PCET) is an active mechanistic component and likely constitutes the dominant pathway for radical generation. In the present study, we have employed femto- and nano-second transient absorption spectroscopy and fluorescence quenching measurements to gain further mechanistic insights. Our findings suggest that an indirect concerted PCET fragmentation is faster and more favorable for generating C-centered radicals from alcohols as compared to non-PCET pathways or PCET processes involving O-centered radical intermediates. These findings underscore the crucial role and intricate nature of PCET mechanisms in these types of processes.

## Results and discussion

### Optimization

To assess whether our previously developed PCET-mediated alkyl radical generation process was compatible with nickel-catalyzed cross-coupling, we initiated our study using alcohol S1 and aryl bromide 1 as model substrates. The substrates were subjected to a nickel catalyst and conditions like our previously developed method for PCET-mediated deconstructive radical formation (Table 1). To our delight, the desired C(sp^3^)–C(sp^2^) coupled product 2 was formed in a 69% yield (Table 1, entry 2) using Me-Acr-Mes^+^ as the photocatalyst, NiCl_2_·glyme (glyme = ethylene glycol dimethyl ether)/4,4′-di-*tert*-butyl-2,2′-dipyridyl (dtbbpy) as the cross-coupling catalyst, 2,4,6-collidine as the base, in 1,2-dichloroethane (DCE) at 35 °C under 450 nm blue light irradiation for 48 h. Following extensive screening of solvents, bases, photocatalysts, nickel catalysts, ligands, and reaction times (see ESI† for details), the yield could be increased to 84% (Table 1, entry 1). The solvent system giving the highest yield involved using 1% ACN as a co-solvent. It is unclear why this specific mixture gave higher yields, however we speculate that this solvent system might provide suitable characteristics in terms of polarity and Brønsted basicity needed to efficiently promote precursor complex formation as well as disaggregation of the successor complex.^26^ The latter would mitigate non-productive back electron transfer. We also evaluated commonly used iridium photocatalysts and tetrabutylphosphonium diphenyl phosphate base, which have previously been employed in nickel-catalyzed cross-coupling and photo-redox PCET reactions. However, these conditions resulted in significantly lower yields (Table 1, entries 3 & 4). Interestingly, the acridinium photocatalyst with a BF_4_^−^ counterion exhibited reduced reactivity, an observation in congruous with recent reports (Table 1, entry 5).^27^ It has been suggested that increased reactivity of acridinium perchlorate as compared with the corresponding tetra fluoroborate may be accounted for by trace chlorine radical generation stemming from the perchlorate salts.^27*a*,28^

**Table 1:** Optimization of reaction conditions^a^

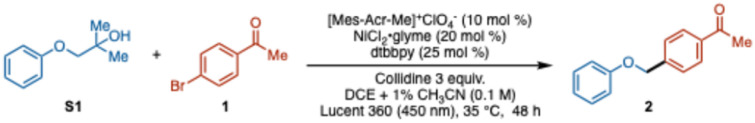		
Entry	Deviation from standard conditions	Yield^b^ (%)
1	No deviation	84 (75^c^)
2	DCE	69 (57^c^)
3	[Ir(dF(CF_3_)ppy)_2_(5,5′-dCF_3_bpy)]^+^PF_6_^−^	14
4	(*n*-Bu)_4_P^+^ (PhO)_2_(O)PO^−^	11
5	[Mes-Acr-Me]^+^BF_4_^−^	54
6	NiCl_2_·glyme (15 mol%)	39
7	24 h	53
8	No photocatalyst	n.d.
9	No light	n.d.
10	No base	n.d.
11	No nickel catalyst	n.d.
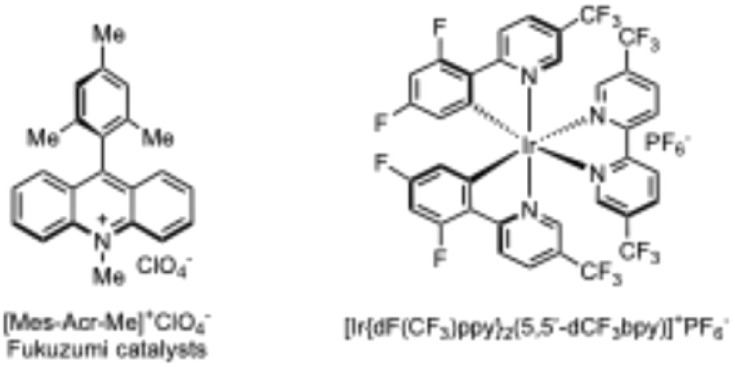		

a Reaction conditions: alcohol S1 (0.3 mmol), aryl bromide 1 (0.1 mmol), 2,4,6-collidine (0.3 mmol), [Mes-Acr-Me]^+^ClO_4_^−^ (0.01 mmol), NiCl_2_·glyme (0.02 mmol), dtbbpy (0.025 mmol), DCE + 1% CH_3_CN (3 mL), time (48 h), photo reactor (Lucent 360, 450 nm, 50% light intensity), temperature (35 °C).

b ^1^H NMR yields determined by using ethylene carbonate as an internal standard.

c Isolated yield.

Reducing the loading of the nickel catalyst led to a decrease in yield (Table 1, entry 6), and shortening the reaction time had a similar negative effect (Table 1, entry 7). Control experiments confirmed that no product formation was observed when any of the key components (photocatalyst, nickel catalyst, base, or blue light irradiation), were omitted (Table 1, entries 8–11), underscoring the essential role of all components in this catalytic system.

### Substrate scope

With the optimized conditions established, we investigated the substrate scope using a range of aryl and heteroaryl halides. The reactions proceeded smoothly with aryl halides bearing electron-withdrawing groups (Table 2). Substrates with various electron-deficient substituents at the *para*-position, such as ketones (2, 3), trifluoromethyl (4), ester (5), aldehyde (6), nitrile (7), and sulfonamide (8), were well-tolerated, yielding the desired cross-coupling products in moderate to good yields (39–81%), demonstrating good chemoselectivity. However, electron-rich bromoarenes showed reduced reactivity, resulting in lower yields (9–10), which agrees well with the current common limitations in the photoredox nickel cross-coupling reactions.^4*c*^

**Table 2:** Scope of aryl halides or heteroaryl halides^a^

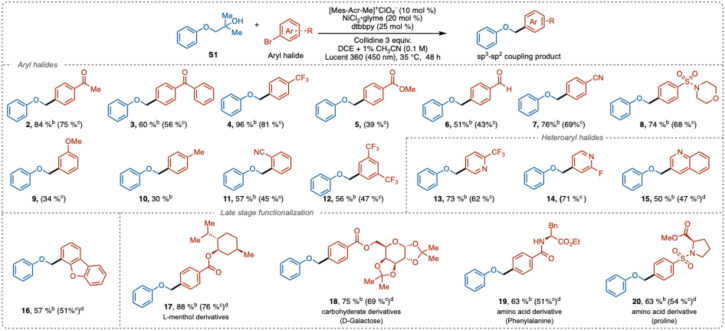

a Reaction conditions: alcohol S1 (0.3 mmol), aryl bromide (0.1 mmol), 2,4,6-collidine (0.3 mmol), [Mes-Acr-Me]^+^ClO_4_^−^ (0.01 mmol), NiCl_2_·glyme (0.02 mmol), dtbbpy (0.025 mmol), DCE + 1% CH_3_CN (3 mL), time (48 h), photo reactor (Lucent 360, 450 nm, 50% light intensity), temperature (35 °C).

b ^1^H NMR yields determined by using ethylene carbonate as an internal standard.

c Isolated yield.

d NiBr_2_·dtbbpy (0.02 mmol) used.

*Ortho*-Substituted and disubstituted aryl bromides were also compatible with this transformation (11–12), highlighting the method's efficiency in sterically hindered environments. A variety of heteroaromatic substrates, including bromopyridines (13–14), quinoline (15), and dibenzofuran (16), were also rather effective coupling partners, yielding products in 47–68%.

To further demonstrate the synthetic utility, we applied our method to the late-stage functionalization of structurally diverse, pharmaceutically relevant compounds such as l-menthol (17), d-galactose (18), and amino acid derivatives (19–20). All these substrates were well-tolerated, delivering the cross-coupling products in good yields (51–76%).

Next, we explored the scope of compatible aliphatic alcohols. Both stabilized and unstabilized carbon-centered radicals were efficiently generated from secondary and tertiary alcohols under our reaction conditions and successfully coupled with aryl halides to form new C(sp^3^)–C(sp^2^) bonds with good to excellent yields (Table 3). Alkyl, cycloalkyl, and heterocyclic groups, including isopropyl (21), *tert*-butyl (22), cyclopentyl (23), cyclohexyl (24), and tetrahydropyran (25), showed good reactivity in the deconstructive cross-coupling arylation. Notably, the sterically hindered and electron-rich *tert*-butyl radical, which provide a product harboring a quaternary carbon center, gave cross-coupling product 22 in 35% isolated yield. This result outperforms the previous reported methods relying on iron-catalyzed generation of the C-centered radical (5%),^23^ while MacMillan's NiBr_2_·dtbbpy/NHC activation method^12*c*^ only generated trace amounts of the aryl coupled product.

**Table 3:** Scope of alcohols^a^

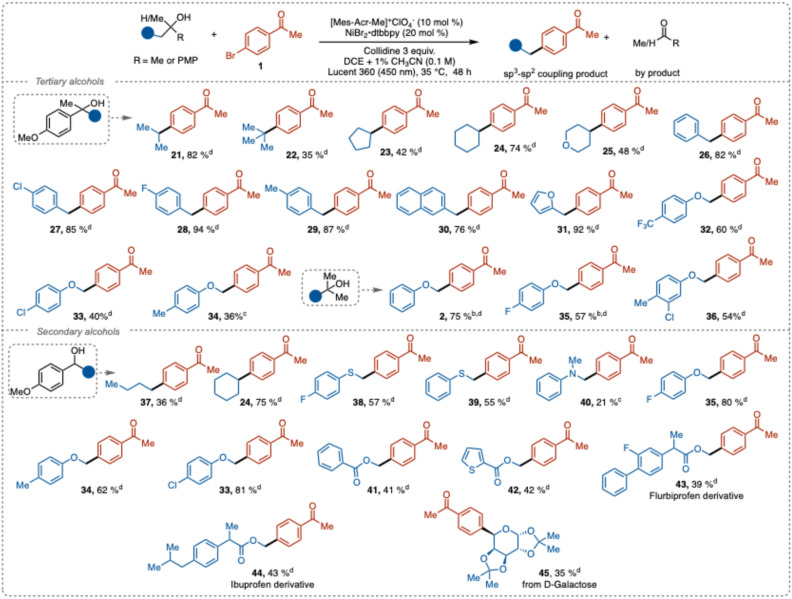

a Reaction conditions: alcohol (0.3 mmol), aryl bromide 1 (0.1 mmol), 2,4,6-collidine (0.3 mmol), [Mes-Acr-Me]^+^ClO_4_^−^ (0.01 mmol), NiBr_2_·dtbbpy (0.02 mmol), DCE + 1% CH_3_CN (3 mL), time (48 h), photo reactor (Lucent 360, 450 nm, 50% light intensity), temperature (35 °C).

b NiCl_2_·glyme (0.02 mmol), dtbbpy (0.025 mmol) used.

c ^1^H NMR yields determined by using ethylene carbonate as an internal standard.

d Isolated yield.

Benzylic derivatives with neutral (26), electron-withdrawing (27), and weak electron-donating groups (28–29), along with naphthalene and furan derivatives (30–31), exhibited excellent reactivity, resulting in good to near quantitative yields (76–94%). Phenolic derivatives with electron-withdrawing (32–33) and electron-donating groups (34) also provided synthetically useful yields. Furthermore, 2-methyl-1-phenoxypropanol derivatives with electron-withdrawing or neutral groups associated with the aromatic ring (2, 35–36) also afforded good product yields. Unfortunately, with the electron-donating groups on the benzene ring the reaction failed to proceed.

Radical precursors based on secondary alcohols also underwent successful C–C bond cleavage to generate the corresponding alkyl radicals, which similarly to the tertiary alcohol substrates engaged prosperously in the cross-coupling reaction (37–45). Compared to tertiary substrates (33, 34, 35), the secondary analogues provided the products in higher yields. A plausible explanation for this result can be attributed to that steric effect the complex formation between the substrate and collidine. A more pronounced acid–base complex formation would facilitate the PCET fragmentation and thus mitigate back electron transfer. Possibly, the sterics of the substrate and the role of the ACN additive might affect the process in a synergistic fashion.

We also explored the reactivity of α-thio- and α-aza-carbon-centered radicals generated from the corresponding congeners of 35. These radicals demonstrated reactivity almost comparable to that of the α-oxo radicals, with the thio derivative yielding cross-coupling products in moderate yields (38–39, 55–57%). Aniline-derived alcohols gave diminished yield (40, 21%), likely due to competing non-productive redox-processes. Alcohol derivatives containing methyl benzoate (41) and methylthiophenecarboxylate (42) provided cross-coupling products in synthetically relevant yield.

Finally, to illustrate the potential of this method for late-stage functionalization of natural products or pharmaceutically relevant compounds, we subjected derivatives of flurbiprofen, ibuprofen, and d-galactose to the deconstructive cross-coupling reaction. Satisfactory yields of the cross-coupling products were obtained (43–45), further demonstrating the versatility of this protocol.

### Mechanistic investigations

**Fig. 3:**
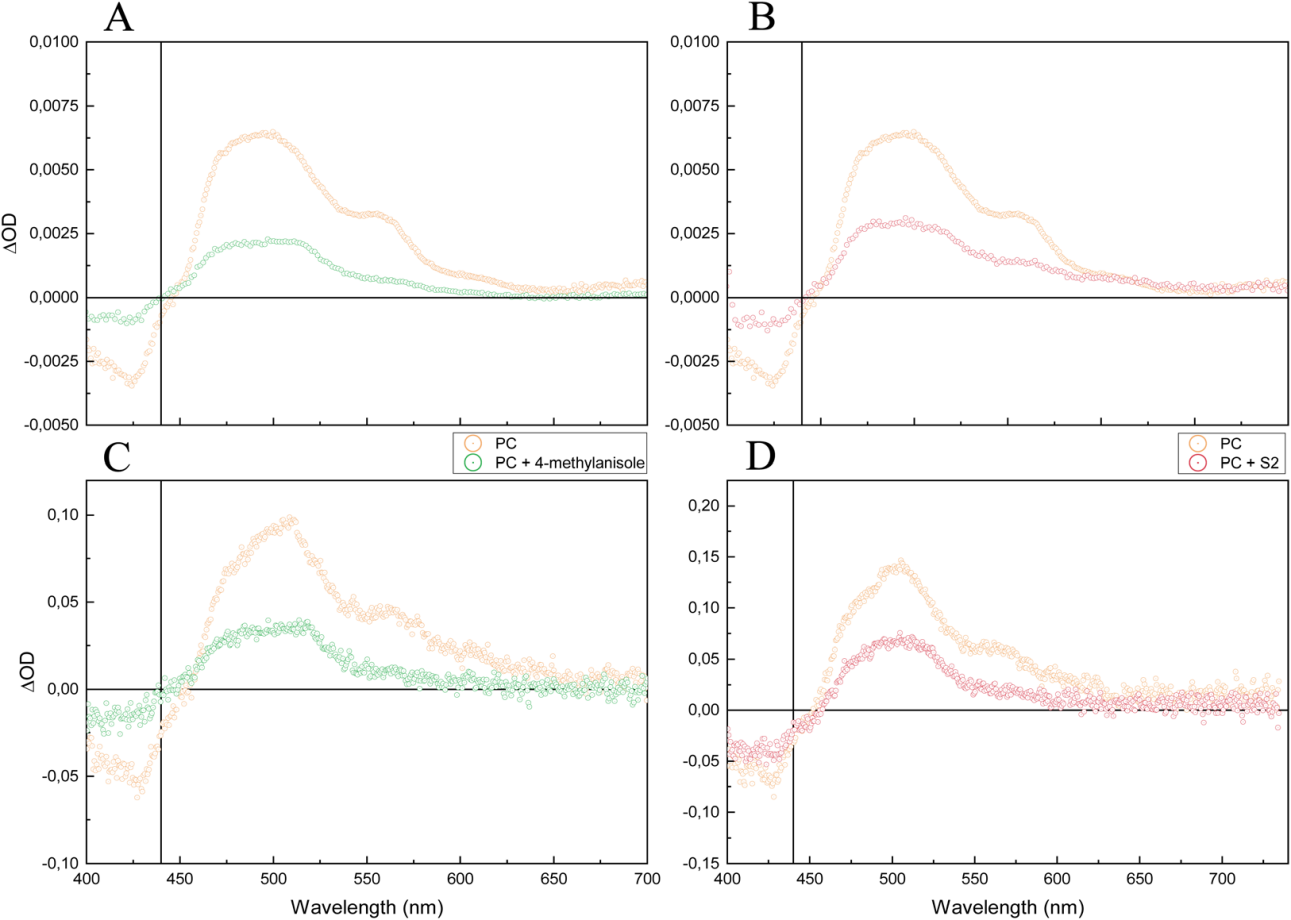
Transient absorption spectra of the Me-Acr^+^-Mes photocatalyst (PC) with the reference molecule (left, 4-methylanisole, 40 mM) and the model alcohol substrate (right, S2, 40 mM). (A) & (B): at 5 ns after laser excitation at 400 nm. Both substrates show a growth of the signal around 450 nm, the characteristic absorption of the methoxybenzene radical cation. (C) & (D): at 100–300 ns after laser excitation at 430 nm (reproduced from ref. 24). Only the 4-methylanisole shows the positive contribution in absorption around 450 nm. The vertical line at 440 nm shows the wavelength at which kinetic traces were collected to follow the disappearance of the methoxybenzene radical cation.

**Fig. 4:**
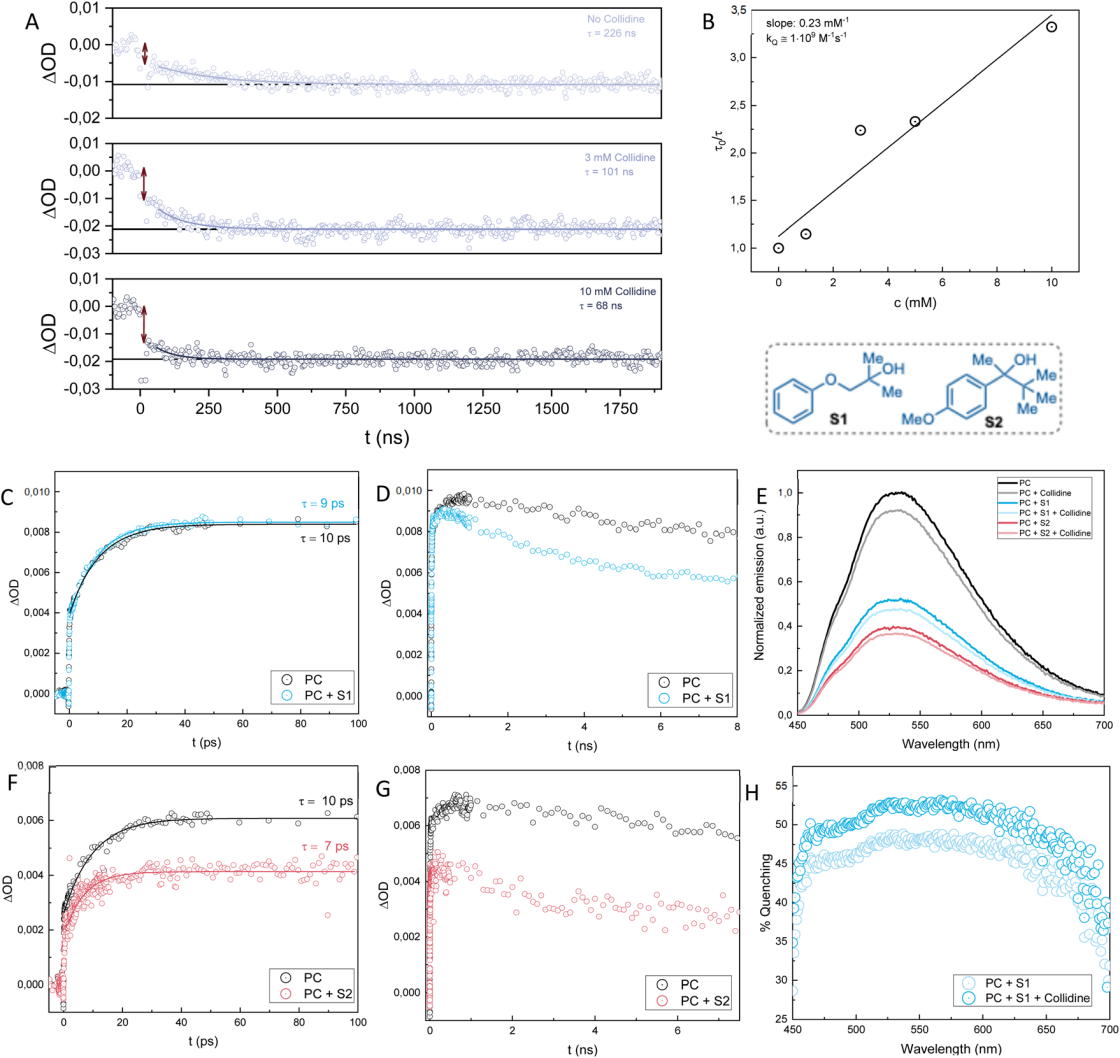
(A): Kinetic traces of changes in absorption at 440 nm upon laser excitation at 430 nm of samples of Me-Acr^+^-Mes and S2 (40 mM) with increasing concentration of collidine (0, 3 and 10 mM). The traces are fitted with single-exponential decays. The double arrows indicate the initial drop in amplitude. (B): Stern–Volmer plot of the lifetimes at 440 nm of Me-Acr^+^-Mes and S2 (40 mM) with increasing concentration of collidine (0, 1, 3, 5, 10 mM). Kinetic traces of changes in absorption at 480 nm on ps (C & F) and ns (D & G) timescales upon laser excitation at 400 nm of Me-Acr^+^-Mes with S1 (40 mM) and S2 (40 mM). (E): Fluorescence spectra of Me-Acr^+^-Mes with S1 (40 mM), S2 (40 mM) and collidine (10 mM) normalized to the emission of Me-Acr^+^-Mes at 530 nm. (H): Percentage of quenching calculated as (1 − *I*_quencher_/*I*_0_)·100, where *I*_quencher_ and *I*_0_ are the emission intensities of Me-Acr^+^-Mes with and without quencher.

In our previous investigation of the mechanism,^24^ we performed nanosecond transient absorption experiments (Fig. 3C and D) on a reference molecule (4-methylanisole) and a model alcohol substrate (S2, Fig. 4) to detect the products of the initial electron transfer. After reductive quenching of the Me-Acr*-Mes by one of the substrates, we expected the formation of the reduced Me-Acr˙-Mes radical, which is characterized by a lower absorbance ratio at 565/500 nm compared to the excited state, together with the methoxybenzene radical cation, which has a maximum of absorption around 450 nm.

Consequently, we could detect the spectral features of the acridine radical for both the 4-methylanisole and S2, but the positive contribution from the methoxybenzene radical cation was only observed for the 4-methylanisole (Fig. 3C). We therefore hypothesized that the methoxybenzene radical cation formed upon quenching of Me-Acr*-Mes by S2 had already been converted to another, spectroscopically silent species in the timeframe of the experiment. This conversion could either progress *via* an intramolecular PCET-based pathway to form an alkoxy radical intermediate or *via* direct fragmentation. For the 4-methylanisole, however, conversion of the methoxybenzene radical was not possible since these mechanistic pathways were not available. Hence, we were able to detect its signal 100s of ns after laser excitation. We thus believed that the methoxybenzene radical signal might have been detectable for the alcohol substrate S2 through investigation of earlier timescales after laser excitation.

To test our hypothesis, we performed femtosecond transient absorption (fs-TA) experiments on the same systems (Fig. 3A and B). The TA spectral evolution of Me-Acr^+^-Mes showed the expected^29^ initial singlet excited state converting into a charge transfer (CT) state of the photocatalyst (Me-Acr˙-Mes˙^+^) on a 10 ps timescale, followed by its conversion to the triplet excited state Me-Acr^+^-Mes (Fig. S3†). In the presence of substrate, the CT state reacted to form the methoxybenzene radical; see below for a more detailed description of these initial processes. As expected, when the transient absorption spectra were recorded at 5 ns after laser excitation, the 4-methylanisole showed the same features as at longer timescales reported before (≥100 ns, Fig. 3A and C).^24^ For the case of the alcohol substrate S2, we were finally able to detect the 450 nm signal of the methoxybenzene radical cation at 5 ns after laser excitation (Fig. 3B), confirming our hypothesis that such radical cation forms as a product of the initial quenching and disappears within a few 10s of ns.

To investigate the lifetime of the methoxybenzene radical of the alcohol substrate S2 and verify whether its disappearance is dependent upon the addition of base (collidine), we measured kinetic traces at 440 nm with increasing concentrations of collidine (Fig. 4A). Without any collidine in the sample mixture, the methoxybenzene radical cation signal disappears with a lifetime of approximately 230 ns. This lifetime measured in the absence of base can be assigned to either the direct fragmentation of the alcohol substrate to yield the C-centered radical, or to PCET oxidation of the alcohol, with proton transfer to, *e.g.*, traces of water, or a combination of both. However, upon the addition of increasing concentration of collidine, the lifetime of the methoxybenzene radical cation decreases. This is strong support for a PCET mechanism since the proton transfer step affects the rate of the electron transfer that consumes the methoxybenzene radical. From a Stern–Volmer analysis of the traces with increasing concentrations of collidine, we could assign a rate constant of 1·10^9^ M^−1^ s^−1^ for the PCET mechanism (Fig. 4B). This data shows that the PCET mechanism is not only active but kinetically outcompetes the direct fragmentation in the presence of the base. Moreover, the traces depicted in Fig. 4A show that the quenching of the methoxybenzene radical cation in the presence of base has a static component, due to the formation of a complex between substrate and base, and a dynamic component, due to the diffusional encounter of substrate and base. The initial drop in amplitude of the signal at *t* = 0, indicated by the double arrows in Fig. 4A, increases with increasing base concentration. At low concentrations, there is negligible complex formation, and the drop is mainly due to the intrinsic absorption differences of the charge-separated (Me-Acr˙-Mes + methoxybenzene˙^+^) and ground state molecules. The drop increases with increasing base, however, which reflects the static component of the quenching that occurs instantaneously (cannot be resolved by the time resolution of the instrument) and increases with increasing concentration of base. The slower decay of the signal, which can be fitted with a single-exponential decay, provides the quenching lifetimes as a function of base concentration and reflects the dynamic component of the quenching.

We then proceeded to investigate the mechanism of the reaction of Me-Acr^+^-Mes with substrate S1. From fluorescence quenching experiments we could observe that S1 is a good quencher of Me-Acr^+^-Mes, although not as good as S2 (Fig. 4E). As observed previously,^24^ collidine itself can quench the fluorescence of Me-Acr^+^-Mes, and adding collidine to the sample mixture of Me-Acr^+^-Mes and S1 shows only an additive effect to the quenching. Since the addition of base does not yield cooperative effects on the quenching by the substrate, we can conclude that even in the case of S1 (as shown previously for S2) the initial quenching is not due to a complex between substrate and base. This is consistent with methoxybenzene, and not the alcohol, being the primary quencher. Moreover, the fraction of quenching due to S1 is higher at longer wavelengths and shows a clear drop in the region of 450–500 nm (Fig. 4H). This agrees well with our previous study,^24^ and it has been shown that the shorter wavelength contribution to the fluorescence of Me-Acr^+^-Mes comes partially from the short-lived (*ca.* 5 ps) excited singlet state localized on the acridinium, while at longer wavelengths the contribution comes mainly from the longer-lived (*ca.* 6 ns) charge transfer state.^29^ It is thus reasonable that S1, due to its steric hindrance, is not prone to form a pre-association complex with Me-Acr^+^-Mes that would be necessary for efficient quenching of the short-lived singlet state. As for S2, Me-Acr^+^-Mes oxidizes S1 primarily in its CT state while the triplet state does not react.

**Fig. 5:**
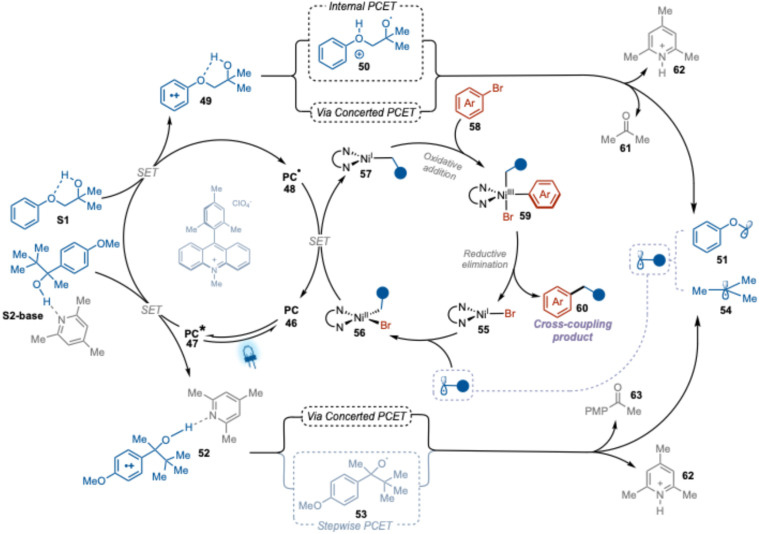
Proposed mechanism of PCET photoredox nickel dual catalytic cross-coupling arylation of alcohol.

The substrate S1 differs partially from S2 when it comes to the TA investigation. Similarly to S2, we were not able to detect the positive signal of the methoxybenzene radical at 450 nm for S1 after 100s of ns, while the spectral features of the Me-Acr˙-Mes radical were evident (see change in 565/500 nm ratio on Fig. S2 right in the ESI†). This made us once again hypothesize a fast intramolecular radical transfer where the methoxybenzene radical cation is converted to another species on this timescale. However, even with fs-TA measurements, we were not able to detect the 450 nm signal of the methoxybenzene radical cation of S1, as opposed to the case of S2 (see Fig. S2 left in the ESI†). Hence, we hypothesize that for S1 the fragmentation pathway is much faster than for S2, making it impossible to detect the methoxybenzene radical cation that should form upon quenching of the Me-Acr^+^-Mes. This could be due to a stronger electronic coupling between the arene unit and the hydroxyl group, which would facilitate through-bond hole transfer. Alternatively, a fragmentative internal PCET where the proton transfers from the hydroxyl group to the ether oxygen leading to the formation of acetone could be envisioned (S1 to 61 in Fig. 5). To complete the analysis of the quenching of Me-Acr^+^-Mes by S2 and S1, we looked at the kinetic traces of absorption changes at 480 nm at early timescales (ps–ns) after laser excitation (Fig. 4C, D, F & G). At this wavelength, all three excited states of Me-Acr^+^-Mes (locally excited singlet, charge transfer, and locally excited triplet) absorb, with different extinction coefficients. Upon addition of S1, the locally excited singlet state lifetime remains almost unperturbed (Fig. 4C), while it is slightly decreased in the presence of S2 (Fig. 4F).

The subsequent decay of the 480 nm signal on the ns timescale is then accelerated in the presence of the substrates (Fig. 4D and G), which indicates the quenching of the charge transfer state.

From the mechanistic investigation, we were able to conclude that the PCET pathway for S2 is active and can outcompete the direct fragmentation in the presence of base, with a rate constant on the order of 10^9^ M^−1^ s^−1^, *i.e.*, almost diffusion-controlled, and an even faster component due to preformed substrate–base complexes. For S1, we hypothesize a faster fragmentation mechanism, possibly due to the favored O-mediated hole transfer to the alcohol unit.

Based on our mechanistic study results and reported literature on the photoredox nickel dual catalytic cross-coupling arylation reactions,^30^ we have postulated a plausible mechanism for our PCET-mediated photoredox nickel dual catalytic cross-coupling alcohol arylation method (Fig. 5).

Upon visible light irradiation, the ground state of the Me-Acr^+^-Mes photocatalyst (PC, 46) is excited to a highly oxidizing singlet state that subsequently evolves to a CT state (PC*, 47). Reductive quenching of the CT state by substrates S1 or S2-base generates the reduced form of photocatalyst (PC˙, 48) and oxidized form of these substrates (49 or 52). The progression of the transformation then depends on the nature of the substrate. The oxidized substrate 49 will generate the key alkyl radical 51*via* an internal PCET process involving the generation of an O-centered radical intermediate (50), or *via* a concerted PCET process. On the other hand, the acid–base complex of oxidized substrate 52 would most likely generate alkyl radical 54 through concerted PCET, where deportation and β-scission occur simultaneously. This is in agreement with previously calculated reactions barriers.^24^ The so-formed radicals will be captured by Ni(i)–Br complex (55) to form a Ni(ii)-alkyl-bromo complex (56). Single electron transfer between the reduced photocatalyst (PC˙, 48) and the complex 56 will close the catalytic cycle of the photocatalyst (46) and generate a Ni(i)-alkyl complex (57). Oxidative addition of the aryl halide to this complex yields Ni(iii)-alkyl-aryl-bromo complex 59, which undergoes reductive elimination to form the final desired cross-coupling product 60 and a Ni(i)-bromo species (55), thereby closing the catalytic cycle of the nickel catalyst.

## Conclusions

In summary, we have demonstrated a practical and efficient method for the deconstructive arylation of aliphatic alcohols through a synergistic photoredox PCET and nickel dual catalytic process. By generating alkyl radicals *via* PCET-mediated β-scission, our approach facilitates the formation of C(sp^3^)–C(sp^2^) bonds between free alcohols and aryl halides under mild conditions. This method not only broadly expands the scope of alcohol-derived radical precursors but also improves the efficiency of cross-coupling reactions, particularly with challenging tertiary alcohols. Mechanistic investigations utilizing femtosecond and nanosecond transient absorption spectroscopy provided key insights into the PCET pathway, revealing its critical role in outcompeting direct fragmentation processes. Our protocol broadens the utility of alcohols in cross-coupling chemistry, offering a scalable and versatile strategy for the synthesis of complex, structurally diverse molecules.

## Data availability

The data supporting this article have been included as part of the ESI.†

## Author contributions

C. J. W. and L. H. supervised the overall project and secured the funding. C. J. W. and Y. P. conceived the idea and designed the study with L. H. and R. C. The optimization is done by Y. P. and B. M. B. Synthesis, purification and characterizations are done by Y. P. and K. B. P. R. C. conducted the mechanistic study. A. B. helped with the femtosecond transient absorption instrumentation. Y. P., R. C. and C. J. W. co-wrote the manuscript. All authors have given approval to the final version of the manuscript.

## Conflicts of interest

The authors declare no competing financial interest.

## Supplementary Material

SC-OLF-D5SC00737B-s001
