# Supplementary material for: PCET-mediated deconstructive cross-coupling of aliphatic alcohols
Source: Chem Sci. 2025 Mar 27;16(18):7720–9. doi: 10.1039/d5sc00737b (PMC11962747; doi:10.1039/d5sc00737b)
Supplement: SC-016-D5SC00737B-s001 [file SC-016-D5SC00737B-s001.pdf]

Supplementary Information (SI) for Chemical Science.
This journal is © The Royal Society of Chemistry 2025

Supporting Information belonging to the manuscript:

PCET-Mediated Deconstructive Cross-Coupling of Aliphatic Alcohols

Yeersen Patehebieke,[1‡] Rima Charaf,[2‡] Kumar Bhaskar Pal,[1] Beatriz Meana Baamonde,[1] Andjela Brnovic,[2] Leif Hammarström,[2*] and Carl-Johan Wallentin[1*]

[1]Department of Chemistry and Molecular Biology, University of Gothenburg, Gothenburg SE 41296, Sweden.

[2]Department of Chemistry - Ångström Laboratory, Uppsala University, Uppsala SE 75120, Sweden.

[‡]These authors contributed equally

*Email: carl.wallentin@chem.gu.se

leif.hammarstrom@kemi.uu.se

Table of Contents

GENERAL INFORMATION **3**

PHOTO-REDOX REACTION STANDARD REACTION SET-UP **4**

SYNTHESIS OF $NIBR_2$•DTBBPY CATALYST **4**

OPTIMIZATION OF REACTION CONDITIONS **5**

GENERAL EXPERIMENTAL PROCEDURE FOR PHOTOREACTION (GP1) **8**

PREPARATION AND CHARACTERIZATION OF CROSS-COUPLING REACTION PRODUCTS SCOPE **8**

SYNTHESIS & CHARACTERIZATION OF STARTING MATERIALS **22**

- GENERAL PROCEDURE 2 FOR THE SYNTHESES OF ALCOHOLS (GP2) 22
- GENERAL PROCEDURE 3 FOR THE SYNTHESES OF ALCOHOLS (GP3) 23
- GENERAL PROCEDURE 4 FOR THE SYNTHESES OF ALCOHOLS (GP4) 23
- PREPARATION AND CHARACTERIZATION OF ALCOHOLS 23

MECHANISTIC STUDY EXPERIMENTS **36**

^{1}H NMR AND ^{13}C NMR SPECTRA **40**

General information

All reactions were carried out in oven-dried microwave tubes (T_{oven} = 150 °C) by using Schlenk techniques and syringes under nitrogen atmosphere. Commercial grade reagents were purchased from Sigma-Aldrich, Fluorochem and VWR Chemicals and used as supplied without further purification, unless otherwise indicated. For thin-layer chromatography (TLC) silica gel coated aluminium plates (60, F_{254}, Merck) were employed and analysed with UV light at 254 or 365 nm and by means of staining with anisaldehyde or $KMnO_4$ stains. The purification of products was performed on silica gel 60 M (40 – 63 µm, VWR Chemicals) by using the flash technique under a pressure of 2 bar or with Büchi Pure C-810 Flash (using Büchi FlashPure EcoFlex cartridges of either 50 µm or 20 µm).

1,2-dichloroethane (DCE) used for photoredox reactions were filtered through neutral Al_2O_3 and degassed prior to use. Other common dry solvents obtained from a solvent purification system or purchased from Sigma-Aldrich and used as supplied without further purification, unless otherwise indicated.

GC-MS(EI) was performed on Agilent Technologies 7820A gas chromatograph with an Agilent 5977E MSD detector. Separations were performed on Agilent 19091S-433 J&W HP-5ms GC Column, 30 m, 0.25 mm, 0.25 µm, 7-inch cage.

HRMS data were recorded with a QExactive HF Orbitrap mass spectrometer interfaced with Dionex Ultimate 3000 liquid chromatography system (Thermo Fisher Scientific). The instrument operated in full MS mode only, where the ion mass spectra were acquired at a resolution of 120 000, maximum injection time 200 ms for 3e6 ions. The Orbitrap was calibrated with Pierce LTQ ESI Positive Ion Calibration Solution prior to the analysis, resulting in mass accuracy better than 5 ppm. Electrospray ionization were performed at 4 kV and 320 degree Celsius using a metal emitter in the ion source. The sample (10 mikroliter) was injected onto a reversed-phase XBridge BEH C18 column (3.5 µm, 2.1x50 mm, Waters Corporation). The analysis was performed using a linear gradient from 10% solvent B (80% acetonitrile in water and 0.1% formic acid) to 100% solvent B over 2.5 min followed by 100% during 17.5 min with flow of 0.300 ml/min and solvent A being 0.1% formic acid in water.

^{1}H-NMR and ^{13}C-NMR spectra were recorded on a Varian NMR 400 or Bruker Avance III HD 600 & 800 MHz spectrometer. Chemical shifts (δ) are reported in ppm relative to the residual solvent peak, and solvent signal was used as the reference for ^{1}H NMR ($CDCl_3$, 7.26 ppm, CD_3OD, 3.31 ppm, CD_3COCD_3, 2.05 ppm) and ^{13}C NMR ($CDCl_3$, 77.16 ppm, CD_3OD, 49.00 ppm, CD_3COCD_3, 206.26, 29.84 ppm). Splitting patterns are indicated as (s) singlet, (d) doublet, (dd) doublet of doublets, (t) triplet, (tt) triplet of triplets, (dt) doublet of triplets, (td) triplet of doublets, (q) quartet, (quint) quintet, (sext) sextet, (hept) heptet, (m) multiplet. Coupling constants (*J*) are reported in Hertz (Hz).

Photo-redox reaction standard reaction set-up

Photo-redox reactions were performed in a Biotage® Microwave Reaction Vials using Lucent 360 photo reactor. 450 nm Light with 50 % intensity. The reaction temperature maintained at 35 ºC by Grant TC120 series of digital heating circulating bath connected to Lucent 360 photo reactor.

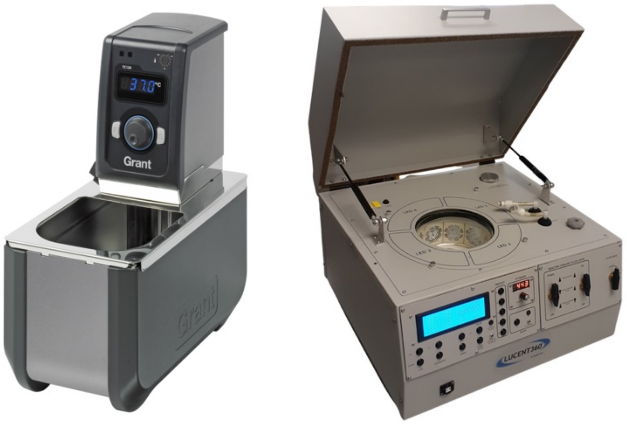

Figure S1. Photo reactor setup

Synthesis of $NiBr_2$·dtbbpy catalyst

$NiBr_2$•dtbbpy was synthesized following a previously reported protocol.[1] A flame-dried 50 mL flask equipped with stir bar was charged with $NiBr_2$•$3H_2O$ (981.18 mg, 3.6 mmol, 1 equiv.), 4,4'-Di-tert-butyl-2,2'-dipyridyl (dtbbpy) (1.07 g, 4.0 mmol, 1.1 equiv.). The system was degassed, and then methanol (15 mL) was added under a nitrogen atmosphere. The flask was then placed in an oil bath (70 °C) and refluxed overnight. After cooling to room temperature, the solvent was concentrated in vacuum to ~10% of its original volume. A 1:4 mixture of THF: diethyl ether (20 mL) was added to the residue, and the mixture was stirred for 3 h under nitrogen. The resulting mixture was then filtered and washed with some diethyl ether. Then dried under vacuum to give the light green solid: 1.32 g, **75 %** yield. The Ni precatalyst was bench-stable was stored in a desiccator.

Optimization of reaction conditions

Table S1. Optimization of solvent and ligand[a], [b], [c], [d]

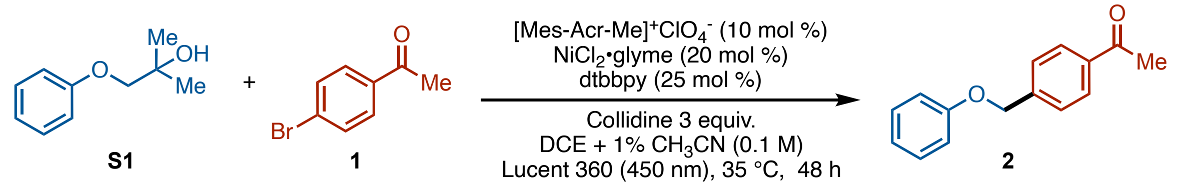

entry	solvent	photo catalyst	base	ligand	yield (%)
1	DCE	$[Mes\text{-}Acr\text{-}Me]^+ClO_4^-$	collidine	dtbbpy	69 (57[e])
2	DCE	$[Mes\text{-}Acr\text{-}Me]^+ClO_4^-$	collidine	dmobpy	53
3	1,2-dichlorobenzene	$[Mes\text{-}Acr\text{-}Me]^+ClO_4^-$	collidine	dtbbpy	n,d.
4	1,2-difluorobenzene	$[Mes\text{-}Acr\text{-}Me]^+ClO_4^-$	collidine	dtbbpy	68
5	1,4-difluorobenzene	$[Mes\text{-}Acr\text{-}Me]^+ClO_4^-$	collidine	dtbbpy	2
6	Acetone	$[Mes\text{-}Acr\text{-}Me]^+ClO_4^-$	collidine	dtbbpy	n,d.
7	Acetonitrile	$[Mes\text{-}Acr\text{-}Me]^+ClO_4^-$	collidine	dtbbpy	8
8	Cyclohexane	$[Mes\text{-}Acr\text{-}Me]^+ClO_4^-$	collidine	dtbbpy	n,d.
9	DCM	$[Mes\text{-}Acr\text{-}Me]^+ClO_4^-$	collidine	dtbbpy	30
10	DMF	$[Mes\text{-}Acr\text{-}Me]^+ClO_4^-$	collidine	dtbbpy	n,d.
11	EtOAc	$[Mes\text{-}Acr\text{-}Me]^+ClO_4^-$	collidine	dtbbpy	n,d.
12	Fluorobenzene	$[Mes\text{-}Acr\text{-}Me]^+ClO_4^-$	collidine	dtbbpy	n,d.
13	Fluorotoluene	$[Mes\text{-}Acr\text{-}Me]^+ClO_4^-$	collidine	dtbbpy	2
14	Perfluorooctane	$[Mes\text{-}Acr\text{-}Me]^+ClO_4^-$	collidine	dtbbpy	2
15	DCE + 1% ACN	$[Mes\text{-}Acr\text{-}Me]^+ClO_4^-$	collidine	dtbbpy	84 (75[e])
16	DCE + 5 % ACN	$[Mes\text{-}Acr\text{-}Me]^+ClO_4^-$	collidine	dtbbpy	62
17	DCE + 1% DMF	$[Mes\text{-}Acr\text{-}Me]^+ClO_4^-$	collidine	dtbbpy	38
18	1,2-difluorobenzene + 1% ACN	$[Mes\text{-}Acr\text{-}Me]^+ClO_4^-$	collidine	dtbbpy	27

[a] Reactions were performed according to the procedure described in the section **General experimental procedure for photoreaction (GP1)**. [b] Standard conditions: alcohol **S1** (0.3 mmol), aryl bromide **1** (0.1 mmol), 2,4,6-collidine (0.3 mmol), $[Mes\text{-}Acr\text{-}Me]^+ClO_4^-$ (0.01 mmol), $NiCl_2$•glyme (0.02 mmol), dtbbpy (0.025 mmol), DCE + 1 % CH_3CN (3 mL), time (48 h), photo reactor (Lucent 360, 450 nm, 50 % light intensity), temperature (35°C); [c] Yields are determined by ^1H NMR using ethylene carbonate as internal standard; [d] Dimethoxybypyridyl = dmobpy; 4,4′-Di-tert-butyl-2,2′-dipyridyl = dtbbpy; [e]: Isolated yield;

Table S2. Optimization of base and photocatalyst[a], [b], [c], [d]

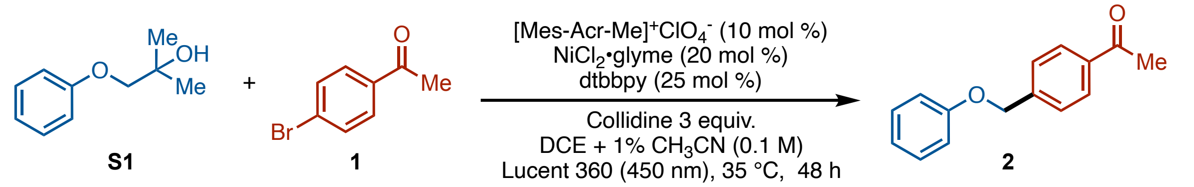

entry	solvent	photo catalyst	base	yield (%)
1	DCE + 1% ACN	$[Mes\text{-}Acr\text{-}Me]^+ClO_4^-$	collidine	84 (75[e])
2	DCE + 1% ACN	$[Mes\text{-}Acr\text{-}Me]^+ClO_4^-$	DBU	n,d.
3	DCE + 1% ACN	$[Mes\text{-}Acr\text{-}Me]^+ClO_4^-$	$(n\text{-}Bu)_4\ P^+(PhO)_2(O)PO^-$	11
4	DCE + 1% ACN	$[Mes\text{-}Acr\text{-}Me]^+ClO_4^-$	$(n\text{-}Bu)_4\ P^+(O)CF_3O^-$	12
5	DCE + 1% ACN	$[Mes\text{-}Acr\text{-}Me]^+ClO_4^-$	4-methoxy-2,6-dimethyl pyridine	61
6	DCE + 1% ACN	$[Mes\text{-}Acr\text{-}Me]^+ClO_4^-$	Barton's base	n,d.
7	DCE + 1% ACN	$[Mes\text{-}Acr\text{-}Me]^+ClO_4^-$	1,1,3,3-tetramethylguanidine	n,d.
8	DCE + 1% ACN	$[Mes\text{-}Acr\text{-}Me]^+ClO_4^-$	2,6-dimethyl-4-dimethylaminopyridine	n,d.
9	DCE + 1% ACN	$[Mes\text{-}Acr\text{-}Me]^+BF_4^-$	collidine	54
10	DCE + 1% ACN	2,4,6-TPPP	collidine	4
11	DCE + 1% ACN	$[Mes\text{-}Acr\text{-}Ph]^+BF_4^-$	collidine	21

12	DCE + 1% ACN	[Ir(dF(CF$_3$)ppy)$_2$(5,5'-dCF$_3$bpy)]PF$_6$	collidine	14
13	DCE + 1% ACN	[Ir(dF(CH$_3$)ppy)$_2$(dtbbpy)]PF$_6$	collidine	n,d.
14	PhCF$_3$	[Ir(dF(CF$_3$)ppy)$_2$(5,5'-dCF$_3$bpy)]PF$_6$	(*n*-Bu)$_4$ P$^+$(PhO)$_2$(O)PO$^-$	28
15	PhCF$_3$	[Ir(dF(CF$_3$)ppy)$_2$(5,5'-dCF$_3$bpy)]PF$_6$	(*n*-Bu)$_4$ P$^+$(O)CF$_3$O$^-$	21
16	1,2-difluorobenzene	[Ir(dF(CF$_3$)ppy)$_2$(5,5'-dCF$_3$bpy)]PF$_6$	(*n*-Bu)$_4$ P$^+$(PhO)$_2$(O)PO$^-$	25
17	DCE + 1% ACN	[Ir(dF(CF$_3$)ppy)$_2$(5,5'-dCF$_3$bpy)]PF$_6$	(*n*-Bu)$_4$ P$^+$(PhO)$_2$(O)PO$^-$	17

[a] Reactions were performed according to the procedure described in the section **General experimental procedure for photoreaction (GP1)**. [b] Standard conditions: alcohol **S1** (0.3 mmol), aryl bromide **1** (0.1 mmol), 2,4,6-collidine (0.3 mmol), [Mes-Acr-Me]$^+$ClO$_4^-$ (0.01 mmol), NiCl$_2$•glyme (0.02 mmol), dtbbpy (0.025 mmol), DCE + 1 % CH$_3$CN (3 mL), time (48 h), photo reactor (Lucent 360, 450 nm, 50 % light intensity), temperature (35°C); [c] Yields are determined by ^{1}H NMR using ethylene carbonate as internal standard; [d] 4,4′-Di-tert-butyl-2,2′-dipyridyl = dtbbpy; [e]: Isolated yield;

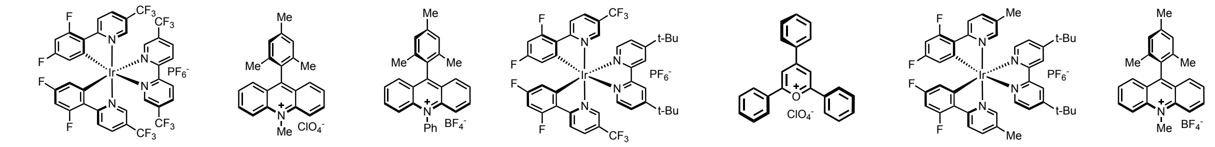

[Ir{dF(CF$_3$)ppy}$_2$(5,5'-dCF$_3$bpy)]$^+$PF$_6^-$ | [Mes-Acr-Me]$^+$ClO$_4^-$ Fukuzumi catalysts | [Mes-Acr-Ph]$^+$BF$_4^-$ | [Ir{dF(CF$_3$)ppy}$_2$(dtbbpy)]$^+$PF$_6^-$ | 2,4,6-TPPP 2,4,6-Triphenylpyrylium perchlorate | [Ir{dF(Me)ppy}$_2$(dtbbpy)]$^+$PF$_6^-$ | [Mes-Acr-Me]$^+$BF$_4^-$

Table S3. Optimization of nickel catalyst, reaction time, and concentration[a], [b], [c], [d]

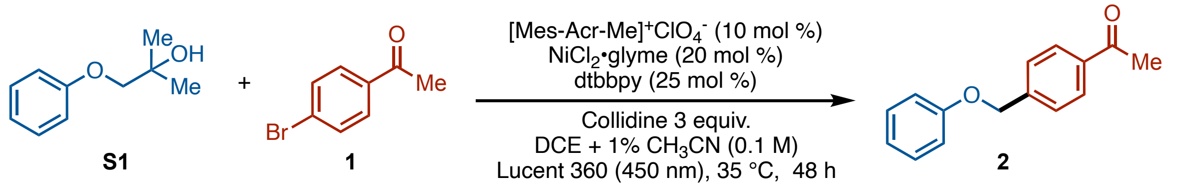

entry	Nickel catalyst (mol %)	solvent	concentration	time	yield (%)
1	NiCl$_2$·glyme (20)	DCE + 1% ACN	0.1 M	48 h	84 (75[e])
2	NiBr$_2$·glyme, (20)	DCE + 1% ACN	0.1 M	48 h	72
3	Ni(II) nitrate hexahydrate,(20)	DCE + 1% ACN	0.1 M	48 h	19
4	Ni(II) hexafluoroacetyl acetone hydrate, (20)	DCE + 1% ACN	0.1 M	48 h	n,d.
5	Ni(II) acetate tetrahydrate, (20)	DCE + 1% ACN	0.1 M	48 h	2
6	Ni(II) acetyl acetonate, (20)	DCE + 1% ACN	0.1 M	48 h	59
7	NiBr$_2$·dtbbpy, (20)	DCE + 1% ACN	0.1 M	48 h	78
8	NiCl$_2$·glyme (20)	DCE + 1% ACN	0.1 M	24 h	53
9	NiCl$_2$·glyme (20)	DCE + 1% ACN	0.1 M	12 h	46
10	NiCl$_2$·glyme (20)	DCE + 1% ACN	0.1 M	6 h	37
11	NiCl$_2$·glyme (15)	DCE	0.1 M	48 h	39
12	NiCl$_2$·glyme (20)	DCE	0.05 M	48 h	49
13	NiCl$_2$·glyme (20)	DCE	0.2 M	48 h	53

[a] Reactions were performed according to the procedure described in the section **General experimental procedure for photoreaction (GP1).** [b] Standard conditions: alcohol **S1** (0.3 mmol), aryl bromide **1** (0.1 mmol), 2,4,6-collidine (0.3 mmol), [Mes-Acr-Me]$^+$ClO$_4^-$ (0.01 mmol), NiCl$_2$•glyme (0.02 mmol), dtbbpy (0.025 mmol), DCE + 1 % CH$_3$CN (3 mL), time (48 h), photo reactor (Lucent 360, 450 nm, 50 % light intensity), temperature (35°C); [c] Yields are determined by ^{1}H NMR using ethylene carbonate as internal standard; [d] 4,4′-Di-tert-butyl-2,2′-dipyridyl = dtbbpy; [e]: Isolated yield;

Table S4. Optimization of light intensity[a], [b], [c], [d]

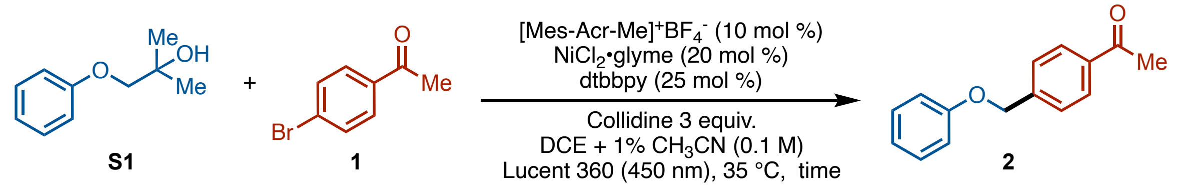

entry	light intensity	photo catalyst	time	yield (%)
1	50 %	$[Mes\text{-}Acr\text{-}Me]^+BF_4^-$	12 h	30 %
2	100 %	$[Mes\text{-}Acr\text{-}Me]^+BF_4^-$	12 h	33 %
3	50 %	$[Mes\text{-}Acr\text{-}Me]^+BF_4^-$	24 h	46 %
4	100 %	$[Mes\text{-}Acr\text{-}Me]^+BF_4^-$	24 h	49 %
5	50 %	$[Mes\text{-}Acr\text{-}Me]^+BF_4^-$	48 h	54 %
6	100 %	$[Mes\text{-}Acr\text{-}Me]^+BF_4^-$	48 h	44 %

[a] Reactions were performed according to the procedure described in the section **General experimental procedure for photoreaction (GP1)**. [b] Standard conditions: alcohol **S1** (0.3 mmol), aryl bromide **1** (0.1 mmol), 2,4,6-collidine (0.3 mmol), $[Mes\text{-}Acr\text{-}Me]^+BF_4^-$ (0.01 mmol), $NiCl_2$•glyme (0.02 mmol), dtbbpy (0.025 mmol), DCE + 1 % CH_3CN (3 mL), temperature (35°C); [c] Yields are determined by ^1H NMR using ethylene carbonate as internal standard; [d] 4,4′-Di-tert-butyl-2,2′-dipyridyl = dtbbpy;

Table S5. Optimization of alcohol equivalence[a], [b], [c], [d]

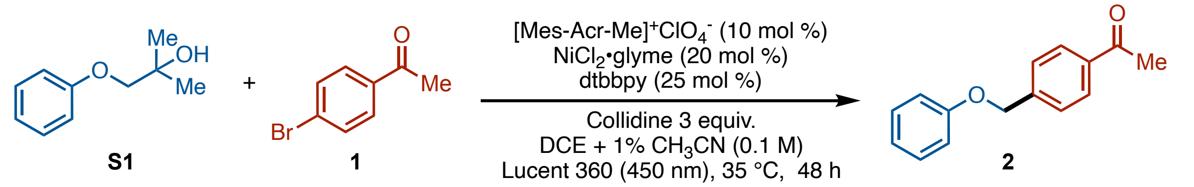

entry	Nickel catalyst (mol %)	Alcohol equiv.	time	yield (%)
1	$NiCl_2$·glyme (20)	3 equiv.	48 h	84 (75[e])
2	$NiCl_2$·glyme (20)	2 equiv.	48 h	35
3	$NiCl_2$·glyme (20)	1 equiv.	48 h	13

[a] Reactions were performed according to the procedure described in the section **General experimental procedure for photoreaction (GP1).** [b] Standard conditions: alcohol **S1** (0.3 mmol), aryl bromide **1** (0.1 mmol), 2,4,6-collidine (0.3 mmol), $[Mes\text{-}Acr\text{-}Me]^+ClO_4^-$ (0.01 mmol), $NiCl_2$•glyme (0.02 mmol), dtbbpy (0.025 mmol), DCE + 1 % CH_3CN (3 mL), time (48 h), photo reactor (Lucent 360, 450 nm, 50 % light intensity), temperature (35°C); [c] Yields are determined by ^1H NMR using ethylene carbonate as internal standard; [d] 4,4′-Di-tert-butyl-2,2′-dipyridyl = dtbbpy; [e]: Isolated yield;

Table S6. Some unsuccessful aromatic coupling partners

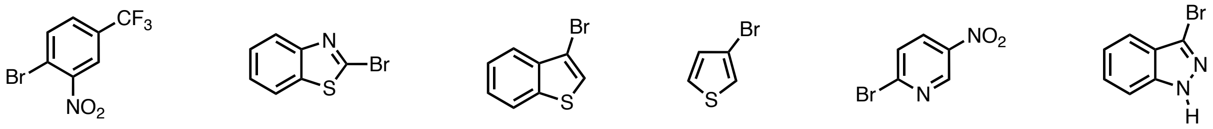

Trace	14 %	Trace	11 %	Trace	Trace

General experimental procedure for photoreaction (GP1)

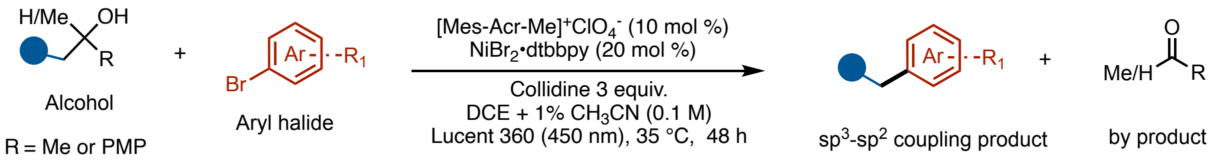

Anhydrous 1,2-Dichloroethane (DCE) and acetonitrile was deoxygenated by sparging with N_2 for 20 min. Fukuzumi catalyst (4.2 mg, 0.01mmol, 10 mol%), alcohol (0.3 mmol, 3.0 equiv.), $NiBr_2$•dtbbpy (9.7 mg, 0.02 mmol, 20 mol%), (if solid) the aryl halide (0.1 mmol, 1.0 equiv.) and a stir bar were added to an oven dried Biotage MW vial (10 mL). The microwave vial was then capped tightly, and vacuum purged 3 times with N_2 gas. After filling with N_2 the third time, anhydrous acetonitrile (30 µL, 1 %), (if liquid) aryl halide (0.1 mmol, 1.0 equiv.), anhydrous DCE (3 mL), and lastly, collidine (0.3 mmol, 3.0 equiv.) was added by syringe. Reaction atmosphere then exchanged to N_2 by "sparging" head space for 5 min. The capped end of the vial was wrapped with parafilm and placed in Lucent 360 photoreactor set up (as illustrated in Figure 1) then stirred under direct blue LED irradiation at 450 nm for 48 hours. The reaction temperature maintained at 35 ºC with Grant TC120 series of digital heating circulating bath connected to Lucent 360 photo reactor.

The two duplicated reactions were combined, and the crude solution was concentrated and purified by Büchi Pure C-810 Flash (using Büchi FlashPure EcoFlex cartridges of either 50 µm or 20 µm) with Petroleum spirit and 3 % EtOAc as eluent mixture. Isolated yields were thereby obtained as an average. Compounds on TLC-plates were visualized with UV (254 nm), anisaldehyde or $KMnO_4$ stains.

Preparation and characterization of cross-coupling reaction products scope

1-(4-(phenoxymethyl)phenyl)ethan-1-one (2)

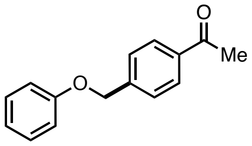

Compound (**2**) was synthesized following GP1 using alcohol **S1** (49.9 mg, 0.3 mmol), 4-bromoacetophenone (19.9 mg, 0.10 mmol), $NiCl_2$•glyme (4.4 mg, 0.02 mmol), dtbbpy (6.7 mg, 0.025 mmol), [Mes-Acr-Me]$^+$$ClO_4^-$ (4.2 mg 0.01 mmol), and 2,4,6-collidine (40 mL, 0.30 mmol). After 48 h, the reaction produced the desired compound as a white solid (16.7 mg, **75 %** yield) after flash chromatography (petroleum spirit /EtOAc 95:5). Spectroscopic data agrees with the reported literature.[2]

^{1}H NMR (400 MHz, $CDCl_3$) δ 7.98 (d, *J* = 8.3 Hz, 2H), 7.53 (d, *J* = 8.5 Hz, 2H), 7.36 – 7.25 (m, 2H), 7.03 – 6.93 (m, 3H), 5.14 (s, 2H), 2.61 (s, 3H) ppm.

^{13}C NMR (101 MHz, $CDCl_3$) δ 197.7, 158.4, 142.5, 136.6, 129.6, 128.6, 127.1, 121.2, 114.8, 69.2, 26.7 ppm

(4-(phenoxymethyl)phenyl)(phenyl)methanone (3)

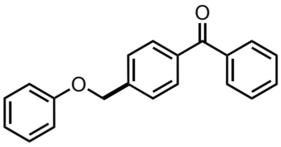

Compound (**3**) was synthesized following GP1 using alcohol **S1** (49.9 mg, 0.3 mmol), aryl bromide (26.1 mg, 0.10 mmol), $NiCl_2$•glyme (4.4 mg, 0.02 mmol), dtbbpy (6.7 mg, 0.025 mmol), [Mes-Acr-Me]$^+$$ClO_4^-$ (4.2 mg 0.01 mmol), and 2,4,6-collidine (40 mL, 0.30 mmol). After 48 h, the reaction

produced the desired compound as a white solid (16.6 mg, **56 %** yield) after flash chromatography (petroleum spirit /EtOAc 95:5). Spectroscopic data agrees with the reported literature.[3]

^{1}H NMR (400 MHz, $CDCl_3$) δ 7.86 – 7.78 (m, 4H), 7.63 – 7.53 (m, 3H), 7.53 – 7.45 (m, 2H), 7.35 – 7.28 (m, 2H), 7.02 – 6.96 (m, 3H), 5.17 (s, 2H) ppm.

^{13}C NMR (101 MHz, $CDCl_3$) δ 196.5, 158.6, 142.0, 137.7, 137.2, 132.6, 130.6, 130.2, 129.7, 128.4, 127.1, 121.4, 115.0, 69.4 ppm.

1-(phenoxymethyl)-4-(trifluoromethyl)benzene (4)

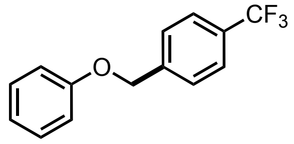

Compound (**4**) was synthesized following GP1 using alcohol **S1** (49.9 mg, 0.3 mmol), aryl bromide (22.5 mg, 0.10 mmol), $NiCl_2$•glyme (4.4 mg, 0.02 mmol), dtbbpy (6.7 mg, 0.025 mmol), [Mes-Acr-Me]$^+ClO_4^-$ (4.2 mg 0.01 mmol), and 2,4,6-collidine (40 mL, 0.30 mmol). After 48 h, the reaction produced the desired compound as a white solid (10.5 mg, **81 %** yield) after flash chromatography (petroleum spirit /EtOAc 95:5). Spectroscopic data agrees with the reported literature.[3]

^{1}H NMR (400 MHz, $CDCl_3$) δ 7.65 (d, J = 8.0 Hz, 2H), 7.56 (d, J = 8.0 Hz, 2H), 7.34 – 7.28 (m, 2H), 7.02 – 6.94 (m, 3H), 5.14 (s, 2H) ppm.

^{13}C NMR (151 MHz, $CDCl_3$) δ 158.5, 141.3, 130.3 (q, $J_{C,F}$ = 32.7 Hz), 129.7, 127.5, 125.7 (q, $J_{C,F}$ = 3.8 Hz), 124.4 (q, $J_{C,F}$ = 272.4 Hz), 121.5, 115.0, 69.2 ppm.

Methyl 4-(phenoxymethyl)benzoate (5)

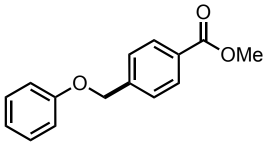

Compound (**5**) was synthesized following GP1 using alcohol **S1** (49.9 mg, 0.3 mmol), aryl bromide (21.5 mg, 0.10 mmol), $NiCl_2$•glyme (4.4 mg, 0.02 mmol), dtbbpy (6.7 mg, 0.025 mmol), [Mes-Acr-Me]$^+ClO_4^-$ (4.2 mg 0.01 mmol), and 2,4,6-collidine (40 mL, 0.30 mmol). After 48 h, the reaction produced the desired compound as a Colorless oil (9.4 mg, **39 %** yield) after flash chromatography (petroleum spirit /EtOAc 95:5). Spectroscopic data agrees with the reported literature.[3]

^{1}H NMR (400 MHz, $CDCl_3$) δ 8.10 – 8.02 (m, 2H), 7.51 (d, J = 8.1 Hz, 2H), 7.34 – 7.27 (m, 2H), 7.01 – 6.94 (m, 3H), 5.13 (s, 2H), 3.92 (s, 3H) ppm.

^{13}C NMR (101 MHz, $CDCl_3$) δ 167.0, 158.6, 142.5, 130.0, 129.8, 129.7, 127.1, 121.4, 115.0, 69.4, 52.3 ppm.

4-(phenoxymethyl)benzaldehyde (6)

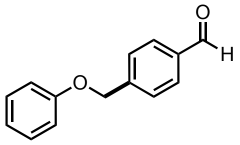

Compound (**6**) was synthesized following GP1 using alcohol **S1** (49.9 mg, 0.3 mmol), aryl bromide (18.5 mg, 0.10 mmol), $NiCl_2$•glyme (4.4 mg, 0.02 mmol), dtbbpy (6.7 mg, 0.025 mmol), [Mes-Acr-Me]$^+ClO_4^-$ (4.2 mg 0.01 mmol), and 2,4,6-collidine (40 mL, 0.30 mmol). After 48 h, the reaction produced the desired compound as a white solid (8.8 mg, **43 %** yield) after flash chromatography (petroleum spirit /EtOAc 95:5). Spectroscopic data agrees with the reported literature.[3]

^{1}H NMR (400 MHz, $CDCl_3$) δ 10.03 (s, 1H), 7.94 – 7.88 (m, 2H), 7.61 (d, J = 7.9 Hz, 2H), 7.34 – 7.27 (m, 2H), 7.01 – 6.95 (m, 3H), 5.16 (s, 2H) ppm.

^{13}C NMR (101 MHz, $CDCl_3$) δ 192.0, 158.5, 144.2, 136.1, 130.2, 129.7, 127.6, 121.5, 114.9, 69.3 ppm.

4-(phenoxymethyl)benzonitrile (7)

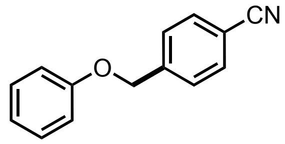

Compound (**7**) was synthesized following GP1 using alcohol **S1** (49.9 mg, 0.3 mmol), aryl bromide (18.2 mg, 0.10 mmol), $NiCl_2$•glyme (4.4 mg, 0.02 mmol), dtbbpy (6.7 mg, 0.025 mmol), [Mes-Acr-Me]$^+ClO_4^-$ (4.2 mg 0.01 mmol), and 2,4,6-collidine (40 mL, 0.30 mmol). After 48 h, the reaction produced the desired compound as a white solid (14.4 mg, **69 %** yield) after flash chromatography (petroleum spirit /EtOAc 95:5). Spectroscopic data agrees with the reported literature.[3]

^{1}H NMR (400 MHz, $CDCl_3$) δ 7.71 – 7.65 (m, 2H), 7.58 – 7.52 (m, 2H), 7.35 – 7.27 (m, 2H), 7.03 – 6.92 (m, 3H), 5.13 (s, 2H) ppm.

^{13}C NMR (101 MHz, $CDCl_3$) δ 158.3, 142.7, 132.5, 129.8, 127.7, 121.6, 118.9, 114.9, 111.8, 68.9 ppm.

4-((4-(phenoxymethyl)phenyl)sulfonyl)morpholine (8)

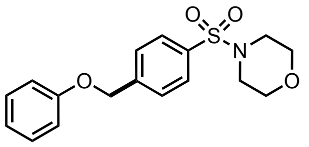

Compound (**8**) was synthesized following GP1 using alcohol **S1** (49.9 mg, 0.3 mmol), aryl bromide (30.6 mg, 0.10 mmol), $NiCl_2$•glyme (4.4 mg, 0.02 mmol), dtbbpy (6.7 mg, 0.025 mmol), [Mes-Acr-Me]$^+ClO_4^-$ (4.2 mg 0.01 mmol), and 2,4,6-collidine (40 mL, 0.30 mmol). After 48 h, the reaction produced the desired compound as a white solid (22.6 mg, **68 %** yield) after flash chromatography (petroleum spirit /EtOAc 95:5). Spectroscopic data agrees with the reported literature.[3]

^{1}H NMR (600 MHz, $CDCl_3$) δ 7.81 – 7.74 (m, 2H), 7.66 – 7.60 (m, 2H), 7.35 – 7.29 (m, 2H), 7.03 – 6.95 (m, 3H), 5.16 (s, 2H), 3.78 – 3.71 (m, 4H), 3.05 – 2.98 (m, 4H) ppm.

^{13}C NMR (151 MHz, $CDCl_3$) δ 158.4, 143.0, 134.7, 129.8, 128.3, 127.8, 121.6, 114.9, 69.0, 66.3, 46.1 ppm.

1-methoxy-3-(phenoxymethyl)benzene (9)

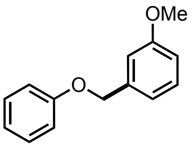

Compound (**9**) was synthesized following GP1 using alcohol **S1** (49.9 mg, 0.3 mmol), 3-Iodoanisole (23.4 mg, 0.10 mmol), $NiCl_2$•glyme (4.4 mg, 0.02 mmol), dtbbpy (6.7 mg, 0.025 mmol), [Mes-Acr-Me]$^+ClO_4^-$ (4.2 mg 0.01 mmol), and 2,4,6-collidine (40 mL, 0.30 mmol). After 48 h, the reaction produced the desired compound as a colorless oil (7.2 mg, **34 %** yield) after flash chromatography (petroleum spirit /EtOAc 95:5). Spectroscopic data agrees with the reported literature.[4]

^{1}H NMR (800 MHz, $CDCl_3$) δ 7.32 – 7.28 (m, 3H), 7.03 – 6.96 (m, 5H), 6.90 – 6.85 (m, 1H), 5.05 (s, 2H), 3.82 (s, 3H) ppm.

^{13}C NMR (201 MHz, $CDCl_3$) δ 160.0, 158.9, 138.8, 129.8, 129.6, 121.1, 119.8, 115.0, 113.6, 113.0, 69.9, 55.4 ppm.

2-(phenoxymethyl)benzonitrile (11)

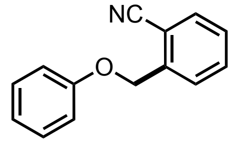

Compound (**11**) was synthesized following GP1 using alcohol **S1** (49.9 mg, 0.3 mmol), aryl bromide (18.2 mg, 0.10 mmol), $NiCl_2$•glyme (4.4 mg, 0.02 mmol), dtbbpy (6.7 mg, 0.025 mmol), [Mes-Acr-Me]$^+ClO_4^-$ (4.2 mg 0.01 mmol), and 2,4,6-collidine (40 mL, 0.30 mmol). After 48 h, the reaction produced the desired compound as a white solid (9.3 mg, **45 %** yield) after flash chromatography (petroleum spirit /EtOAc 95:5). Spectroscopic data agrees with the reported literature.[5]

^{1}H NMR (400 MHz, $CDCl_3$) δ 7.74 – 7.68 (m, 2H), 7.66 – 7.60 (m, 1H), 7.46 – 7.40 (m, 1H), 7.36 – 7.28 (m, 2H), 7.04 – 6.98 (m, 3H), 5.28 (s, 2H) ppm.

1-(phenoxymethyl)-3,5-bis(trifluoromethyl)benzene (12)

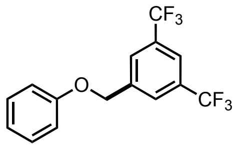

Compound (**12**) was synthesized following GP1 using alcohol **S1** (49.9 mg, 0.3 mmol), aryl bromide (29.3 mg, 0.10 mmol), $NiCl_2$•glyme (4.4 mg, 0.02 mmol), dtbbpy (6.7 mg, 0.025 mmol), [Mes-Acr-Me]$^+ClO_4^-$ (4.2 mg 0.01 mmol), and 2,4,6-collidine (40 mL, 0.30 mmol). After 48 h, the reaction produced the desired compound as a colorless oil (14.9 mg, **47 %** yield) after flash chromatography (petroleum spirit /EtOAc 95:5).

^{1}H NMR (400 MHz, $CDCl_3$) δ 7.92 (s, 2H), 7.85 (s, 1H), 7.38 – 7.30 (m, 2H), 7.06 – 6.96 (m, 3H), 5.17 (s, 2H) ppm.

^{13}C NMR (101 MHz, $CDCl_3$) δ 158.2, 139.9, 132.1 (q, $J_{C,F}$ = 33.4 Hz), 129.9, 127.4 (q, $J_{C,F}$ = 3.8 Hz), 127.4 (q, $J_{C,F}$ = 3.8 Hz), 123.4 (q, $J_{C,F}$ = 272.0 Hz), 122.0 (hept, $J_{C,F}$ = 3.8 Hz), 121.9, 114.9, 68.5 ppm.

HRMS (ESI) m/z: exact mass calculated for $C_{15}H_{11}F_6O^+$ [$(M+H)^+$]: 321,0709, Found: 321,0730.

5-(phenoxymethyl)-2-(trifluoromethyl)pyridine (13)

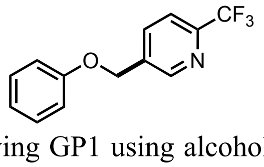

Compound (**13**) was synthesized following GP1 using alcohol **S1** (49.9 mg, 0.3 mmol), aryl bromide (22.6 mg, 0.10 mmol), $NiCl_2$•glyme (4.4 mg, 0.02 mmol), dtbbpy (6.7 mg, 0.025 mmol), [Mes-Acr-Me]$^+ClO_4^-$ (4.2 mg 0.01 mmol), and 2,4,6-collidine (40 mL, 0.30 mmol). After 48 h, the reaction produced the desired compound as a colorless oil (15.7 mg, **62 %** yield) after flash chromatography (petroleum spirit /EtOAc 95:5).

^{1}H NMR (400 MHz, $CDCl_3$) δ 8.80 (dd, J = 1.4, 0.7 Hz, 1H), 8.01 – 7.94 (m, 1H), 7.72 (d, J = 8.1 Hz, 1H), 7.36 – 7.28 (m, 2H), 7.05 – 6.94 (m, 3H), 5.17 (s, 2H) ppm.

^{13}C NMR (101 MHz, $CDCl_3$) δ 158.1, 149.0, 148.0 (q, $J_{C,F}$ = 34.9 Hz), 136.4, 136.2, 129.9, 121.9, 121.6 (q, $J_{C,F}$ = 273.9 Hz), 120.5 (q, $J_{C,F}$ = 2.7 Hz), 114.9, 66.8 ppm.

HRMS (ESI) m/z: exact mass calculated for $C_{13}H_{11}F_3NO^+$ [$(M+H)^+$]: 254,0787, Found: 254,0799.

2-fluoro-4-(phenoxymethyl)pyridine (14)

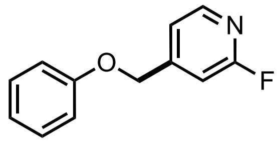

Compound (**14**) was synthesized following GP1 using alcohol **S1** (49.9 mg, 0.3 mmol), aryl bromide (17.6 mg, 0.10 mmol), $NiCl_2$•glyme (4.4 mg, 0.02 mmol), dtbbpy (6.7 mg, 0.025 mmol), $[Mes\text{-}Acr\text{-}Me]^+ClO_4^-$ (4.2 mg 0.01 mmol), and 2,4,6-collidine (40 mL, 0.30 mmol). After 48 h, the reaction produced the desired compound as a colorless oil (14.4 mg, **71 %** yield) after flash chromatography (petroleum spirit /EtOAc 95:5).

^{1}H NMR (800 MHz, $CDCl_3$) δ 8.21 (d, J = 5.2 Hz, 1H), 7.32 (t, J = 7.8 Hz, 2H), 7.23 (d, J = 5.2 Hz, 1H), 7.03 (s, 1H), 7.01 (t, J = 7.3 Hz, 1H), 6.95 (d, J = 8.1 Hz, 2H), 5.11 (s, 2H) ppm.

^{13}C NMR (201 MHz, $CDCl_3$) δ 164.3 (d, J = 238.7 Hz), 158.0, 152.5 (d, J = 7.7 Hz), 148.0 (d, J = 15.3 Hz), 129.8, 121.8, 119.1 (d, J = 4.1 Hz), 114.8, 107.3 (d, J = 38.7 Hz), 67.7 (d, J = 3.1 Hz) ppm.

HRMS (ESI) m/z: exact mass calculated for $C_{12}H_{11}FNO^+$ $[(M+H)^+]$: 204,0819, Found: 204,0831.

3-(phenoxymethyl)quinoline (15)

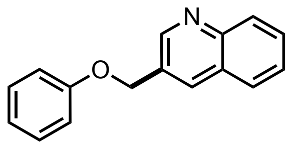

Compound (**15**) was synthesized following GP1 using alcohol **S1** (49.9 mg, 0.3 mmol), aryl bromide (20.8 mg, 0.10 mmol), $NiBr_2$•dtbbpy (9.7 mg, 0.02 mmol), $[Mes\text{-}Acr\text{-}Me]^+ClO_4^-$ (4.2 mg 0.01 mmol), and 2,4,6-collidine (40 mL, 0.30 mmol). After 48 h, the reaction produced the desired compound as a colorless oil (19.4 mg, **51 %** yield) after flash chromatography (petroleum spirit /EtOAc 95:5).

^{1}H NMR (600 MHz, $CDCl_3$) δ 8.99 (d, J = 2.2 Hz, 1H), 8.23 (dd, J = 2.3, 1.1 Hz, 1H), 8.15 – 8.11 (m, 1H), 7.87 – 7.83 (m, 1H), 7.75 – 7.71 (m, 1H), 7.59 – 7.55 (m, 1H), 7.35 – 7.30 (m, 2H), 7.05 – 6.98 (m, 3H), 5.27 (s, 2H) ppm.

^{13}C NMR (151 MHz, $CDCl_3$) δ 158.5, 150.3, 148.0, 134.7, 130.0, 129.8, 129.6, 129.5, 128.0, 127.9, 127.1, 121.6, 115.0, 67.9 ppm.

HRMS (ESI) m/z: exact mass calculated for $C_{16}H_{14}NO^+$ $[(M+H)^+]$: 236,1070, Found: 236,1081.

4-(phenoxymethyl)dibenzo[b,d]furan (16)

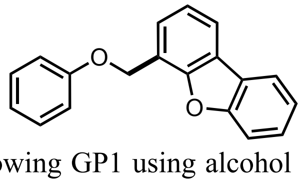

Compound (**16**) was synthesized following GP1 using alcohol **S1** (49.9 mg, 0.3 mmol), aryl bromide (24.7 mg, 0.10 mmol), $NiBr_2$•dtbbpy (9.7 mg, 0.02 mmol), $[Mes\text{-}Acr\text{-}Me]^+ClO_4^-$ (4.2 mg 0.01 mmol), and 2,4,6-collidine (40 mL, 0.30 mmol). After 48 h, the reaction produced the desired compound as a white solid (12.3 mg, **51 %** yield) after flash chromatography (petroleum spirit /EtOAc 95:5). Spectroscopic data agrees with the reported literature.[3]

^{1}H NMR (600 MHz, $CDCl_3$) δ 8.00 – 7.91 (m, 2H), 7.61 (d, J = 8.0 Hz, 2H), 7.50 – 7.46 (m, 1H), 7.39 – 7.35 (m, 2H), 7.35 – 7.30 (m, 2H), 7.10 – 7.06 (m, 2H), 7.01 – 6.97 (m, 1H), 5.49 (s, 2H) ppm.

^{13}C NMR (151 MHz, $CDCl_3$) δ 158.9, 156.3, 153.9, 129.7, 127.4, 126.7, 124.4, 124.3, 123.1, 123.0, 121.2, 121.2, 120.9, 120.5, 115.0, 111.9, 64.6 ppm.

(1R,2S,5R)-2-isopropyl-5-methylcyclohexyl 4-(phenoxymethyl)benzoate (17)

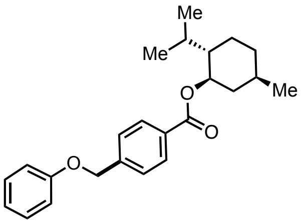

Compound (**17**) was synthesized following GP1 using alcohol **S1** (49.9 mg, 0.3 mmol), aryl bromide **S16** (33.9 mg, 0.10 mmol), $NiBr_2$•dtbbpy (9.7 mg, 0.02 mmol), $[Mes\text{-}Acr\text{-}Me]^+ClO_4^-$ (4.2 mg 0.01 mmol), and 2,4,6-collidine (40 mL, 0.30 mmol). After 48 h, the reaction produced the desired compound as a pale yellow oil (30.7 mg, **84 %** yield) after flash chromatography (petroleum spirit /EtOAc 95:5).

^{1}H NMR (600 MHz, $CDCl_3$) δ 8.09 – 8.03 (m, 2H), 7.50 (d, J = 8.2 Hz, 2H), 7.32 – 7.27 (m, 2H), 7.00 – 6.94 (m, 3H), 5.14 (s, 2H), 4.94 (td, J = 10.9, 4.4 Hz, 1H), 2.16 – 2.08 (m, 1H), 2.00 – 1.91 (m, 1H), 1.78 – 1.69 (m, 2H), 1.62 – 1.51 (m, 3H), 1.19 – 1.06 (m, 2H), 0.93 (d, J = 7.0 Hz, 3H), 0.92 (d, J = 7.0 Hz, 3H), 0.80 (d, J = 7.0 Hz, 3H) ppm.

^{13}C NMR (151 MHz, $CDCl_3$) δ 166.0, 158.6, 142.2, 130.5, 130.0, 129.7, 127.1, 121.3, 115.0, 75.0, 69.4, 47.4, 41.1, 34.5, 31.6, 26.7, 23.8, 22.2, 20.9, 16.7 ppm.

HRMS (ESI) m/z: exact mass calculated for $C_{24}H_{31}O_3^+$ [$(M+H)^+$]: 367,2268, Found: 367,2298.

((3a*R*,5*R*,5a*S*,8a*S*,8b*R*)-2,2,7,7-tetramethyltetrahydro-5*H*-bis([1,3]dioxolo)[4,5-*b*:4',5'-*d*]pyran-5-yl)methyl 4-(phenoxymethyl)benzoate (18)

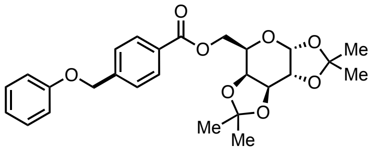

Compound (**18**) was synthesized following GP1 using alcohol **S1** (49.9 mg, 0.3 mmol), aryl bromide **S32** (44.2 mg, 0.10 mmol), $NiBr_2$•dtbbpy (9.7 mg, 0.02 mmol), $[Mes\text{-}Acr\text{-}Me]^+ClO_4^-$ (4.2 mg 0.01 mmol), and 2,4,6-collidine (40 mL, 0.30 mmol). After 48 h, the reaction produced the desired compound as a white solid (32.4 mg, **69 %** yield) after flash chromatography (pentane/EtOAc 5:1).

^{1}H NMR (600 MHz, $CDCl_3$) δ 8.11 – 8.02 (m, 2H), 7.50 (d, J = 8.1 Hz, 2H), 7.31 – 7.28 (m, 2H), 7.00 – 6.95 (m, 3H), 5.57 (d, J = 4.9 Hz, 1H), 5.13 (s, 2H), 4.65 (dd, J = 7.9, 2.4 Hz, 1H), 4.53 (dd, J = 11.5, 4.8 Hz, 1H), 4.43 (dd, J = 11.5, 7.6 Hz, 1H), 4.35 (dd, J = 5.0, 2.5 Hz, 1H), 4.33 (dd, J = 7.9, 1.9 Hz, 1H), 4.21 – 4.15 (m, 1H), 1.52 (s, 3H), 1.48 (s, 3H), 1.36 (s, 3H), 1.34 (s, 3H).ppm.

^{13}C NMR (151 MHz, $CDCl_3$) δ 166.3, 158.6, 142.6, 130.2, 129.7, 129.7, 127.1, 121.4, 115.0, 109.9, 109.0, 96.5, 71.3, 70.9, 70.7, 69.4, 66.3, 64.1, 26.2, 26.1, 25.1, 24.6 ppm.

HRMS (ESI) m/z: exact mass calculated for $C_{26}H_{31}O_8^+$ [$(M+H)^+$]: 471,2014, Found: 471,2040.

ethyl (4-(phenoxymethyl)benzoyl)-L-phenylalaninate (19)

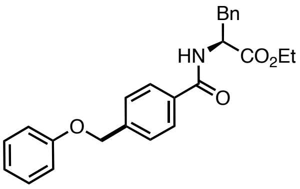

Compound (**19**) was synthesized following GP1 using alcohol **S1** (49.9 mg, 0.3 mmol), aryl bromide **S33** (37.5 mg, 0.10 mmol), $NiBr_2$•dtbbpy (9.7 mg, 0.02 mmol), $[Mes\text{-}Acr\text{-}Me]^+ClO_4^-$ (4.2 mg 0.01 mmol), and 2,4,6-collidine (40 mL, 0.30 mmol). After 48 h, the reaction produced the desired compound as a white solid (20.6 mg, **51 %** yield) after flash chromatography (pentane/EtOAc 7:1).

^{1}H NMR (600 MHz, $CDCl_3$) δ 7.74 (d, J = 8.1 Hz, 2H), 7.49 (d, J = 8.0 Hz, 2H), 7.31 – 7.27 (m, 4H), 7.25 – 7.20 (m, 1H), 7.16 – 7.13 (m, 2H), 7.00 – 6.94 (m, 3H), 6.59 (d, J = 7.5 Hz, 1H), 5.12 (s, 2H), 5.07 (dt, J = 7.3, 5.6 Hz, 1H), 4.22 (q, J = 7.2 Hz, 2H), 3.29 (dd, J = 13.9, 6.0 Hz, 1H), 3.24 (dd, J = 13.9, 5.3 Hz, 1H), 1.28 (t, J = 7.2 Hz, 3H).

^{13}C NMR (151 MHz, $CDCl_3$) δ 171.7, 166.6, 158.6, 141.2, 136.0, 133.6, 129.7, 129.6, 128.7, 127.5, 127.4, 127.3, 121.4, 115.0, 69.3, 61.8, 53.7, 38.1, 14.3 ppm.

HRMS (ESI) m/z: exact mass calculated for $C_{25}H_{26}NO_4^+$ $[(M+H)^+]$: 404,1857, Found: 404,1880.

methyl ((4-(phenoxymethyl)phenyl)sulfonyl)-D-prolinate (20)

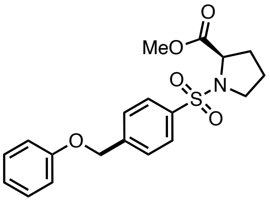

Compound (**20**) was synthesized following GP1 using alcohol **S1** (49.9 mg, 0.3 mmol), aryl bromide **S34** (34.7 mg, 0.10 mmol), $NiBr_2$•dtbbpy (9.7 mg, 0.02 mmol), $[Mes\text{-}Acr\text{-}Me]^+ClO_4^-$ (4.2 mg 0.01 mmol), and 2,4,6-collidine (40 mL, 0.30 mmol). After 48 h, the reaction produced the desired compound as a colorless oil (20.2 mg, **54 %** yield) after flash chromatography (pentane/EtOAc 6:1).

^{1}H NMR (600 MHz, $CDCl_3$) δ 7.90 (d, J = 8.1 Hz, 2H), 7.60 (d, J = 8.1 Hz, 2H), 7.34 – 7.28 (m, 2H), 7.02 – 6.95 (m, 3H), 5.14 (s, 2H), 4.34 (dd, J = 8.5, 3.8 Hz, 1H), 3.70 (s, 3H), 3.53 – 3.46 (m, 1H), 3.35 (dt, J = 9.0, 7.1 Hz, 1H), 2.06 – 1.95 (m, 3H), 1.84 – 1.75 (m, 1H) ppm.

^{13}C NMR (151 MHz, $CDCl_3$) δ 172.7, 158.4, 142.6, 137.9, 129.8, 127.9, 127.7, 121.6, 114.9, 69.0, 60.5, 52.6, 48.5, 31.1, 24.8 ppm.

HRMS (ESI) m/z: exact mass calculated for $C_{19}H_{22}NO_5S^+$ $[(M+H)^+]$: 376,1213, Found: 376,1233.

1-(4-isopropylphenyl)ethan-1-one (21)

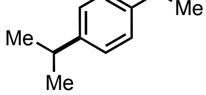

Compound (**21**) was synthesized following GP1 using tertiary alcohol **S18** (58.3 mg, 0.3 mmol), 4-bromoacetophenone (19.9 mg, 0.10 mmol), $NiBr_2$•dtbbpy (9.7 mg, 0.02 mmol), $[Mes\text{-}Acr\text{-}Me]^+ClO_4^-$ (4.2 mg 0.01 mmol), and 2,4,6-collidine (40 mL, 0.30 mmol). After 48 h, the reaction produced the desired compound as a colorless oil (13.3 mg, **82 %** yield) after flash chromatography (petroleum spirit /EtOAc 95:5). Spectroscopic data agrees with the reported literature.[6]

¹H NMR (600 MHz, $CDCl_3$) δ 7.90 (d, *J* = 8.3 Hz, 2H), 7.31 (d, *J* = 8.3 Hz, 2H), 2.97 (hept, *J* = 7.0 Hz, 1H), 2.58 (s, 3H), 1.27 (d, *J* = 6.9 Hz, 6H) ppm.

¹³C NMR (151 MHz, $CDCl_3$) δ 198.0, 154.8, 135.2, 128.7, 126.8, 34.4, 26.7, 23.8 ppm.

1-(4-(tert-butyl)phenyl)ethan-1-one (22)

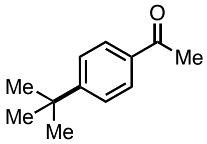

Compound (**22**) was synthesized following GP1 using tertiary alcohol **S2** (62.5 mg, 0.3 mmol), 4-bromoacetophenone (19.9 mg, 0.10 mmol), $NiBr_2$•dtbbpy (9.7 mg, 0.02 mmol), [Mes-Acr-Me]$^+ClO_4^-$ (4.2 mg 0.01 mmol), and 2,4,6-collidine (40 mL, 0.30 mmol). After 48 h, the reaction produced the desired compound as a colorless oil (6.2 mg, **35 %** yield) after flash chromatography (petroleum spirit /EtOAc 95:5). Spectroscopic data agrees with the reported literature.[7]

¹H NMR (600 MHz, $CDCl_3$) δ 7.90 (d, *J* = 8.5 Hz, 2H), 7.48 (d, *J* = 8.5 Hz, 2H), 2.58 (s, 3H), 1.34 (s, 9H) ppm.

¹³C NMR (151 MHz, $CDCl_3$) δ 198.0, 157.0, 134.8, 128.4, 125.6, 35.2, 31.2, 26.7 ppm.

1-(4-cyclopentylphenyl)ethan-1-one (23)

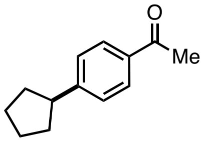

Compound (**23**) was synthesized following GP1 using tertiary alcohol **S17** (66.1 mg, 0.3 mmol), 4-bromoacetophenone (19.9 mg, 0.10 mmol), $NiBr_2$•dtbbpy (9.7 mg, 0.02 mmol), [Mes-Acr-Me]$^+ClO_4^-$ (4.2 mg 0.01 mmol), and 2,4,6-collidine (40 mL, 0.30 mmol). After 48 h, the reaction produced the desired compound as a colorless oil (7.6 mg, **42 %** yield) after flash chromatography (petroleum spirit /EtOAc 95:5). Spectroscopic data agrees with the reported literature.[8]

¹H NMR (800 MHz, $CDCl_3$) δ 7.88 (d, *J* = 7.9 Hz, 2H), 7.32 (d, *J* = 7.9 Hz, 2H), 3.09 – 3.01 (m, 1H), 2.58 (s, 3H), 2.13 – 2.06 (m, 2H), 1.86 – 1.80 (m, 2H), 1.74 – 1.68 (m, 2H), 1.64 – 1.59 (m, 2H) ppm.

¹³C NMR (201 MHz, $CDCl_3$) δ 198.0, 152.7, 135.1, 128.6, 127.4, 46.1, 34.6, 26.7, 25.7 ppm.

1-(4-cyclohexylphenyl)ethan-1-one (24)

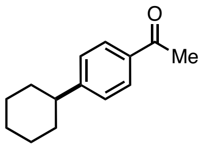

Compound (**24**) was synthesized following GP1 using tertiary alcohol **S5** (70.3 mg, 0.3 mmol) or secondary alcohol **S27** (66.1 mg, 0.3 mmol), 4-bromoacetophenone (19.9 mg, 0.10 mmol), $NiBr_2$•dtbbpy (9.7 mg, 0.02 mmol), [Mes-Acr-Me]$^+ClO_4^-$ (4.2 mg 0.01 mmol), and 2,4,6-collidine (40 mL, 0.30 mmol). After 48 h, the reaction produced the desired compound as a white solid (15.7 mg, **74 %** yield or 15.8 mg, **75 %** yield) after flash chromatography (petroleum spirit /EtOAc 95:5). Spectroscopic data agrees with the reported literature.[9]

¹H NMR (600 MHz, $CDCl_3$) δ 7.89 (dd, *J* = 8.4, 1.8 Hz, 2H), 7.30 (dd, *J* = 8.4, 1.9 Hz, 2H), 2.61 – 2.53 (m, 4H), 1.91 – 1.83 (m, 4H), 1.79 – 1.74 (m, 1H), 1.48 – 1.35 (m, 4H), 1.31 – 1.22 (m, 1H) ppm.

¹³C NMR (151 MHz, CDCl3) δ 198.0, 153.9, 135.2, 128.7, 127.2, 44.8, 34.3, 26.9, 26.7, 26.2 ppm.

1-(4-(tetrahydro-2H-pyran-4-yl)phenyl)ethan-1-one (25)

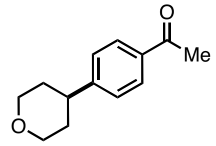

Compound (**25**) was synthesized following GP1 using tertiary alcohol **S6** (70.9 mg, 0.3 mmol), 4-bromoacetophenone (19.9 mg, 0.10 mmol), NiBr$_2$•dtbbpy (9.7 mg, 0.02 mmol), [Mes-Acr-Me]$^+$ClO$_4^-$ (4.2 mg 0.01 mmol), and 2,4,6-collidine (40 mL, 0.30 mmol). After 48 h, the reaction produced the desired compound as a pale yellow oil (10.0 mg, **48 %** yield) after flash chromatography (petroleum spirit /EtOAc 95:5). Spectroscopic data agrees with the reported literature.[10]

^{1}H NMR (400 MHz, CDCl$_3$) δ 7.92 (d, *J* = 8.4 Hz, 2H), 7.32 (d, *J* = 8.3 Hz, 2H), 4.11 – 4.05 (m, 2H), 3.54 (td, *J* = 11.5, 2.7 Hz, 2H), 2.88 – 2.78 (m, 1H), 2.59 (s, 3H), 1.90 – 1.74 (m, 4H) ppm.

^{13}C NMR (151 MHz, CDCl$_3$) δ 197.9, 151.5, 135.7, 128.9, 127.1, 68.3, 41.8, 33.7, 26.7 ppm.

1-(4-benzylphenyl)ethan-1-one (26)

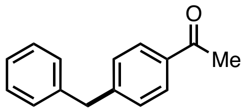

Compound (**26**) was synthesized following GP1 using tertiary alcohol **S7** (72.7 mg, 0.3 mmol), 4-bromoacetophenone (19.9 mg, 0.10 mmol), NiBr$_2$•dtbbpy (9.7 mg, 0.02 mmol), [Mes-Acr-Me]$^+$ClO$_4^-$ (4.2 mg 0.01 mmol), and 2,4,6-collidine (40 mL, 0.30 mmol). After 48 h, the reaction produced the desired compound as a white solid (17.2 mg, **82 %** yield) after flash chromatography (petroleum spirit /EtOAc 95:5). Spectroscopic data agrees with the reported literature.[11]

^{1}H NMR (600 MHz, CDCl$_3$) δ 7.89 (d, *J* = 8.1 Hz, 2H), 7.33 – 7.27 (m, 4H), 7.25 – 7.21 (m, 1H), 7.20 – 7.17 (m, 2H), 4.04 (s, 2H), 2.58 (s, 3H) ppm.

^{13}C NMR (151 MHz, CDCl$_3$) δ 197.9, 146.9, 140.2, 135.4, 129.2, 129.1, 128.8, 126.5, 42.0, 26.7 ppm.

1-(4-(4-chlorobenzyl)phenyl)ethan-1-one (27)

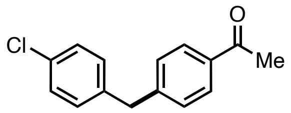

Compound (**27**) was synthesized following GP1 using tertiary alcohol **S8** (83.0 mg, 0.3 mmol), 4-bromoacetophenone (19.9 mg, 0.10 mmol), NiBr$_2$•dtbbpy (9.7 mg, 0.02 mmol), [Mes-Acr-Me]$^+$ClO$_4^-$ (4.2 mg 0.01 mmol), and 2,4,6-collidine (40 mL, 0.30 mmol). After 48 h, the reaction produced the desired compound as a pale yellow oil (21.3 mg, **85 %** yield) after flash chromatography (petroleum spirit /EtOAc 95:5). Spectroscopic data agrees with the reported literature.[12]

^{1}H NMR (600 MHz, CDCl$_3$) δ 7.89 (d, *J* = 8.3 Hz, 2H), 7.29 – 7.23 (m, 4H), 7.13 – 7.08 (m, 2H), 4.00 (s, 2H), 2.58 (s, 3H) ppm.

^{13}C NMR (151 MHz, CDCl$_3$) δ 197.8, 146.3, 138.6, 135.6, 132.4, 130.4, 129.2, 128.9, 128.9, 41.3, 26.7 ppm.

1-(4-(4-fluorobenzyl)phenyl)ethan-1-one (28)

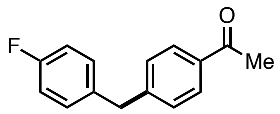

Compound (**28**) was synthesized following GP1 using tertiary alcohol **S9** (78.1 mg, 0.3 mmol), 4-bromoacetophenone (19.9 mg, 0.10 mmol), NiBr$_2$•dtbbpy (9.7 mg, 0.02 mmol), [Mes-Acr-Me]$^+$ClO$_4^-$

(4.2 mg 0.01 mmol), and 2,4,6-collidine (40 mL, 0.30 mmol). After 48 h, the reaction produced the desired compound as a pale yellow oil (23.1 mg, **94 %** yield) after flash chromatography (petroleum spirit /EtOAc 95:5). Spectroscopic data agrees with the reported literature.[12]

^{1}H NMR (600 MHz, $CDCl_3$) δ 7.89 (d, J = 8.3 Hz, 2H), 7.26 (d, J = 8.3 Hz, 2H), 7.17 – 7.10 (m, 2H), 7.02 – 6.95 (m, 2H), 4.00 (s, 2H), 2.58 (s, 3H) ppm.

^{13}C NMR (151 MHz, $CDCl_3$) δ 197.9, 161.7 (d, $J_{C,F}$ = 244.6 Hz), 146.7, 135.8 (d, $J_{C,F}$ = 3.4 Hz), 135.5, 130.5 (d, $J_{C,F}$ = 7.9 Hz), 129.1, 128.8, 115.6 (d, $J_{C,F}$ = 21.1 Hz), 41.2, 26.7 ppm.

1-(4-(4-methylbenzyl)phenyl)ethan-1-one (29)

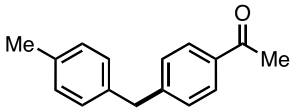

Compound (**29**) was synthesized following GP1 using tertiary alcohol **S10** (76.9 mg, 0.3 mmol), 4-bromoacetophenone (19.9 mg, 0.10 mmol), $NiBr_2$•dtbbpy (9.7 mg, 0.02 mmol), [Mes-Acr-Me]$^+ClO_4^-$ (4.2 mg 0.01 mmol), and 2,4,6-collidine (40 mL, 0.30 mmol). After 48 h, the reaction produced the desired compound as a white solid (21.7 mg, **87 %** yield) after flash chromatography (petroleum spirit /EtOAc 95:5). Spectroscopic data agrees with the reported literature.[12]

^{1}H NMR (600 MHz, $CDCl_3$) δ 7.88 (d, J = 8.4 Hz, 2H), 7.28 (d, J = 8.4 Hz, 2H), 7.12 (d, J = 8.0 Hz, 2H), 7.07 (d, J = 8.1 Hz, 2H), 4.00 (s, 2H), 2.58 (s, 3H), 2.33 (s, 3H) ppm.

^{13}C NMR (151 MHz, $CDCl_3$) δ 197.9, 147.3, 137.1, 136.1, 135.3, 129.5, 129.2, 128.9, 128.8, 41.6, 26.7, 21.1 ppm.

1-(4-(naphthalen-2-ylmethyl)phenyl)ethan-1-one (30)

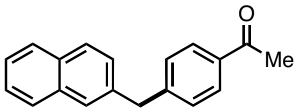

Compound (**30**) was synthesized following GP1 using tertiary alcohol **S11** (87.7 mg, 0.3 mmol), 4-bromoacetophenone (19.9 mg, 0.10 mmol), $NiBr_2$•dtbbpy (9.7 mg, 0.02 mmol), [Mes-Acr-Me]$^+ClO_4^-$ (4.2 mg 0.01 mmol), and 2,4,6-collidine (40 mL, 0.30 mmol). After 48 h, the reaction produced the desired compound as a colorless oil (28.6 mg, **76 %** yield) after flash chromatography (petroleum spirit /EtOAc 95:5). Spectroscopic data agrees with the reported literature.[13]

^{1}H NMR (600 MHz, $CDCl_3$) δ 7.91 – 7.88 (m, 2H), 7.82 – 7.79 (m, 1H), 7.77 (dd, J = 7.9, 1.2 Hz, 2H), 7.63 (dd, J = 1.8, 0.9 Hz, 1H), 7.48 – 7.43 (m, 2H), 7.34 – 7.31 (m, 2H), 7.29 (dd, J = 8.4, 1.8 Hz, 1H), 4.20 (s, 2H), 2.58 (s, 3H) ppm.

^{13}C NMR (151 MHz, $CDCl_3$) δ 197.9, 146.8, 137.7, 135.5, 133.7, 132.3, 129.4, 128.8, 128.5, 127.8, 127.7, 127.5, 127.4, 126.3, 125.7, 42.2, 26.7 ppm.

1-(4-(furan-2-ylmethyl)phenyl)ethan-1-one (31)

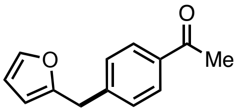

Compound (**31**) was synthesized following GP1 using tertiary alcohol **S20** (69.7 mg, 0.3 mmol), 4-bromoacetophenone (19.9 mg, 0.10 mmol), $NiBr_2$•dtbbpy (9.7 mg, 0.02 mmol), [Mes-Acr-Me]$^+ClO_4^-$ (4.2 mg 0.01 mmol), and 2,4,6-collidine (40 mL, 0.30 mmol). After 48 h, the reaction produced the desired compound as a colorless oil (18.5 mg, **92 %** yield) after flash chromatography (petroleum spirit /EtOAc 95:5).

^{1}H NMR (600 MHz, $CDCl_3$) δ 7.92 – 7.88 (m, 2H), 7.36 – 7.29 (m, 3H), 6.31 (dd, J = 3.2, 1.9 Hz, 1H), 6.07 – 6.02 (m, 1H), 4.03 (s, 2H), 2.58 (s, 3H) ppm.

^{13}**C NMR** (151 MHz, $CDCl_3$) δ 197.9, 153.4, 143.9, 141.9, 135.7, 129.0, 128.8, 110.5, 106.8, 34.6, 26.7 ppm.

HRMS (ESI) m/z: exact mass calculated for $C_{13}H_{13}O_2^+$ [(M+H)$^+$]: 201,0910, Found: 201,0922.

1-(4-((4-(trifluoromethyl)phenoxy)methyl)phenyl)ethan-1-one (32)

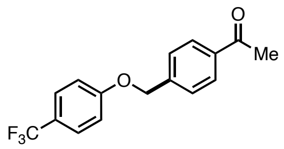

Compound (**32**) was synthesized following GP1 using tertiary alcohol **S12** (97.9 mg, 0.3 mmol), 4-bromoacetophenone (19.9 mg, 0.10 mmol), $NiBr_2$•dtbbpy (9.7 mg, 0.02 mmol), [Mes-Acr-Me]$^+ClO_4^-$ (4.2 mg 0.01 mmol), and 2,4,6-collidine (40 mL, 0.30 mmol). After 48 h, the reaction produced the desired compound as a white solid (17.6 mg, **60 %** yield) after flash chromatography (petroleum spirit /EtOAc 95:5).

^{1}**H NMR** (400 MHz, $CDCl_3$) δ 8.03 – 7.95 (m, 2H), 7.60 – 7.49 (m, 4H), 7.06 – 6.99 (m, 2H), 5.18 (s, 2H), 2.62 (s, 3H) ppm.

^{13}**C NMR** (151 MHz, CDCl3) δ 197.7, 160.9, 141.7, 137.1, 128.9, 127.3, 127.2 (q, $J_{C,F}$ = 3.8 Hz), 124.5 (q, $J_{C,F}$ = 271.2 Hz), 123.6 (q, $J_{C,F}$ = 32.9 Hz), 115.0, 114.8, 69.6, 26.8.

HRMS (ESI) m/z: exact mass calculated for $C_{16}H_{14}F_3O_2^+$ [(M+H)$^+$]: 295,0940, Found: 295,0963.

1-(4-((4-chlorophenoxy)methyl)phenyl)ethan-1-one (33)

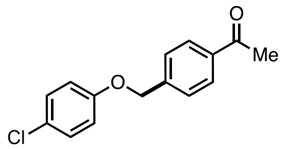

Compound (**33**) was synthesized following GP1 using tertiary alcohol **S14** (87.8 mg, 0.3 mmol) or secondary alcohol **S23** (83.6 mg, 0.3 mmol), 4-bromoacetophenone (19.9 mg, 0.10 mmol), $NiBr_2$•dtbbpy (9.7 mg, 0.02 mmol), [Mes-Acr-Me]$^+ClO_4^-$ (4.2 mg 0.01 mmol), and 2,4,6-collidine (40 mL, 0.30 mmol). After 48 h, the reaction produced the desired compound as a white solid (10.4 mg, **40 %** yield) or (22.0 mg, **81 %** yield) after flash chromatography (petroleum spirit /EtOAc 95:5).

^{1}**H NMR** (600 MHz, $CDCl_3$) δ 8.00 – 7.96 (m, 2H), 7.53 – 7.48 (m, 2H), 7.26 – 7.22 (m, 2H), 6.91 – 6.86 (m, 2H), 5.10 (s, 2H), 2.61 (s, 3H) ppm.

^{13}**C NMR** (151 MHz, $CDCl_3$) δ 197.9, 157.1, 142.1, 136.9, 129.6, 128.8, 127.3, 126.3, 116.3, 69.7, 26.8 ppm.

HRMS (ESI) m/z: exact mass calculated for $C_{15}H_{14}ClO_2^+$ [(M+H)$^+$]: 261,0677, Found: 261,0697.

1-(4-((p-tolyloxy)methyl)phenyl)ethan-1-one (34)

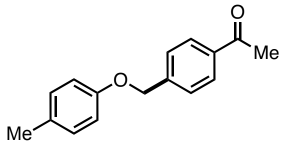

Compound (**34**) was synthesized following GP1 using secondary alcohol **S24** (77.5 mg, 0.3 mmol), 4-bromoacetophenone (19.9 mg, 0.10 mmol), $NiBr_2$•dtbbpy (9.7 mg, 0.02 mmol), [Mes-Acr-Me]$^+ClO_4^-$ (4.2 mg 0.01 mmol), and 2,4,6-collidine (40 mL, 0.30 mmol). After 48 h, the reaction produced the desired compound as a colorless oil (14.9 mg, **62 %** yield) after flash chromatography (petroleum spirit /EtOAc 95:5).

^{1}H NMR (800 MHz, $CDCl_3$) δ 7.97 (d, *J* = 7.8 Hz, 2H), 7.52 (d, *J* = 7.9 Hz, 2H), 7.09 (d, *J* = 8.0 Hz, 2H), 6.86 (d, *J* = 7.8 Hz, 2H), 5.11 (s, 2H), 2.61 (s, 3H), 2.29 (s, 3H) ppm.

^{13}C NMR (201 MHz, $CDCl_3$) δ 197.9, 156.5, 142.9, 136.7, 130.7, 130.1, 128.8, 127.3, 114.8, 69.5, 26.8, 20.6 ppm.

HRMS (ESI) m/z: exact mass calculated for $C_{16}H_{17}O_2^+$ [$(M+H)^+$]: 241,1223, Found: 241,1240.

1-(4-((4-fluorophenoxy)methyl)phenyl)ethan-1-one (35)

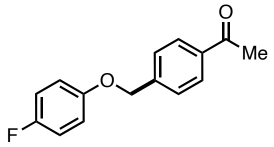

Compound (**35**) was synthesized following GP1 using tertiary alcohol **S3** (55.2 mg, 0.3 mmol) or secondary alcohol **S25** (78.7 mg, 0.3 mmol), 4-bromoacetophenone (19.9 mg, 0.10 mmol), $NiCl_2$•glyme (4.4 mg, 0.02 mmol), dtbbpy (6.7 mg, 0.025 mmol), $[Mes\text{-}Acr\text{-}Me]^+ClO_4^-$ (4.2 mg 0.01 mmol), and 2,4,6-collidine (40 mL, 0.30 mmol). After 48 h, the reaction produced the desired compound as a white solid (13.9 mg, **57 %** yield) or (19.5 mg, **80 %** yield) after flash chromatography (petroleum spirit /EtOAc 95:5).

^{1}H NMR (600 MHz, $CDCl_3$) δ 8.00 – 7.96 (m, 2H), 7.54 – 7.49 (m, 2H), 7.01 – 6.95 (m, 2H), 6.91 – 6.88 (m, 2H), 5.09 (s, 2H), 2.61 (s, 3H) ppm.

^{13}C NMR (151 MHz, $CDCl_3$) δ 197.9, 157.6 (d, J_{CF} = 239.4 Hz), 154.7, 142.4, 136.8, 128.8, 127.3, 116.1 (d, J_{CF} = 15.9 Hz), 116.0 (d, J_{CF} = 7.6 Hz), 70.0, 26.8 ppm.

HRMS (ESI) m/z: exact mass calculated for $C_{15}H_{14}FO_2^+$ [$(M+H)^+$]: 245,0973, Found: 245,0991.

1-(4-((3-chloro-4-methylphenoxy)methyl)phenyl)ethan-1-one (36)

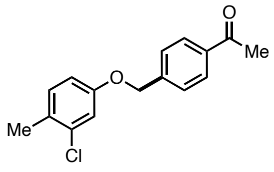

Compound (**36**) was synthesized following GP1 using tertiary alcohol **S4** (64.4 mg, 0.3 mmol), 4-bromoacetophenone (19.9 mg, 0.10 mmol), $NiBr_2$•dtbbpy (9.7 mg, 0.02 mmol), $[Mes\text{-}Acr\text{-}Me]^+ClO_4^-$ (4.2 mg 0.01 mmol), and 2,4,6-collidine (40 mL, 0.30 mmol). After 48 h, the reaction produced the desired compound as a white solid (14.8 mg, **54 %** yield) after flash chromatography (petroleum spirit /EtOAc 95:5).

^{1}H NMR (600 MHz, $CDCl_3$) δ 8.00 – 7.96 (m, 2H), 7.53 – 7.49 (m, 2H), 7.12 (d, *J* = 8.4 Hz, 1H), 6.98 (d, *J* = 2.6 Hz, 1H), 6.77 (dd, *J* = 8.4, 2.6 Hz, 1H), 5.09 (s, 2H), 2.61 (s, 3H), 2.30 (s, 3H) ppm.

^{13}C NMR (151 MHz, $CDCl_3$) δ 197.8, 157.2, 142.2, 136.9, 134.8, 131.5, 128.8, 128.7, 127.3, 115.7, 113.6, 69.7, 26.8, 19.2 ppm.

HRMS (ESI) m/z: exact mass calculated for $C_{16}H_{16}ClO_2^+$ [$(M+H)^+$]: 275,0834, Found: 275,0853

1-(4-butylphenyl)ethan-1-one (37)

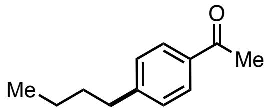

Compound (**37**) was synthesized following GP1 using secondary alcohol **S19** (58.3 mg, 0.3 mmol), 4-bromoacetophenone (19.9 mg, 0.10 mmol), $NiBr_2$•dtbbpy (9.7 mg, 0.02 mmol), $[Mes\text{-}Acr\text{-}Me]^+ClO_4^-$ (4.2 mg 0.01 mmol), and 2,4,6-collidine (40 mL, 0.30 mmol). After 48 h, the reaction produced the

desired compound as a colorless oil (6.4 mg, 36 **%** yield) after flash chromatography (petroleum spirit /EtOAc 95:5). Spectroscopic data agrees with the reported literature.[14]

^{1}H NMR (600 MHz, $CDCl_3$) δ 7.88 (d, J = 8.2 Hz, 2H), 7.27 (d, J = 7.7 Hz, 2H), 2.67 (t, J = 7.7 Hz, 2H), 2.58 (s, 3H), 1.64 – 1.59 (m, 2H), 1.39 – 1.33 (m, 2H), 0.93 (t, J = 7.3 Hz, 3H) ppm.

^{13}C NMR (151 MHz, $CDCl_3$) δ 198.1, 149.0, 135.0, 128.8, 128.6, 35.8, 33.4, 26.7, 22.5, 14.0 ppm.

1-(4-(((4-fluorophenyl)thio)methyl)phenyl)ethan-1-one (38)

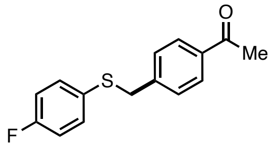

Compound (**38**) was synthesized following GP1 using secondary alcohol **S22** (83.5 mg, 0.3 mmol), 4-bromoacetophenone (19.9 mg, 0.10 mmol), $NiBr_2$•dtbbpy (9.7 mg, 0.02 mmol), [Mes-Acr-Me]$^+ClO_4^-$ (4.2 mg 0.01 mmol), and 2,4,6-collidine (40 mL, 0.30 mmol). After 48 h, the reaction produced the desired compound as a white solid (14.9 mg, **57 %** yield) after flash chromatography (petroleum spirit /EtOAc 95:5).

^{1}H NMR (600 MHz, $CDCl_3$) δ 7.84 (d, J = 8.3 Hz, 2H), 7.28 – 7.20 (m, 4H), 6.95 – 6.89 (m, 2H), 4.02 (s, 2H), 2.56 (s, 3H) ppm.

^{13}C NMR (151 MHz, $CDCl_3$) δ 197.80, 162.46 (d, J_{CF} = 247.7 Hz), 143.39, 136.15, 134.12 (d, J_{CF} = 7.8 Hz), 129.98 (d, J_{CF} = 3.7 Hz), 129.14, 128.69, 116.20 (d, J_{CF} = 21.9 Hz), 40.46, 26.75 ppm.

HRMS (ESI) m/z: exact mass calculated for $C_{15}H_{14}FOS^+$ [$(M+H)^+$]: 261,0744, Found: 261,0762.

1-(4-((phenylthio)methyl)phenyl)ethan-1-one (39)

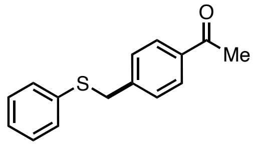

Compound (**39**) was synthesized following GP1 using secondary alcohol **S21** (78.1 mg, 0.3 mmol), 4-bromoacetophenone (19.9 mg, 0.10 mmol), $NiBr_2$•dtbbpy (9.7 mg, 0.02 mmol), [Mes-Acr-Me]$^+ClO_4^-$ (4.2 mg 0.01 mmol), and 2,4,6-collidine (40 mL, 0.30 mmol). After 48 h, the reaction produced the desired compound as a colorless oil (13.4 mg, **55 %** yield) after flash chromatography (petroleum spirit /EtOAc 95:5). Spectroscopic data agrees with the reported literature.[15]

^{1}H NMR (600 MHz, $CDCl_3$) δ 7.86 (d, J = 8.3 Hz, 2H), 7.35 (d, J = 8.3 Hz, 2H), 7.30 – 7.28 (m, 2H), 7.26 – 7.23 (m, 2H), 7.22 – 7.18 (m, 1H), 4.13 (s, 2H), 2.58 (s, 3H) ppm.

^{13}C NMR (151 MHz, $CDCl_3$) δ 197.8, 143.4, 136.2, 135.5, 130.6, 129.1, 129.1, 128.7, 127.0, 39.2, 26.8 ppm.

4-acetylbenzyl benzoate (41)

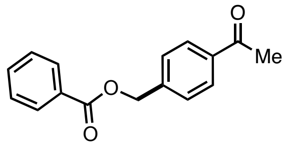

Compound (**41**) was synthesized following GP1 using secondary alcohol **S28** (81.7 mg, 0.3 mmol), 4-bromoacetophenone (19.9 mg, 0.10 mmol), $NiBr_2$•dtbbpy (9.7 mg, 0.02 mmol), [Mes-Acr-Me]$^+ClO_4^-$ (4.2 mg 0.01 mmol), and 2,4,6-collidine (40 mL, 0.30 mmol). After 48 h, the reaction produced the desired compound as a colorless oil (10.4 mg, **41 %** yield) after flash chromatography (petroleum spirit /EtOAc 95:5).

^{1}**H NMR** (600 MHz, $CDCl_3$) δ 8.11 – 8.07 (m, 2H), 8.00 – 7.96 (m, 2H), 7.60 – 7.56 (m, 1H), 7.56 – 7.52 (m, 2H), 7.48 – 7.44 (m, 2H), 5.42 (s, 2H), 2.61 (s, 3H) ppm.

^{13}**C NMR** (151 MHz, $CDCl_3$) δ 197.8, 166.4, 141.5, 137.0, 133.4, 130.7, 129.9, 128.8, 128.6, 128.0, 66.0, 26.8 ppm.

HRMS (ESI) m/z: exact mass calculated for $C_{16}H_{15}O_3^+$ [(M+H)$^+$]: 255,1016, Found: 255,1033.

4-acetylbenzyl thiophene-2-carboxylate (42)

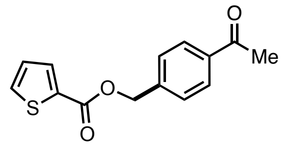

Compound (**42**) was synthesized following GP1 using secondary alcohol **S29** (83.5 mg, 0.3 mmol), 4-bromoacetophenone (19.9 mg, 0.10 mmol), $NiBr_2$•dtbbpy (9.7 mg, 0.02 mmol), [Mes-Acr-Me]$^+$$ClO_4^-$ (4.2 mg 0.01 mmol), and 2,4,6-collidine (40 mL, 0.30 mmol). After 48 h, the reaction produced the desired compound as a colorless oil (10.8 mg, **42 %** yield) after flash chromatography (petroleum spirit /EtOAc 95:5).

^{1}**H NMR** (600 MHz, $CDCl_3$) δ 8.00 – 7.96 (m, 2H), 7.85 (dd, *J* = 3.8, 1.3 Hz, 1H), 7.59 (dd, *J* = 5.0, 1.3 Hz, 1H), 7.54 – 7.50 (m, 2H), 7.12 (dd, *J* = 4.9, 3.7 Hz, 1H), 5.39 (s, 2H), 2.61 (s, 3H) ppm.

^{13}**C NMR** (151 MHz, $CDCl_3$) δ 197.8, 162.0, 141.2, 137.0, 134.0, 133.3, 133.0, 128.8, 128.0, 128.0, 66.0, 26.8 ppm.

HRMS (ESI) m/z: exact mass calculated for $C_{14}H_{13}O_3S^+$ [(M+H)$^+$]: 261,0580, Found: 261,0599.

4-acetylbenzyl 2-(2-fluoro-[1,1'-biphenyl]-4-yl)propanoate (43)

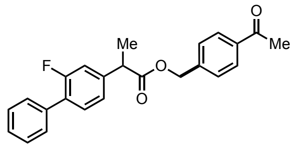

Compound (**43**) was synthesized was synthesized following GP1 using secondary alcohol **S30** (118.3 mg, 0.3 mmol), 4-bromoacetophenone (19.9 mg, 0.10 mmol), $NiBr_2$•dtbbpy (9.7 mg, 0.02 mmol), [Mes-Acr-Me]$^+$$ClO_4^-$ (4.2 mg 0.01 mmol), and 2,4,6-collidine (40 mL, 0.30 mmol). After 48 h, the reaction produced the desired compound as a colorless oil (14.9 mg, **39 %** yield) after flash chromatography (petroleum spirit /EtOAc 95:5).

^{1}**H NMR** (600 MHz, $CDCl_3$) δ 7.94 – 7.89 (m, 2H), 7.55 – 7.52 (m, 2H), 7.46 – 7.43 (m, 2H), 7.41 – 7.34 (m, 4H), 7.15 (dd, *J* = 7.9, 1.8 Hz, 1H), 7.11 (dd, *J* = 11.5, 1.8 Hz, 1H), 5.23 – 5.15 (m, 2H), 3.83 (q, *J* = 7.2 Hz, 1H), 2.58 (s, 3H), 1.57 (d, *J* = 7.2 Hz, 3H).

^{13}**C NMR** (151 MHz, $CDCl_3$) δ 197.7, 173.7, 159.8 (d, *J* = 248.2 Hz), 141.6 (d, *J* = 7.3 Hz), 141.2, 137.0, 135.6 (d, *J* = 7.4 Hz), 131.0 (d, *J* = 4.0 Hz), 129.1 (d, *J* = 2.9 Hz), 128.7, 128.6, 128.1 (d, *J* = 13.6 Hz), 127.9, 127.8, 123.7 (d, *J* = 3.5 Hz), 115.4 (d, *J* = 23.7 Hz), 66.0, 45.2, 26.8, 18.4 ppm.

HRMS (ESI) m/z: exact mass calculated for $C_{24}H_{22}FO_3^+$ [(M+H)$^+$]: 377,1548, Found: 377,1569.

4-acetylbenzyl 2-(4-isobutylphenyl)propanoate (44)

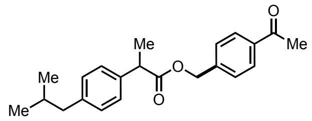

Compound (**44**) was synthesized was synthesized following GP1 using secondary alcohol **S31** (106.9 mg, 0.3 mmol), 4-bromoacetophenone (19.9 mg, 0.10 mmol), $NiBr_2$•dtbbpy (9.7 mg, 0.02 mmol), [Mes-Acr-Me]$^+$$ClO_4^-$ (4.2 mg 0.01 mmol), and 2,4,6-collidine (40 mL, 0.30 mmol). After 48 h, the reaction

produced the desired compound as a colorless oil (14.6 mg, **43 %** yield) after flash chromatography (petroleum spirit /EtOAc 95:5).

^{1}H NMR (600 MHz, $CDCl_3$) δ 7.90 – 7.85 (m, 2H), 7.29 – 7.26 (m, 2H), 7.22 – 7.18 (m, 2H), 7.12 – 7.08 (m, 2H), 5.20 – 5.11 (m, 2H), 3.77 (q, *J* = 7.2 Hz, 1H), 2.58 (s, 3H), 2.46 (d, *J* = 7.2 Hz, 2H), 1.86 (hept, *J* = 6.7 Hz, 1H), 1.52 (d, *J* = 7.1 Hz, 3H), 0.91 (d, *J* = 6.6 Hz, 6H) ppm.

^{13}C NMR (151 MHz, $CDCl_3$) δ 197.8, 174.5, 141.6, 140.9, 137.5, 136.8, 129.5, 128.6, 127.5, 127.4, 65.6, 45.3, 45.2, 30.4, 26.8, 22.5, 18.4 ppm.

HRMS (ESI) m/z: exact mass calculated for $C_{22}H_{27}O_3^+$ [$(M+H)^+$]: 339,1955, Found: 339,1976.

1-(4-((3aR,5R,5aS,8aS,8bR)-2,2,7,7-tetramethyltetrahydro-5H-bis([1,3]dioxolo)[4,5-b:4',5'-d]pyran-5-yl)phenyl)ethan-1-one (45)

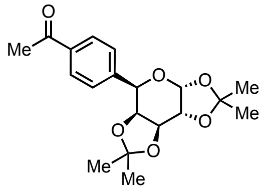

Compound (**45**) was synthesized was synthesized following GP1 using secondary alcohol **S15** low diastereomer (110.0 mg, 0.3 mmol), 4-bromoacetophenone (19.9 mg, 0.10 mmol), $NiBr_2$•dtbbpy (9.7 mg, 0.02 mmol), $[Mes\text{-}Acr\text{-}Me]^+ClO_4^-$ (4.2 mg 0.01 mmol), and 2,4,6-collidine (40 mL, 0.30 mmol). After 48 h, the reaction produced the desired compound as a white solid (11.90 mg, **34% yield**) after flash chromatography (pentane/EtOAc 5:1).

^{1}H NMR (600 MHz, $CDCl_3$) δ 7.95 (d, *J* = 8.0 Hz, 2H), 7.49 (d, *J* = 7.9 Hz, 2H), 5.72 (d, *J* = 5.0 Hz, 1H), 4.94 (s, 1H), 4.73 (dd, *J* = 7.8, 2.4 Hz, 1H), 4.48 – 4.37 (m, 2H), 2.59 (s, 3H), 1.57 (s, 3H), 1.44 (s, 3H), 1.38 (s, 3H), 1.29 (s, 3H) ppm.

^{13}C NMR (151 MHz, $CDCl_3$) δ 198.0, 143.3, 136.4, 128.3, 127.1, 109.6, 109.0, 97.0, 73.8, 71.2, 70.7, 69.4, 26.8, 26.3, 26.0, 25.1, 24.4 ppm.

HRMS (ESI) m/z: exact mass calculated for $C_{19}H_{25}O_6^+$ [$(M+H)^+$]: 349,1646, Found: 349,1665. Reaction was conducted with low diastereomer.

Synthesis & characterization of starting materials

Three general procedures have been used to prepare the main portion of the starting materials. The specific syntheses of starting materials beyond these three methods are described in the following section together with the characterization data for that compound.

General procedure 2 for the syntheses of alcohols (GP2)

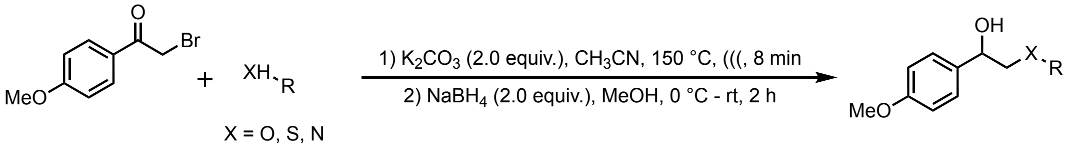

To an oven-dried 20 mL microwave vial equipped with a stirring bar, K_2CO_3 (10.0 mmol, 2.0 equiv.) was added, followed by α-bromo-4-methoxyacetophenone (5.0 mmol, 1.0 equiv.), phenol (thiol, amine or carboxylic acid) (5.0 mmol, 1.0 equiv.), and CH_3CN (12 mL). The vial was then capped and reacted in the BIOTAGE Initiator Microwave Synthesizer at 150 °C for 8 minutes. After the reaction was completed, the K_2CO_3 was filtered out and washed with some CH_3CN. The solvent is evaporated to obtain the ketone, which was then dissolved in MeOH (25 mL) or DCM. Next, $NaBH_4$ (10.0 mmol, 2.0 equiv.) was added to the reaction vessel with three portions at 0 °C (after the addition was complete, the

ice bath was not removed, allowing the reaction to gradually reach room temperature), and the mixture was stirred for 2 hours (completion of the reaction was monitored by TLC). Afterwards, the reaction was carefully quenched with saturated NH_4Cl solution, and the aqueous layer was extracted with 3 x EtOAc. The combined organic layers were washed with brine, dried over Na_2SO_4, and concentrated. Lastly, the crude product was purified using Silica gel flash column chromatography, eluting with 5% EtOAc/ petroleum spirit. The purified product was then confirmed by NMR and/or HR-MS.

General procedure 3 for the syntheses of alcohols (GP3)

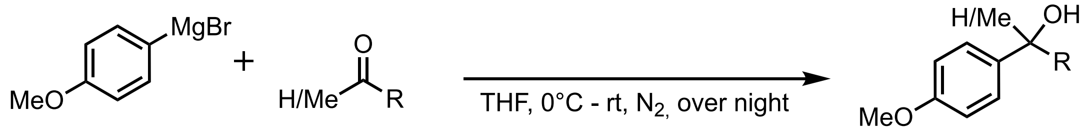

To an oven dried 50 mL round-bottom flask equipped with stirring bar, magnesium turnings (9.0 mmol, 1.5 equiv.), iodine (a little bit), and dry THF (10 mL) was added under N_2 gas. The mixture was stirred for 5 min, then 4-bromoanisole (6.0 mmol, 1.2 equiv.) which pre-dissolved in 3 mL dry THF was added in a dropwise manner. Subsequently, the mixture was warmed to 40°C and further stirring for 4 hours to complete the Grignard formation. Next, the reaction was cooled to 0°C and a diluted THF (10 mL) solution of the aldehyde or ketone (5.0 mmol, 1.0 equiv.) was dropwise added in 30min. The system was stirred overnight and allowed to gradually warm to rt. After confirmation by TLC and GC-MS the reaction would be quenched with cold saturated NH_4Cl, extracted with 3 x EtOAc washes, and dried over Na_2SO_4. The concentrated crude was then purified by Flash Silica column chromatography with eluent of 3% EtOAc/Hexane. The purified product was then confirmed by NMR and/or HR-MS.

General procedure 4 for the syntheses of alcohols (GP4)

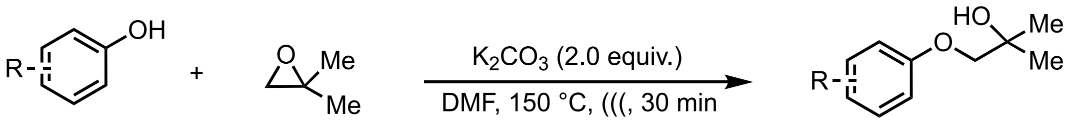

To an oven-dried 20 mL microwave vial equipped with a stirring bar, K_2CO_3 (20.0 mmol, 2.0 equiv.) was added, followed by phenol (10.0 mmol, 1.0 equiv.), 1,1-dimethyloxirane (30.0 mmol, 3.0 equiv.), and dry DMF (10 mL). The vial was then capped and reacted in the BIOTAGE Initiator Microwave Synthesizer at 150 °C for 30 minutes. After the reaction was completed, H_2O (20 mL) was added to the reaction crude, and the aqueous layer was extracted with 3 x EtOAc. The combined organic layers were washed with brine until the DMF was fully washed away, then the organic layer dried over Na_2SO_4, and concentrated to give title compounds almost pure. If necessary, the crude product was purified using Silica gel flash column chromatography, eluting with 5% EtOAc/ petroleum spirit. The purified product was then confirmed by NMR and/or HR-MS.

Preparation and characterization of alcohols

2-methyl-1-phenoxypropan-2-ol (S1):

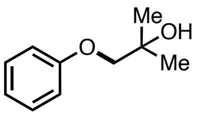

Synthesized according to ***GP4*** using 1,1-dimethyloxirane (30.0 mmol, 3.0 equiv.) and phenol (10.0 mmol, 1.0 equiv.). Purification via flash chromatography using silica gel (petroleum spirit / EtOAc 95:5) afforded **S1** as a white powder (1545.8 mg, **93 %** yield). Spectroscopic data agrees with the literature.[16]

^{1}H NMR (400 MHz, $CDCl_3$) δ 7.33 – 7.26 (m, 2H), 7.00 – 6.90 (m, 3H), 3.80 (s, 2H), 2.28 (s, 1H), 1.35 (s, 6H) ppm.

2-(4-methoxyphenyl)-3,3-dimethylbutan-2-ol (S2):

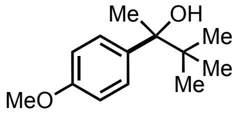

Synthesized according to ***GP3*** using pinacolone (5.0 mmol, 1.0 equiv.) and 4-bromoanisole (6.0 mmol, 1.2 equiv.). Purification via flash chromatography using silica gel (petroleum spirit / EtOAc 95:5) afforded **S2** as a white solid (364.6 mg, **35 %** yield). Spectroscopic data agrees with the reported literature.[17]

^{1}H NMR (600 MHz, $CDCl_3$) δ 7.29 (d, *J* = 8.8 Hz, 2H), 6.77 (d, *J* = 8.9 Hz, 2H), 3.74 (s, 3H), 1.51 (s, 3H), 1.50 (s, 1H), 0.85 (s, 9H) ppm.

1-(4-fluorophenoxy)-2-methylpropan-2-ol (S3):

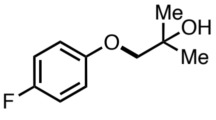

Synthesized according to ***GP4*** using 1,1-dimethyloxirane (30.0 mmol, 3.0 equiv.) and 4-fluorophenol (10.0 mmol, 1.0 equiv.). Purification via flash chromatography using silica gel (petroleum spirit / EtOAc 95:5) afforded **S3** as Colorless oil (1565.8 mg, **85 %** yield).

^{1}H NMR (600 MHz, $CDCl_3$) δ 7.00 – 6.95 (m, 2H), 6.88 – 6.84 (m, 2H), 3.75 (s, 2H), 2.22 (s, 1H), 1.34 (s, 6H) ppm.

^{13}C NMR (151 MHz, $CDCl_3$) δ 157.6 (d, *J* = 238.7 Hz), 155.1, 116.0 (d, *J* = 23.2 Hz), 115.8 (d, *J* = 8.0 Hz), 76.9, 70.2, 26.3 ppm.

HRMS (ESI) m/z: exact mass calculated for $C_{10}H_{14}FO_2^+$ $[(M+H)^+]$: 185,0972, Found: 185,0977.

1-(3-chloro-4-methylphenoxy)-2-methylpropan-2-ol (S4):

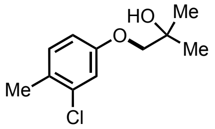

Synthesized according to ***GP4*** using 1,1-dimethyloxirane (9.0 mmol, 3.0 equiv.) and 3-chloro-4-methylphenol (3.0 mmol, 1.0 equiv.). afforded **S4** as a pale-yellow oil (625.1 mg, **97 %** yield).

^{1}H NMR (400 MHz, $CDCl_3$) δ 7.11 (dd, *J* = 8.4, 0.8 Hz, 1H), 6.94 (d, *J* = 2.6 Hz, 1H), 6.74 (dd, *J* = 8.4, 2.6 Hz, 1H), 3.75 (s, 2H), 2.30 (s, 3H), 2.18 (s, 1H), 1.33 (s, 6H) ppm.

^{13}C NMR (151 MHz, $CDCl_3$) δ 157.6, 134.7, 131.4, 128.5, 115.4, 113.4, 76.5, 70.2, 26.3, 19.2 ppm.

HRMS (ESI) m/z: exact mass calculated for $C_{11}H_{16}ClO_2^+$ $[(M+H)^+]$: 215,0833 , Found: 215,0839.

1-cyclohexyl-1-(4-methoxyphenyl)ethan-1-ol (S5):

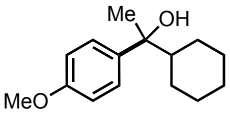

Synthesized according to ***GP3*** using 1-Cyclohexylethanone (3.0 mmol, 1.0 equiv.) and 4-bromoanisole (3.6 mmol, 1.2 equiv.). Purification via flash chromatography using silica gel (petroleum spirit / EtOAc 95:5) afforded **S5** as a colorless oil (407.5 mg, **58 %** yield). Spectroscopic data agrees with the literature.[18]

^{1}**H NMR** (400 MHz, $CDCl_3$) δ 7.35 – 7.28 (m, 2H), 6.89 – 6.82 (m, 2H), 3.81 (s, 3H), 1.77 – 1.62 (m, 4H), 1.61 – 1.53 (m, 3H), 1.51 (s, 3H), 1.23 – 1.12 (m, 2H), 1.07 (tt, *J* = 12.9, 3.1 Hz, 1H), 1.00 – 0.89 (m, 2H) ppm.

1-(4-methoxyphenyl)-1-(tetrahydro-2H-pyran-4-yl)ethan-1-ol (S6):

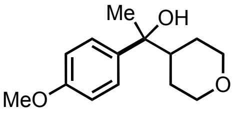

Synthesized according to ***GP3*** using 1-(tetrahydro-2*H*-pyran-4-yl)ethan-1-one (3.0 mmol, 1.0 equiv.) and 4-bromoanisole (3.6 mmol, 1.2 equiv.). Purification via flash chromatography using silica gel (petroleum spirit / EtOAc 95:5) afforded **S6** as a white solid (441.0 mg, **62 %** yield).

^{1}**H NMR** (600 MHz, $CDCl_3$) δ 7.33 – 7.30 (m, 2H), 6.89 – 6.85 (m, 2H), 3.99 – 3.93 (m, 2H), 3.81 (s, 3H), 3.34 – 3.27 (m, 2H), 1.82 – 1.76 (m, 1H), 1.61 (s, 1H), 1.53 (s, 3H), 1.52 – 1.49 (m, 1H), 1.45 – 1.37 (m, 3H) ppm.

^{13}**C NMR** (151 MHz, $CDCl_3$) δ 158.5, 139.3, 126.6, 113.5, 75.8, 68.4, 68.3, 55.4, 46.8, 27.6, 27.5, 26.5 ppm.

HRMS (ESI) m/z: exact mass calculated for $C_{14}H_{21}O_3^+$ [$(M+H)^+$]: 237,1485, Found: 237,1479.

2-(4-methoxyphenyl)-1-phenylpropan-2-ol (S7):

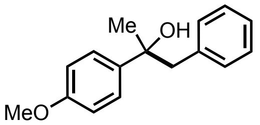

Synthesized according to ***GP3*** using 4-methoxyacetophenone (5.0 mmol, 1.0 equiv.) and benzyl bromide (6.0 mmol, 1.2 equiv.). Purification via flash chromatography using silica gel (petroleum spirit / EtOAc 95:5) afforded **S7** as a pale-yellow oil (523 mg, **43 %** yield). Spectroscopic data agrees with the literature.[19]

^{1}**H NMR** (400 MHz, $CDCl_3$) δ 7.34 – 7.28 (m, 2H), 7.24 – 7.18 (m, 3H), 7.00 (dd, *J* = 7.4, 2.2 Hz, 2H), 6.89 – 6.83 (m, 2H), 3.81 (s, 3H), 3.11 (d, *J* = 13.3 Hz, 1H), 3.00 (d, *J* = 13.2 Hz, 1H), 1.81 (s, 1H), 1.54 (s, 3H) ppm.

1-(4-chlorophenyl)-2-(4-methoxyphenyl)propan-2-ol (S8):

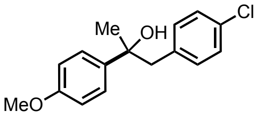

Synthesized according to ***GP3*** using 4- chlorophenyl acetone (3.0 mmol, 1.0 equiv.) and 4-bromoanisole (3.6 mmol, 1.2 equiv.). Purification via flash chromatography using silica gel (petroleum spirit / EtOAc 95:5) afforded **S8** as a white solid (472.0 mg, **57 %** yield). Spectroscopic data agrees with the literature.[20]

^{1}**H NMR** (400 MHz, $CDCl_3$) δ 7.27 (d, *J* = 8.9 Hz, 2H), 7.17 (d, *J* = 8.4 Hz, 2H), 6.89 (d, *J* = 8.4 Hz, 2H), 6.85 (d, *J* = 8.9 Hz, 2H), 3.81 (s, 3H), 3.05 (d, *J* = 13.3 Hz, 1H), 2.97 (d, *J* = 13.3 Hz, 1H), 1.73 (s, 1H), 1.54 (s, 3H) ppm.

^{13}**C NMR** (101 MHz, $CDCl_3$) δ 158.5, 139.4, 135.6, 132.6, 132.0, 128.2, 126.3, 113.5, 74.4, 55.4, 50.1, 29.5 ppm.

1-(4-fluorophenyl)-2-(4-methoxyphenyl)propan-2-ol (S9):

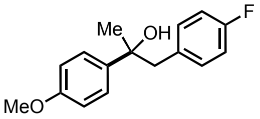

Synthesized according to ***GP3*** using 4- fluorophenyl acetone (3.0 mmol, 1.0 equiv.) and 4-bromoanisole (3.6 mmol, 1.2 equiv.). Purification via flash chromatography using silica gel (petroleum spirit / EtOAc 95:5) afforded **S9** as a white solid (576.0 mg, **74 %** yield).

^{1}H NMR (600 MHz, $CDCl_3$) δ 7.30 – 7.25 (m, 2H), 6.96 – 6.83 (m, 6H), 3.81 (s, 3H), 3.06 (d, *J* = 13.5 Hz, 1H), 2.98 (d, *J* = 13.5 Hz, 1H), 1.79 (s, 1H), 1.54 (s, 3H) ppm.

^{13}C NMR (151 MHz, $CDCl_3$) δ 161.9 (d, *J* = 244.6 Hz), 158.5, 139.6, 132.8, 132.1 (d, *J* = 7.8 Hz), 126.3, 114.9 (d, *J* = 21.0 Hz), 113.5, 74.4, 55.4, 49.9, 29.5 ppm.

HRMS (ESI) m/z: exact mass calculated for $C_{16}H_{16}FO_2^-$ [(M-H)$^-$]: 259,1140, Found: 259,1131.

2-(4-methoxyphenyl)-1-(p-tolyl)propan-2-ol (S10):

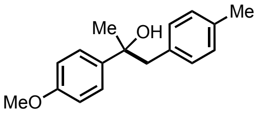

Synthesized according to ***GP3*** using 4-methoxyacetophenone (3.0 mmol, 1.0 equiv.) and 4-methyl benzyl bromide (4.5 mmol, 1.5 equiv.). Purification via flash chromatography using silica gel (petroleum spirit / EtOAc 95:5) afforded **S10** as a white solid (461.0 mg, **60 %** yield).

^{1}H NMR (600 MHz, $CDCl_3$) δ 7.33 – 7.29 (m, 2H), 7.05 – 7.01 (m, 2H), 6.90 – 6.84 (m, 4H), 3.81 (s, 3H), 3.07 (d, *J* = 13.3 Hz, 1H), 2.96 (d, *J* = 13.3 Hz, 1H), 2.30 (s, 3H), 1.81 (s, 1H), 1.53 (s, 3H) ppm.

^{13}C NMR (151 MHz, $CDCl_3$) δ 158.4, 140.1, 136.3, 133.8, 130.6, 128.9, 126.3, 113.5, 74.3, 55.4, 50.3, 29.6, 21.2 ppm.

HRMS (ESI) m/z: exact mass calculated for $C_{17}H_{21}O_2^+$ [(M+H)$^+$]: 257,1536, Found: 257,1553.

2-(4-methoxyphenyl)-1-(naphthalen-2-yl)propan-2-ol (S11):

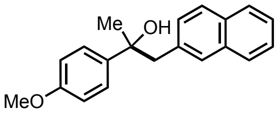

Synthesized according to ***GP3*** using 4-methoxyacetophenone (1.0 mmol, 1.0 equiv.) and 2-bromomethyl naphthalen (1.5 mmol, 1.5 equiv.). Purification via flash chromatography using silica gel (petroleum spirit / EtOAc 95:5) afforded **S11** as a white solid (281.0 mg, **96 %** yield).

^{1}H NMR (400 MHz, $CDCl_3$) δ 7.81 – 7.72 (m, 2H), 7.66 (d, *J* = 8.4 Hz, 1H), 7.54 – 7.50 (m, 1H), 7.48 – 7.40 (m, 2H), 7.36 – 7.30 (m, 2H), 7.04 (dd, *J* = 8.4, 1.7 Hz, 1H), 6.89 – 6.84 (m, 2H), 3.82 (s, 3H), 3.27 (d, *J* = 13.2 Hz, 1H), 3.17 (d, *J* = 13.3 Hz, 1H), 1.88 (s, 1H), 1.55 (s, 3H) ppm.

^{13}C NMR (151 MHz, $CDCl_3$) δ 158.5, 139.9, 134.7, 133.4, 132.4, 129.3, 129.2, 127.8, 127.7, 127.6, 126.4, 126.1, 125.7, 113.5, 74.5, 55.4, 50.9, 29.7 ppm.

HRMS (ESI) m/z: exact mass calculated for $C_{20}H_{21}O_2^+$ [(M+H)$^+$]: 293,1536, Found: 293,1555.

2-(4-methoxyphenyl)-1-(4-(trifluoromethyl)phenoxy)propan-2-ol (S12):

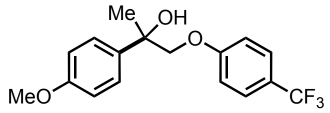

Synthesized according to ***GP3*** using 1-(4-(trifluoromethyl)phenoxy)propan-2-one (10.0 mmol, 1.0 equiv.) and 4-bromoanisole (12.0 mmol, 1.2 equiv.). Purification via flash chromatography using silica gel (petroleum spirit / EtOAc 95:5) afforded **S12** as a white solid (2.136 g, **65 %** yield).

^{1}H NMR (400 MHz, $CDCl_3$) δ 7.56 – 7.51 (m, 2H), 7.47 – 7.42 (m, 2H), 6.98 – 6.94 (m, 2H), 6.94 – 6.89 (m, 2H), 4.12 (d, *J* = 9.0 Hz, 1H), 4.02 (d, *J* = 9.1 Hz, 1H), 3.82 (s, 3H), 2.64 (s, 1H), 1.68 (s, 3H) ppm.

^{13}C NMR (151 MHz, $CDCl_3$) δ 161.2, 159.1, 136.5, 127.1 (q, *J* = 4.0 Hz), 126.5, 124.5 (q, *J* = 271.0 Hz), 123.6 (q, *J* = 32.8 Hz), 114.8, 113.9, 76.1, 73.5, 55.4, 26.6 ppm.

HRMS (ESI) m/z: exact mass calculated for $C_{17}H_{16}F_3O_3^-$ [(M-H)$^-$]: 325,1057, Found: 325,1043.

2-(4-methoxyphenyl)-1-(p-tolyloxy)propan-2-ol (S13):

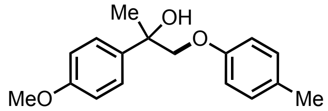

Synthesized according to ***GP4*** using p-cresol (2.4 mmol, 1.2 equiv.), 2-(4-methoxyphenyl)-2-methyloxirane (2.0 mmol, 1.0 equiv.), K_2CO_3 (4.8 mmol, 2.4 equiv.), reaction time 2 h. Purification via flash chromatography using silica gel (petroleum spirit / EtOAc 95:5) afforded **S13** as a colorless oil (117.0 mg, **22 %** yield).

^{1}H NMR (400 MHz, $CDCl_3$) δ 7.47 – 7.42 (m, 2H), 7.09 – 7.03 (m, 2H), 6.92 – 6.88 (m, 2H), 6.82 – 6.77 (m, 2H), 4.06 (d, *J* = 9.1 Hz, 1H), 3.96 (d, *J* = 9.0 Hz, 1H), 3.81 (s, 3H), 2.77 (s, 1H), 2.28 (s, 3H), 1.64 (s, 3H) ppm.

^{13}C NMR (151 MHz, $CDCl_3$) δ 158.9, 156.7, 137.1, 130.6, 130.1, 126.5, 114.7, 113.8, 76.2, 73.6, 55.4, 26.7, 20.6 ppm.

HRMS (ESI) m/z: exact mass calculated for $C_{17}H_{19}O_3^-$ [(M-H)$^-$]: 271,1340, Found: 271,1348.

1-(4-chlorophenoxy)-2-(4-methoxyphenyl)propan-2-ol (S14):

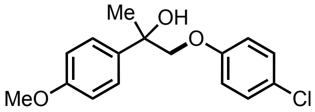

Synthesized according to ***GP4*** using 4-chlorophenol (2.4 mmol, 1.2 equiv.), 2-(4-methoxyphenyl)-2-methyloxirane (2.0 mmol, 1.0 equiv.), K_2CO_3 (4.8 mmol, 2.4 equiv.), reaction time 2 h. Purification via flash chromatography using silica gel (petroleum spirit / EtOAc 95:5) afforded **S14** as a colorless oil (208.0 mg, **35 %** yield).

^{1}H NMR (600 MHz, $CDCl_3$) δ 7.47 – 7.41 (m, 2H), 7.25 – 7.20 (m, 2H), 6.93 – 6.89 (m, 2H), 6.84 – 6.80 (m, 2H), 4.05 (d, *J* = 9.0 Hz, 1H), 3.96 (d, *J* = 9.0 Hz, 1H), 3.82 (s, 3H), 2.66 (s, 1H), 1.65 (s, 3H) ppm.

^{13}C NMR (151 MHz, $CDCl_3$) δ 159.0, 157.4, 136.7, 129.5, 126.5, 126.3, 116.2, 113.8, 76.4, 73.5, 55.4, 26.7 ppm.

HRMS (ESI) m/z: exact mass calculated for $C_{16}H_{16}ClO_3^-$ [(M-H)$^-$]: 291,0793, Found: 291,0786.

(4-methoxyphenyl)((3aR,5aS,8aS,8bR)-2,2,7,7-tetramethyltetrahydro-5H-bis([1,3]dioxolo)[4,5-b:4',5'-d]pyran-5-yl)methanol (S15):

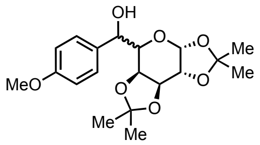

dr 1:1.2

To a nitrogen-purged, oven-dried 100 mL round-bottom flask containing aldehyde (1.66 g, 6.43 mmol) in dry Et_2O (15 mL), cooled to 0-5° C in an ice bath, 4-methoxyphenylmagnesium bromide (19.3 mL, 9.65 mmol, 0.5 M in THF) was added dropwise over 5 minutes. The reaction was stirred at that temperature for 15 minutes and then was allowed to warm to room temperature slowly. After 2 hours of stirring, saturated NH_4Cl was slowly added while keeping the reaction flask in an ice bath. Then the layers were separated. The aqueous layer was extracted with Et_2O (2*25 mL) and then combined organic extracts with brine, dried over anhydrous Na_2SO_4. After completion, the mixture was concentrated *in vacuo* and purified by column chromatography (pentane/EtOAc 4:1) to obtain the desired product. *dr* 1:1.2, based on ^{1}H NMR of the crude reaction mixture. Reaction was conducted following the reported literature procedure.[21]

Compound **S15** top diastereomer (810 mg, **34 %** yield) obtained as a colorless oil after flash chromatography (Pentane/EtOAc 4:1).

^{1}H NMR (600 MHz, $CDCl_3$) δ 7.43 – 7.37 (m, 2H), 6.91 – 6.87 (m, 2H), 5.61 (d, J = 5.1 Hz, 1H), 4.84 (d, J = 8.4 Hz, 1H), 4.45 (dd, J = 8.0, 2.3 Hz, 1H), 4.29 (dd, J = 5.0, 2.3 Hz, 1H), 3.82 (dd, J = 8.0, 1.6 Hz, 1H), 3.80 (s, 3H), 3.72 (dd, J = 8.4, 1.6 Hz, 1H), 3.05 (d, J = 1.0 Hz, 1H), 1.53 (s, 3H), 1.50 (s, 3H), 1.32 (s, 3H), 1.27 (s, 3H) ppm.

^{13}C NMR (151 MHz, $CDCl_3$) δ 159.5, 131.4, 128.6, 113.8, 109.4, 108.9, 96.6, 72.9, 72.3, 71.0, 70.9, 70.6, 55.3, 26.2, 26.1, 25.1, 24.3 ppm.

HRMS (ESI) m/z: exact mass calculated for $C_{19}H_{26}O_7Na^+$ [(M+Na)$^+$]: 389.1571, Found: 389.1588.

Compound **S15** Low diastereomer (1.00 g, **43 %** yield) obtained as a colorless oil after flash chromatography (Pentane/EtOAc 4:1).

^{1}H NMR (600 MHz, $CDCl_3$) δ 7.35 – 7.30 (m, 2H), 6.90 – 6.85 (m, 2H), 5.51 (d, J = 5.0 Hz, 1H), 4.84 (t, J = 6.0 Hz, 1H), 4.57 (dd, J = 8.1, 2.4 Hz, 1H), 4.41 (dd, J = 8.1, 1.8 Hz, 1H), 4.27 (dd, J = 5.1, 2.4 Hz, 1H), 3.88 (dd, J = 7.1, 1.8 Hz, 1H), 3.78 (s, 3H), 3.23 (d, J = 5.6 Hz, 1H), 1.50 (s, 3H), 1.48 (s, 3H), 1.34 (s, 3H), 1.28 (s, 3H) ppm.

^{13}C NMR (151 MHz, $CDCl_3$) δ 159.2, 133.6, 127.9, 113.8, 109.5, 108.6, 96.7, 73.3, 71.2, 70.9, 70.6, 69.9, 55.3, 26.0, 26.0, 24.9, 24.4.ppm.

HRMS (ESI) m/z: exact mass calculated for $C_{19}H_{26}O_7Na^+$ [(M+Na)$^+$]: 389.1571, Found: 389.1590.

The NMR data for both diastereomers aligns well with the values reported in the literature.[22]

(1R,2S,5R)-2-isopropyl-5-methylcyclohexyl 4-bromobenzoate (S16):

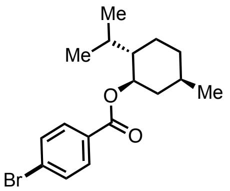

Synthesized according to reported procedure[23] : add the solution of 4-bromobenzoyl chloride (5.0 mmol, 1.0 equiv.) in DCM (10 mL) to a solution of L-menthol (5.0 mmol, 1.0 equiv.), DMAP (0.5 mmol, 0.1 equiv.) and Et_3N (10.0 mmol, 2.0 equiv.) in DCM (10 mL) dropwise using a syringe at 0°C. Stir the reaction mixture for 30 min at 0°C then allow the mixture to stir at room temperature overnight. Dilute the mixture with saturated NH_4Cl solution (20 mL), extract the mixture with DCM (3 x 10 mL), wash the organic layer with Brine, dry over Na_2SO_4, evaporate the solvent. Purification via flash chromatography using silica gel (petroleum spirit / EtOAc 20:1) afforded **S16** as a white solid (1.53 g, **90 %** yield). Spectroscopic data agrees with the literature.[23]

^{1}H NMR (600 MHz, $CDCl_3$) δ 7.92 – 7.88 (m, 2H), 7.60 – 7.55 (m, 2H), 4.92 (td, *J* = 10.9, 4.4 Hz, 1H), 2.14 – 2.09 (m, 1H), 1.96 – 1.88 (m, 1H), 1.76 – 1.70 (m, 2H), 1.62 – 1.50 (m, 2H), 1.17 – 1.06 (m, 2H), 0.96 – 0.89 (m, 7H), 0.79 (d, *J* = 7.0 Hz, 3H) ppm.

^{13}C NMR (151 MHz, $CDCl_3$) δ 165.5, 131.8, 131.3, 129.9, 127.9, 75.4, 47.4, 41.1, 34.4, 31.6, 26.7, 23.8, 22.2, 20.9, 16.7 ppm.

1-cyclopentyl-1-(4-methoxyphenyl)ethan-1-ol (S17):

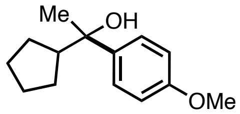

Synthesized according to ***GP3*** using 1-cyclopentylethanone (5.0 mmol, 1.0 equiv.) and 4-bromoanisole (5.5 mmol, 1.1 equiv.). Purification via flash chromatography using silica gel (petroleum spirit / EtOAc 95:5) afforded **S17** as a pale-yellow oil (660.1 mg, **60 %** yield).

^{1}H NMR (600 MHz, $CDCl_3$) δ 7.38 – 7.34 (m, 2H), 6.88 – 6.84 (m, 2H), 3.80 (s, 3H), 2.33 – 2.26 (m, 1H), 1.72 – 1.66 (m, 1H), 1.60 – 1.56 (m, 2H), 1.54 – 1.44 (m, 4H), 1.52 (s, 3H), 1.41 – 1.35 (m, 1H), 1.29 – 1.23 (m, 1H) ppm.

^{13}C NMR (151 MHz, $CDCl_3$) δ 158.1, 140.9, 126.3, 113.3, 75.5, 55.4, 51.3, 29.1, 27.5, 27.2, 26.1, 25.8 ppm.

HRMS (ESI) m/z: exact mass calculated for $C_{14}H_{21}O_2^+$ [$(M+H)^+$]: 221,1536, Found: 221,1553.

2-(4-methoxyphenyl)-3-methylbutan-2-ol (S18):

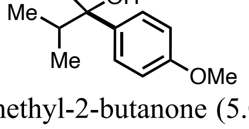

Synthesized according to ***GP3*** using 3-methyl-2-butanone (5.0 mmol, 1.0 equiv.) and 4-bromoanisole (6.0 mmol, 1.2 equiv.). Purification via flash chromatography using silica gel (petroleum spirit / EtOAc 95:5) afforded **S18** as a colorless oil (823 mg, **85 %** yield). Spectroscopic data agrees with the reported literature.[24]

^{1}H NMR (400 MHz, $CDCl_3$) δ 7.37 – 7.30 (m, 2H), 6.89 – 6.84 (m, 2H), 3.81 (s, 3H), 1.99 (sept, *J* = 6.8 Hz, 1H), 1.58 (brs, 1H), 1.51 (s, 3H), 0.86 (d, *J* = 6.8 Hz, 3H), 0.82 (d, *J* = 6.8 Hz, 3H) ppm.

1-(4-methoxyphenyl)pentan-1-ol (S19):

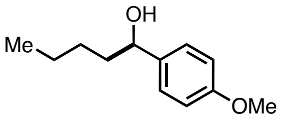

Synthesized according to ***GP3*** using p-anisaldehyde (5.0 mmol, 1.0 equiv.) and butyl magnesium chloride (6.0 mmol, 1.2 equiv.). Purification via flash chromatography using silica gel (petroleum spirit / EtOAc 95:5) afforded **S19** as a white solid (996 mg, **96 %** yield). Spectroscopic data agrees with the reported literature.[25]

^{1}H NMR (600 MHz, $CDCl_3$) δ 7.27 (d, J = 8.6 Hz, 2H), 6.88 (d, J = 8.6 Hz, 2H), 4.61 (t, J = 6.8 Hz, 1H), 3.81 (s, 3H), 1.84 – 1.77 (m, 1H), 1.74 (s, 1H), 1.72 – 1.65 (m, 1H), 1.41 – 1.30 (m, 3H), 1.26 – 1.19 (m, 1H), 0.88 (t, J = 7.2 Hz, 3H). ppm.

^{13}C NMR (151 MHz, $CDCl_3$) δ 159.1, 137.2, 127.3, 113.9, 74.5, 55.4, 38.8, 28.2, 22.8, 14.2 ppm.

1-(furan-2-yl)-2-(4-methoxyphenyl)propan-2-ol (S20):

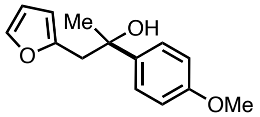

Synthesized according to ***GP3*** using 1-(2-Furyl)acetone (5.0 mmol, 1.0 equiv.) and 4-bromoanisole (5.5 mmol, 1.2 equiv.). Purification via flash chromatography using silica gel (petroleum spirit / EtOAc 95:5) afforded **S20** as a colorless oil (636.0 mg, **55 %** yield).

^{1}H NMR (600 MHz, $CDCl_3$) δ 7.38 – 7.34 (m, 2H), 7.31 (dd, J = 1.9, 0.8 Hz, 1H), 6.88 – 6.84 (m, 2H), 6.26 (dd, J = 3.2, 1.9 Hz, 1H), 5.98 – 5.94 (m, 1H), 3.80 (s, 3H), 3.14 (d, J = 14.9 Hz, 1H), 3.08 (d, J = 14.9 Hz, 1H), 2.31 (s, 1H), 1.54 (s, 3H) ppm.

^{13}C NMR (151 MHz, $CDCl_3$) δ 158.5, 152.2, 141.9, 139.6, 126.1, 113.5, 110.4, 108.3, 74.0, 55.4, 42.9, 29.7 ppm.

HRMS (ESI) m/z: exact mass calculated for $C_{14}H_{17}O_3^+$ [$(M+H)^+$]: 233,1172, not detected using ESI. **GC-MS (EI, 70 eV)** m/z: exact mass calculated for $C_{14}H_{16}O_3^+$ [M^+]: 232,1094, 232.1 ([M^+], 1.0), 214.1 (10.3), 151.1 (100.0), 135.1 (8.6), 109.1 (6.4), 77.1 (3.8), 43.1 (15.3).

1-(4-methoxyphenyl)-2-(phenylthio)ethan-1-ol (S21):

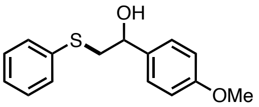

Synthesized according to ***GP2*** using α-Bromo-4-methoxyacetophenone (5.0 mmol, 1.0 equiv.) and thiophenol (5.0 mmol, 1.0 equiv.). Purification via flash chromatography using silica gel (petroleum spirit / EtOAc 95:5) afforded **S21** as a pale-yellow oil (806 mg, **62 %** yield). Spectroscopic data agrees with the literature.[26]

^{1}H NMR (600 MHz, $CDCl_3$) δ 7.44 – 7.40 (m, 2H), 7.34 – 7.27 (m, 4H), 7.25 – 7.22 (m, 1H), 6.91 – 6.86 (m, 2H), 4.69 (dt, J = 9.4, 3.0 Hz, 1H), 3.80 (s, 3H), 3.30 (dd, J = 13.8, 3.7 Hz, 1H), 3.10 (dd, J = 13.8, 9.3 Hz, 1H), 2.76 (d, J = 2.4 Hz, 1H) ppm.

^{13}C NMR (151 MHz, $CDCl_3$) δ 159.5, 135.1, 134.4, 130.3, 129.3, 127.3, 126.9, 114.1, 71.5, 55.4, 44.1 ppm.

2-((4-fluorophenyl)thio)-1-(4-methoxyphenyl)ethan-1-ol (S22):

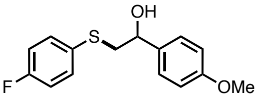

Synthesized according to ***GP2*** using α-Bromo-4-methoxyacetophenone (5.0 mmol, 1.0 equiv.) and 4-Fluorothiophenol (5.0 mmol, 1.0 equiv.). Purification via flash chromatography using silica gel (petroleum spirit / EtOAc 95:5) afforded **S22** as a pale-yellow oil (730 mg, **52 %** yield).

^{1}H NMR (600 MHz, $CDCl_3$) δ 7.43 – 7.37 (m, 2H), 7.26 – 7.22 (m, 2H), 7.04 – 6.98 (m, 2H), 6.89 – 6.84 (m, 2H), 4.64 (dd, *J* = 9.2, 3.9 Hz, 1H), 3.79 (s, 3H), 3.20 (dd, *J* = 13.7, 3.9 Hz, 1H), 3.07 (dd, *J* = 13.7, 9.1 Hz, 1H), 2.84 (s, 1H) ppm.

^{13}C NMR (151 MHz, $CDCl_3$) δ 162.2 (d, *J* = 247.2 Hz), 159.5, 134.3, 133.2 (d, *J* = 8.0 Hz), 130.1, 127.3, 116.3 (d, *J* = 21.9 Hz), 114.1, 71.5, 55.4, 45.1 ppm.

HRMS (ESI) m/z: exact mass calculated for $C_{15}H_{16}FO_2S^+$ [$(M+H)^+$]: 279,0850, Found: 279,0870.

2-(4-chlorophenoxy)-1-(4-methoxyphenyl)ethan-1-ol (S23):

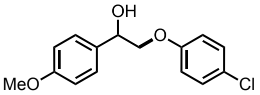

Synthesized according to ***GP2*** using α-bromo-4-methoxyacetophenone (5.0 mmol, 1.0 equiv.) and 4-chlorophenol (5.0 mmol, 1.0 equiv.). Purification via flash chromatography using silica gel (petroleum spirit / EtOAc 95:5) afforded **S23** as a white solid (1.351 g, **96 %** yield). Spectroscopic data agrees with the literature.[27]

^{1}H NMR (400 MHz, $CDCl_3$) δ 7.37 – 7.31 (m, 2H), 7.25 – 7.18 (m, 2H), 6.94 – 6.88 (m, 2H), 6.84 – 6.78 (m, 2H), 5.02 (dd, *J* = 7.7, 4.3 Hz, 1H), 4.03 – 3.94 (m, 2H), 3.80 (s, 3H), 3.34 (s, 1H) ppm.

^{13}C NMR (101 MHz, $CDCl_3$) δ 159.4, 157.0, 131.8, 129.3, 127.5, 125.9, 115.9, 113.9, 73.5, 71.9, 55.2 ppm.

1-(4-methoxyphenyl)-2-(p-tolyloxy)ethan-1-ol (S24):

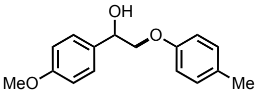

Synthesized according to ***GP2*** using α-bromo-4-methoxyacetophenone (5.0 mmol, 1.0 equiv.) and p-sol (5.0 mmol, 1.0 equiv.). Purification via flash chromatography using silica gel (petroleum spirit / EtOAc 95:5) afforded **S24** as a white solid (1.211 g, **94 %** yield). Spectroscopic data agrees with the literature.[27]

^{1}H NMR (600 MHz, $CDCl_3$) δ 7.37 (d, *J* = 8.6 Hz, 2H), 7.08 (d, *J* = 8.6 Hz, 2H), 6.92 (d, *J* = 8.7 Hz, 2H), 6.82 (d, *J* = 8.6 Hz, 2H), 5.06 (dt, *J* = 9.0, 2.8 Hz, 1H), 4.04 (dd, *J* = 9.6, 3.2 Hz, 1H), 3.96 (t, *J* = 9.3 Hz, 1H), 3.82 (s, 3H), 2.73 (d, *J* = 2.4 Hz, 1H), 2.29 (s, 3H) ppm.

^{13}C NMR (151 MHz, $CDCl_3$) δ 159.7, 156.4, 131.9, 130.7, 130.1, 127.7, 114.6, 114.1, 73.6, 72.4, 55.5, 20.6 ppm.

2-(4-fluorophenoxy)-1-(4-methoxyphenyl)ethan-1-ol (S25):

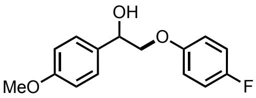

Synthesized according to ***GP2*** using α-bromo-4-methoxyacetophenone (5.0 mmol, 1.0 equiv.) and 4-Fluorophenol (5.0 mmol, 1.0 equiv.). Purification via flash chromatography using silica gel (petroleum spirit / EtOAc 95:5) afforded **S25** as a white solid (1.283 g, **97 %** yield). Spectroscopic data agrees with the literature.[27]

^{1}H NMR (600 MHz, $CDCl_3$) δ 7.37 (d, *J* = 8.7 Hz, 2H), 7.00 – 6.95 (m, 2H), 6.93 (d, *J* = 8.7 Hz, 2H), 6.89 – 6.84 (m, 2H), 5.06 (dt, *J* = 8.8, 2.9 Hz, 1H), 4.03 (dd, *J* = 9.5, 3.3 Hz, 1H), 3.96 (t, *J* = 9.1 Hz, 1H), 3.82 (s, 3H), 2.68 (d, *J* = 2.5 Hz, 1H) ppm.

^{13}C NMR (151 MHz, $CDCl_3$) δ 159.7, 157.7 (d, *J* = 238.9 Hz), 154.7, 131.8, 127.7, 116.1 (d, *J* = 23.1 Hz), 115.8 (d, *J* = 8.1 Hz), 114.2, 74.1, 72.3, 55.5 ppm.

1-(4-methoxyphenyl)-2-(methyl(phenyl)amino)ethan-1-ol (S26):

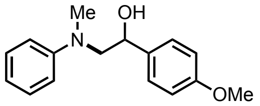

Synthesized according to ***GP2*** using α-bromo-4-methoxyacetophenone (5.0 mmol, 1.0 equiv.) and 4-methylaniline (5.0 mmol, 1.0 equiv.). Purification via flash chromatography using silica gel (petroleum spirit / EtOAc 95:5) afforded **S26** as a pale-yellow oil (1.245 g, **97 %** yield). Spectroscopic data agrees with the literature.[27]

^{1}H NMR (600 MHz, $CDCl_3$) δ 7.37 – 7.33 (m, 2H), 7.30 – 7.24 (m, 2H), 6.94 – 6.90 (m, 2H), 6.88 – 6.83 (m, 2H), 6.81 – 6.76 (m, 1H), 4.96 (ddd, *J* = 8.8, 4.2, 2.0 Hz, 1H), 3.82 (s, 3H), 3.51 (dd, *J* = 14.7, 9.0 Hz, 1H), 3.40 (dd, *J* = 14.7, 4.2 Hz, 1H), 2.94 (s, 3H), 2.46 (d, *J* = 2.1 Hz, 1H) ppm.

^{13}C NMR (151 MHz, $CDCl_3$) δ 159.4, 150.1, 134.2, 129.4, 127.3, 117.6, 114.1, 113.4, 71.5, 62.1, 55.5, 39.5 ppm.

Cyclohexyl(4-methoxyphenyl)methanol (S27):

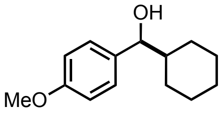

Synthesized according to ***GP3*** using p-anisaldehyde (2.0 mmol, 1.0 equiv.) and bromocyclohexane (4.0 mmol, 2.0 equiv.). Purification via flash chromatography using silica gel (petroleum spirit / EtOAc 95:5) afforded **S27** as a white solid (300.5 mg, **68 %** yield). Spectroscopic data agrees with the reported literature.[18]

^{1}H NMR (400 MHz, $CDCl_3$) δ 7.22 (d, *J* = 8.6 Hz, 2H), 6.87 (d, *J* = 8.6 Hz, 2H), 4.31 (dd, *J* = 7.5, 3.0 Hz, 1H), 3.81 (s, 3H), 2.05 – 1.96 (m, 1H), 1.81 – 1.75 (m, 1H), 1.74 (d, *J* = 3.1 Hz, 1H), 1.70 – 1.57 (m, 3H), 1.39 – 1.31 (m, 1H), 1.30 – 1.08 (m, 3H), 1.08 – 0.97 (m, 1H), 0.94 – 0.83 (m, 1H) ppm.

2-hydroxy-2-(4-methoxyphenyl)ethyl benzoate (S28):

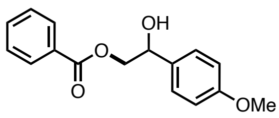

The synthesis was carried out according to **GP2** using α-bromo-4-methoxyacetophenone (5.0 mmol, 1.0 equiv.) and benzoic acid (5.0 mmol, 1.0 equiv.) to obtain the crude ketone product. The resulting β-ketoester, without purification, was dissolved in dry THF (10 mL) and stirred under a nitrogen atmosphere at 0 °C. BH_3•DMS (2 equiv.), dissolved in dry THF (2 mL), was added dropwise over 1 hour. After the addition, the reaction mixture was stirred for an additional hour at room temperature.

The reaction was quenched with MeOH (10 mL) at 0 °C. The solvent was removed under vacuum, and the residue was treated with water (25 mL) and extracted with EtOAc (3 × 50 mL). The organic layers were washed with brine, dried over Na_2SO_4, and concentrated under reduced pressure to afford the crude product. Purification by flash chromatography on silica gel (petroleum spirit/EtOAc, 9:1) yielded compound **S28** as a white solid (736 mg, **60 %** yield). Spectroscopic data agrees with the reported literature.[28]

^{1}H NMR (600 MHz, $CDCl_3$) δ 8.06 (d, *J* = 7.9 Hz, 2H), 7.58 (t, *J* = 7.5 Hz, 1H), 7.45 (t, *J* = 7.7 Hz, 2H), 7.38 (d, *J* = 8.7 Hz, 2H), 6.92 (d, *J* = 8.7 Hz, 2H), 5.07 (dt, *J* = 8.2, 3.2 Hz, 1H), 4.49 (dd, *J* = 11.5, 3.5 Hz, 1H), 4.42 (dd, *J* = 11.5, 8.3 Hz, 1H), 3.82 (s, 3H), 2.52 (d, *J* = 3.2 Hz, 1H) ppm.

^{13}C NMR (151 MHz, $CDCl_3$) δ 166.9, 159.7, 133.4, 132.1, 130.0, 129.9, 128.6, 127.6, 114.2, 72.3, 69.9, 55.5 ppm.

2-hydroxy-2-(4-methoxyphenyl)ethyl thiophene-2-carboxylate (S29):

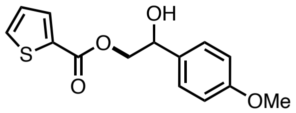

The synthesis was carried out according to **GP2** using α-bromo-4-methoxyacetophenone (5.0 mmol, 1.0 equiv.) and 2-thiophenecarboxylic acid (5.0 mmol, 1.0 equiv.) to obtain the crude ketone product. The resulting β-ketoester, without purification, was dissolved in dry THF (10 mL) and stirred under a nitrogen atmosphere at 0 °C. BH_3•DMS (2 equiv.), dissolved in dry THF (2 mL), was added dropwise over 1 hour. After the addition, the reaction mixture was stirred for an additional hour at room temperature.

The reaction was quenched with MeOH (10 mL) at 0 °C. The solvent was removed under vacuum, and the residue was treated with water (25 mL) and extracted with EtOAc (3 × 50 mL). The organic layers were washed with brine, dried over Na_2SO_4, and concentrated under reduced pressure to afford the crude product. Purification by flash chromatography on silica gel (petroleum spirit/EtOAc, 9:1) yielded compound **S29** as a white solid (1.078 g, **77 %** yield).

^{1}H NMR (600 MHz, $CDCl_3$) δ 7.84 (dd, *J* = 3.7, 1.3 Hz, 1H), 7.59 (dd, *J* = 5.0, 1.3 Hz, 1H), 7.40 – 7.35 (m, 2H), 7.12 (dd, *J* = 5.0, 3.7 Hz, 1H), 6.95 – 6.89 (m, 2H), 5.04 (dt, *J* = 8.2, 3.3 Hz, 1H), 4.46 (dd, *J* = 11.5, 3.5 Hz, 1H), 4.38 (dd, *J* = 11.5, 8.3 Hz, 1H), 3.82 (s, 3H), 2.49 (d, *J* = 3.2 Hz, 1H) ppm.

^{13}C NMR (151 MHz, $CDCl_3$) δ 162.4, 159.7, 134.0, 133.4, 132.9, 132.0, 128.0, 127.6, 114.2, 72.2, 70.0, 55.5 ppm.

HRMS (ESI) m/z: exact mass calculated for $C_{14}H_{15}O_4S^+$ [$(M+H)^+$]: 279,0686, Found: 279,0691.

2-hydroxy-2-(4-methoxyphenyl)ethyl 2-(2-fluoro-[1,1'-biphenyl]-4-yl)propanoate (S30):

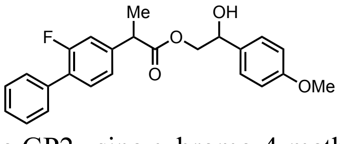

The synthesis was performed according to GP2 using α-bromo-4-methoxyacetophenone (2.0 mmol, 1.0 equiv.) and 2-(2-fluoro-[1,1'-biphenyl]-4-yl)propanoic acid (2.0 mmol, 1.0 equiv.) to obtain the crude ketone. The resulting β-ketoester, without purification, was dissolved in dry THF (10 mL) and stirred under a nitrogen atmosphere at 0 °C. BH_3•DMS (2.0 M in toluene, 1.0 mL, 1.0 equiv.) was added dropwise at 0 °C. After the addition, the reaction mixture was stirred for 30 minutes at 0 °C, then allowed to warm to room temperature and stirred overnight.

The reaction was quenched with ice at 0 °C, followed by the addition of ice-cold water (10 mL). The reaction residue was extracted with EtOAc (3 × 30 mL), and the combined organic layers were washed with brine, dried over Na_2SO_4, and concentrated under reduced pressure to afford the crude product. Purification by flash chromatography on silica gel (petroleum spirit/EtOAc, 8:2) yielded compound **S30** as a colorless oil (racemic mixture) (571 mg, **72 %** yield).

^{1}H NMR (600 MHz, CDCl3) δ 7.57 – 7.53 (m, 2H), 7.47 – 7.43 (m, 2H), 7.42 – 7.35 (m, 2H), 7.26 – 7.22 (m, 2H), 7.15 – 7.08 (m, 2H), 6.88 – 6.84 (m, 2H), 4.91 – 4.84 (m, 1H), 4.31 – 4.18 (m, 2H), 3.82 – 3.78 (m, 1H), 3.78 – 3.76 (m, 3H), 2.37 – 2.27 (m, 1H), 1.56 – 1.52 (m, 3H) ppm.

^{13}C NMR (151 MHz, $CDCl_3$) δ 174.2, 174.1, 159.8 (d, J = 248.5 Hz), 159.7, 141.7 (d, J = 7.4 Hz), 141.7 (d, J = 7.5 Hz), 135.6, 131.9 (d, J = 4.8 Hz), 131.0 (d, J = 3.8 Hz), 129.1 (d, J = 2.8 Hz), 128.6, 128.0 (d, J = 13.5 Hz), 127.8, 127.5, 123.7, 123.6, 123.6, 115.4 (d, J = 23.7 Hz), 114.1, 72.0, 69.6, 69.6, 55.4, 45.1, 45.1, 18.5 ppm.

HRMS (ESI) m/z: exact mass calculated for $C_{24}H_{24}FO_4^+$ [$(M+H)^+$]: 395,1653, Found: 395,1681.

2-hydroxy-2-(4-methoxyphenyl)ethyl 2-(4-isobutylphenyl)propanoate (S31):

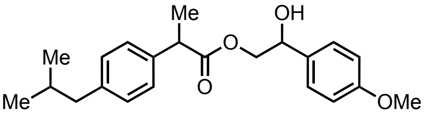

The synthesis was performed according to GP2 using α-bromo-4-methoxyacetophenone (2.0 mmol, 1.0 equiv.) and 2-(4-isobutylphenyl)propanoic acid (2.0 mmol, 1.0 equiv.) to obtain the crude ketone. The resulting β-ketoester, without purification, was dissolved in dry THF (10 mL) and stirred under a nitrogen atmosphere at 0 °C. BH_3•DMS (2.0 M in toluene, 1.0 mL, 1.0 equiv.) was added dropwise at 0 °C. After the addition, the reaction mixture was stirred for 30 minutes at 0 °C, then allowed to warm to room temperature and stirred overnight.

The reaction was quenched with ice at 0 °C, followed by the addition of ice-cold water (10 mL). The reaction residue was extracted with EtOAc (3 × 30 mL), and the combined organic layers were washed with brine, dried over Na_2SO_4, and concentrated under reduced pressure to afford the crude product. Purification by flash chromatography on silica gel (petroleum spirit/EtOAc, 8:2) yielded compound **S31** as a colorless oil (racemic mixture) (483mg, **68 %** yield).

^{1}H NMR (600 MHz, $CDCl_3$) δ 7.25 – 7.21 (m, 2H), 7.21 – 7.17 (m, 2H), 7.14 – 7.08 (m, 2H), 6.90 – 6.82 (m, 2H), 4.86 – 4.78 (m, 1H), 4.28 – 4.09 (m, 2H), 3.80 (s, 3H), 3.77 – 3.72 (m, 1H), 2.46 (d, J = 7.2 Hz, 2H), 2.38 – 2.27 (m, 1H), 1.91 – 1.81 (m, 1H), 1.50 (dd, J = 7.2, 0.9 Hz, 3H), 0.91 (d, J = 6.6 Hz, 6H) ppm.
^{13}C NMR (151 MHz, $CDCl_3$) δ 174.9, 174.9, 159.6, 140.8, 140.8, 137.7, 137.7, 131.9, 131.9, 129.6, 129.6, 127.6, 127.5, 127.3, 127.2, 114.0, 72.0, 71.9, 69.5, 69.5, 55.4, 45.2, 45.2, 45.2, 30.3, 22.5, 18.5 ppm.

HRMS (ESI) m/z: exact mass calculated for $C_{22}H_{29}O_4^+$ [$(M+H)^+$]: 357,2061, Found: 357,2083.

((3aR,5R,5aS,8aS,8bR)-2,2,7,7-tetramethyltetrahydro-5H-bis([1,3]dioxolo)[4,5-b:4',5'-d]pyran-5-yl)methyl 4-bromobenzoate (S32):

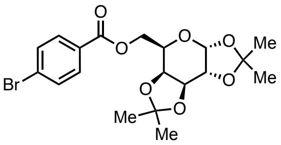

Following modified literature procedure:[29] ((3aR,5R,5aS,8aS,8bR)-2,2,7,7-tetramethyltetrahydro-5H-bis([1,3]dioxolo)[4,5-b:4',5'-d]pyran-5-yl)methanol (780 mg, 3.00 mmol) was dissolved in Et_3N (10 mL), followed by the addition of DMAP (73 mg, 0.60 mmol). The mixture was stirred for 5 minutes at room temperature before placing the flask in an ice bath. Into the solution 4-bromobenzoyl chloride (988 mg, 4.5 mmol) was added in portions and slowly the reaction temperature was elevated to room temperature. Then after stirring the reaction mixture for 2 h, 20 mL DCM was added to the reaction mixture. The organic layer was washed with 1N HCl followed by sturated $NaHCO_3$. Organic layer was collected, dried over anhydrous Na_2SO_4, concentrated *in vacuo* and was purified by column chromatography (pentane/EtOAc 4:1) to obtain the desired product **S32** as a white solid (1.11 g, **84 %** yield).

^{1}H NMR (600 MHz, $CDCl_3$) δ 7.90 – 7.86 (m, 2H), 7.57 – 7.53 (m, 2H), 5.54 (d, J = 5.0 Hz, 1H), 4.63 (dd, J = 7.9, 2.5 Hz, 1H), 4.50 (dd, J = 11.6, 4.6 Hz, 1H), 4.40 (dd, J = 11.5, 7.7 Hz, 1H), 4.33 (dd, J =

5.0, 2.5 Hz, 1H), 4.29 (dd, J = 7.9, 1.9 Hz, 1H), 4.17 – 4.12 (m, 1H), 1.48 (s, 3H), 1.45 (s, 3H), 1.33 (s, 3H), 1.31 (s, 3H). ppm.

^{13}C NMR (151 MHz, CDCl3) δ 165.8, 131.8, 131.3, 129.1, 128.2, 109.8, 108.9, 96.4, 71.2, 70.8, 70.6, 66.2, 64.3, 26.1, 26.1, 25.1, 24.6 ppm.

HRMS (ESI) m/z: exact mass calculated for $C_{19}H_{24}BrO_7^+$ [(M+H)$^+$]: 443,0700, Found: 443,0723. Experimental data is in agreement with the reported literature data.[30]

Ethyl (4-bromobenzoyl)-L-phenylalaninate (S33):

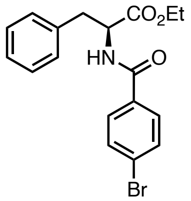

Following a modified literature procedure:[31] L-Phenylalanine ethyl ester hydrochloride (459 mg, 2.0 mmol), DMAP (24 mg, 0.2 mmol) and triethylamine (2.8 mL, 10.0 mmol) were combined in CH_2Cl_2 (5 mL) and stirred at 0 °C for 10 minutes. Then 4-bromobenzoyl chloride (658 mg, 3.0 mmol) was added in portions and the solution was stirred at 0 °C for an additional 1 h. Subsequently, the mixture was allowed to warm to room temperature and stirred for 5h. The mixture was washed with 1 M HCl solution (25 ml*2) and the organic layer was dried over Na_2SO_4, filtered, and the solvent was removed under vacuum, yielding a pale yellow solid. The crude product was purified by column chromatography (pentane/EtOAc 3:1) to obtain the desired product **S33** as a white solid (554 mg, 74% yield).

^{1}H NMR (600 MHz, $CDCl_3$) δ 7.63 – 7.59 (m, 2H), 7.59 – 7.55 (m, 2H), 7.35 – 7.24 (m, 3H), 7.19 – 7.12 (m, 2H), 6.60 (d, J = 7.5 Hz, 1H), 5.06 (dt, J = 7.5, 5.7 Hz, 1H), 4.24 (q, J = 7.1 Hz, 2H), 3.35 – 3.19 (m, 2H), 1.31 (t, J = 7.2 Hz, 3H) ppm.

^{13}C NMR (151 MHz, $CDCl_3$) δ 171.7, 166.0, 135.9, 132.9, 132.0, 129.5, 128.7, 128.7, 127.3, 126.6, 61.9, 53.7, 38.0, 14.3 ppm.

HRMS (ESI) m/z: exact mass calculated for $C_{18}H_{19}BrNO_3^+$ [(M+H)$^+$]: 376,0543, Found: 376,0564.

Methyl ((4-bromophenyl)sulfonyl)-L-prolinate (S34):

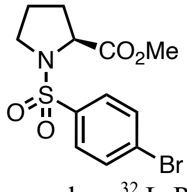

Following a slightly modified reported literature procedure,[32] L-Phenylalanine ethyl ester hydrochloride (331 mg, 2.0 mmol), DMAP (24 mg, 0.2 mmol) and triethylamine (2.8 mL, 10.0 mmol) were combined in CH_2Cl_2 (5 mL) and stirred at 0 °C for 10 minutes. Then 4-Bromobenzenesulfonyl chloride (766 mg, 3.0 mmol) was added in portions and the solution was stirred at 0 °C for an additional 1 h. Subsequently, the mixture was allowed to warm to room temperature and stirred for 5h. The mixture was washed with 1 M HCl solution (25 ml*2) and the organic layer was dried over Na_2SO_4, filtered, and the solvent was removed under vacuum, yielding a pale yellow solid. The crude product was purified by column chromatography (pentane/EtOAc 5:1) to obtain the desired product **S34** as a white solid (280 mg, 45% yield).

^{1}H NMR ^{1}H NMR (600 MHz, $CDCl_3$) δ 7.77 – 7.73 (m, 2H), 7.68 – 7.63 (m, 2H), 4.34 (dd, J = 8.6, 3.8 Hz, 1H), 3.71 (s, 3H), 3.48 – 3.42 (m, 1H), 3.37 – 3.31 (m, 1H), 2.13 – 2.05 (m, 1H), 2.04 – 1.93 (m, 2H), 1.87 – 1.78 (m, 1H).

^{13}C NMR (151 MHz, $CDCl_3$) δ 172.5, 137.7, 132.4, 129.1, 127.9, 60.5, 52.6, 48.5, 31.1, 24.8.

HRMS (ESI) m/z: exact mass calculated for $C_{12}H_{15}BrNO_4S^+$ $[(M+H)^+]$: 347,9900, Found: 347,9919.

Mechanistic study experiments

Emission spectra were recorded on a Fluorometer Horiba Max 4, with the excitation wavelength set at 430 nm, slits at 1 nm and integration time at 1 s.

TA measurements on the nanosecond timescale were performed as described previously[27] using an excitation wavelength of 430 nm and energies of ca. 7 mJ/pulse.

TA measurements on the femtosecond timescale were performed using a Ti:sapphire-based amplifier that included oscillator and pump lasers (Libra, Coherent). The laser’s fundamental output (800nm, 3 kHz) was divided into a pump and probe beams using a beam splitter, and these were directed towards a UV−vis−NIR TA spectrometer (TAS, Newport Corp.). The pump beam was frequency doubled to 400 nm through second harmonic generation. Before reaching the sample, the pump beam passed through a depolarizer and was attenuated with a neutral density filter. The probe supercontinuum was generated from a calcium fluoride crystal (UV−vis), and its path was controlled using an optical delay line with a time window of up to 8 ns. The broadband UV-Vis probe was then focused onto an optical fiber coupled to a MS260i spectrometer (Newport Corp.) after passing through the sample placed in quartz cuvette (1 mm pathlength or 2 mm pathlength together with magnetic stirring). Magnetic stirring was employed for samples susceptible to photodegradation. The instrument response function (IRF) was 130−150 fs. Sample absorbance was adjusted to approximately ∼0.3−0.4 at the excitation wavelength. The measurements were conducted with pump powers of 1-1.3 mW (0.67-0.87 mJ/pulse), focused on the sample in an approximately 0.1 mm^2 spot. The TA data were analyzed using Surface Xplorer software for background removal, time-zero correction, and for fitting the chirp to a third-order polynomial. The scans were carefully reviewed for inconsistencies, with no signs of photodamage. All experiments were performed in dichloroethane at an ambient temperature.

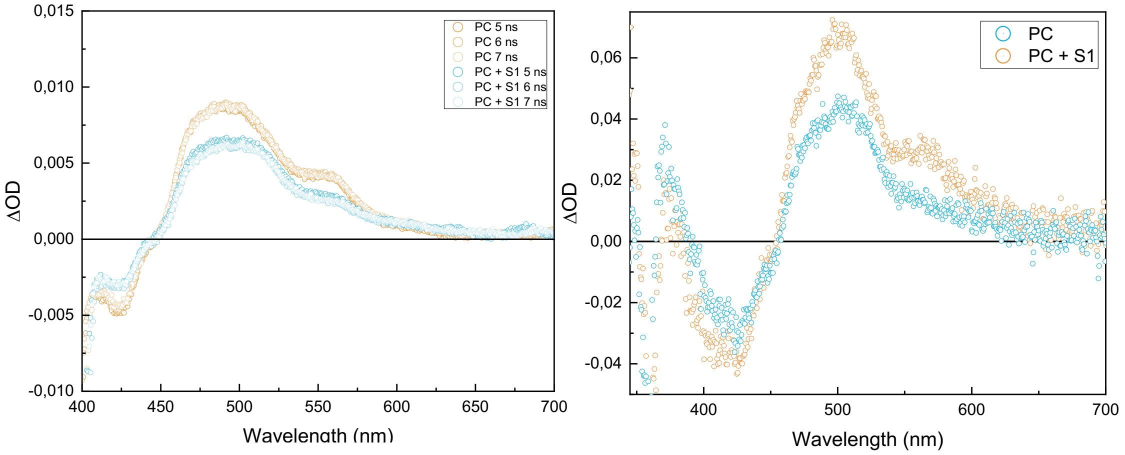

Figure S2. Transient absorption spectra of the Me-Acr$^+$-Mes photocatalyst (PC) with **S1** (40 mM)). Left: between 4 and 7 ns after laser excitation at 400 nm. Right: at 100 ns after laser excitation at 430 nm.

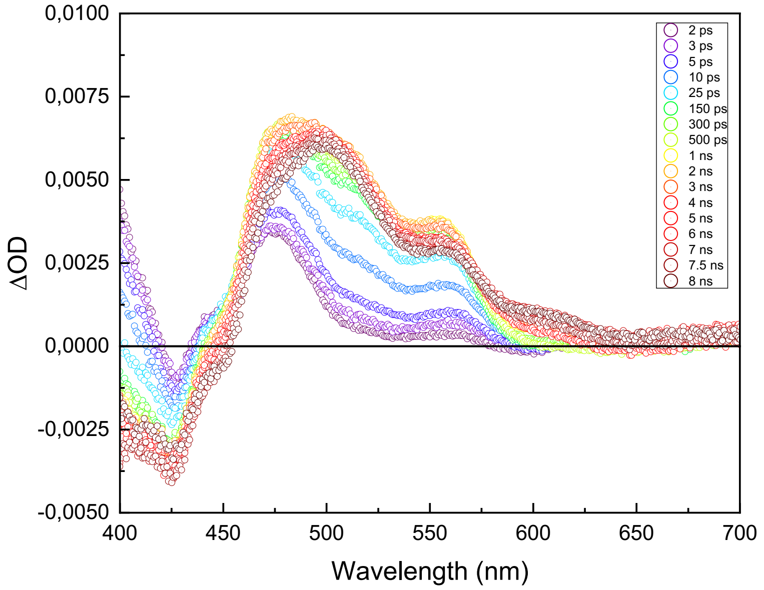

Figure S3. Transient absorption spectra of Me-Acr$^+$-Mes at various times after laser excitation at 400 nm.

Citation list

(1) Gao, Y.; Yang, C.; Bai, S.; Liu, X.; Wu, Q.; Wang, J.; Jiang, C.; Qi, X. Visible-Light-Induced Nickel-Catalyzed Cross-Coupling with Alkylzirconocenes from Unactivated Alkenes. *Chem* **2020**, *6* (3), 675-688.

(2) Chen, S.-C.; Zhu, Q.; Cao, Y.; Li, C.; Guo, Y.; Kong, L.; Che, J.; Guo, Z.; Chen, H.; Zhang, N.; Fang, X.; Lu, J.-T.; Luo, T. Dealkenylative Ni-Catalyzed Cross-Coupling Enabled by Tetrazine and Photoexcitation. *Journal of the American Chemical Society* **2021**, *143* (35), 14046-14052.

(3) Lv, X.-Y.; Abrams, R.; Martin, R. Dihydroquinazolinones as adaptative C(sp3) handles in arylations and alkylations via dual catalytic C–C bond-functionalization. *Nature Communications* **2022**, *13* (1), 2394.

(4) Bastick, K. A. C.; Watson, A. J. B. Pd-Catalyzed Homologation of Arylboronic Acids as a Platform for the Diversity-Oriented Synthesis of Benzylic C–X Bonds. *Synlett* **2023**, *34* (18), 2097-2102.

(5) Tan, L.; Pan, Y.; Zeng, Q.-Y.; Wang, Z.-Y.; Xu, H.; Dai, H.-X. Palladium-Catalyzed Directed Carbon–Carbon Bond Activation of Aryl Nitriles for Cyano Transfer. *Organic Letters* **2024**, *26* (11), 2260-2265.

(6) Xu, N.; Zhou, L.; Zhao, L.; Guo, M.; Yang, L.; Han, W. Iron-Catalyzed Oxidative C−C Bond Cleavage of Unstrained Tertiary Alkyl Aromatics to Arylketones. *Advanced Synthesis & Catalysis* **2023**, *365* (15), 2524-2529.

(7) Abuhafez, N.; Ehlers, A. W.; de Bruin, B.; Gramage-Doria, R. Markovnikov-Selective Cobalt-Catalyzed Wacker-Type Oxidation of Styrenes into Ketones under Ambient Conditions Enabled by Hydrogen Bonding. *Angewandte Chemie International Edition* **2024**, *63* (3), e202316825.

(8) Ni, P.; Yang, L.; Yang, J.; Cheng, R.; Zhu, W.; Ma, Y.; Ye, J. para-Selective, Direct C(sp2)–H Alkylation of Electron-Deficient Arenes by the Electroreduction Process. *The Journal of Organic Chemistry* **2023**, *88* (9), 5248-5253.

(9) Basch, C. H.; Liao, J.; Xu, J.; Piane, J. J.; Watson, M. P. Harnessing Alkyl Amines as Electrophiles for Nickel-Catalyzed Cross Couplings via C–N Bond Activation. *Journal of the American Chemical Society* **2017**, *139* (15), 5313-5316.

(10) Molander, G. A.; Traister, K. M.; O'Neill, B. T. Engaging Nonaromatic, Heterocyclic Tosylates in Reductive Cross-Coupling with Aryl and Heteroaryl Bromides. *The Journal of Organic Chemistry* **2015**, *80* (5), 2907-2911.

(11) Huang, L.; Ji, T.; Rueping, M. Remote Nickel-Catalyzed Cross-Coupling Arylation via Proton-Coupled Electron Transfer-Enabled C–C Bond Cleavage. *J. Am. Chem. Soc.* **2020**, *142* (7), 3532-3539.

(12) Tian, R.-G.; Wang, L.; Zheng, L.; Tian, S.-K. Nickel-Catalyzed Inter- and Intramolecular Reductive Cross-Coupling Reactions of Arylbenzylammonium Salts through Highly Site-Selective C–N Bond Cleavage. *ACS Catalysis* **2024**, *14* (7), 5039-5046.

(13) Cao, Z.-C.; Yu, D.-G.; Zhu, R.-Y.; Wei, J.-B.; Shi, Z.-J. Direct cross-coupling of benzyl alcohols to construct diarylmethanes via palladium catalysis. *Chemical Communications* **2015**, *51* (13), 2683-2686.

(14) Ackermann, L.; Kapdi, A. R.; Schulzke, C. Air-Stable Secondary Phosphine Oxide or Chloride (Pre)Ligands for Cross-Couplings of Unactivated Alkyl Chlorides. *Organic Letters* **2010**, *12* (10), 2298-2301.

(15) Sun, K.; Sun, T.; Jiang, Y.; Shi, J.; Sun, W.; Zheng, Y.; Wang, Z.; Li, Z.; Lv, X.; Zhang, X.; Luo, F.; Liu, S. Iron-catalyzed benzylic C–H thiolation via photoinduced ligand-to-metal charge-transfer. *Chemical Communications* **2024**, *60* (44), 5755-5758.

(16) Xiong, Y.; Zhang, X.; Guo, H.-M.; Wu, X. Photoredox/persistent radical cation dual catalysis for alkoxy radical generation from alcohols. *Organic Chemistry Frontiers* **2022**, *9* (13), 3532-3539.

(17) Lykakis, I. N.; Tanielian, C.; Orfanopoulos, M. Decatungstate Photocatalyzed Oxidation of Aryl Alkanols. Electron Transfer or Hydrogen Abstraction Mechanism? *Organic Letters* **2003**, *5* (16), 2875-2878.

(18) Wu, H.; Li, X.; Yang, L.; Chen, W.; Zou, C.; Deng, W.; Wang, Z.; Hu, J.; Li, Y.; Huang, Y. Cathodic Carbonyl Alkylation of Aryl Ketones or Aldehydes with Unactivated Alkyl Halides. *Organic Letters* **2022**, *24* (50), 9342-9347.

(19) Li, C.-C.; Dai, X.-J.; Wang, H.; Zhu, D.; Gao, J.; Li, C.-J. Iron-Catalyzed Nucleophilic Addition Reaction of Organic Carbanion Equivalents via Hydrazones. *Organic Letters* **2018**, *20* (13), 3801-3805.
(20) Altmann, L.-M.; Zantop, V.; Wenisch, P.; Diesendorf, N.; Heinrich, M. R. Visible Light Promoted, Catalyst-Free Radical Carbohydroxylation and Carboetherification under Mild Biomimetic Conditions. *Chemistry – A European Journal* **2021**, *27* (7), 2452-2462.
(21) Sasaki, E.; Lin, C.-I.; Lin, K.-Y.; Liu, H.-w. Construction of the Octose 8-Phosphate Intermediate in Lincomycin A Biosynthesis: Characterization of the Reactions Catalyzed by LmbR and LmbN. *Journal of the American Chemical Society* **2012**, *134* (42), 17432-17435.
(22) Wouters, A. D.; Bessa, A. B.; Sachini, M.; Wessjohann, L. A.; Luedtke, D. S. Boron–zinc exchange in the diastereoselective arylation of sugar-based aldehydes: Stereoselective synthesis of (+)-7-epi-goniofufurone and analogues. *Synthesis* **2013**, *45* (16), 2222-2233.
(23) Dan, X.; Yang, Q.; Xing, L.; Tang, Y.; Wang, W.; Cai, Y. Heterogeneous Metallaphotocatalytic C(sp2)–C(sp3) Cross-Coupling Reactions with Integrated Bipyridyl-Ni(II)-Carbon Nitride. *Organic Letters* **2023**, *25* (22), 4124-4129.
(24) Zong, H.; Huang, H.; Liu, J.; Bian, G.; Song, L. Added-Metal-Free Catalytic Nucleophilic Addition of Grignard Reagents to Ketones. *The Journal of Organic Chemistry* **2012**, *77* (10), 4645-4652.
(25) Shi, L.; Horn, M.; Kobayashi, S.; Mayr, H. Carbocationic n-endo-trig Cyclizations. *Chemistry – A European Journal* **2009**, *15* (34), 8533-8541.
(26) Das, A.; Thomas, K. R. J. Tuning Selectivity in the Visible-Light-Promoted Coupling of Thiols with Alkenes by EDA vs TOCO Complex Formation. *ACS Omega* **2023**, *8* (20), 18275-18289.
(27) Patehebieke, Y.; Charaf, R.; Bryce-Rogers, H. P.; Ye, K.; Ahlquist, M.; Hammarström, L.; Wallentin, C.-J. β-Scission of Secondary Alcohols via Photosensitization: Synthetic Utilization and Mechanistic Insights. *ACS Catal.* **2024**, *14* (1), 585-593.
(28) Tan, Y.-F.; Zhao, Y.-N.; Yang, D.; Lv, J.-F.; Guan, Z.; He, Y.-H. Electrochemical Synthesis of β-Iodoesters by 1,2-Iodoesterization of Unactivated Alkenes with Carboxylic Acids and Tetrabutylammonium Iodide. *The Journal of Organic Chemistry* **2023**, *88* (8), 5161-5171.
(29) Andersen, S. M.; Heuckendorff, M.; Jensen, H. H. 3-(Dimethylamino)-1-propylamine: A Cheap and Versatile Reagent for Removal of Byproducts in Carbohydrate Chemistry. *Organic Letters* **2015**, *17* (4), 944-947.
(30) Ahrweiler, E.; Schoetz, M. D.; Singh, G.; Bindschaedler, Q. P.; Sorroche, A.; Schoenebeck, F. Triply Selective & Sequential Diversification at Csp3: Expansion of Alkyl Germane Reactivity for C−C & C−Heteroatom Bond Formation. *Angewandte Chemie International Edition* **2024**, *63* (16), e202401545.
(31) Pugnal, L. V. B. L.; Vega, K. B.; Pissinati, E. F.; Correia, J. T. M.; Zukerman-Schpector, J.; Martin, R.; Paixão, M. W. 1,2-Arylsulfonylation of Vinyl Ethers and Esters Enabled by Dual Ni Catalysis. *ChemCatChem* **2023**, *15* (14), e202201434.
(32) Benediktsdottir, A.; Lu, L.; Cao, S.; Zamaratski, E.; Karlén, A.; Mowbray, S. L.; Hughes, D.; Sandström, A. Antibacterial sulfonimidamide-based oligopeptides as type I signal peptidase inhibitors: Synthesis and biological evaluation. *European Journal of Medicinal Chemistry* **2021**, *224*, 113699.

^{1}H NMR and ^{13}C NMR spectra

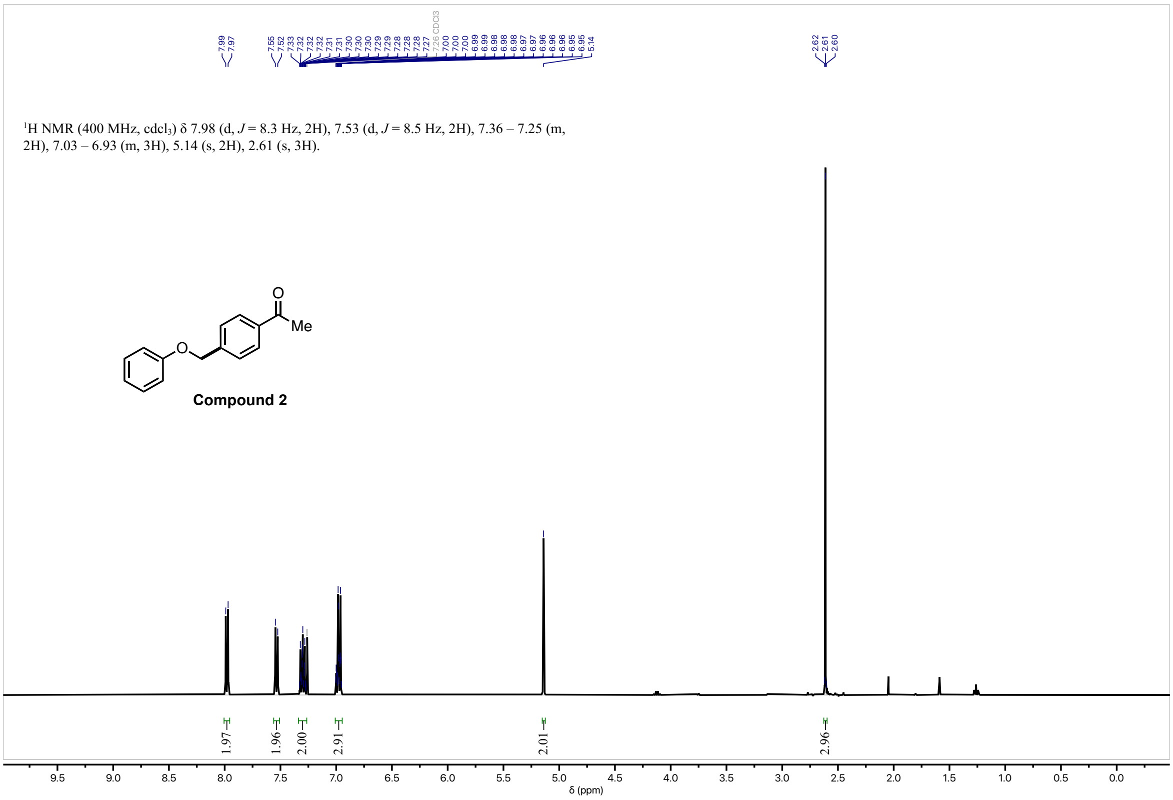

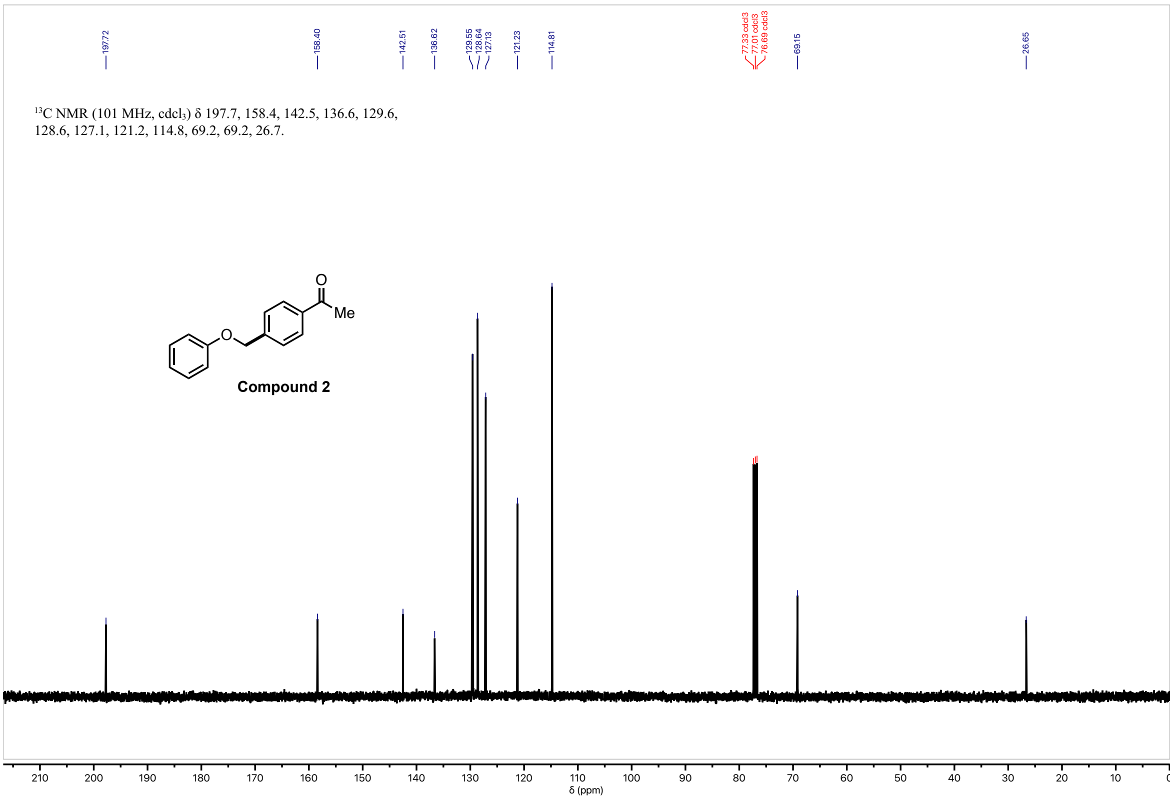

^{1}H NMR (400 MHz, cdcl$_3$) δ 7.86 – 7.78 (m, 4H), 7.63 – 7.53 (m, 3H), 7.53 – 7.45 (m, 2H), 7.35 – 7.28 (m, 2H), 7.02 – 6.96 (m, 3H), 5.17 (s, 2H).

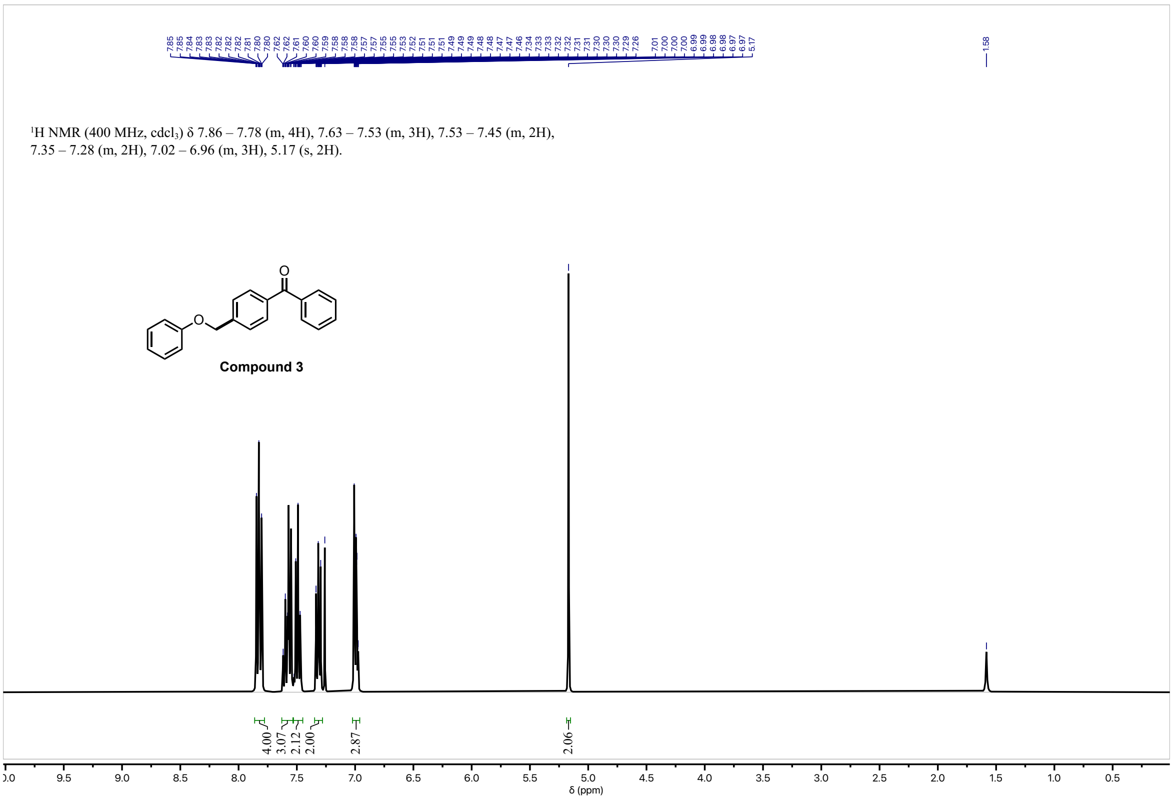

^{13}C NMR (101 MHz, cdcl$_3$) δ 196.5, 158.6, 142.0, 137.7, 137.2, 132.6, 130.6, 130.2, 129.7, 128.4, 127.1, 121.4, 115.0, 69.4.

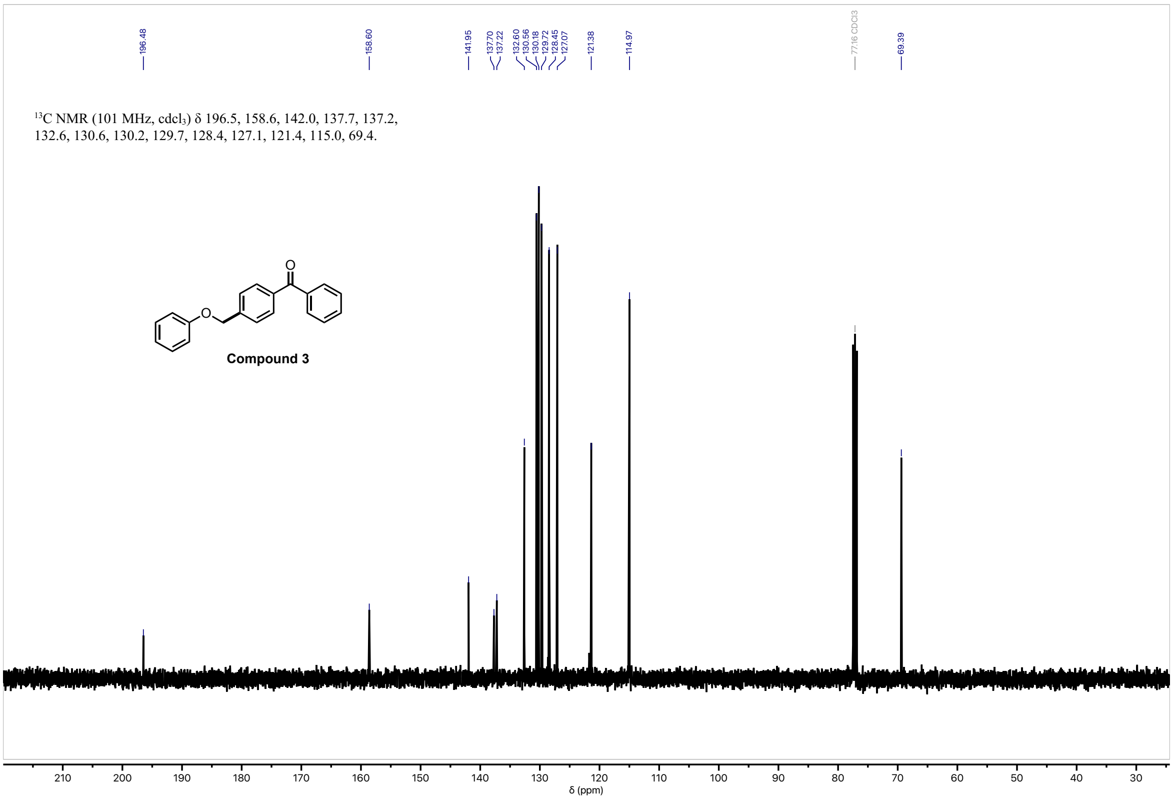

^{1}H NMR (400 MHz, cdcl$_3$) δ 7.65 (d, J = 8.0 Hz, 2H), 7.56 (d, J = 8.0 Hz, 2H), 7.34 – 7.28 (m, 2H), 7.02 – 6.94 (m, 3H), 5.14 (s, 2H).

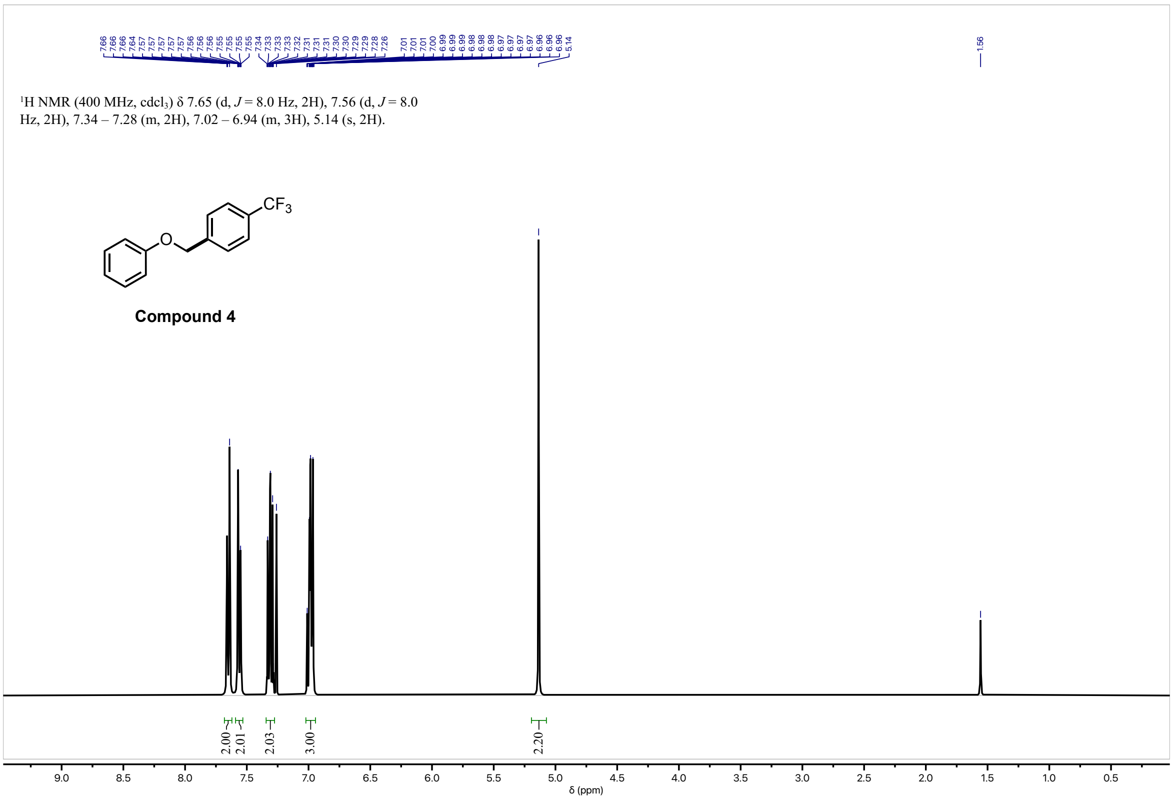

^{13}C NMR (151 MHz, CDCl$_3$) δ 158.5, 141.3, 130.3 (q, $J_{C,F}$ = 32.7 Hz), 129.7, 127.5, 125.7 (q, $J_{C,F}$ = 3.8 Hz), 124.4 (q, $J_{C,F}$ = 272.4 Hz), 121.5, 115.0, 69.2.

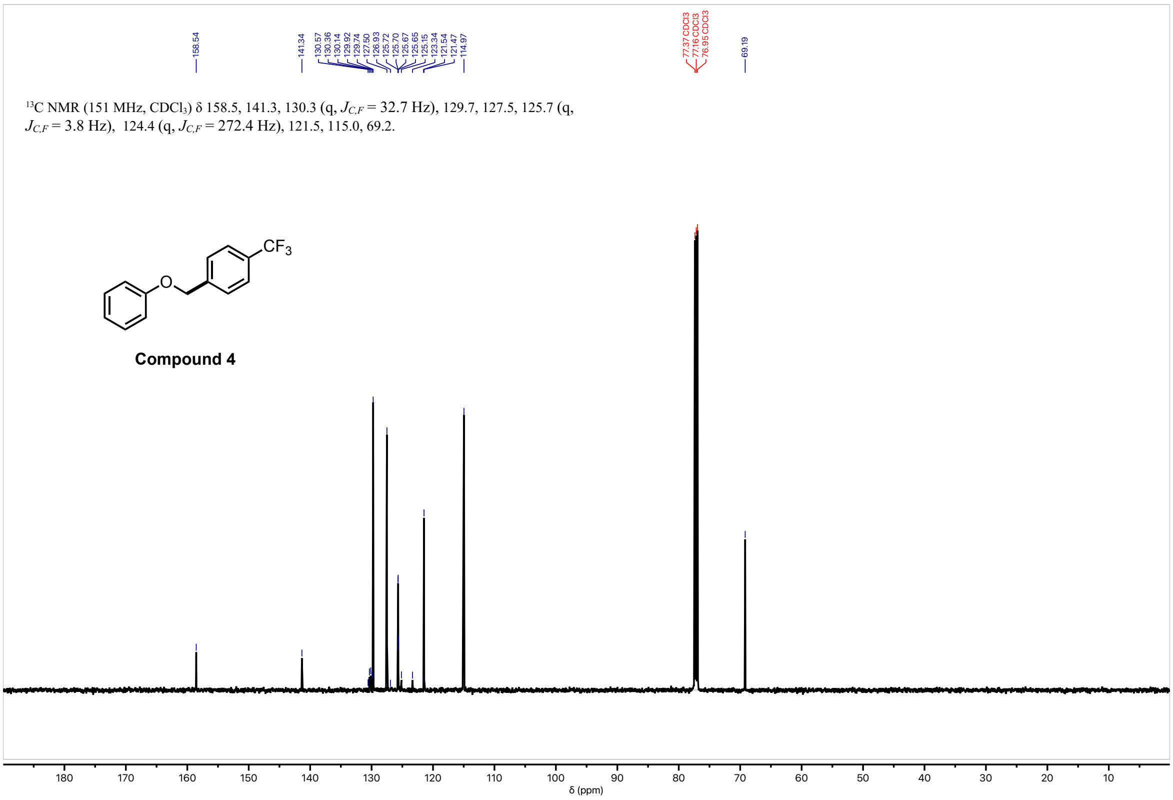

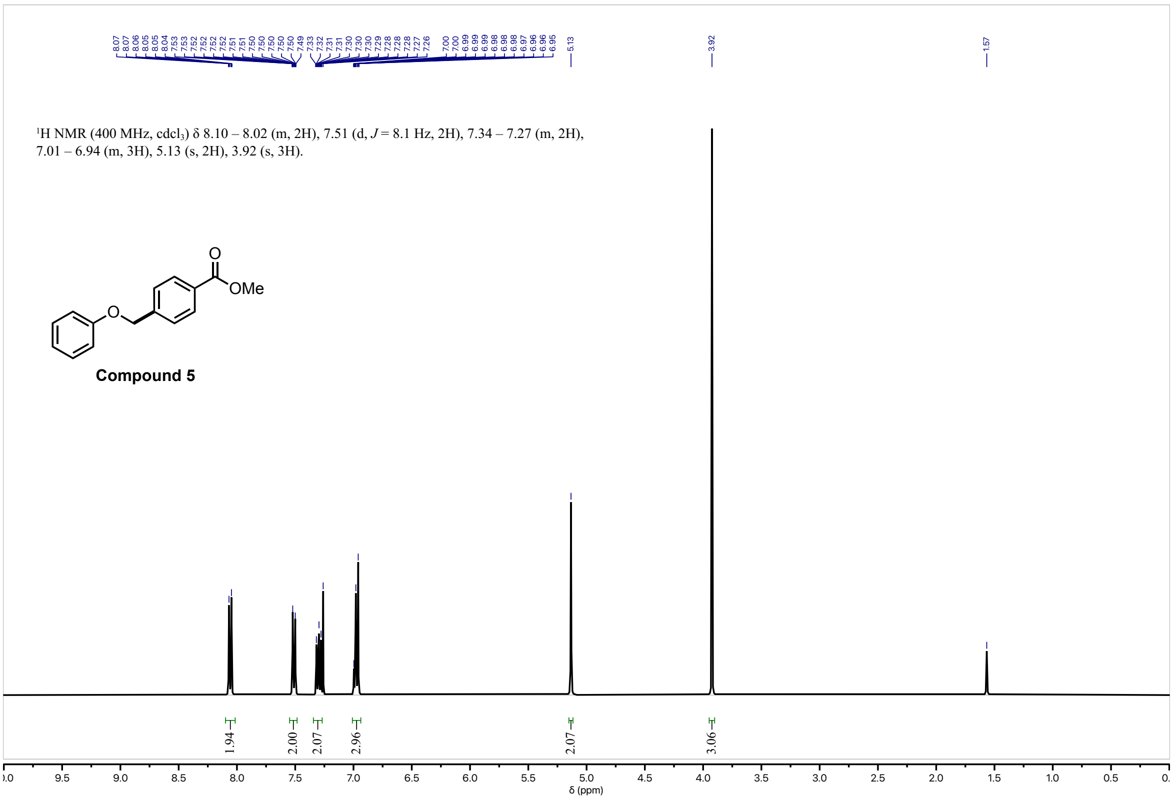
1H NMR (400 MHz, cdcl3) δ 8.10 – 8.02 (m, 2H), 7.51 (d, J = 8.1 Hz, 2H), 7.34 – 7.27 (m, 2H), 7.01 – 6.94 (m, 3H), 5.13 (s, 2H), 3.92 (s, 3H).
OMe
O
Compound 5
δ (ppm)

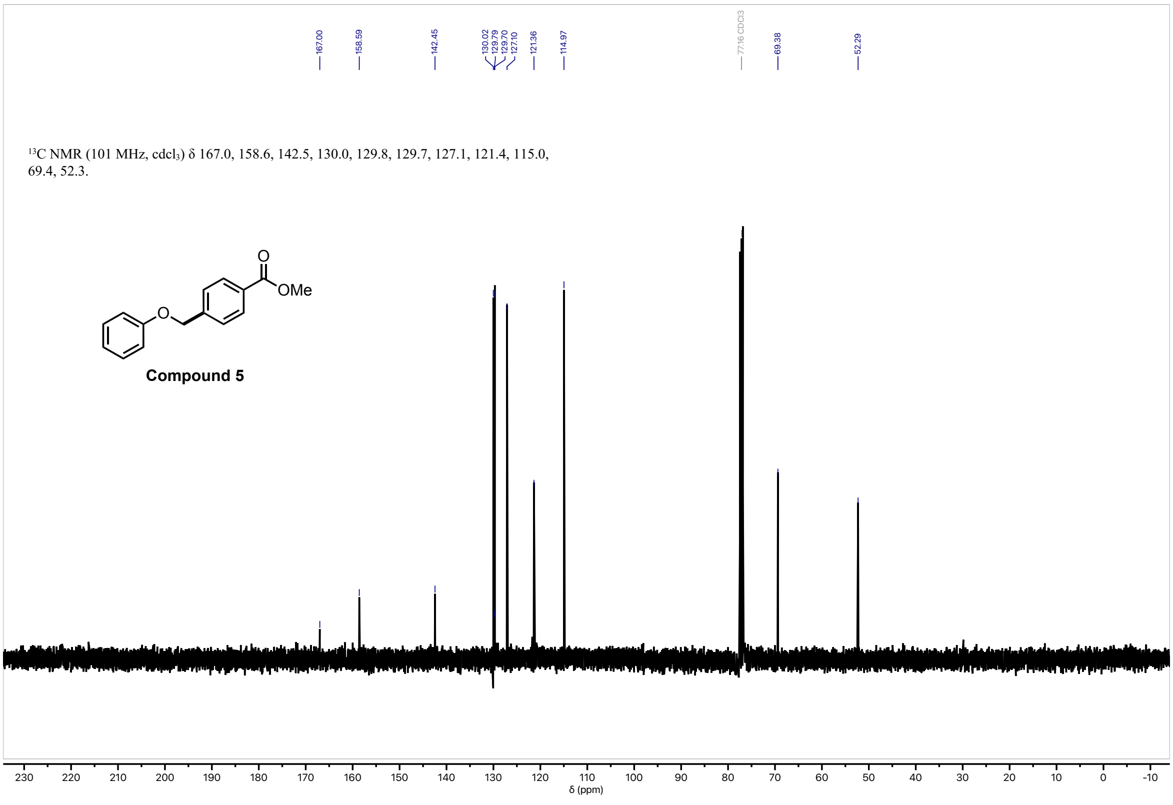
13C NMR (101 MHz, cdcl3) δ 167.0, 158.6, 142.5, 130.0, 129.8, 129.7, 127.1, 121.4, 115.0, 69.4, 52.3.
OMe
O
Compound 5
δ (ppm)

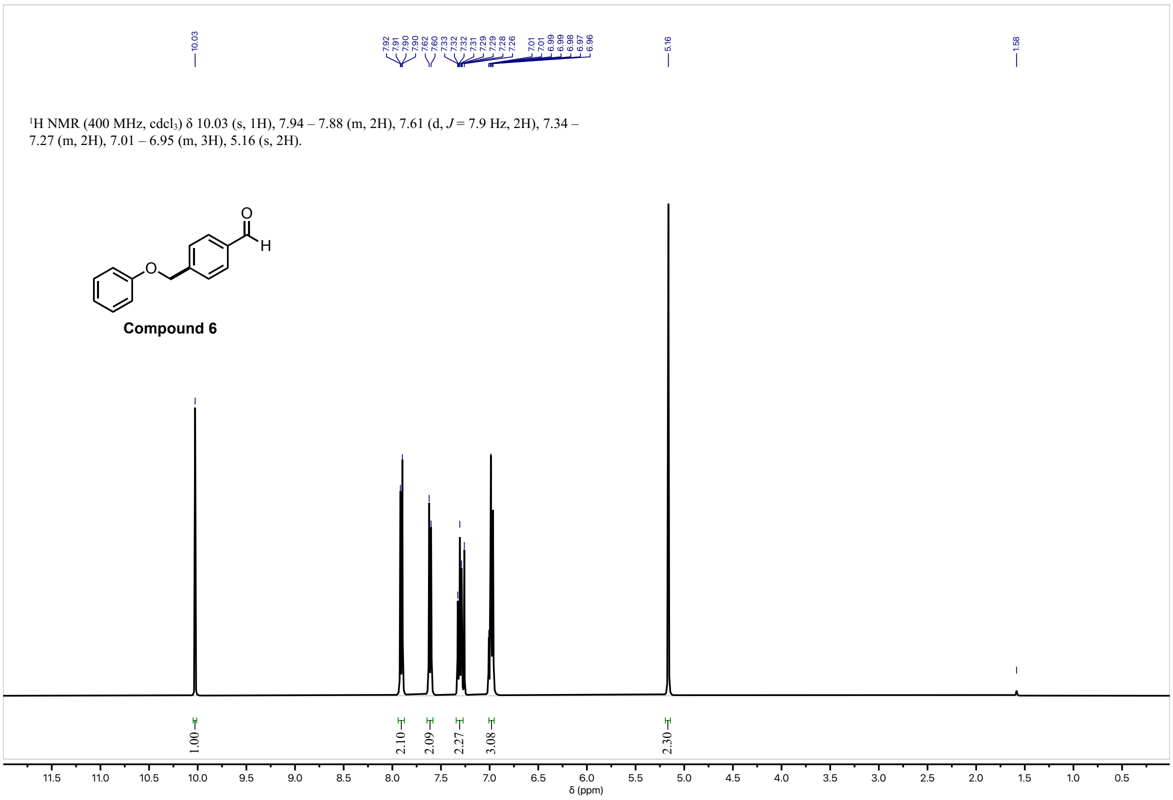
^{1}H NMR (400 MHz, cdcl$_3$) δ 10.03 (s, 1H), 7.94 – 7.88 (m, 2H), 7.61 (d, J = 7.9 Hz, 2H), 7.34 – 7.27 (m, 2H), 7.01 – 6.95 (m, 3H), 5.16 (s, 2H).
Compound 6
δ (ppm)

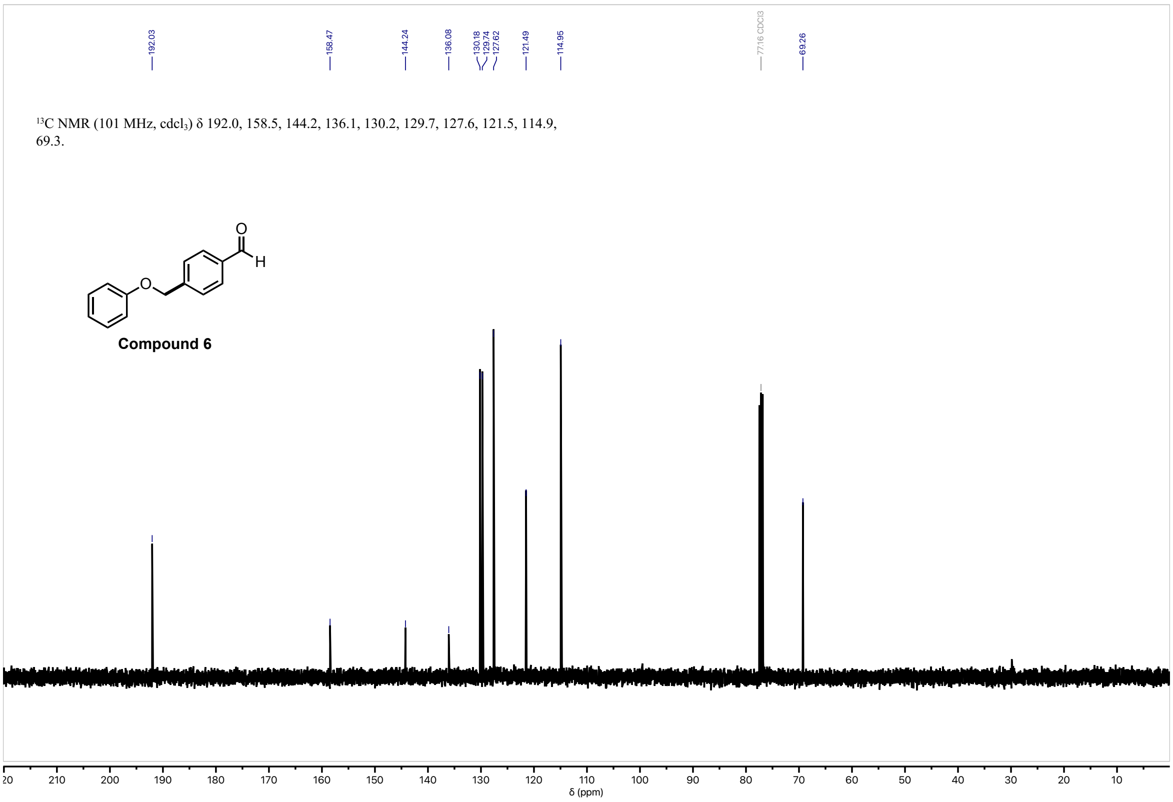
^{13}C NMR (101 MHz, cdcl$_3$) δ 192.0, 158.5, 144.2, 136.1, 130.2, 129.7, 127.6, 121.5, 114.9, 69.3.
Compound 6
δ (ppm)

¹H NMR (400 MHz, cdcl$_3$) δ 7.71 – 7.65 (m, 2H), 7.58 – 7.52 (m, 2H), 7.35 – 7.27 (m, 2H), 7.03 – 6.92 (m, 3H), 5.13 (s, 2H).

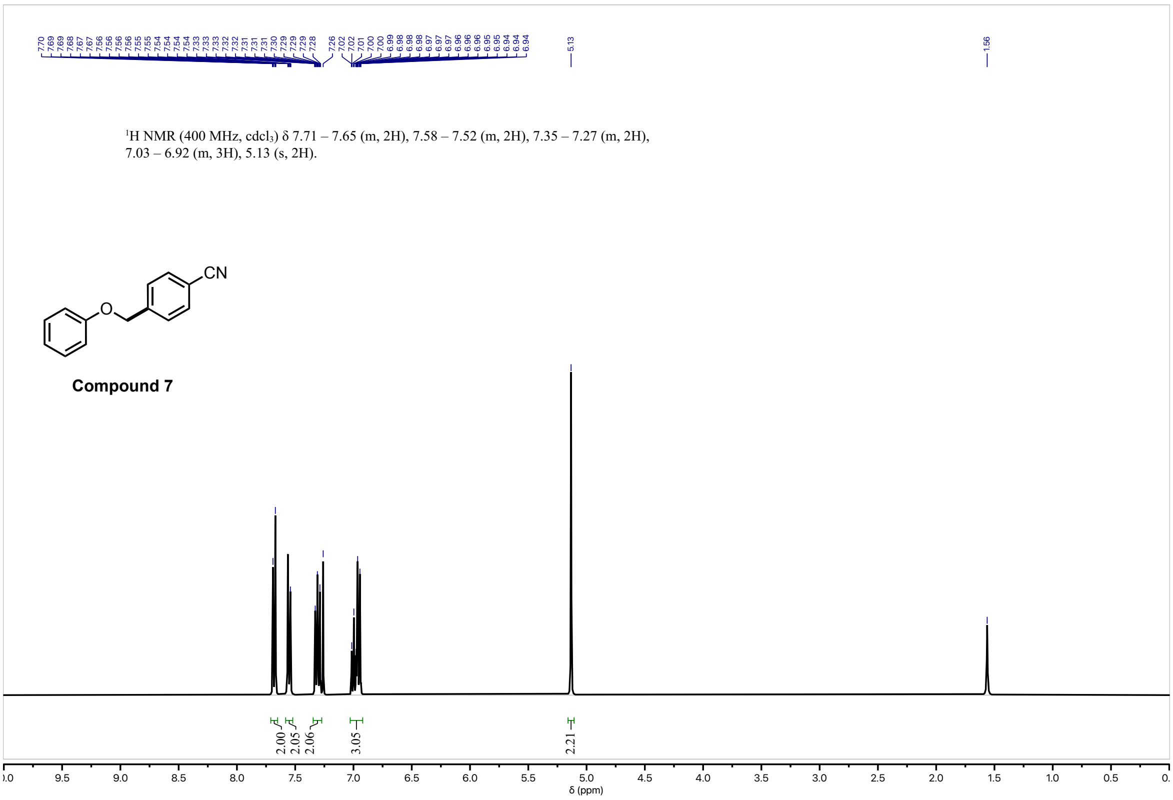

¹³C NMR (101 MHz, cdcl$_3$) δ 158.3, 142.7, 132.5, 129.8, 127.7, 121.6, 118.9, 114.9, 111.8, 68.9.

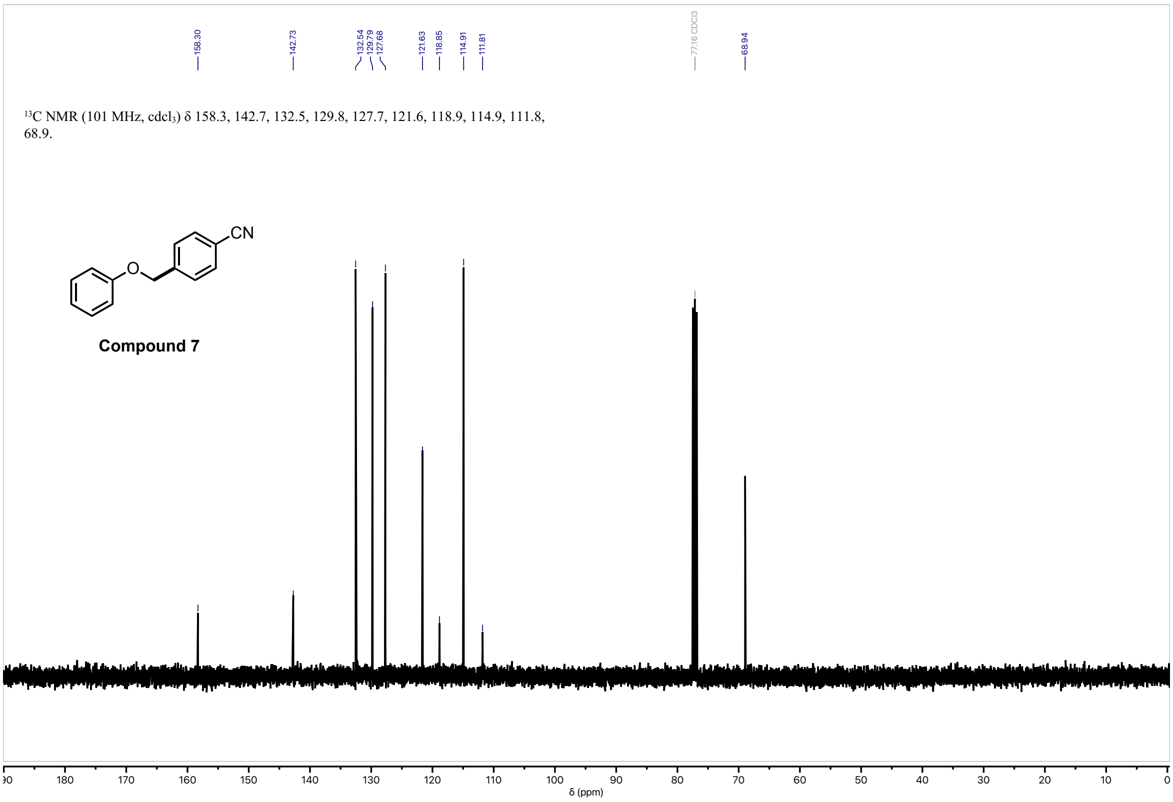

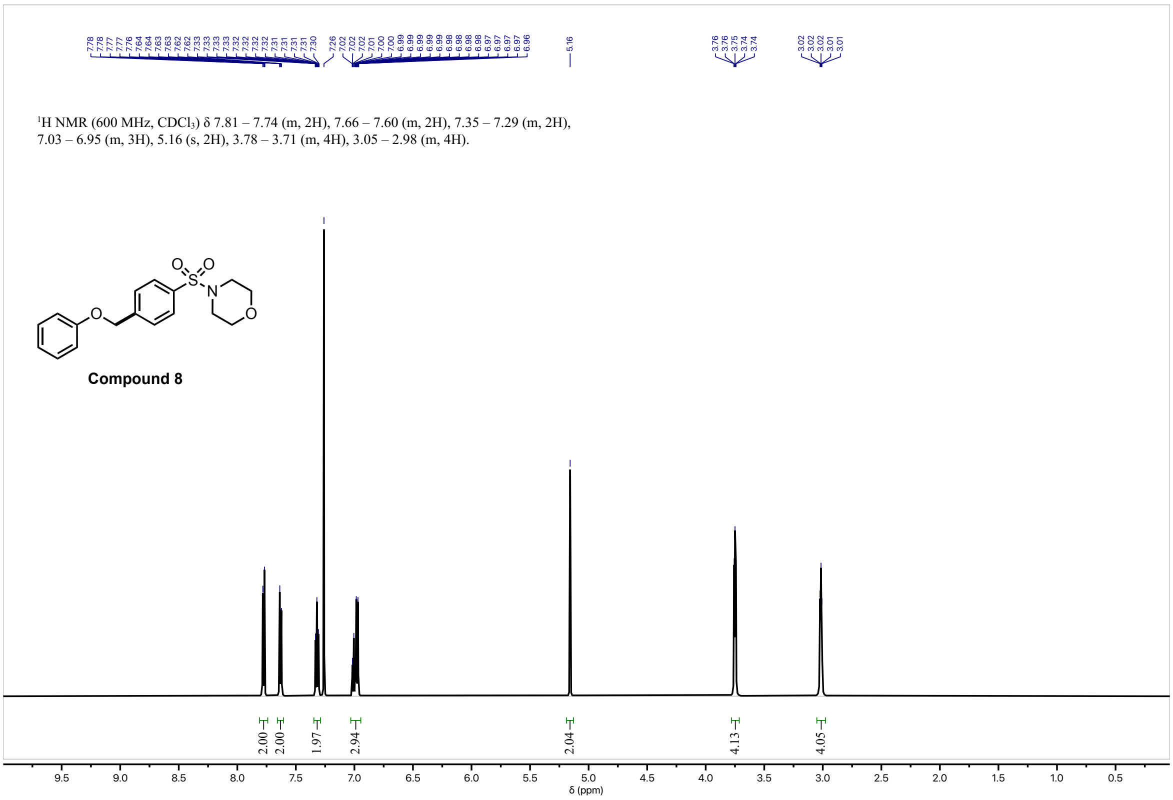
^{1}H NMR (600 MHz, CDCl3) δ 7.81 – 7.74 (m, 2H), 7.66 – 7.60 (m, 2H), 7.35 – 7.29 (m, 2H), 7.03 – 6.95 (m, 3H), 5.16 (s, 2H), 3.78 – 3.71 (m, 4H), 3.05 – 2.98 (m, 4H).
Compound 8
δ (ppm)

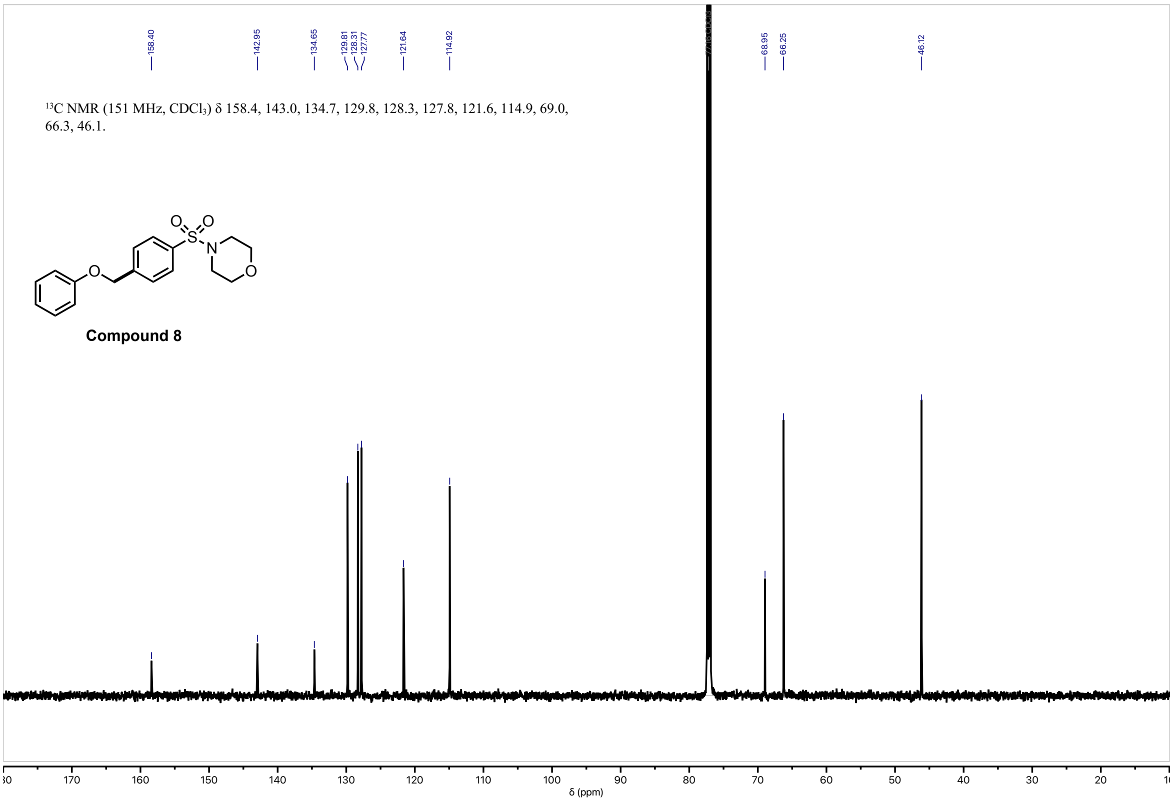
^{13}C NMR (151 MHz, CDCl3) δ 158.4, 143.0, 134.7, 129.8, 128.3, 127.8, 121.6, 114.9, 69.0, 66.3, 46.1.
Compound 8
δ (ppm)

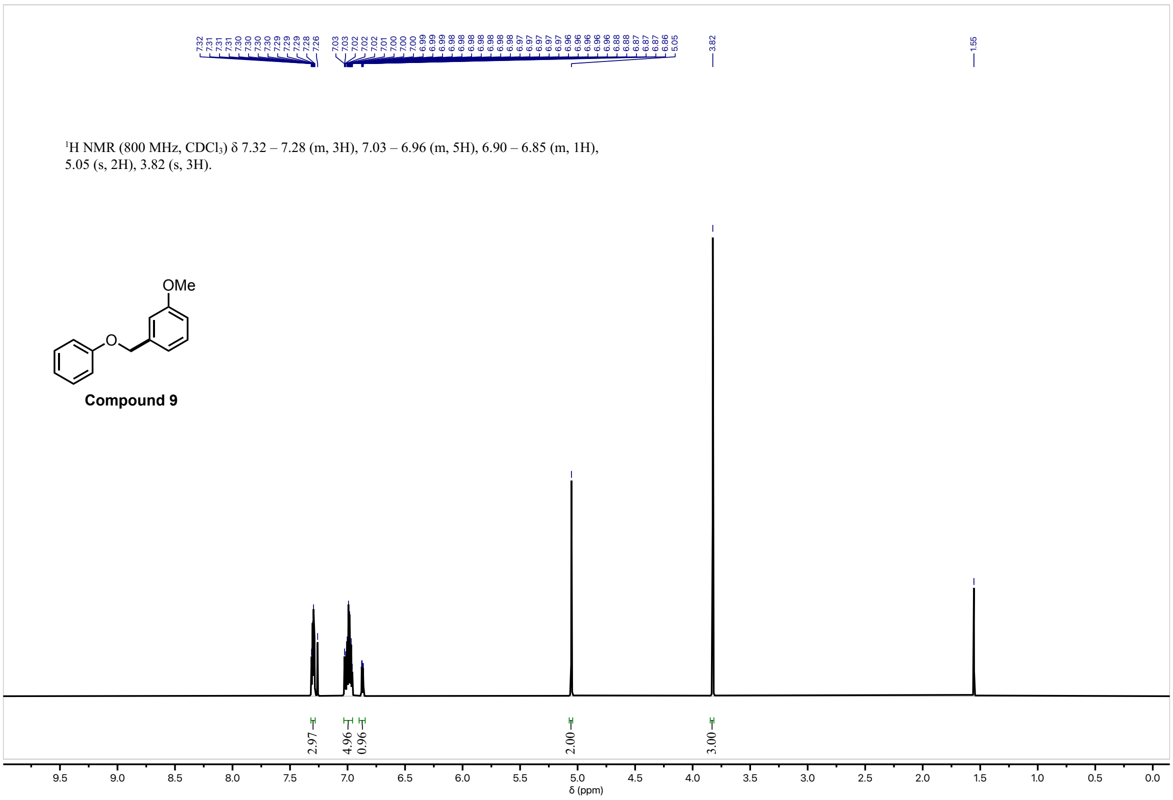

^{1}H NMR (800 MHz, $CDCl_3$) δ 7.32 – 7.28 (m, 3H), 7.03 – 6.96 (m, 5H), 6.90 – 6.85 (m, 1H), 5.05 (s, 2H), 3.82 (s, 3H).

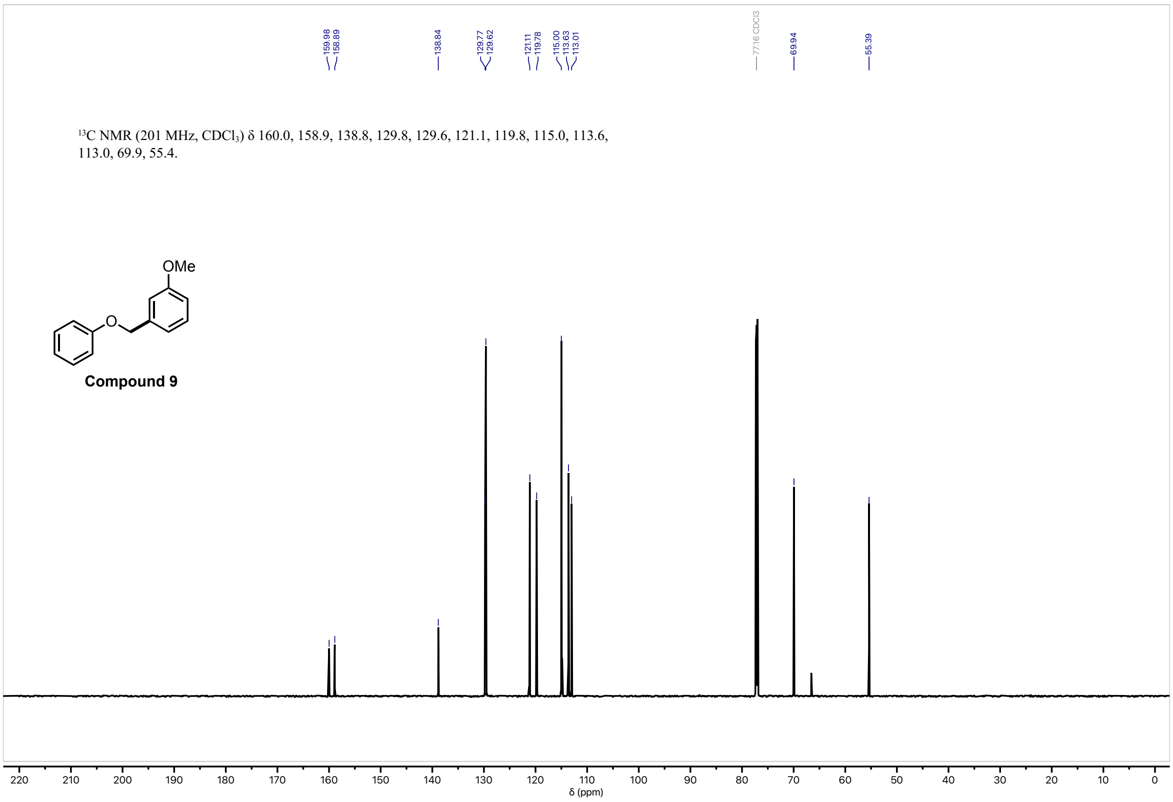

^{13}C NMR (201 MHz, $CDCl_3$) δ 160.0, 158.9, 138.8, 129.8, 129.6, 121.1, 119.8, 115.0, 113.6, 113.0, 69.9, 55.4.

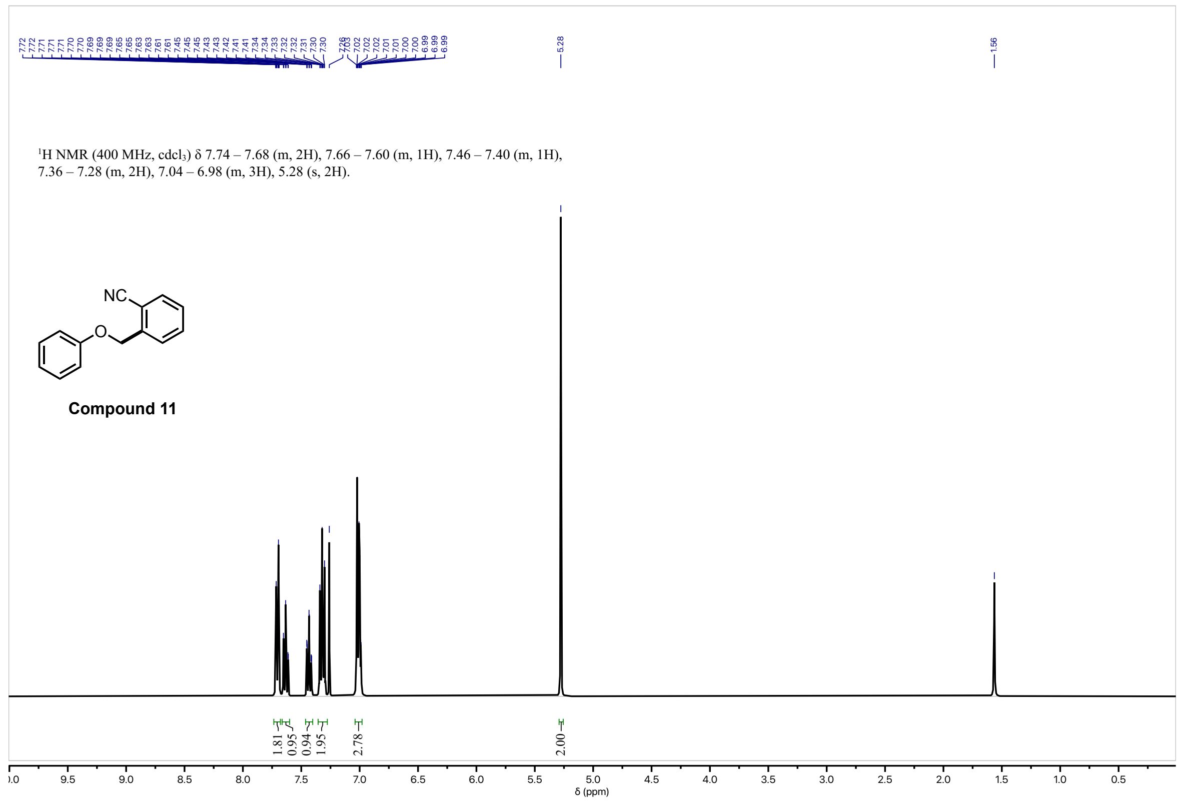
¹H NMR (400 MHz, cdcl₃) δ 7.74 – 7.68 (m, 2H), 7.66 – 7.60 (m, 1H), 7.46 – 7.40 (m, 1H), 7.36 – 7.28 (m, 2H), 7.04 – 6.98 (m, 3H), 5.28 (s, 2H).
NC
O
Compound 11
1.81
0.95
0.94
1.95
2.78
2.00
δ (ppm)

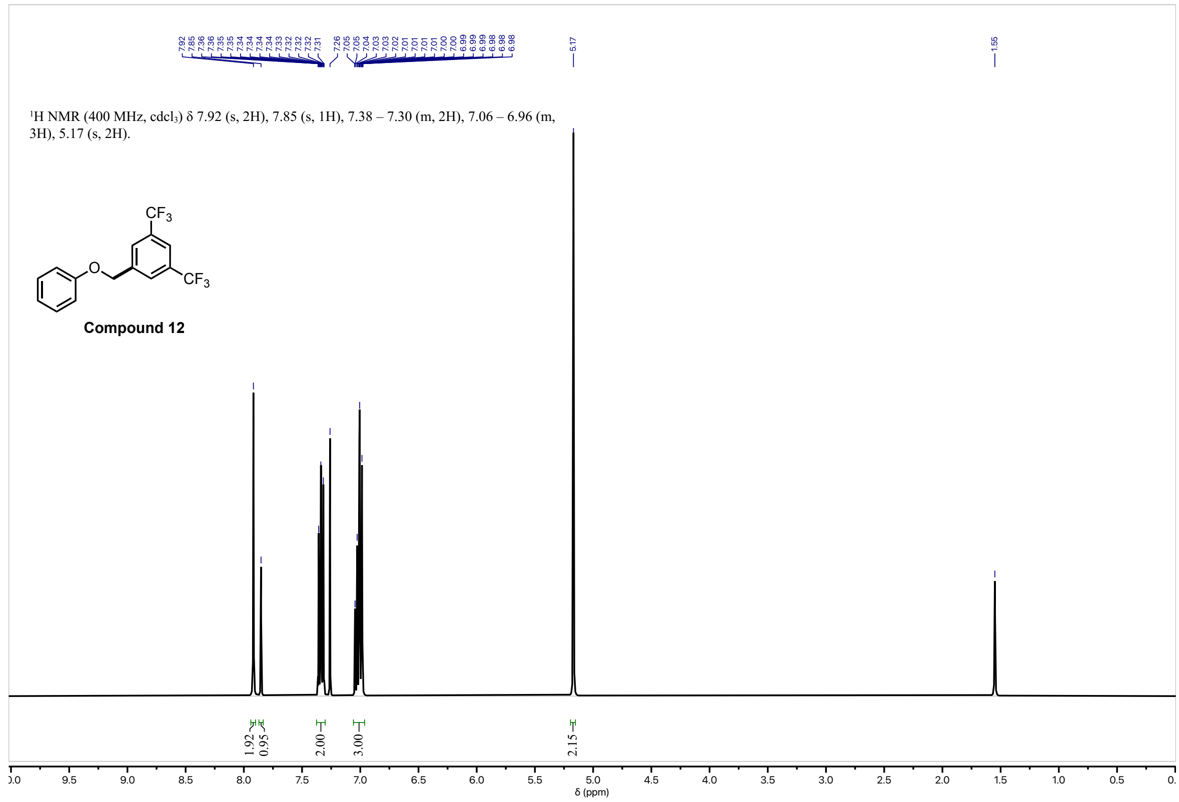
¹H NMR (400 MHz, cdcl₃) δ 7.92 (s, 2H), 7.85 (s, 1H), 7.38 – 7.30 (m, 2H), 7.06 – 6.96 (m, 3H), 5.17 (s, 2H).
CF3
O
CF3
Compound 12
1.92
0.95
2.00
3.00
2.15
δ (ppm)

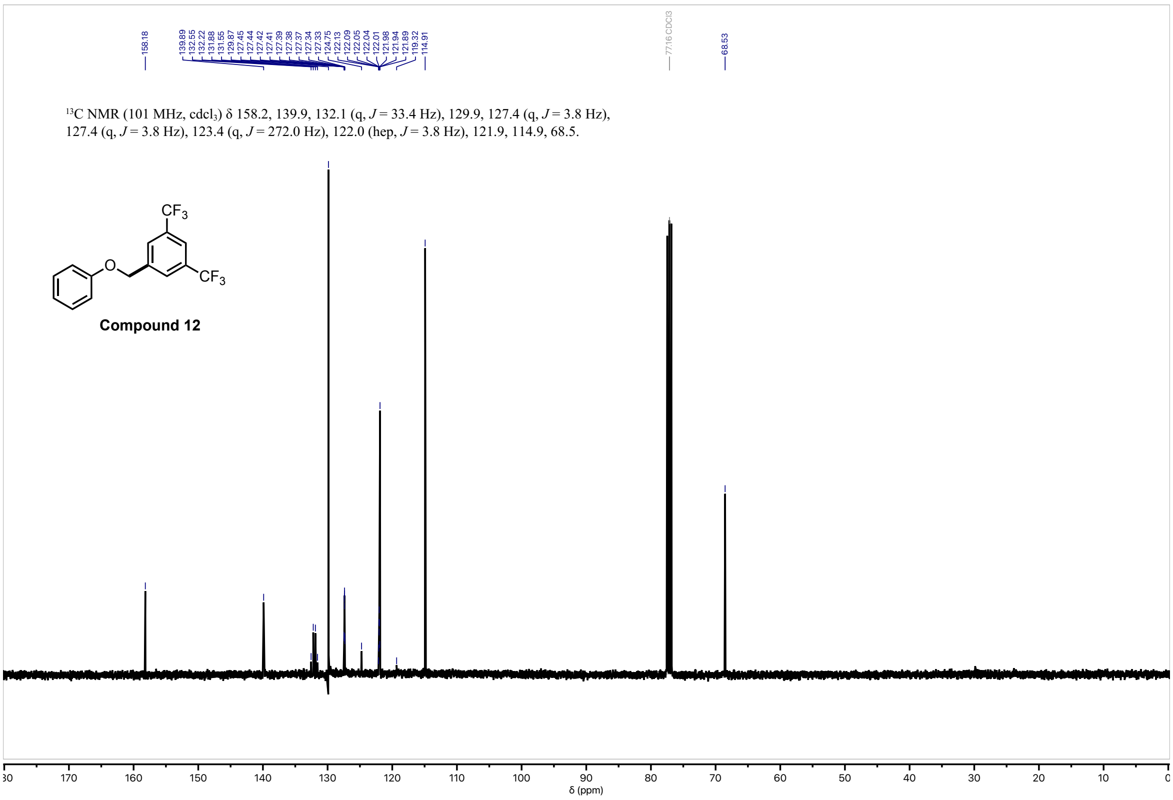
^{13}C NMR (101 MHz, cdcl3) δ 158.2, 139.9, 132.1 (q, J = 33.4 Hz), 129.9, 127.4 (q, J = 3.8 Hz), 127.4 (q, J = 3.8 Hz), 123.4 (q, J = 272.0 Hz), 122.0 (hep, J = 3.8 Hz), 121.9, 114.9, 68.5.
CF3
O
CF3
Compound 12
δ (ppm)

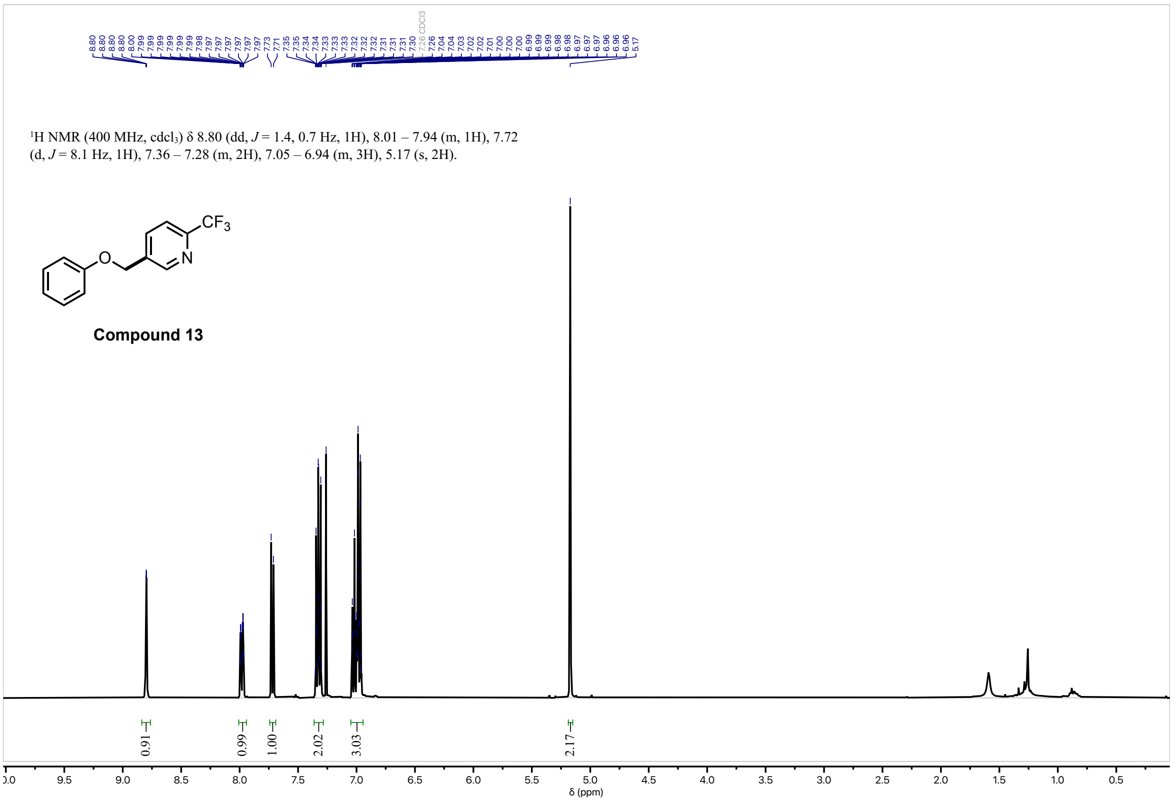
^{1}H NMR (400 MHz, cdcl3) δ 8.80 (dd, J = 1.4, 0.7 Hz, 1H), 8.01 – 7.94 (m, 1H), 7.72 (d, J = 8.1 Hz, 1H), 7.36 – 7.28 (m, 2H), 7.05 – 6.94 (m, 3H), 5.17 (s, 2H).
CF3
O
N
Compound 13
0.91
0.99
1.00
2.02
3.03
2.17
δ (ppm)

^{13}C NMR (101 MHz, cdcl$_3$) δ 158.1, 149.0, 148.0 (q, J = 34.9 Hz), 136.4, 136.2, 129.9, 121.9, 121.6 (q, J = 273.9 Hz), 120.5 (q, J = 2.7 Hz), 114.9, 66.8.

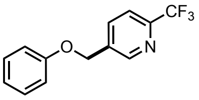

Compound 13

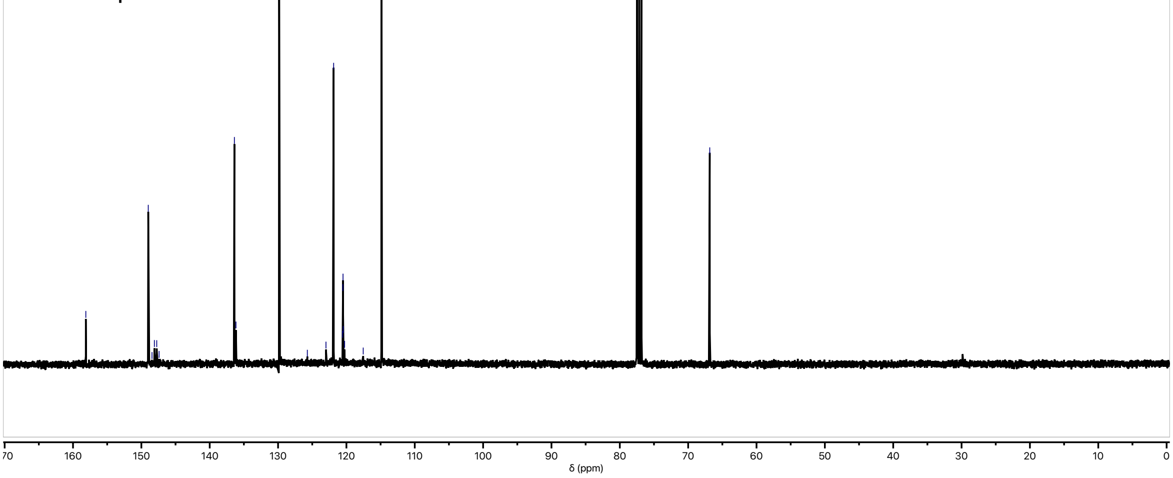

^{1}H NMR (800 MHz, CDCl$_3$) δ 8.21 (d, J = 5.2 Hz, 1H), 7.32 (t, J = 7.8 Hz, 2H), 7.23 (d, J = 5.2 Hz, 1H), 7.03 (s, 1H), 7.01 (t, J = 7.3 Hz, 1H), 6.95 (d, J = 8.1 Hz, 2H), 5.11 (s, 2H).

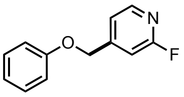

Compound 14

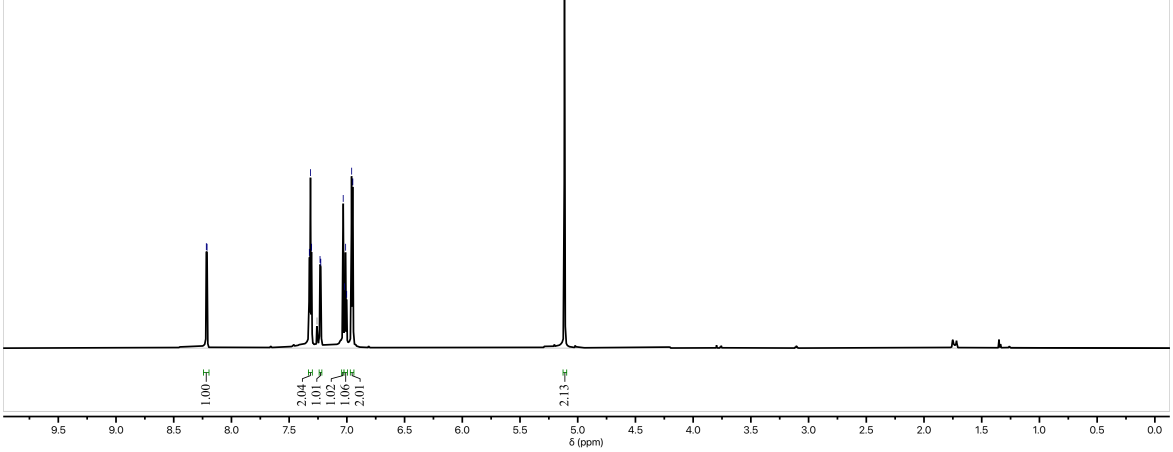

^{13}C NMR (201 MHz, $CDCl_3$) δ 164.3 (d, J = 238.7 Hz), 158.0, 152.5 (d, J = 7.7 Hz), 148.0 (d, J = 15.3 Hz), 129.8, 121.8, 119.1 (d, J = 4.1 Hz), 114.8, 107.3 (d, J = 38.7 Hz), 67.7 (d, J = 3.1 Hz).

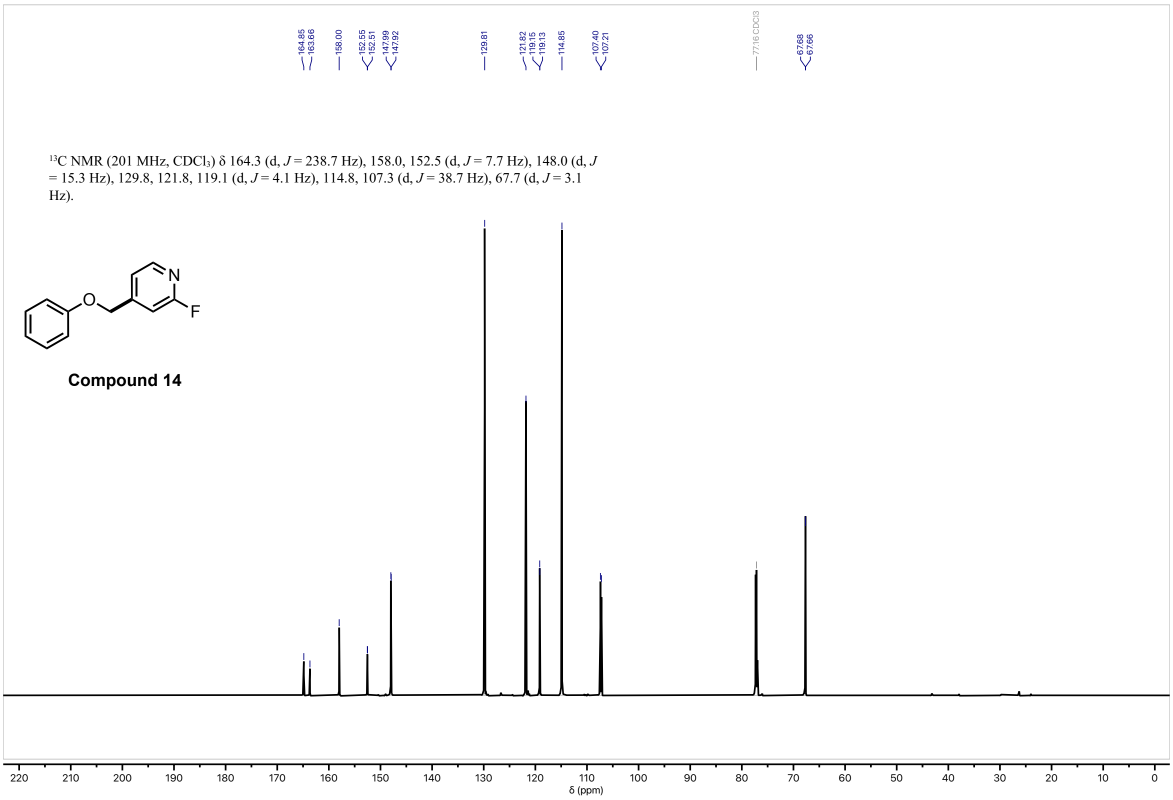

Compound 14

^{1}H NMR (600 MHz, $CDCl_3$) δ 8.99 (d, J = 2.2 Hz, 1H), 8.23 (dd, J = 2.3, 1.1 Hz, 1H), 8.15 – 8.11 (m, 1H), 7.87 – 7.83 (m, 1H), 7.75 – 7.71 (m, 1H), 7.59 – 7.55 (m, 1H), 7.35 – 7.30 (m, 2H), 7.05 – 6.98 (m, 3H), 5.27 (s, 2H).

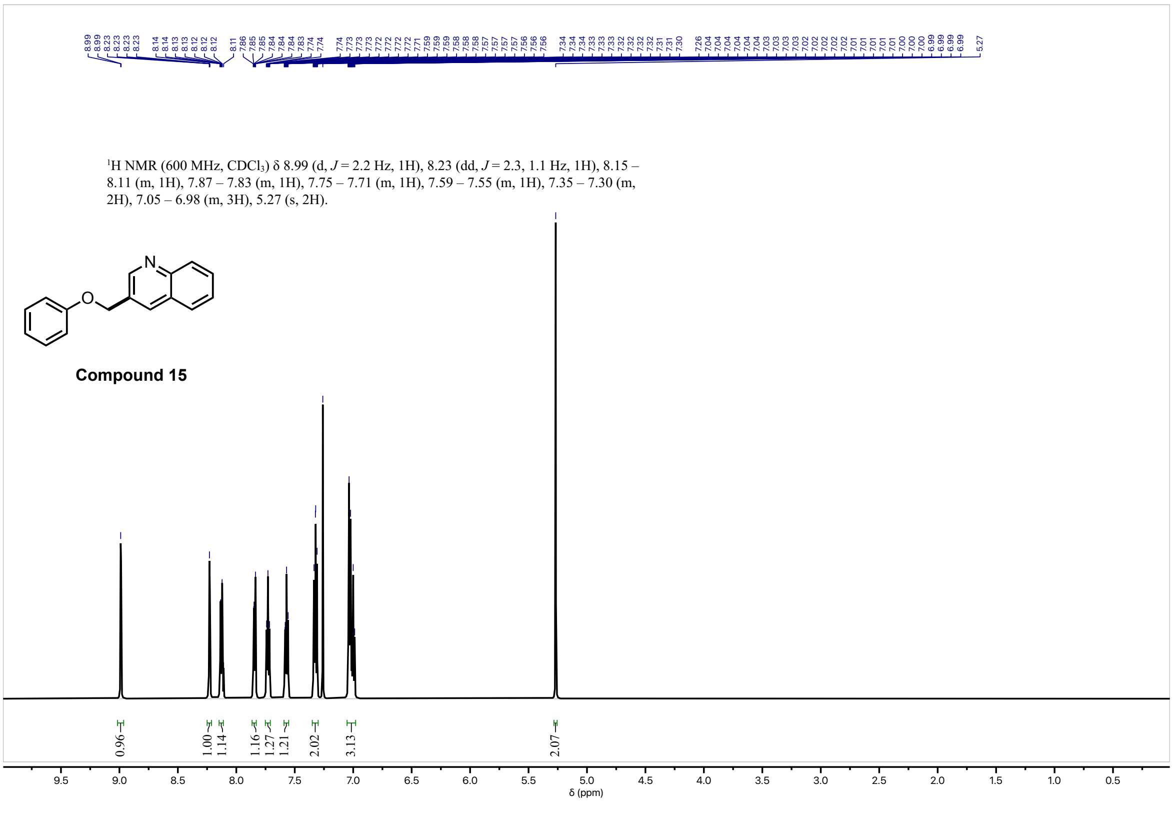

Compound 15

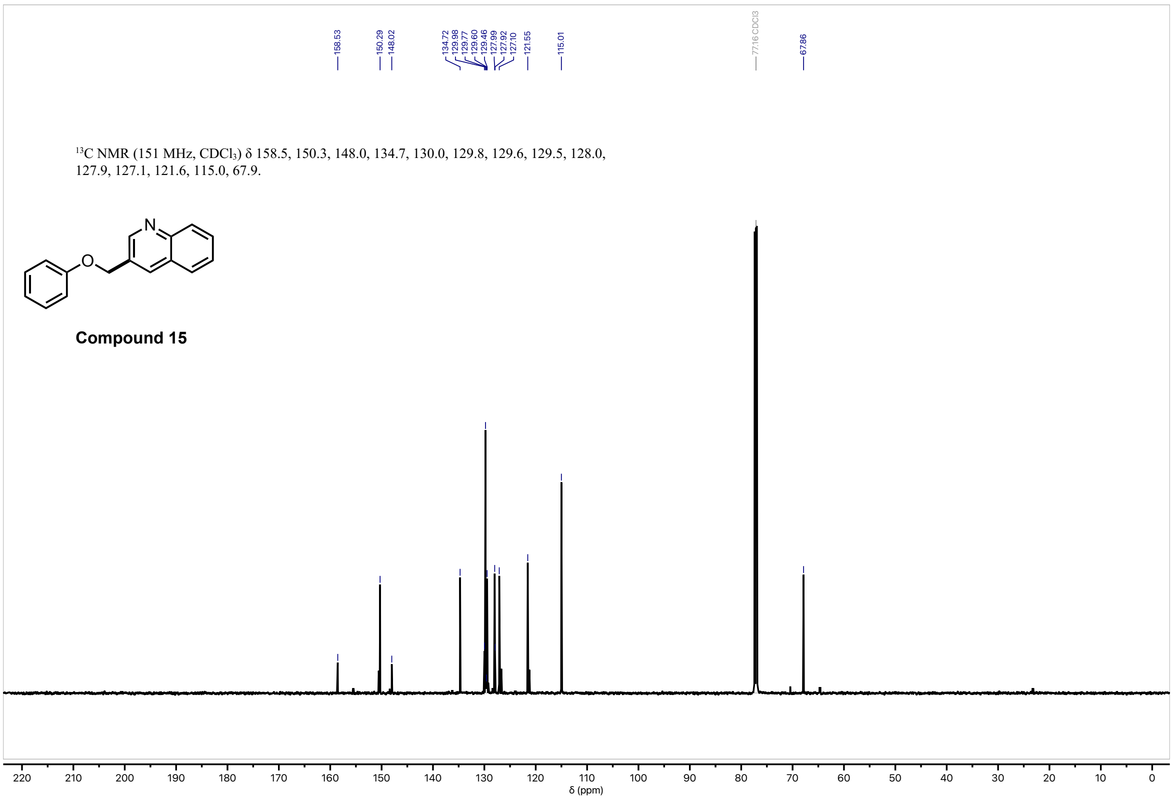

^{13}C NMR (151 MHz, $CDCl_3$) δ 158.5, 150.3, 148.0, 134.7, 130.0, 129.8, 129.6, 129.5, 128.0, 127.9, 127.1, 121.6, 115.0, 67.9.

Compound 15

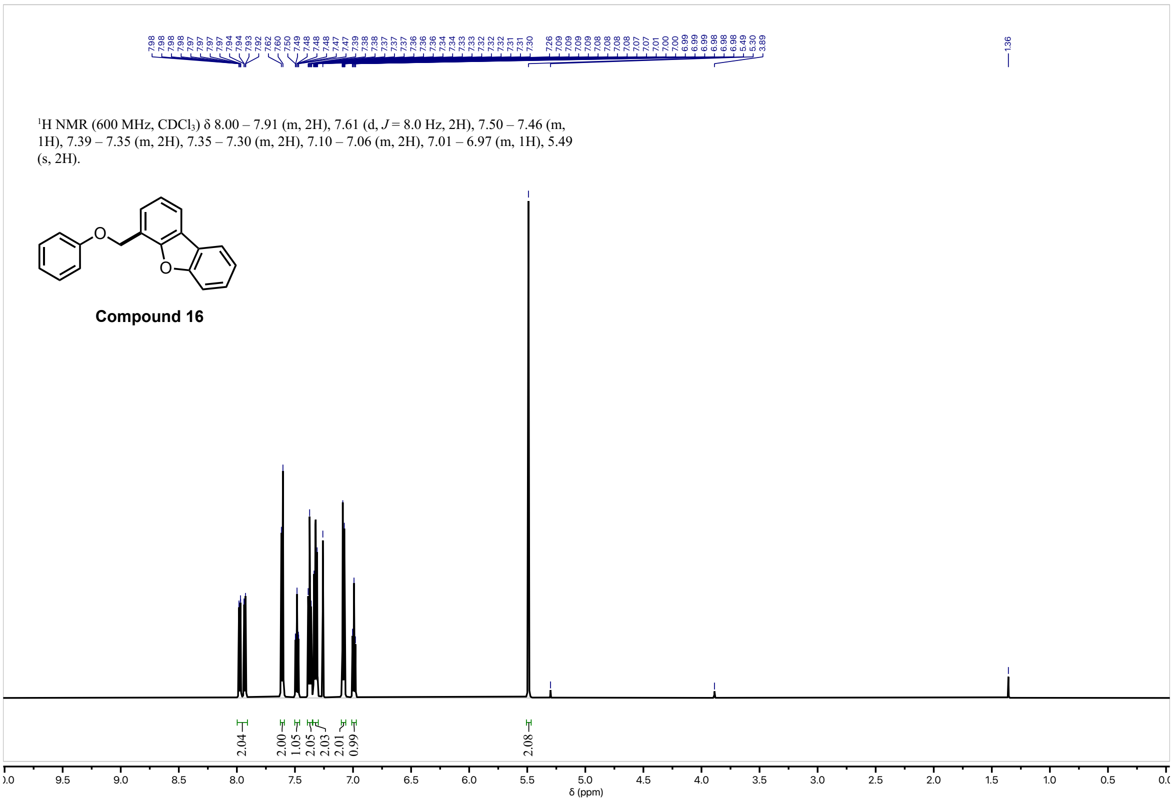

^{1}H NMR (600 MHz, $CDCl_3$) δ 8.00 – 7.91 (m, 2H), 7.61 (d, J = 8.0 Hz, 2H), 7.50 – 7.46 (m, 1H), 7.39 – 7.35 (m, 2H), 7.35 – 7.30 (m, 2H), 7.10 – 7.06 (m, 2H), 7.01 – 6.97 (m, 1H), 5.49 (s, 2H).

Compound 16

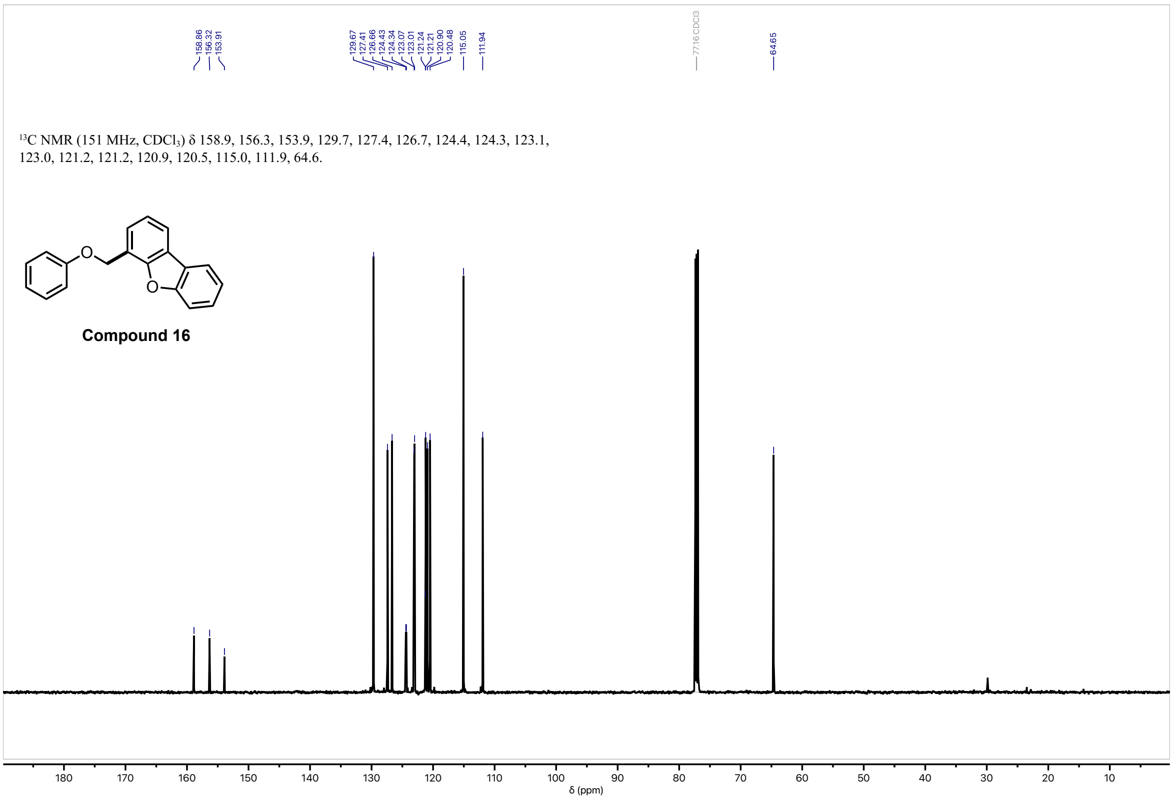

^{13}C NMR (151 MHz, $CDCl_3$) δ 158.9, 156.3, 153.9, 129.7, 127.4, 126.7, 124.4, 124.3, 123.1, 123.0, 121.2, 121.2, 120.9, 120.5, 115.0, 111.9, 64.6.

Compound 16

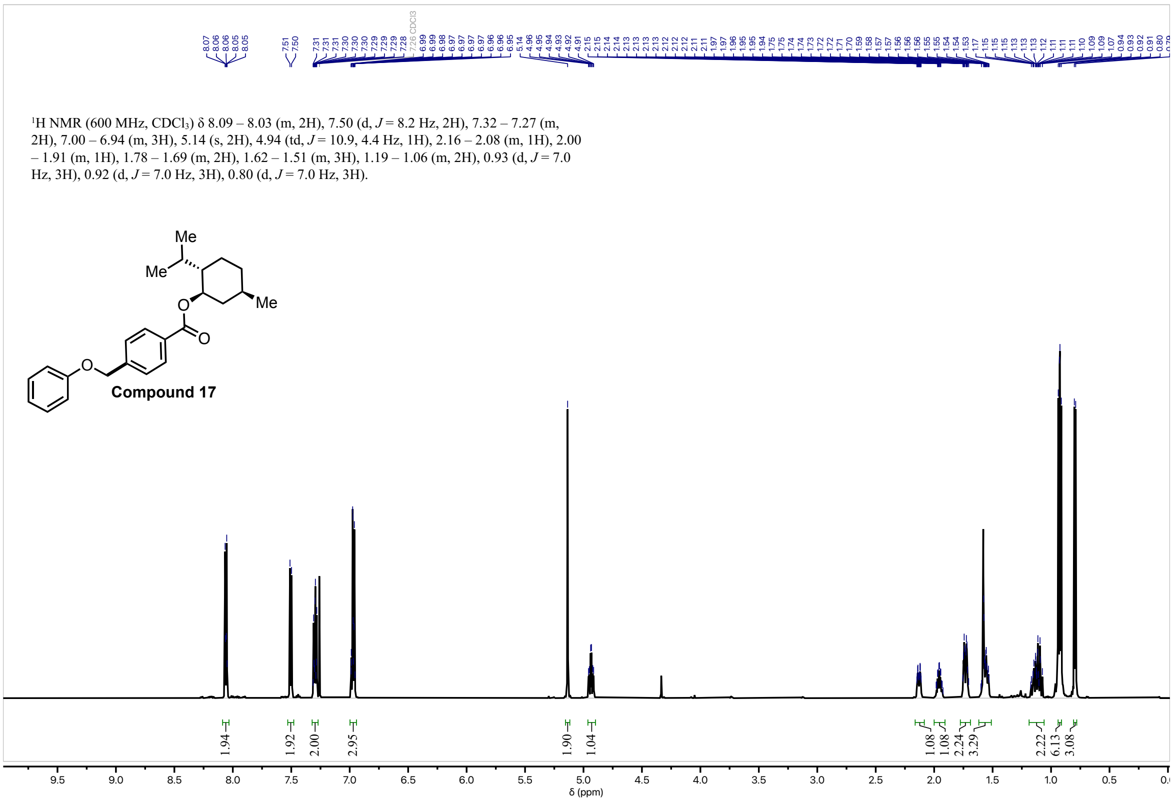

^{1}H NMR (600 MHz, $CDCl_3$) δ 8.09 – 8.03 (m, 2H), 7.50 (d, *J* = 8.2 Hz, 2H), 7.32 – 7.27 (m, 2H), 7.00 – 6.94 (m, 3H), 5.14 (s, 2H), 4.94 (td, *J* = 10.9, 4.4 Hz, 1H), 2.16 – 2.08 (m, 1H), 2.00 – 1.91 (m, 1H), 1.78 – 1.69 (m, 2H), 1.62 – 1.51 (m, 3H), 1.19 – 1.06 (m, 2H), 0.93 (d, *J* = 7.0 Hz, 3H), 0.92 (d, *J* = 7.0 Hz, 3H), 0.80 (d, *J* = 7.0 Hz, 3H).

Compound 17

^{13}C NMR (151 MHz, $CDCl_3$) δ 166.0, 158.6, 142.2, 130.5, 130.0, 129.7, 127.1, 121.3, 115.0, 75.0, 69.4, 47.4, 41.1, 34.5, 31.6, 26.7, 23.8, 22.2, 20.9, 16.7.

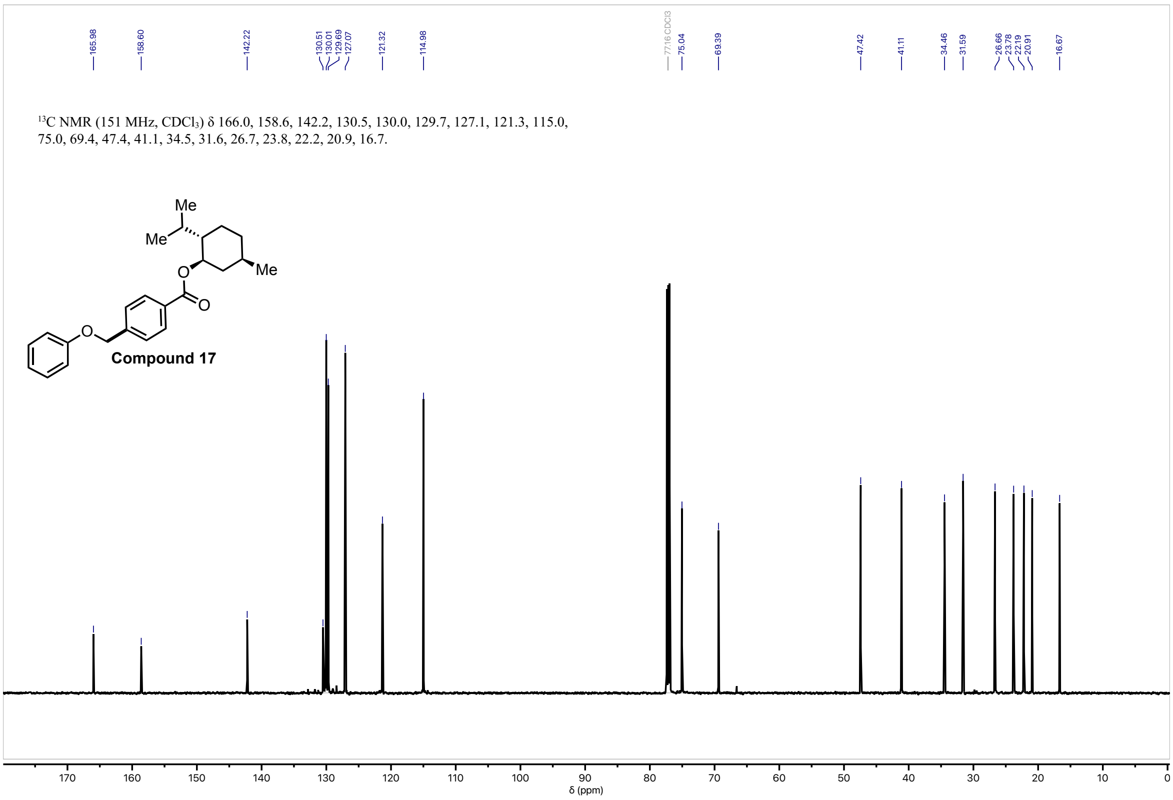

Compound 17

^{1}H NMR (600 MHz, $CDCl_3$) δ 8.11 – 8.02 (m, 2H), 7.50 (d, *J* = 8.1 Hz, 2H), 7.31 – 7.28 (m, 2H), 7.00 – 6.95 (m, 3H), 5.57 (d, *J* = 4.9 Hz, 1H), 5.13 (s, 2H), 4.65 (dd, *J* = 7.9, 2.4 Hz, 1H), 4.53 (dd, *J* = 11.5, 4.8 Hz, 1H), 4.43 (dd, *J* = 11.5, 7.6 Hz, 1H), 4.35 (dd, *J* = 5.0, 2.5 Hz, 1H), 4.33 (dd, *J* = 7.9, 1.9 Hz, 1H), 4.21 – 4.15 (m, 1H), 1.52 (s, 3H), 1.48 (s, 3H), 1.36 (s, 3H), 1.34 (s, 3H).

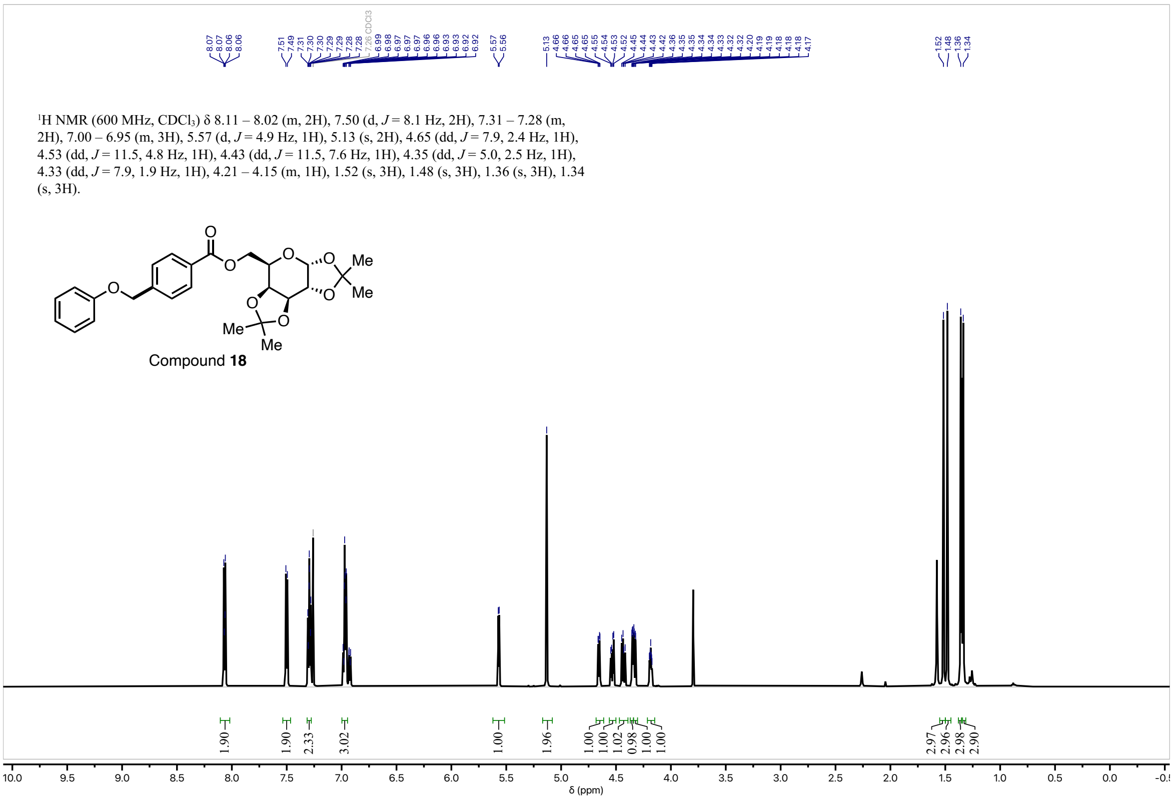

Compound **18**

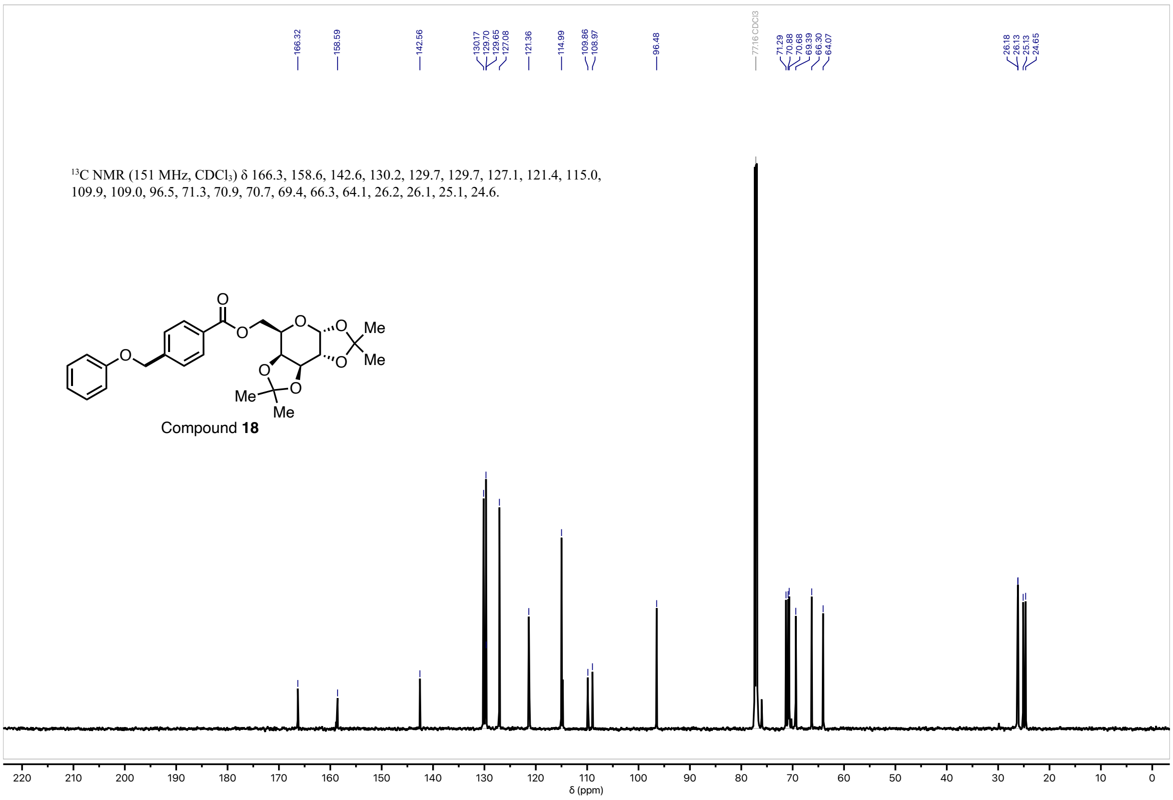

^{13}C NMR (151 MHz, $CDCl_3$) δ 166.3, 158.6, 142.6, 130.2, 129.7, 129.7, 127.1, 121.4, 115.0, 109.9, 109.0, 96.5, 71.3, 70.9, 70.7, 69.4, 66.3, 64.1, 26.2, 26.1, 25.1, 24.6.

Compound **18**

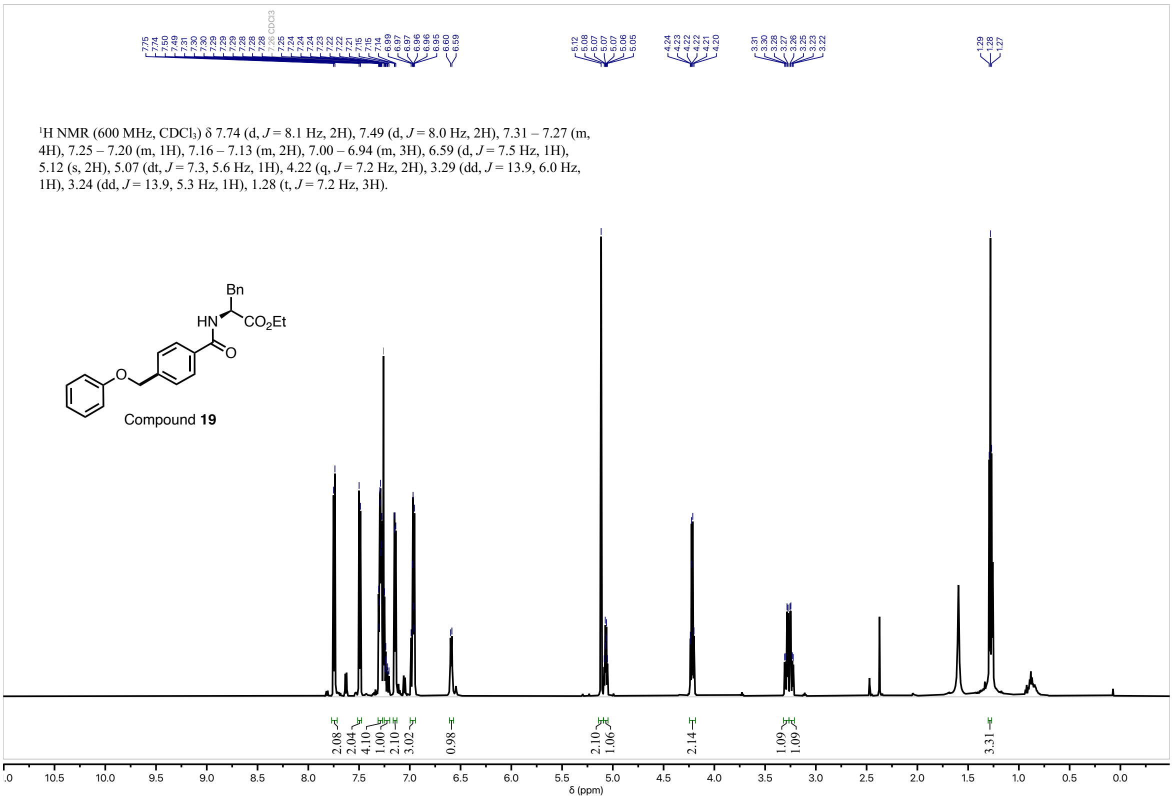

^{1}H NMR (600 MHz, $CDCl_3$) δ 7.74 (d, J = 8.1 Hz, 2H), 7.49 (d, J = 8.0 Hz, 2H), 7.31 – 7.27 (m, 4H), 7.25 – 7.20 (m, 1H), 7.16 – 7.13 (m, 2H), 7.00 – 6.94 (m, 3H), 6.59 (d, J = 7.5 Hz, 1H), 5.12 (s, 2H), 5.07 (dt, J = 7.3, 5.6 Hz, 1H), 4.22 (q, J = 7.2 Hz, 2H), 3.29 (dd, J = 13.9, 6.0 Hz, 1H), 3.24 (dd, J = 13.9, 5.3 Hz, 1H), 1.28 (t, J = 7.2 Hz, 3H).

Compound **19**

^{13}C NMR (151 MHz, $CDCl_3$) δ 171.7, 166.6, 158.6, 141.2, 136.0, 133.6, 129.7, 129.6, 128.7, 127.5, 127.4, 127.3, 121.3, 115.0, 69.3, 61.8, 53.7, 38.1, 14.3.

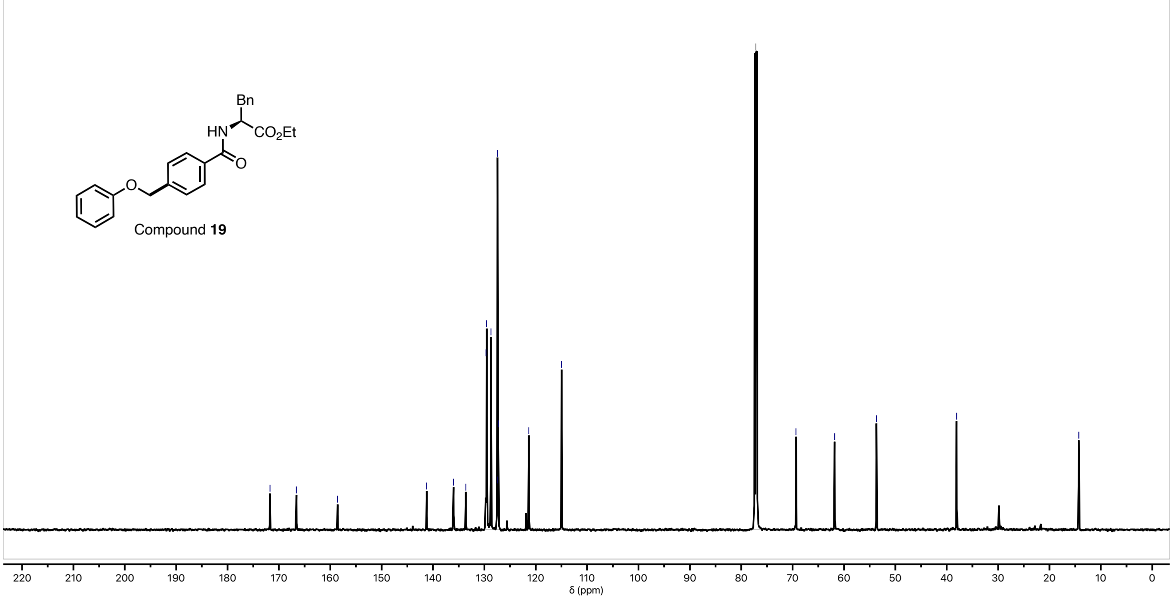

^{1}H NMR (600 MHz, $CDCl_3$) δ 7.90 (d, J = 8.1 Hz, 2H), 7.60 (d, J = 8.1 Hz, 2H), 7.34 – 7.28 (m, 2H), 7.02 – 6.95 (m, 3H), 5.14 (s, 2H), 4.34 (dd, J = 8.5, 3.8 Hz, 1H), 3.70 (s, 3H), 3.53 – 3.46 (m, 1H), 3.35 (dt, J = 9.0, 7.1 Hz, 1H), 2.06 – 1.95 (m, 3H), 1.84 – 1.75 (m, 1H).

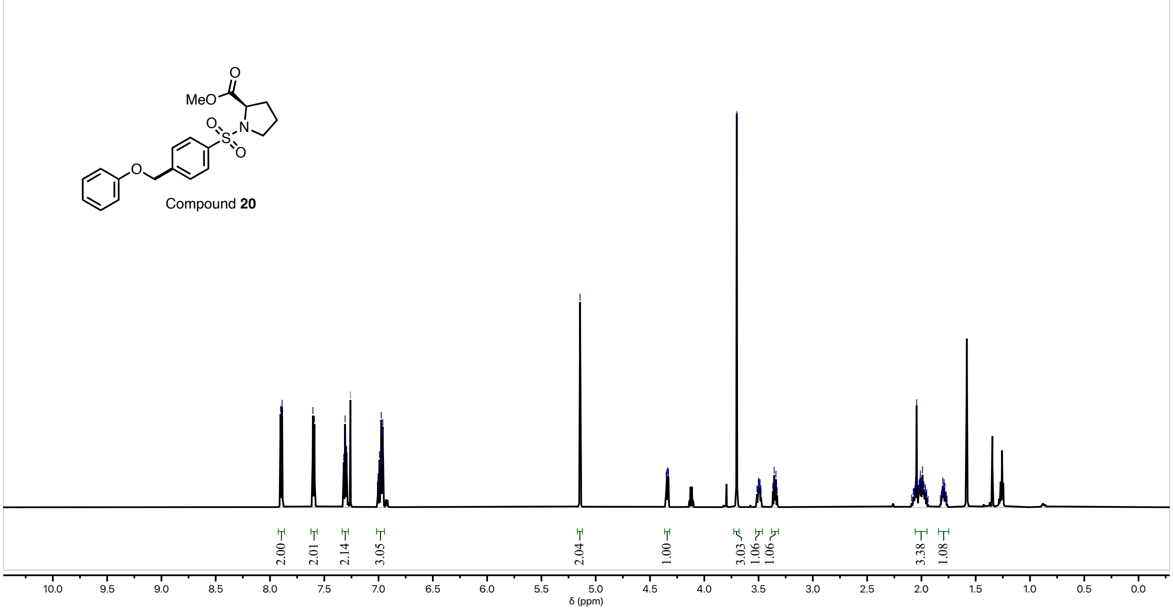

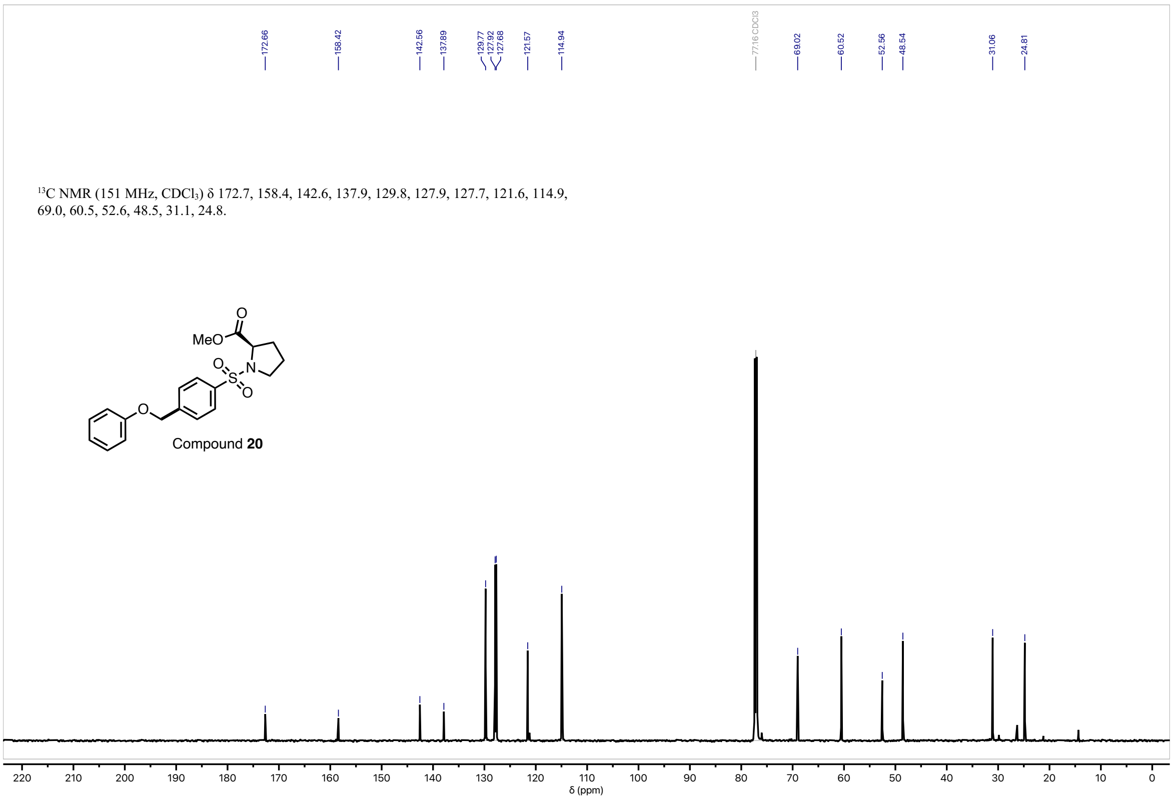

^{13}C NMR (151 MHz, $CDCl_3$) δ 172.7, 158.4, 142.6, 137.9, 129.8, 127.9, 127.7, 121.6, 114.9, 69.0, 60.5, 52.6, 48.5, 31.1, 24.8.

Compound **20**

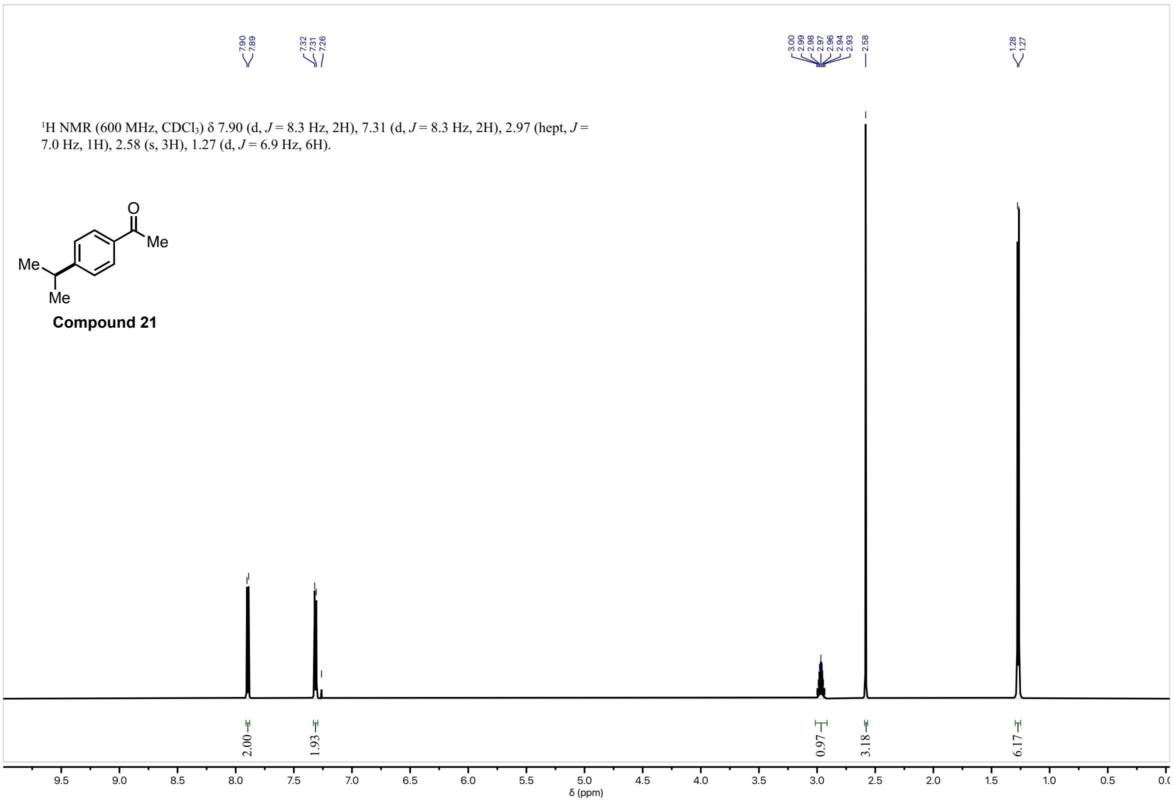

^{1}H NMR (600 MHz, $CDCl_3$) δ 7.90 (d, J = 8.3 Hz, 2H), 7.31 (d, J = 8.3 Hz, 2H), 2.97 (hept, J = 7.0 Hz, 1H), 2.58 (s, 3H), 1.27 (d, J = 6.9 Hz, 6H).

Compound 21

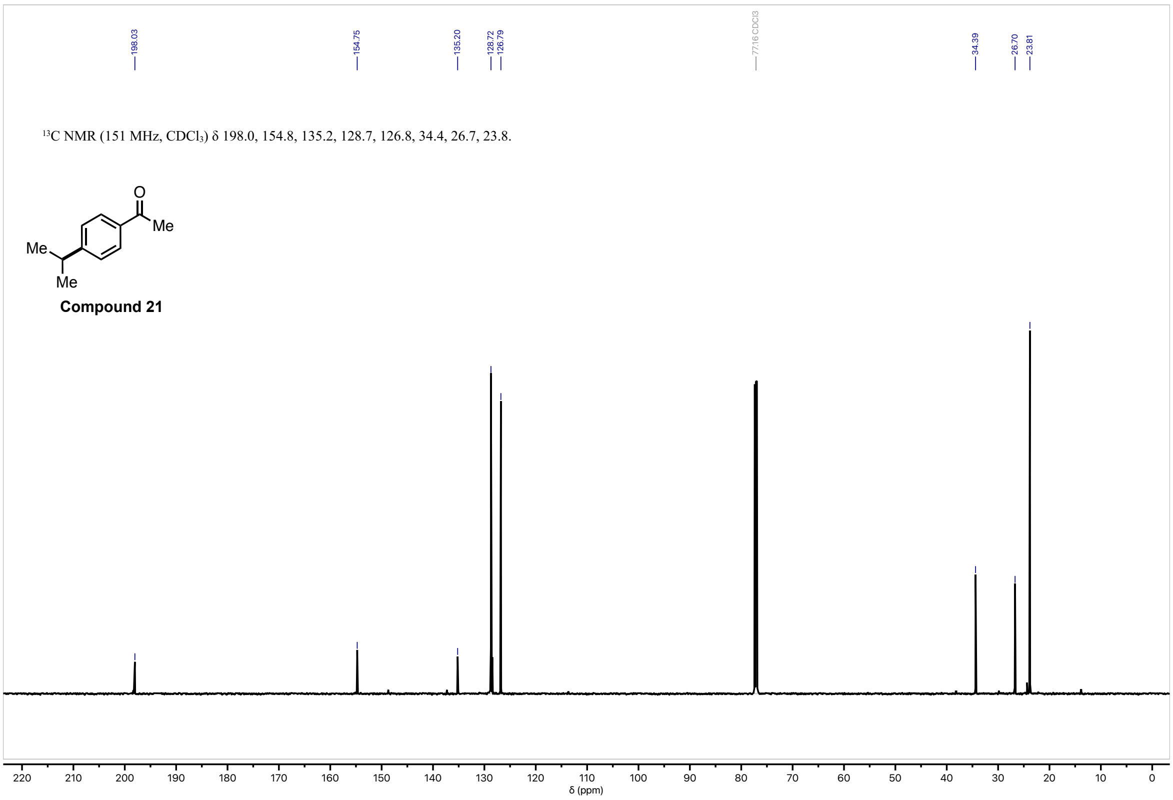
198.03
154.75
135.20
128.72
126.79
77.16 CDCl3
34.39
26.70
23.81
^{13}C NMR (151 MHz, $CDCl_3$) δ 198.0, 154.8, 135.2, 128.7, 126.8, 34.4, 26.7, 23.8.
O
Me
Me
Me
Compound 21
δ (ppm)

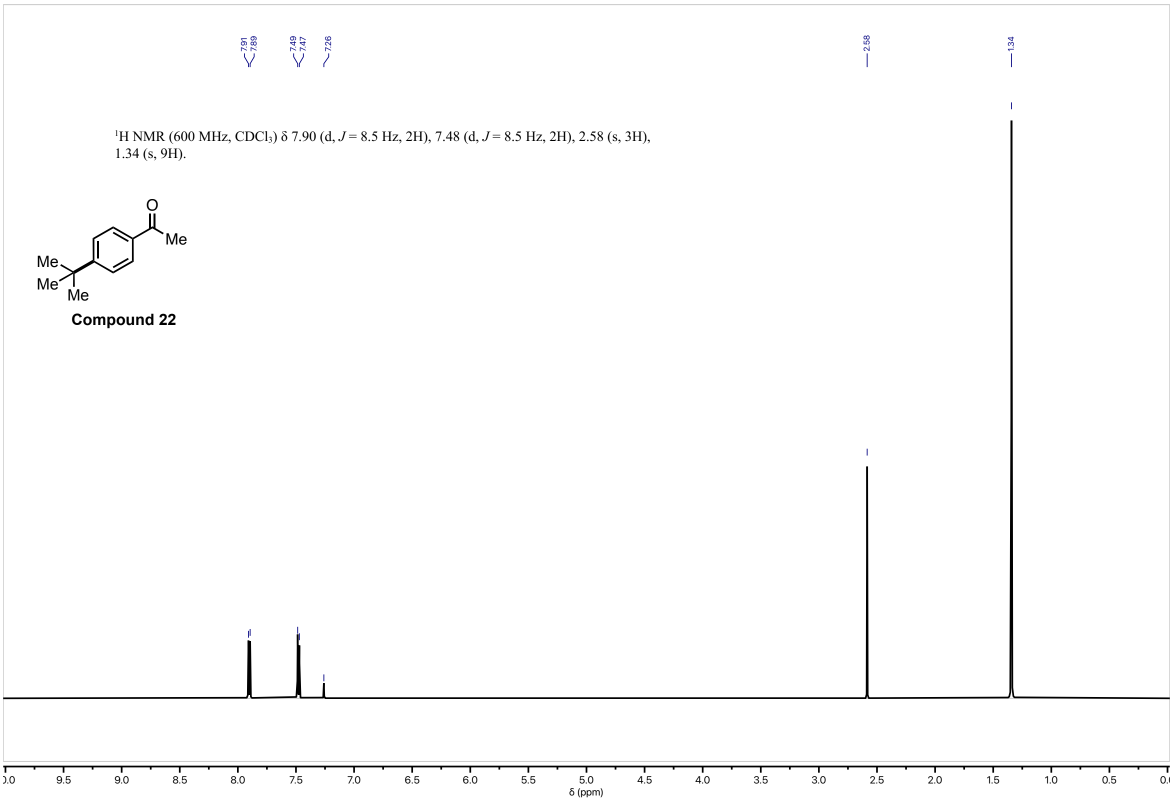
7.91
7.89
7.49
7.47
7.26
2.58
1.34
^{1}H NMR (600 MHz, $CDCl_3$) δ 7.90 (d, J = 8.5 Hz, 2H), 7.48 (d, J = 8.5 Hz, 2H), 2.58 (s, 3H), 1.34 (s, 9H).
O
Me
Me
Me
Me
Compound 22
δ (ppm)

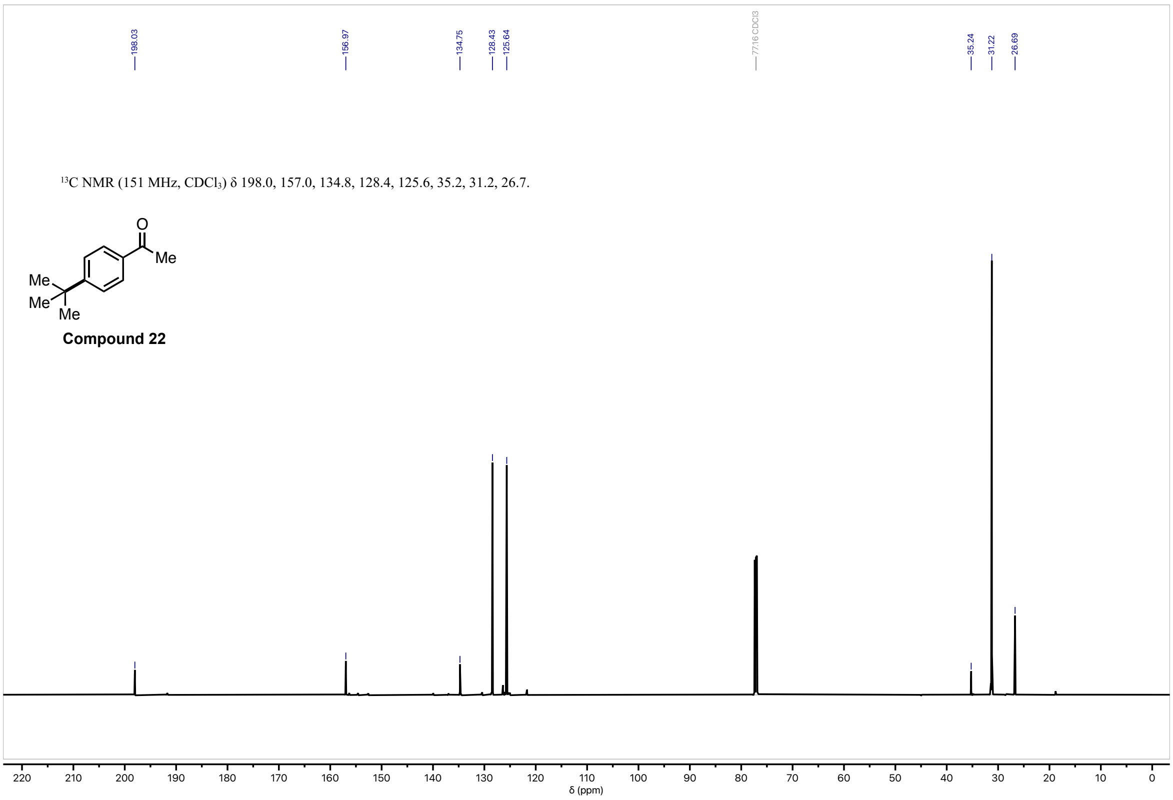

^{13}C NMR (151 MHz, $CDCl_3$) δ 198.0, 157.0, 134.8, 128.4, 125.6, 35.2, 31.2, 26.7.

Compound 22

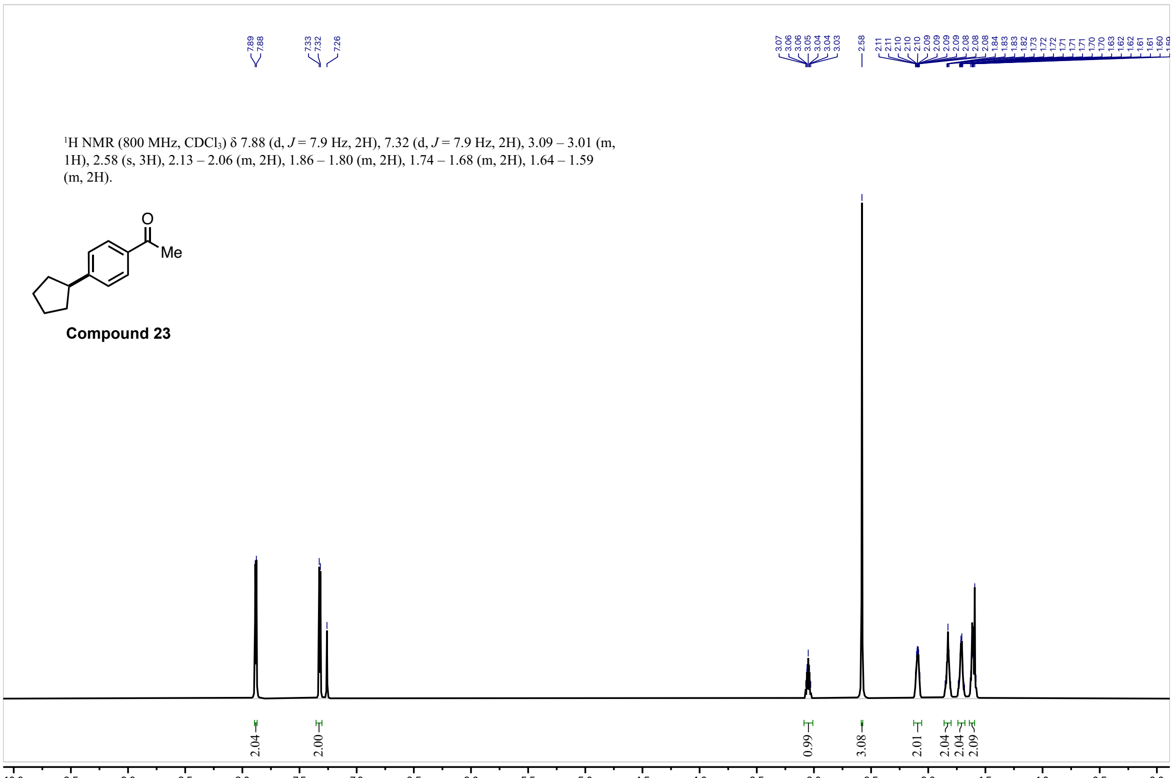

^{1}H NMR (800 MHz, $CDCl_3$) δ 7.88 (d, J = 7.9 Hz, 2H), 7.32 (d, J = 7.9 Hz, 2H), 3.09 – 3.01 (m, 1H), 2.58 (s, 3H), 2.13 – 2.06 (m, 2H), 1.86 – 1.80 (m, 2H), 1.74 – 1.68 (m, 2H), 1.64 – 1.59 (m, 2H).

Compound 23

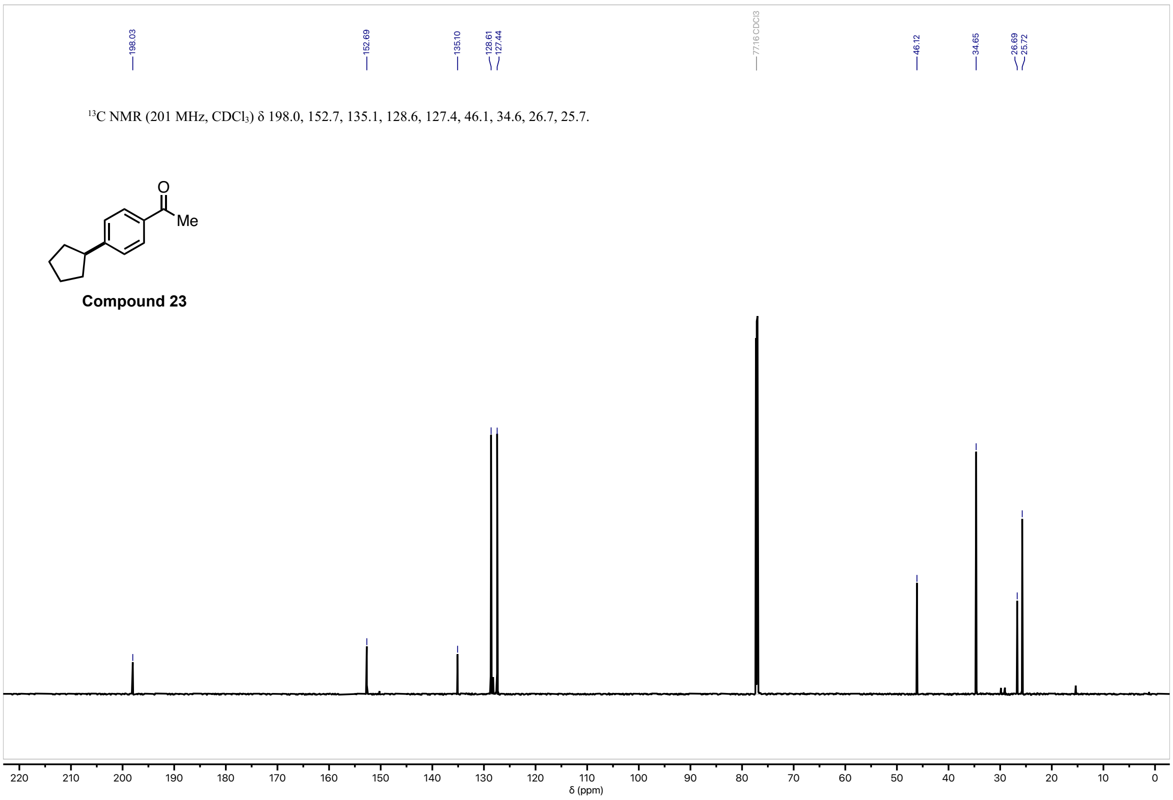
198.03
152.69
135.10
128.61
127.44
77.16 CDCl3
46.12
34.65
26.69
25.72
^{13}C NMR (201 MHz, $CDCl_3$) δ 198.0, 152.7, 135.1, 128.6, 127.4, 46.1, 34.6, 26.7, 25.7.
O
Me
Compound 23
δ (ppm)

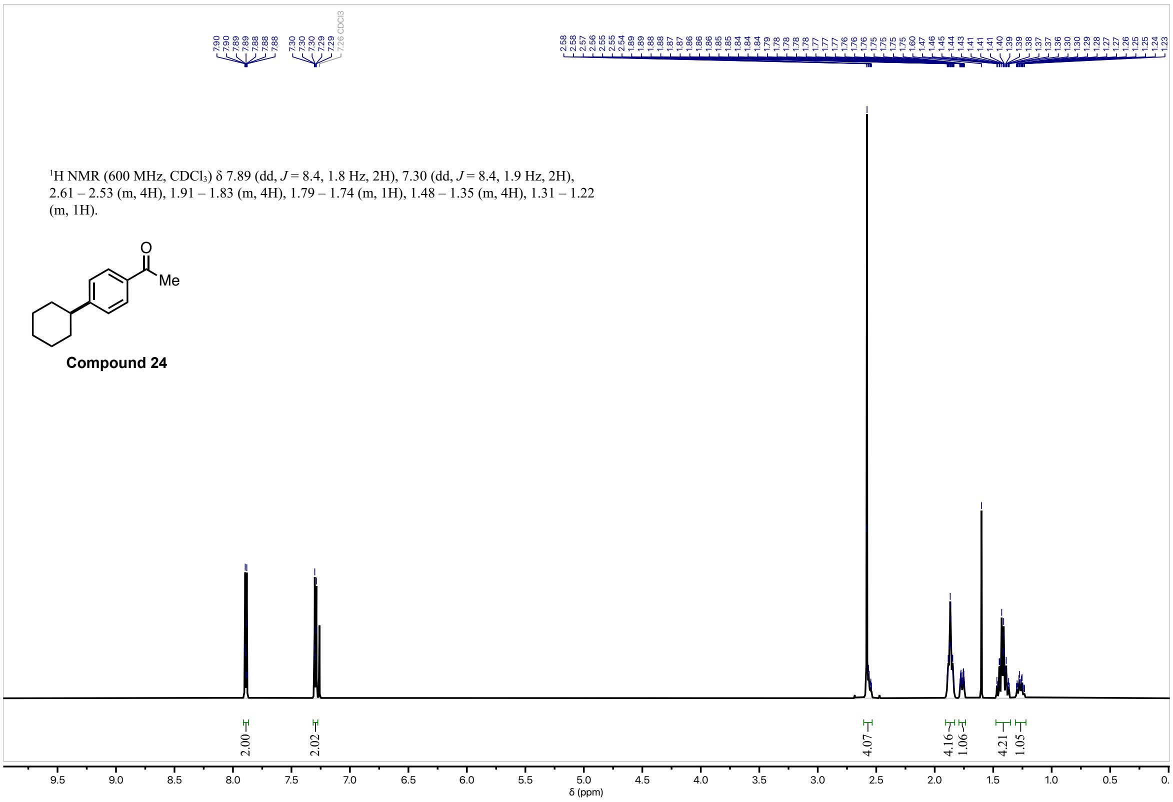
7.90
7.90
7.89
7.89
7.88
7.88
7.88
7.30
7.30
7.30
7.29
7.29
7.26 CDCl3
^{1}H NMR (600 MHz, $CDCl_3$) δ 7.89 (dd, J = 8.4, 1.8 Hz, 2H), 7.30 (dd, J = 8.4, 1.9 Hz, 2H), 2.61 – 2.53 (m, 4H), 1.91 – 1.83 (m, 4H), 1.79 – 1.74 (m, 1H), 1.48 – 1.35 (m, 4H), 1.31 – 1.22 (m, 1H).
O
Me
Compound 24
2.00
2.02
4.07
4.16
1.06
4.21
1.05
δ (ppm)

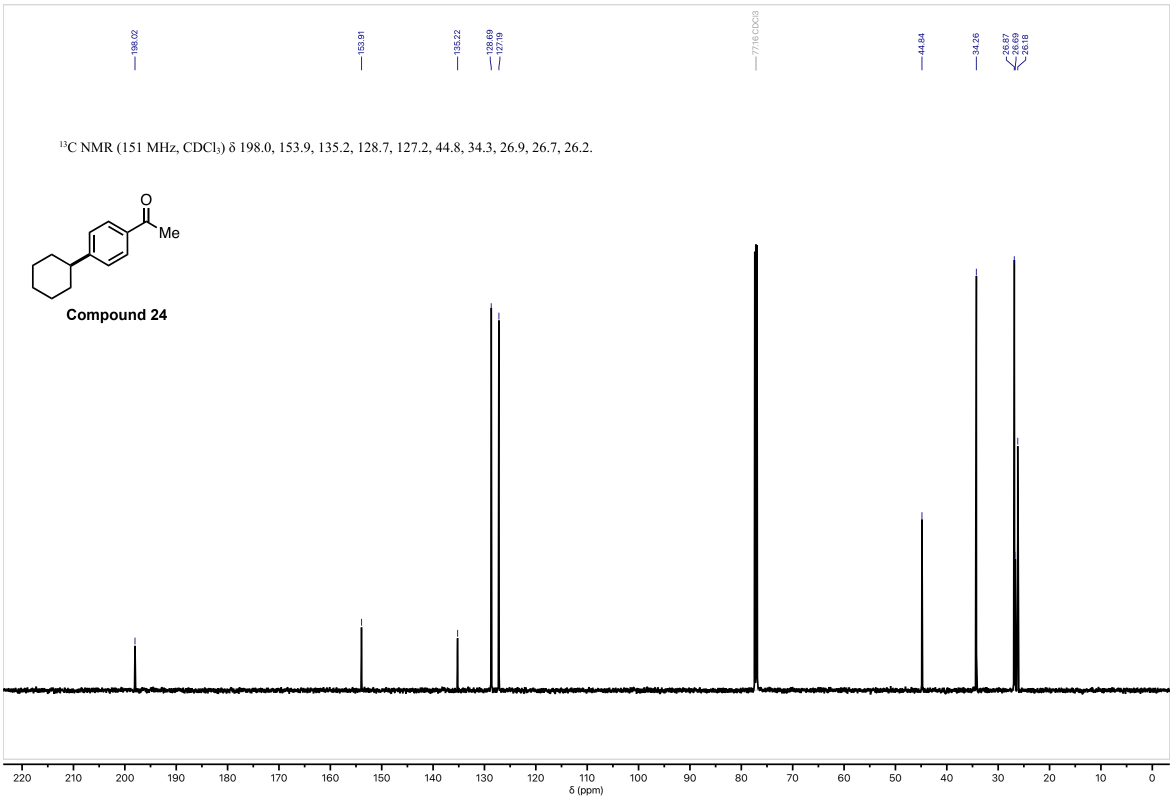

^{13}C NMR (151 MHz, CDCl$_3$) δ 198.0, 153.9, 135.2, 128.7, 127.2, 44.8, 34.3, 26.9, 26.7, 26.2.

Compound 24

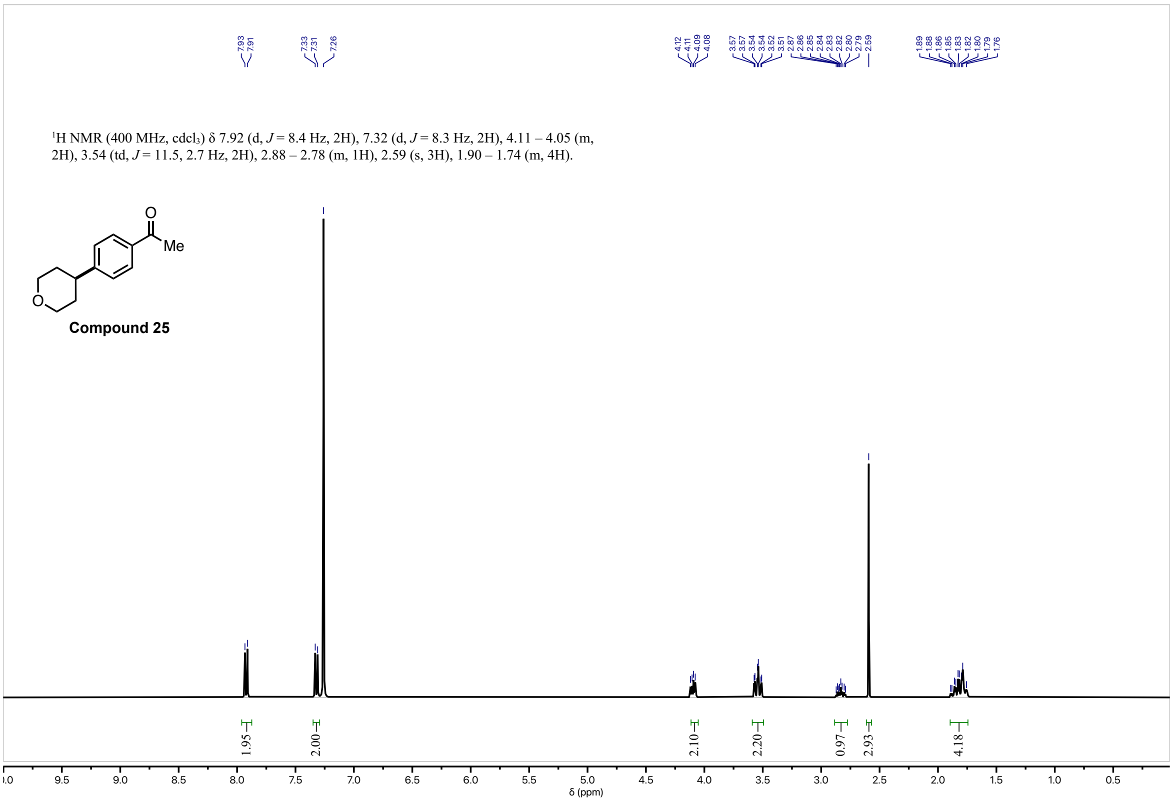

^{1}H NMR (400 MHz, cdcl$_3$) δ 7.92 (d, J = 8.4 Hz, 2H), 7.32 (d, J = 8.3 Hz, 2H), 4.11 – 4.05 (m, 2H), 3.54 (td, J = 11.5, 2.7 Hz, 2H), 2.88 – 2.78 (m, 1H), 2.59 (s, 3H), 1.90 – 1.74 (m, 4H).

Compound 25

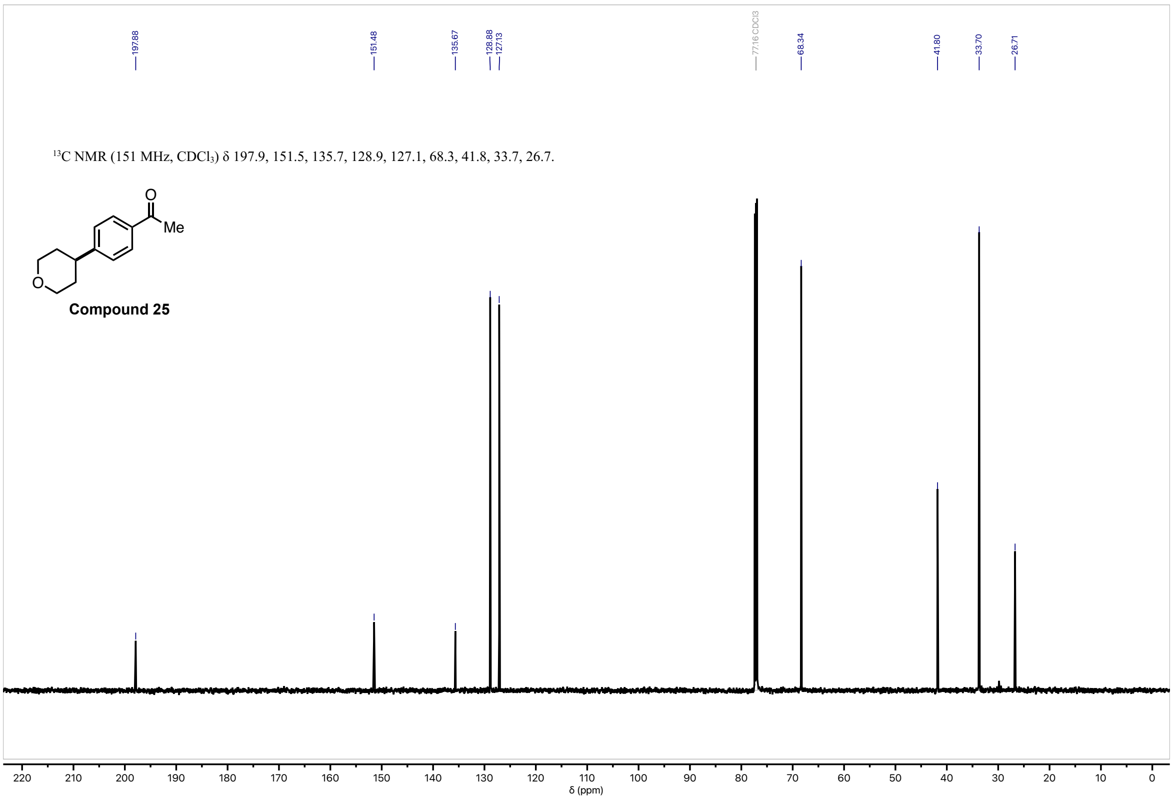

^{13}C NMR (151 MHz, $CDCl_3$) δ 197.9, 151.5, 135.7, 128.9, 127.1, 68.3, 41.8, 33.7, 26.7.

Compound 25

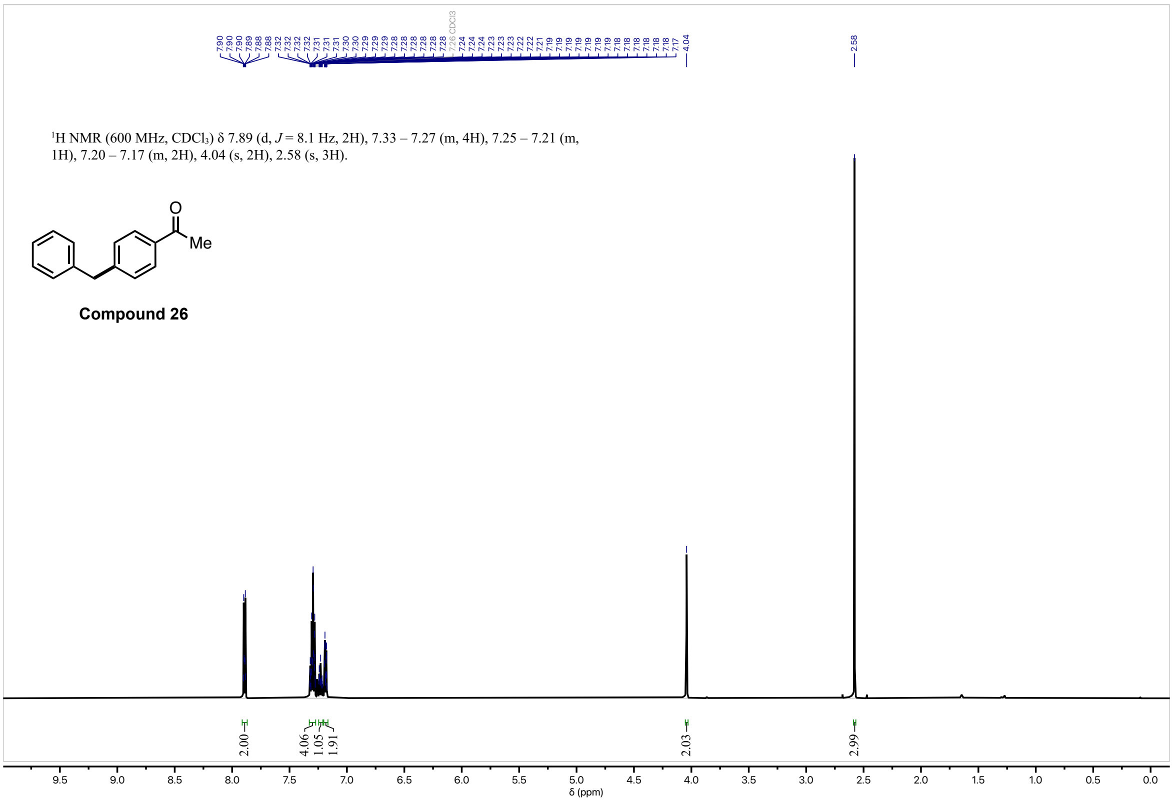

^{1}H NMR (600 MHz, $CDCl_3$) δ 7.89 (d, J = 8.1 Hz, 2H), 7.33 – 7.27 (m, 4H), 7.25 – 7.21 (m, 1H), 7.20 – 7.17 (m, 2H), 4.04 (s, 2H), 2.58 (s, 3H).

Compound 26

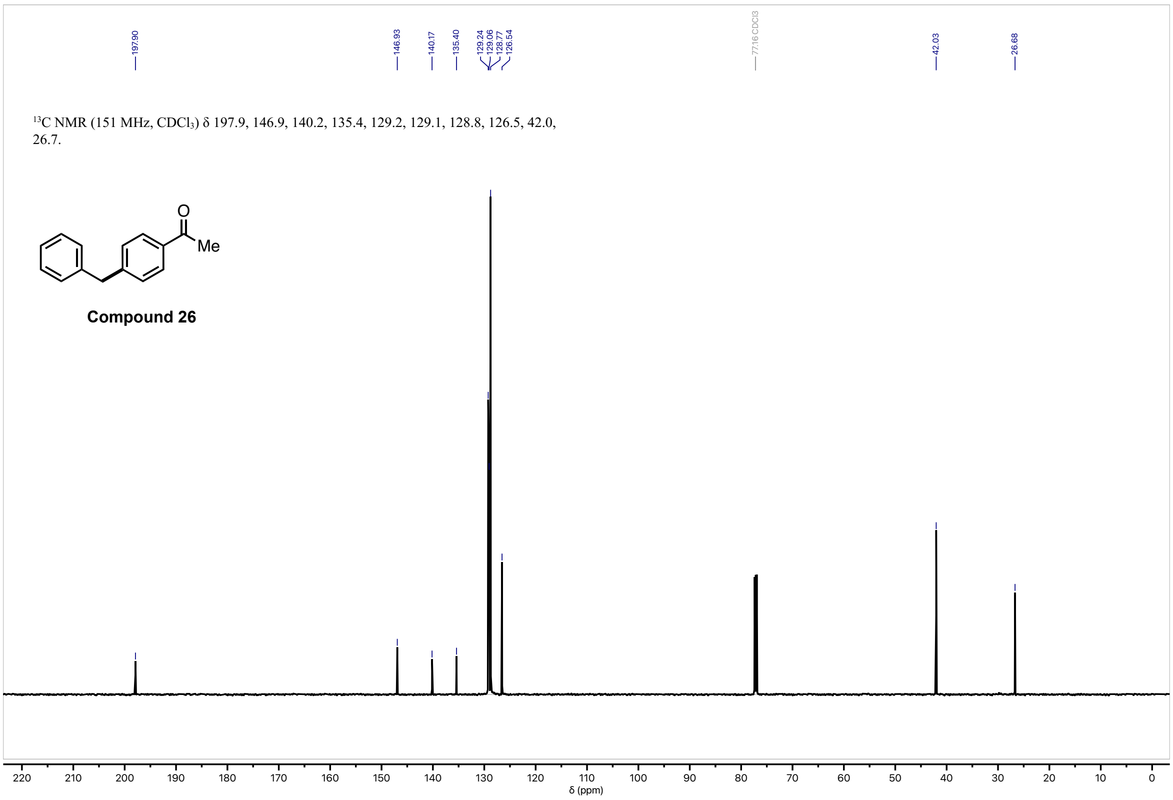
^{13}C NMR (151 MHz, CDCl$_3$) δ 197.9, 146.9, 140.2, 135.4, 129.2, 129.1, 128.8, 126.5, 42.0, 26.7.
O
Me
Compound 26
δ (ppm)

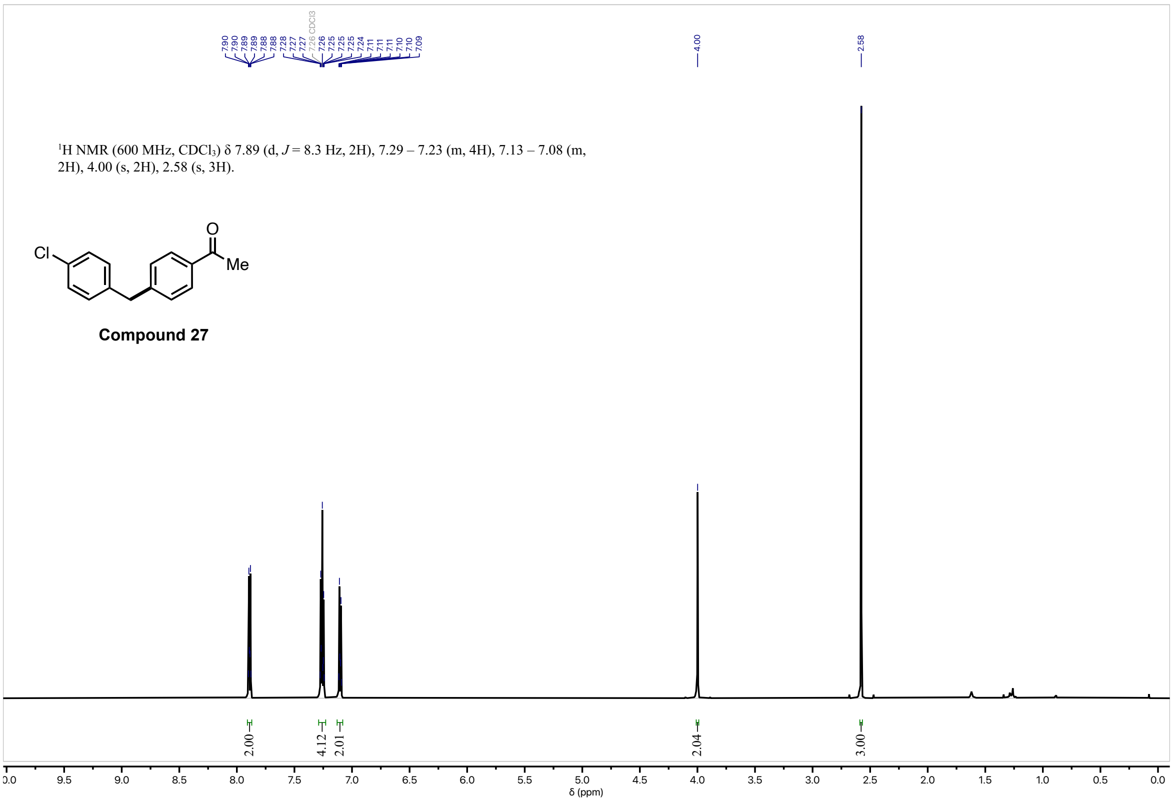
^{1}H NMR (600 MHz, CDCl$_3$) δ 7.89 (d, J = 8.3 Hz, 2H), 7.29 – 7.23 (m, 4H), 7.13 – 7.08 (m, 2H), 4.00 (s, 2H), 2.58 (s, 3H).
Cl
O
Me
Compound 27
δ (ppm)

^{13}C NMR (151 MHz, $CDCl_3$) δ 197.8, 146.3, 138.6, 135.6, 132.4, 130.4, 129.2, 128.9, 128.9, 41.3, 26.7.

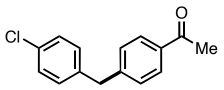

Compound 27

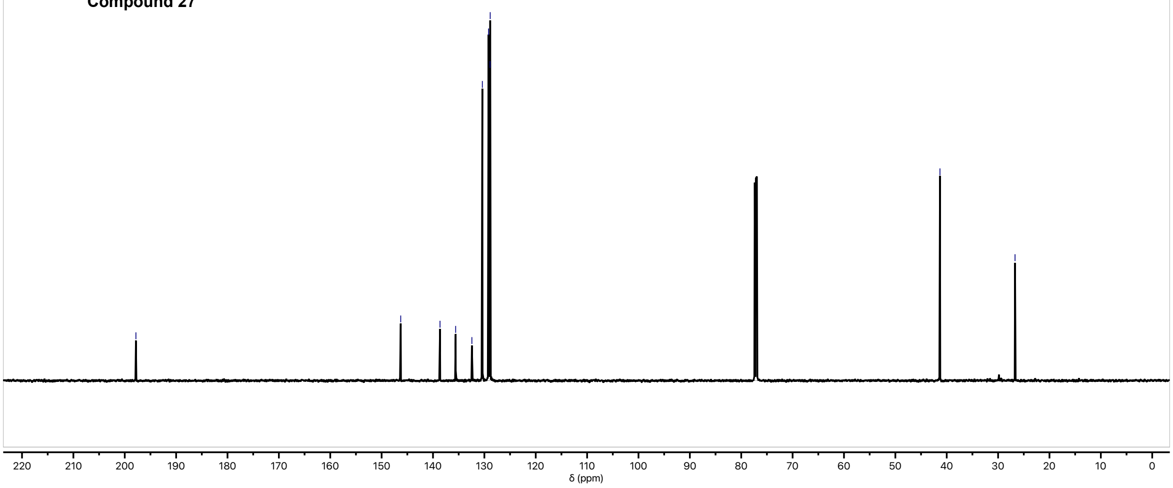

^{1}H NMR (600 MHz, $CDCl_3$) δ 7.89 (d, J = 8.3 Hz, 2H), 7.26 (d, J = 8.3 Hz, 2H), 7.17 – 7.10 (m, 2H), 7.02 – 6.95 (m, 2H), 4.00 (s, 2H), 2.58 (s, 3H).

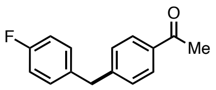

Compound 28

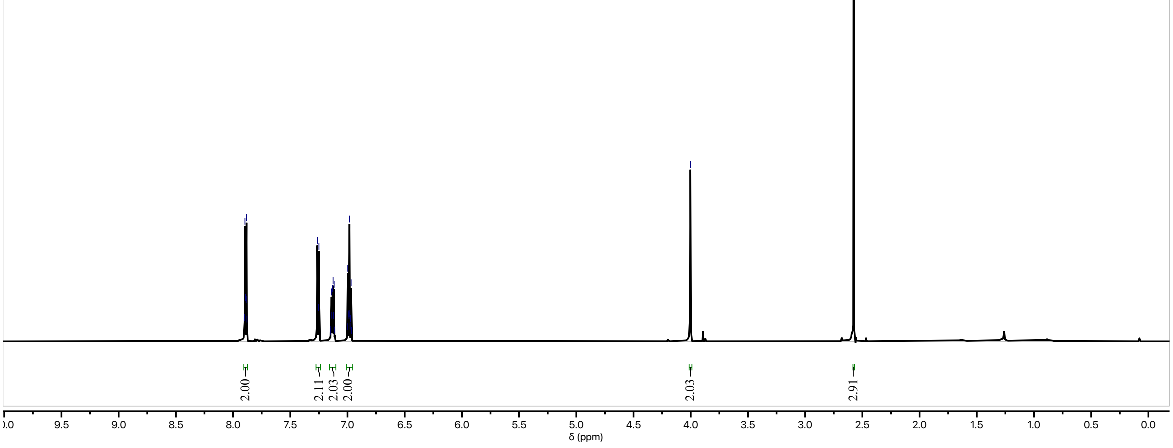

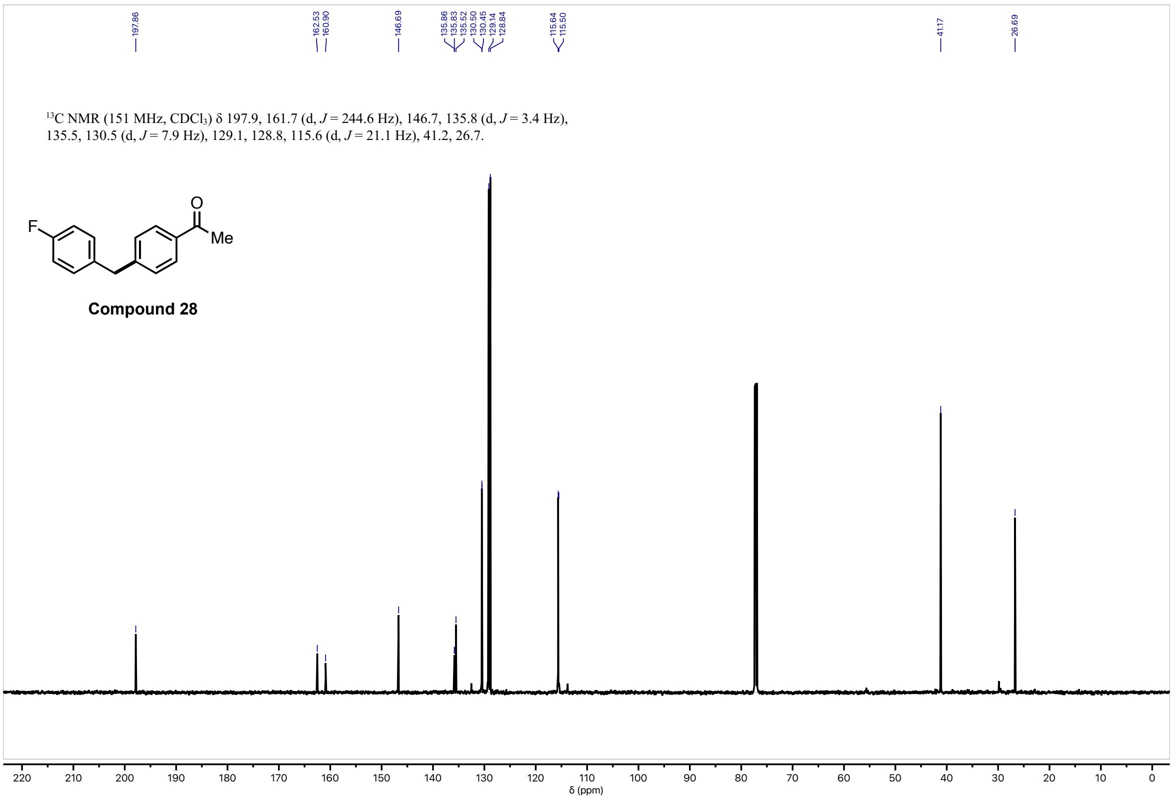
^{13}C NMR (151 MHz, CDCl$_3$) δ 197.9, 161.7 (d, J = 244.6 Hz), 146.7, 135.8 (d, J = 3.4 Hz), 135.5, 130.5 (d, J = 7.9 Hz), 129.1, 128.8, 115.6 (d, J = 21.1 Hz), 41.2, 26.7.
F
O
Me
Compound 28
δ (ppm)

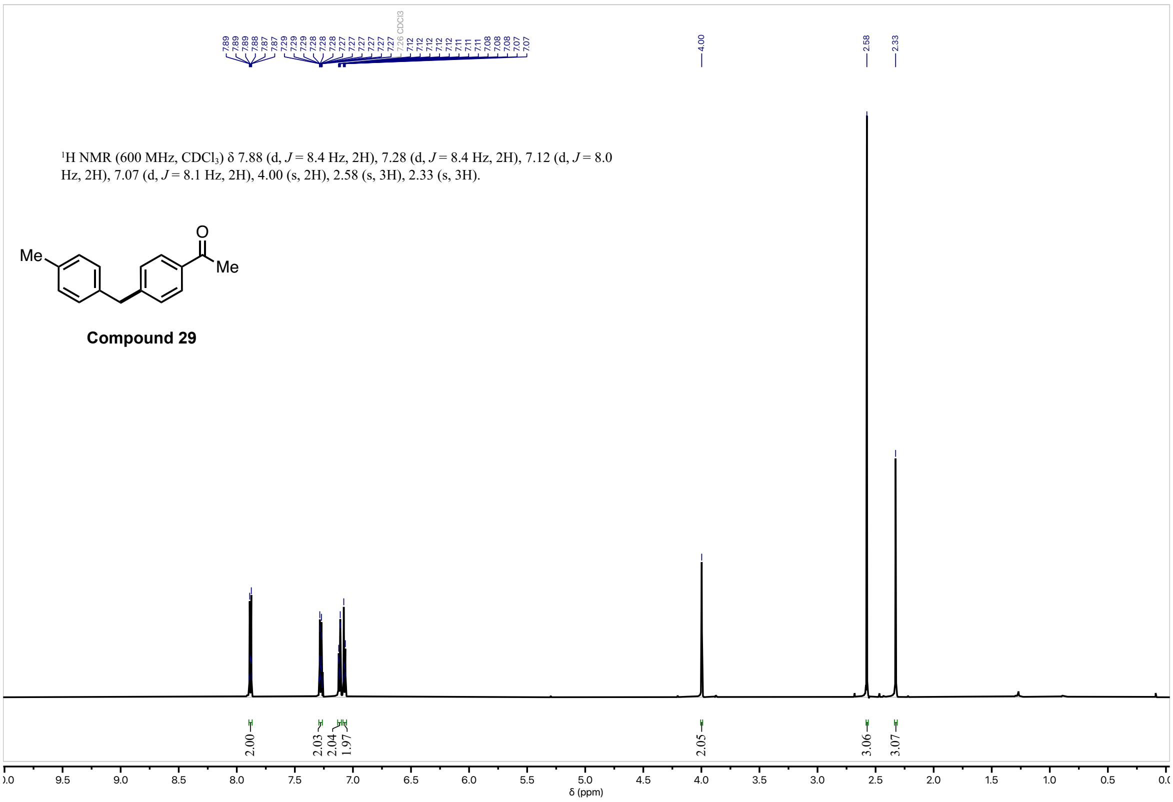
^{1}H NMR (600 MHz, CDCl$_3$) δ 7.88 (d, J = 8.4 Hz, 2H), 7.28 (d, J = 8.4 Hz, 2H), 7.12 (d, J = 8.0 Hz, 2H), 7.07 (d, J = 8.1 Hz, 2H), 4.00 (s, 2H), 2.58 (s, 3H), 2.33 (s, 3H).
Me
O
Me
Compound 29
δ (ppm)

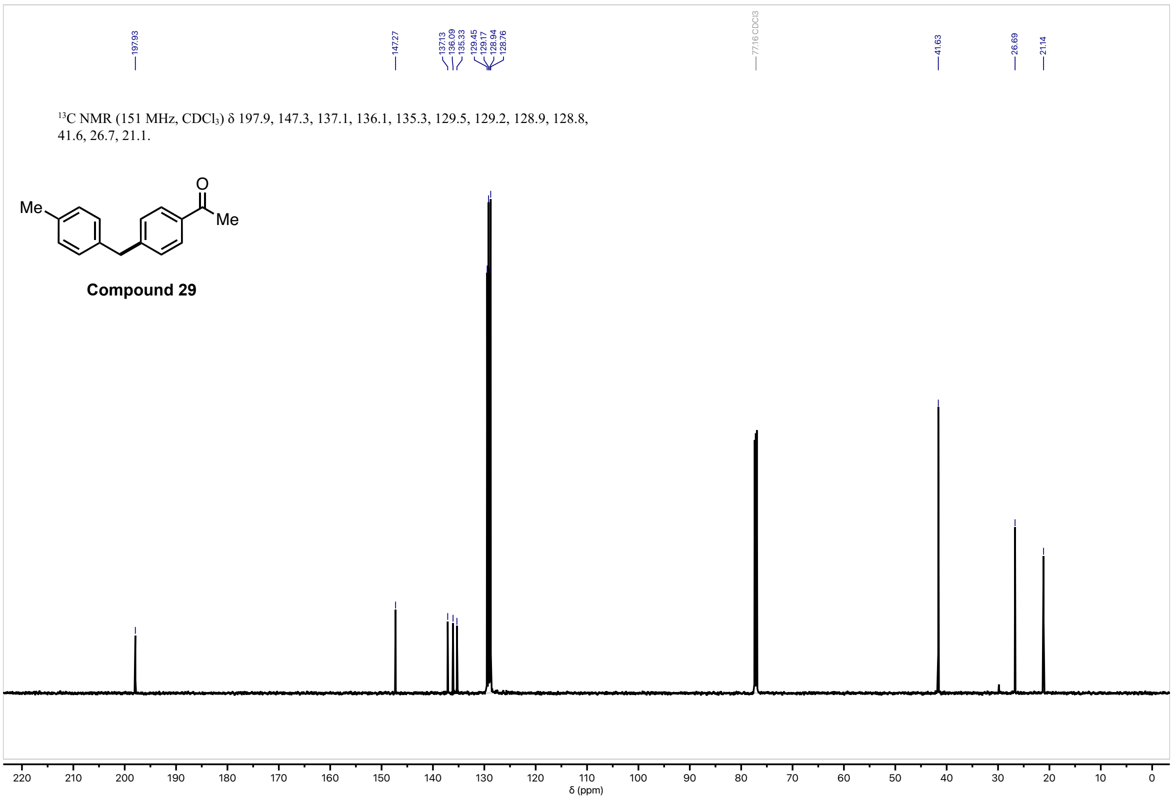

Compound 29

^{13}C NMR (151 MHz, $CDCl_3$) δ 197.9, 147.3, 137.1, 136.1, 135.3, 129.5, 129.2, 128.9, 128.8, 41.6, 26.7, 21.1.

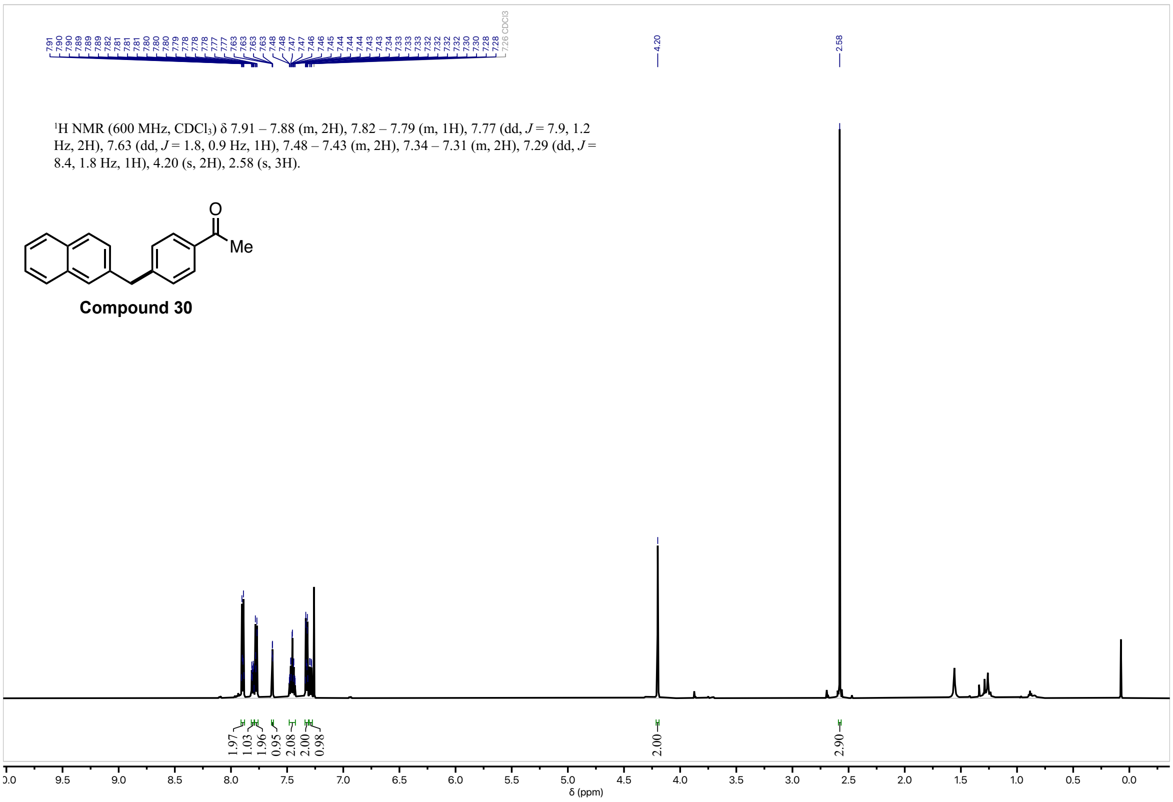

Compound 30

^{1}H NMR (600 MHz, $CDCl_3$) δ 7.91 – 7.88 (m, 2H), 7.82 – 7.79 (m, 1H), 7.77 (dd, J = 7.9, 1.2 Hz, 2H), 7.63 (dd, J = 1.8, 0.9 Hz, 1H), 7.48 – 7.43 (m, 2H), 7.34 – 7.31 (m, 2H), 7.29 (dd, J = 8.4, 1.8 Hz, 1H), 4.20 (s, 2H), 2.58 (s, 3H).

^{13}C NMR (151 MHz, $CDCl_3$) δ 197.9, 146.8, 137.7, 135.5, 133.7, 132.3, 129.4, 128.8, 128.5, 127.8, 127.7, 127.5, 127.4, 126.3, 125.7, 42.2, 26.7.

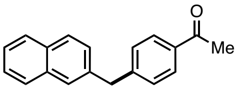

Compound 30

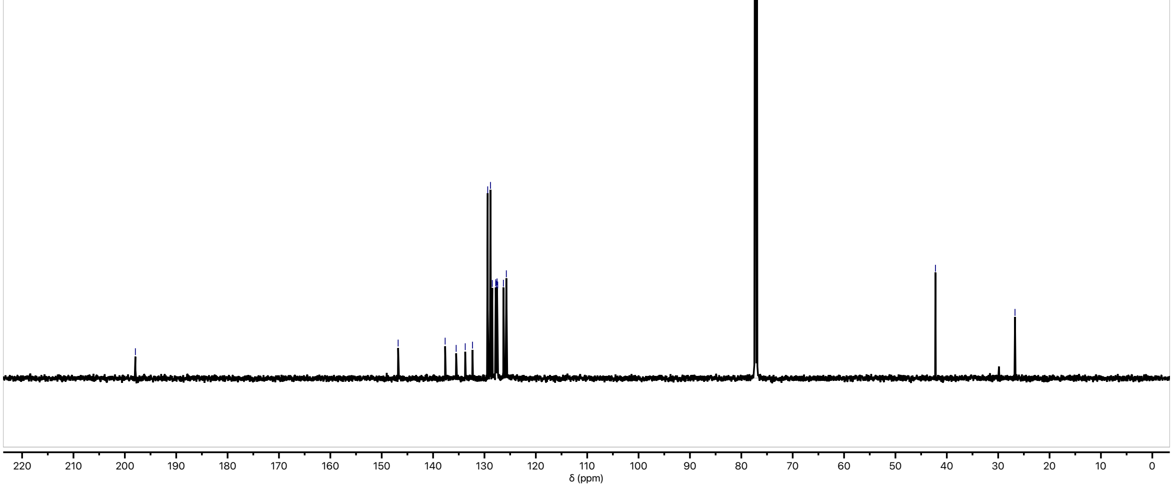

^{1}H NMR (600 MHz, $CDCl_3$) δ 7.92 – 7.88 (m, 2H), 7.36 – 7.29 (m, 3H), 6.31 (dd, J = 3.2, 1.9 Hz, 1H), 6.07 – 6.02 (m, 1H), 4.03 (s, 2H), 2.58 (s, 3H).

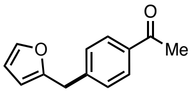

Compound 31

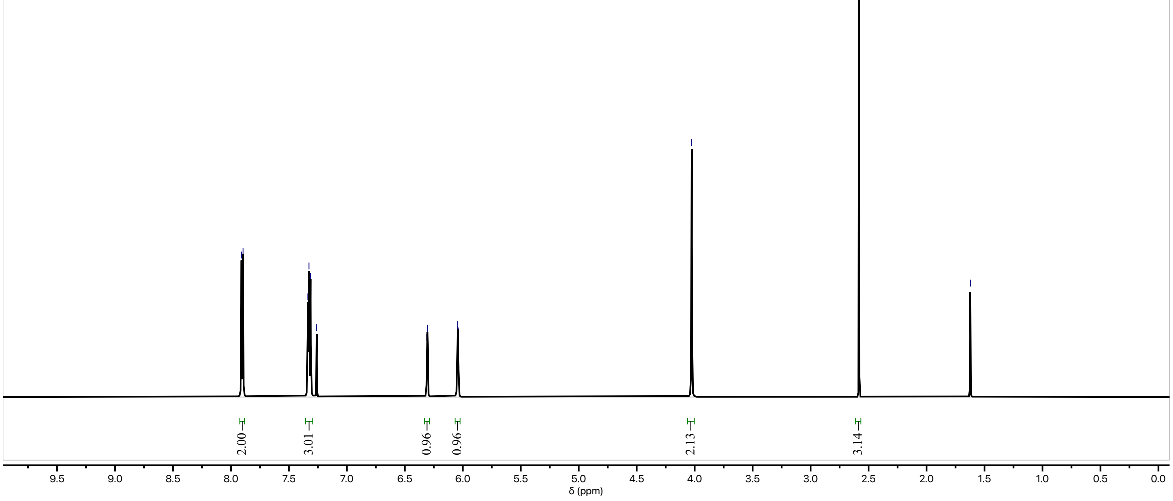

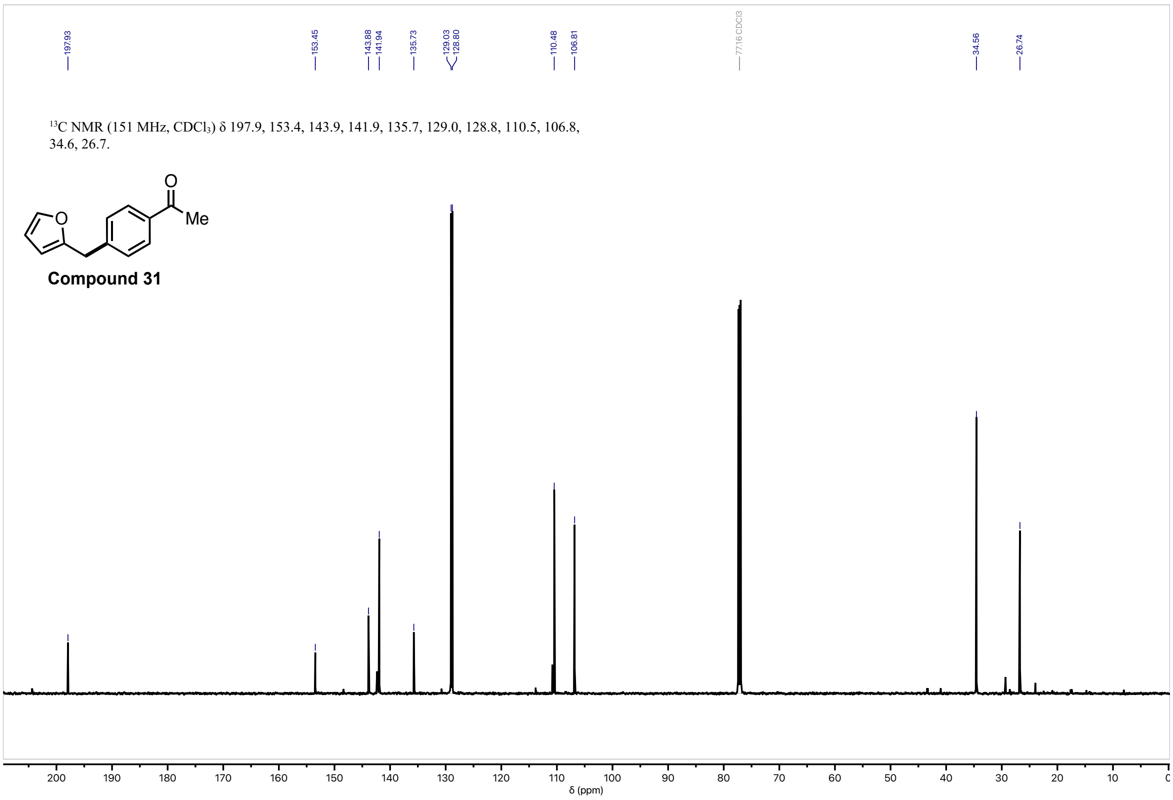

^{13}C NMR (151 MHz, $CDCl_3$) δ 197.9, 153.4, 143.9, 141.9, 135.7, 129.0, 128.8, 110.5, 106.8, 34.6, 26.7.

Compound 31

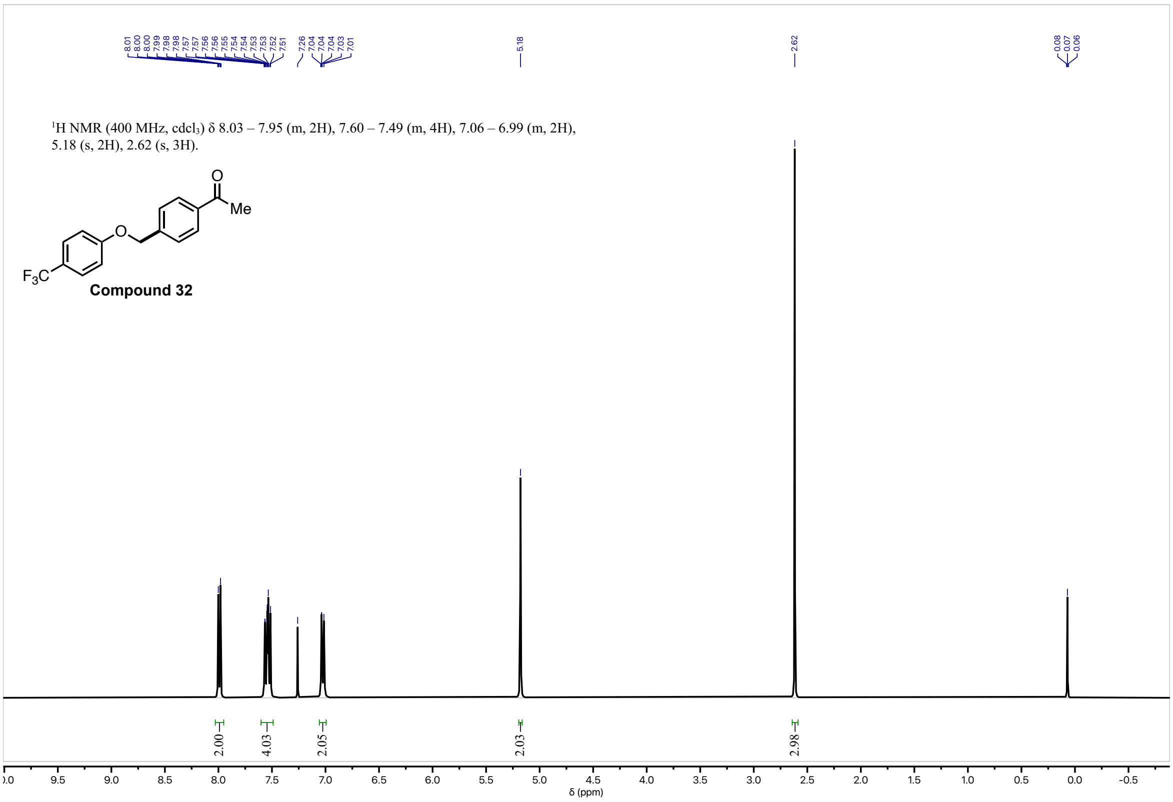

^{1}H NMR (400 MHz, $cdcl_3$) δ 8.03 – 7.95 (m, 2H), 7.60 – 7.49 (m, 4H), 7.06 – 6.99 (m, 2H), 5.18 (s, 2H), 2.62 (s, 3H).

Compound 32

^{13}C NMR (151 MHz, $CDCl_3$) δ 197.7, 160.9, 141.7, 137.1, 128.9, 127.3, 127.2 (q, *J* = 3.8 Hz), 124.5 (q, *J* = 271.2 Hz), 123.6 (q, *J* = 32.9 Hz), 115.0, 114.8, 69.6, 26.8.

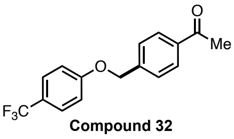

Compound 32

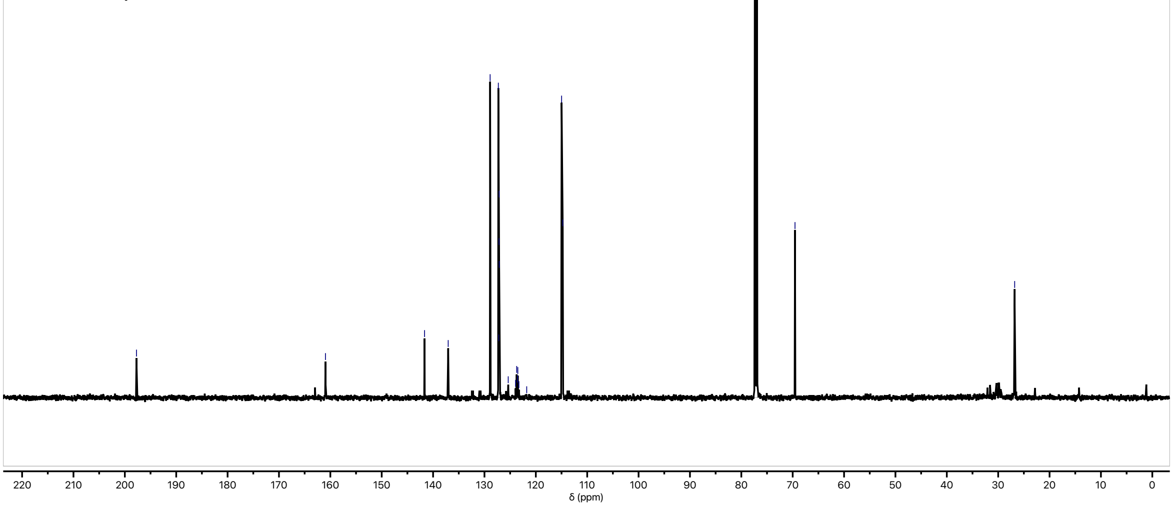

^{1}H NMR (600 MHz, $CDCl_3$) δ 8.00 – 7.96 (m, 2H), 7.53 – 7.48 (m, 2H), 7.26 – 7.22 (m, 2H), 6.91 – 6.86 (m, 2H), 5.10 (s, 2H), 2.61 (s, 3H).

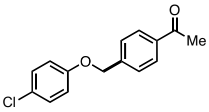

Compound 33

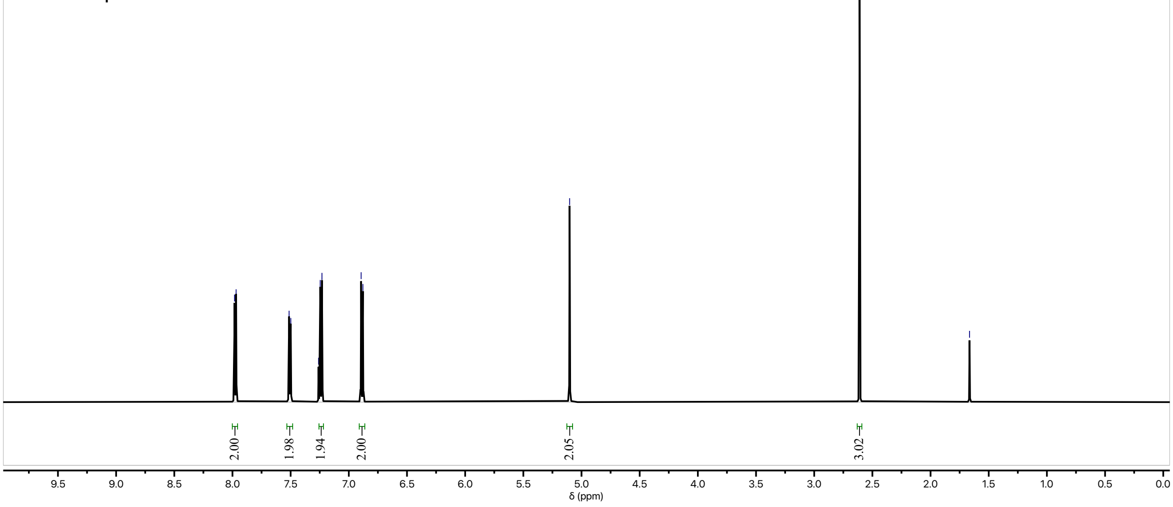

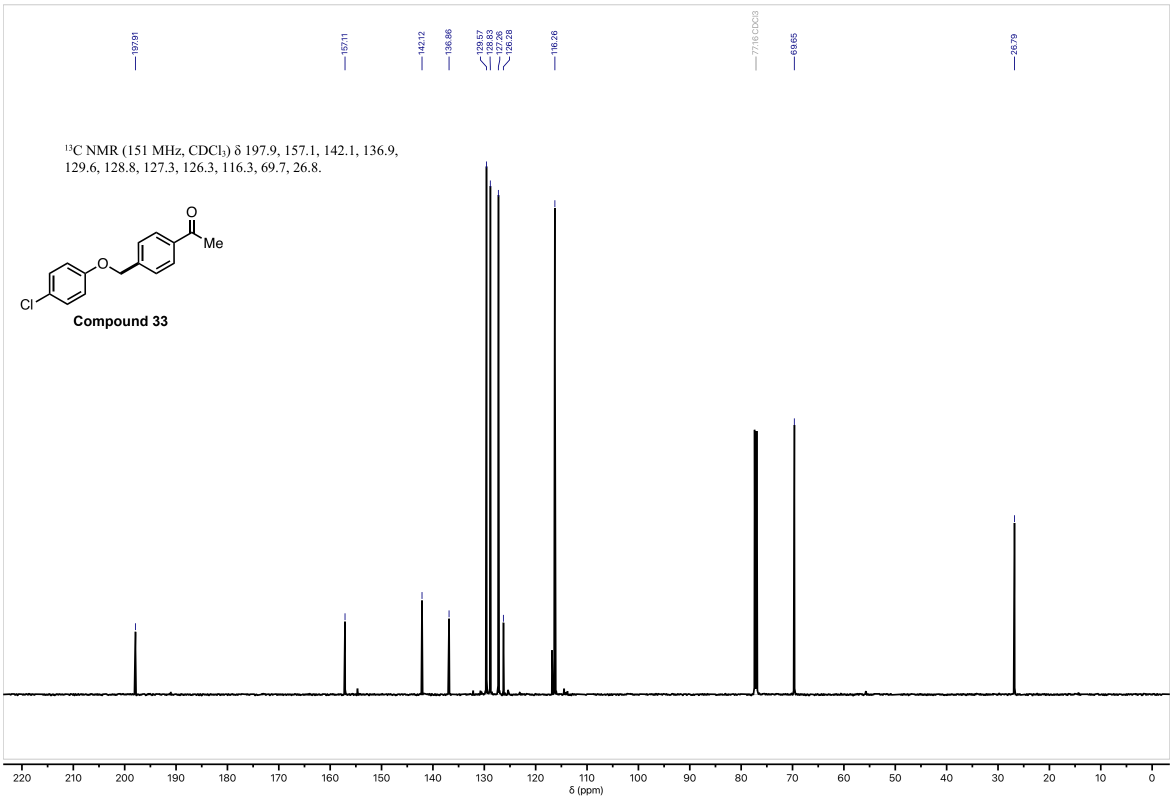

^{13}C NMR (151 MHz, CDCl$_3$) δ 197.9, 157.1, 142.1, 136.9, 129.6, 128.8, 127.3, 126.3, 116.3, 69.7, 26.8.
Compound 33
δ (ppm)

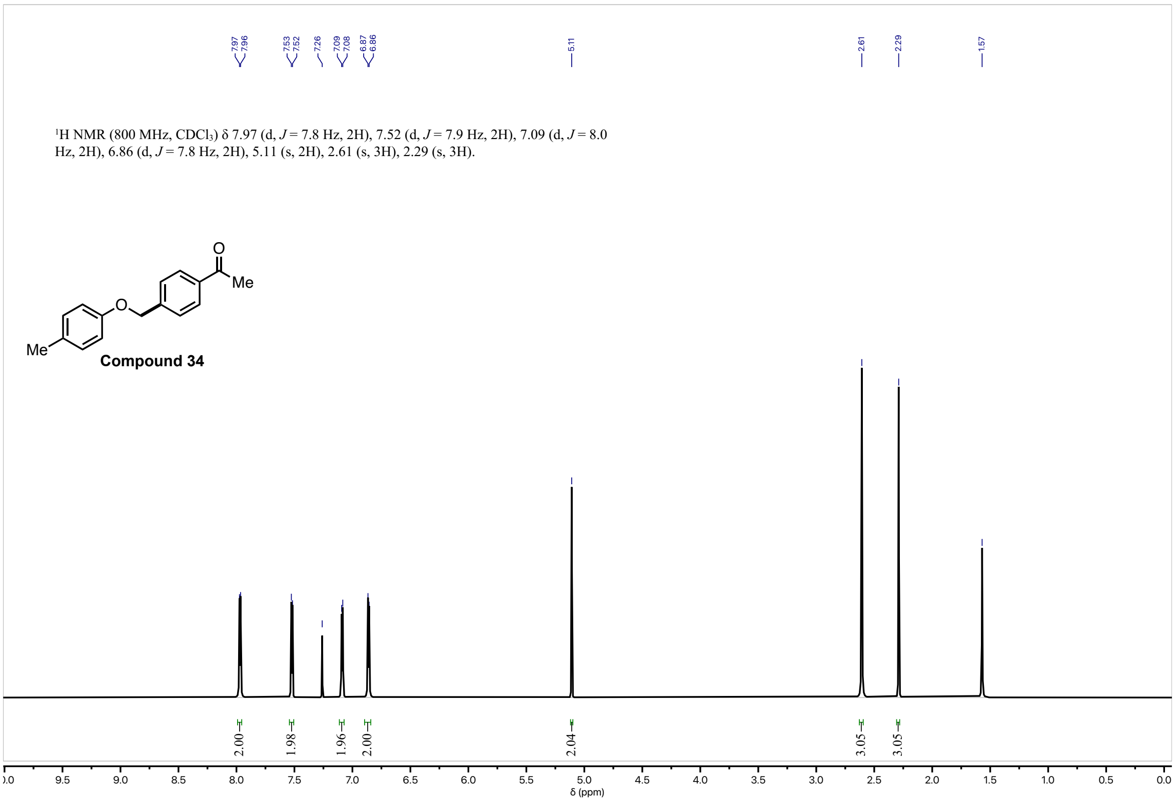

^{1}H NMR (800 MHz, CDCl$_3$) δ 7.97 (d, J = 7.8 Hz, 2H), 7.52 (d, J = 7.9 Hz, 2H), 7.09 (d, J = 8.0 Hz, 2H), 6.86 (d, J = 7.8 Hz, 2H), 5.11 (s, 2H), 2.61 (s, 3H), 2.29 (s, 3H).
Compound 34
δ (ppm)

^{13}C NMR (201 MHz, $CDCl_3$) δ 197.9, 156.5, 142.9, 136.7, 130.7, 130.1, 128.8, 127.3, 114.8, 69.5, 26.8, 20.6.

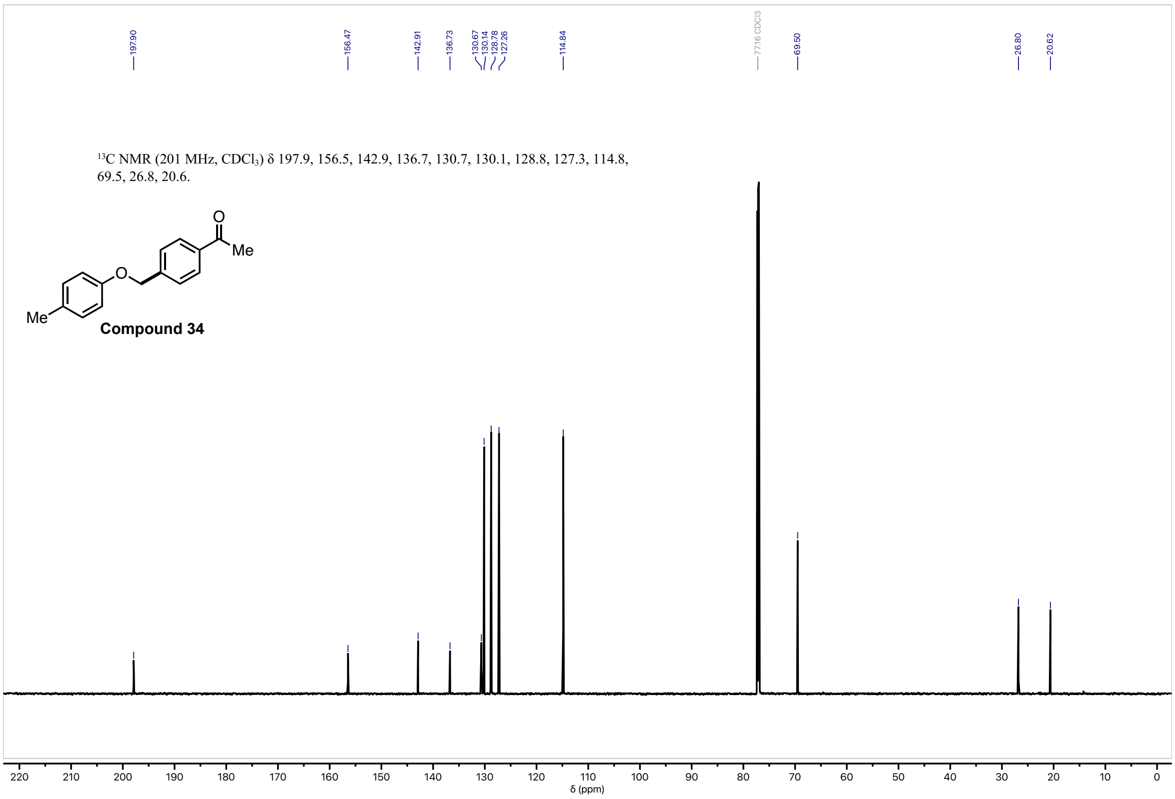

Compound 34

^{1}H NMR (600 MHz, $CDCl_3$) δ 8.00 – 7.96 (m, 2H), 7.54 – 7.49 (m, 2H), 7.01 – 6.95 (m, 2H), 6.91 – 6.88 (m, 2H), 5.09 (s, 2H), 2.61 (s, 3H).

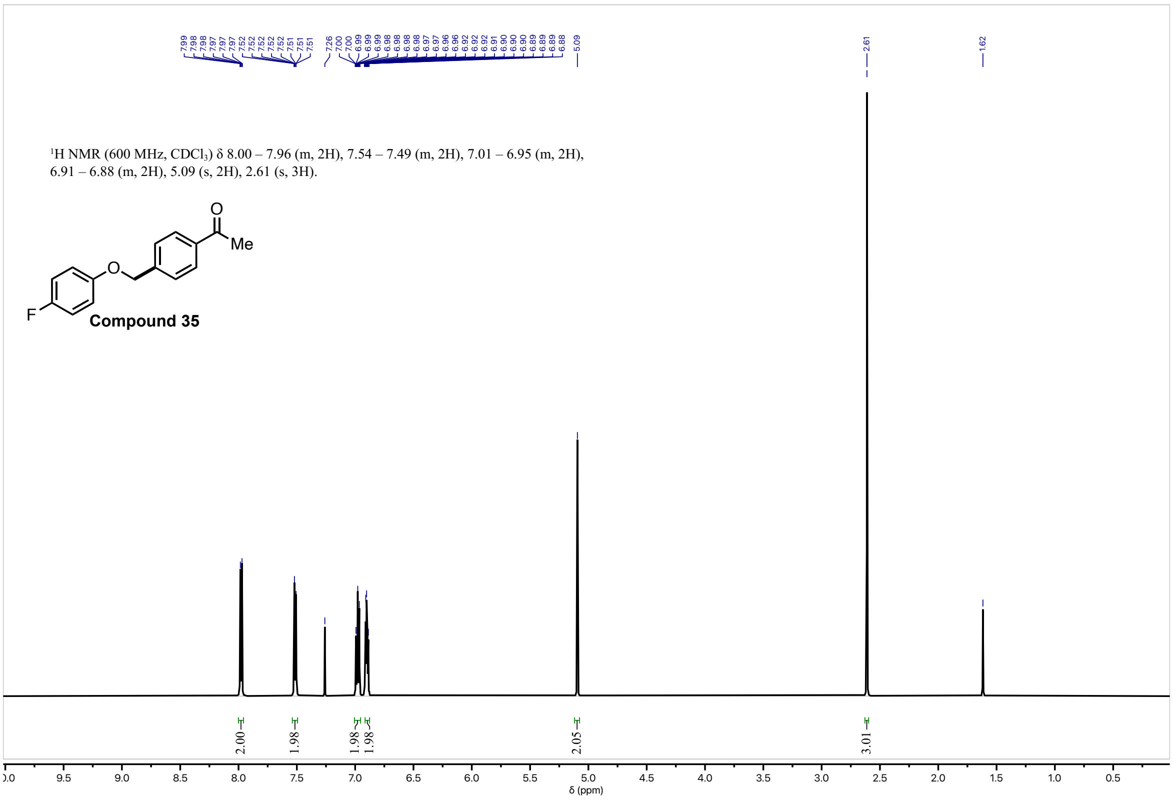

Compound 35

^{13}C NMR (151 MHz, $CDCl_3$) δ 197.9, 157.6 (d, *J* = 239.4 Hz), 154.7, 142.4, 136.8, 128.8, 127.3, 116.1 (d, *J* = 15.9 Hz), 116.0 (d, *J* = 7.6 Hz), 70.0, 26.8.

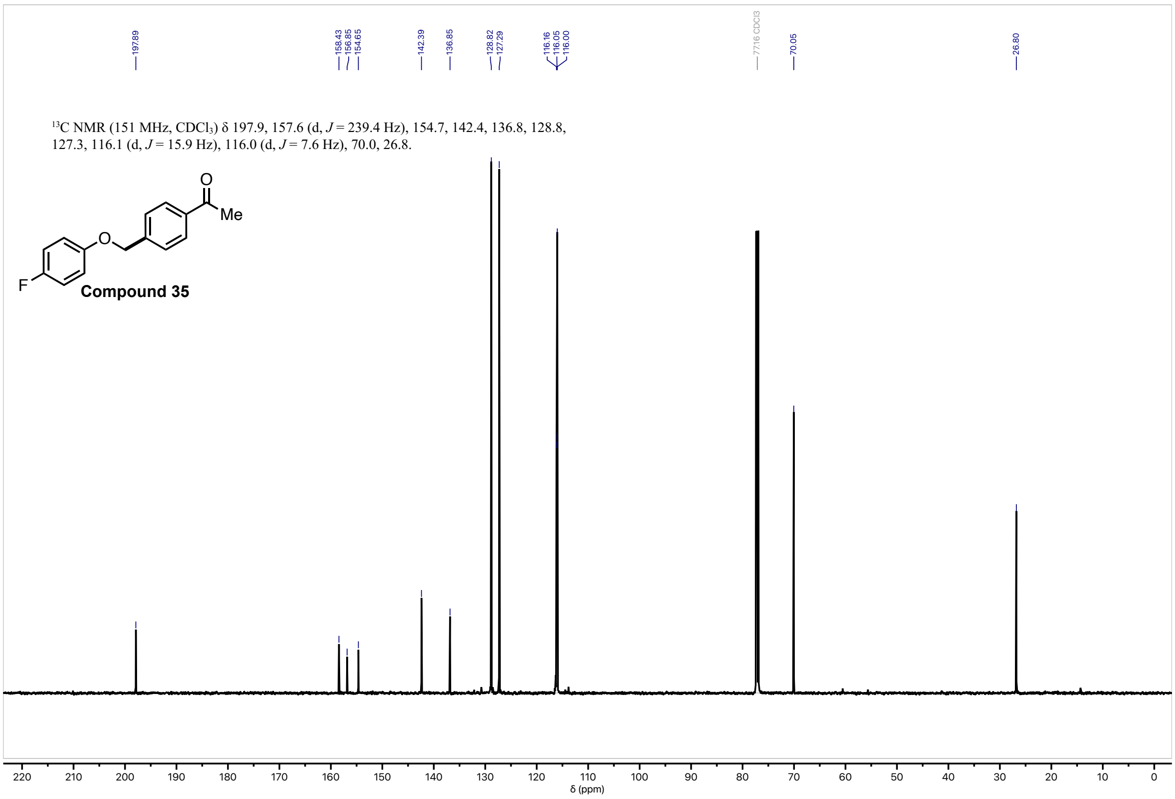

^{1}H NMR (600 MHz, $CDCl_3$) δ 8.00 – 7.96 (m, 2H), 7.53 – 7.49 (m, 2H), 7.12 (d, *J* = 8.4 Hz, 1H), 6.98 (d, *J* = 2.6 Hz, 1H), 6.77 (dd, *J* = 8.4, 2.6 Hz, 1H), 5.09 (s, 2H), 2.61 (s, 3H), 2.30 (s, 3H).

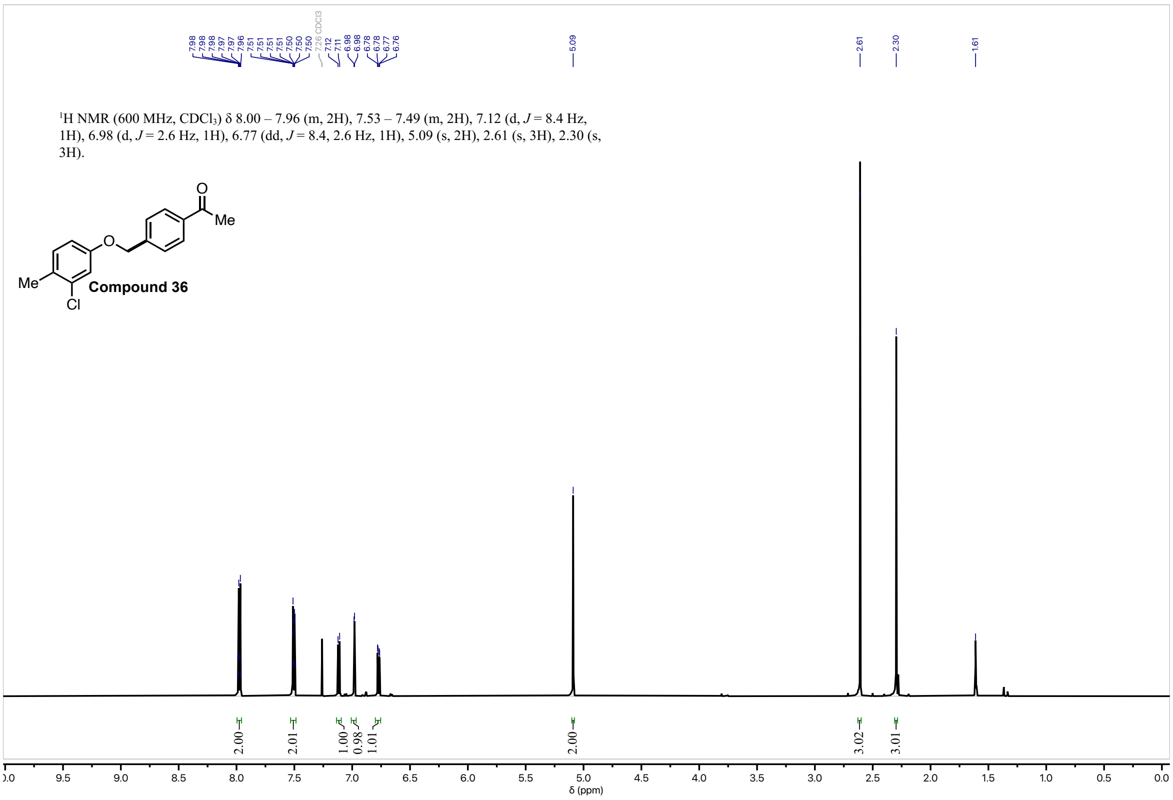

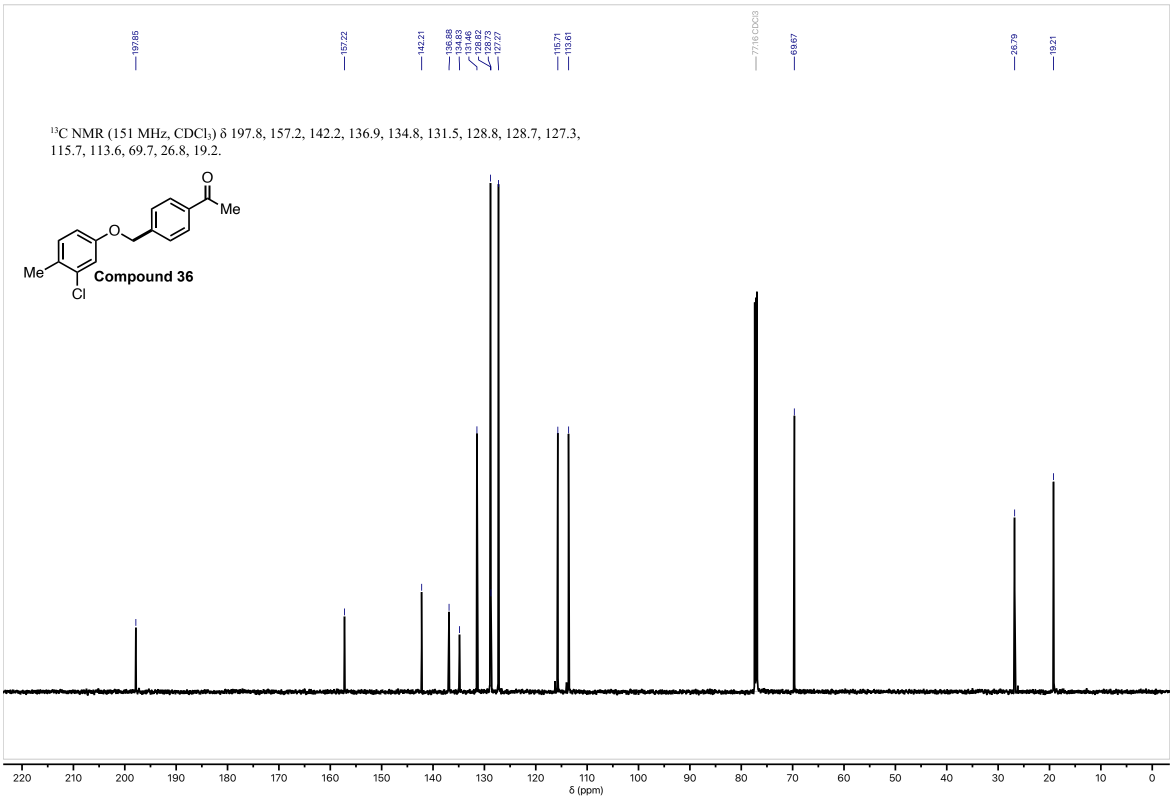

197.85
157.22
142.21
136.88
134.83
131.46
128.82
128.73
127.27
115.71
113.61
77.16 CDCl3
69.67
26.79
19.21
13C NMR (151 MHz, CDCl3) δ 197.8, 157.2, 142.2, 136.9, 134.8, 131.5, 128.8, 128.7, 127.3, 115.7, 113.6, 69.7, 26.8, 19.2.
O
Me
O
Me
Cl
Compound 36
δ (ppm)

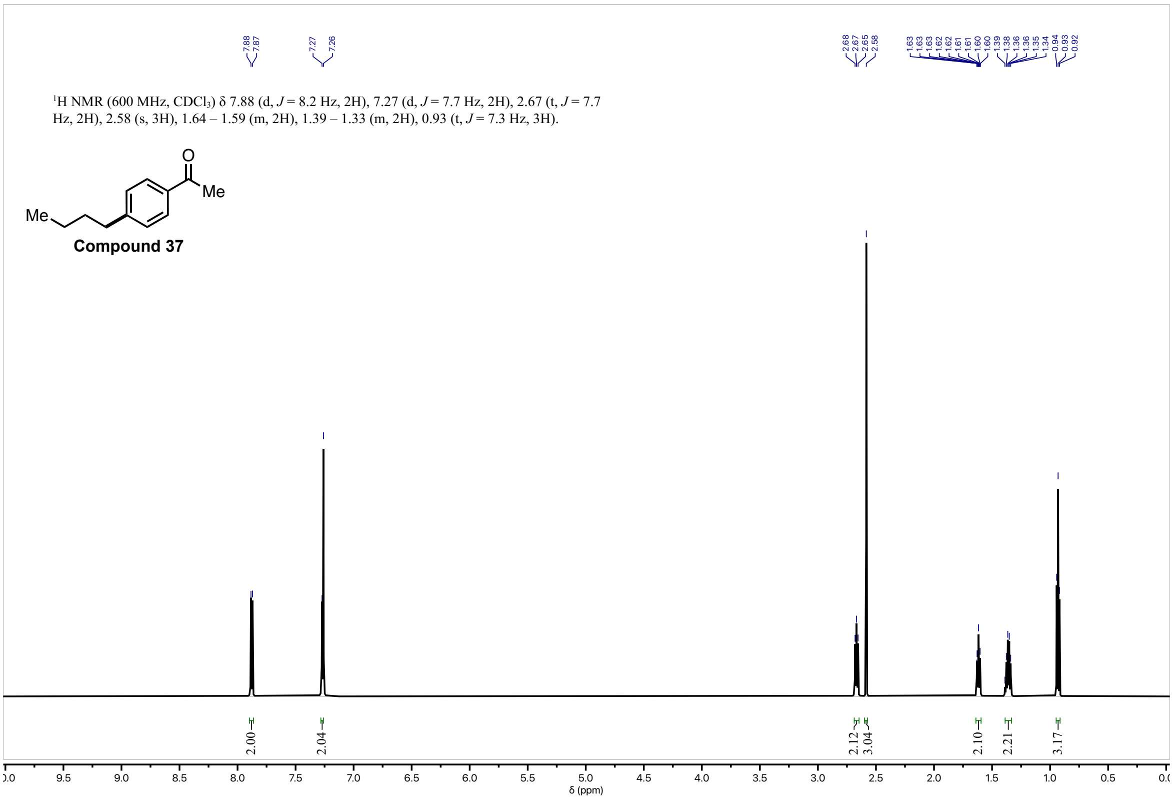

7.88
7.87
7.27
7.26
2.68
2.67
2.65
2.58
1.63
1.63
1.62
1.62
1.61
1.61
1.60
1.60
1.39
1.38
1.36
1.36
1.35
1.34
0.94
0.93
0.92
1H NMR (600 MHz, CDCl3) δ 7.88 (d, J = 8.2 Hz, 2H), 7.27 (d, J = 7.7 Hz, 2H), 2.67 (t, J = 7.7 Hz, 2H), 2.58 (s, 3H), 1.64 – 1.59 (m, 2H), 1.39 – 1.33 (m, 2H), 0.93 (t, J = 7.3 Hz, 3H).
O
Me
Me
Compound 37
2.00
2.04
2.12
3.04
2.10
2.21
3.17
δ (ppm)

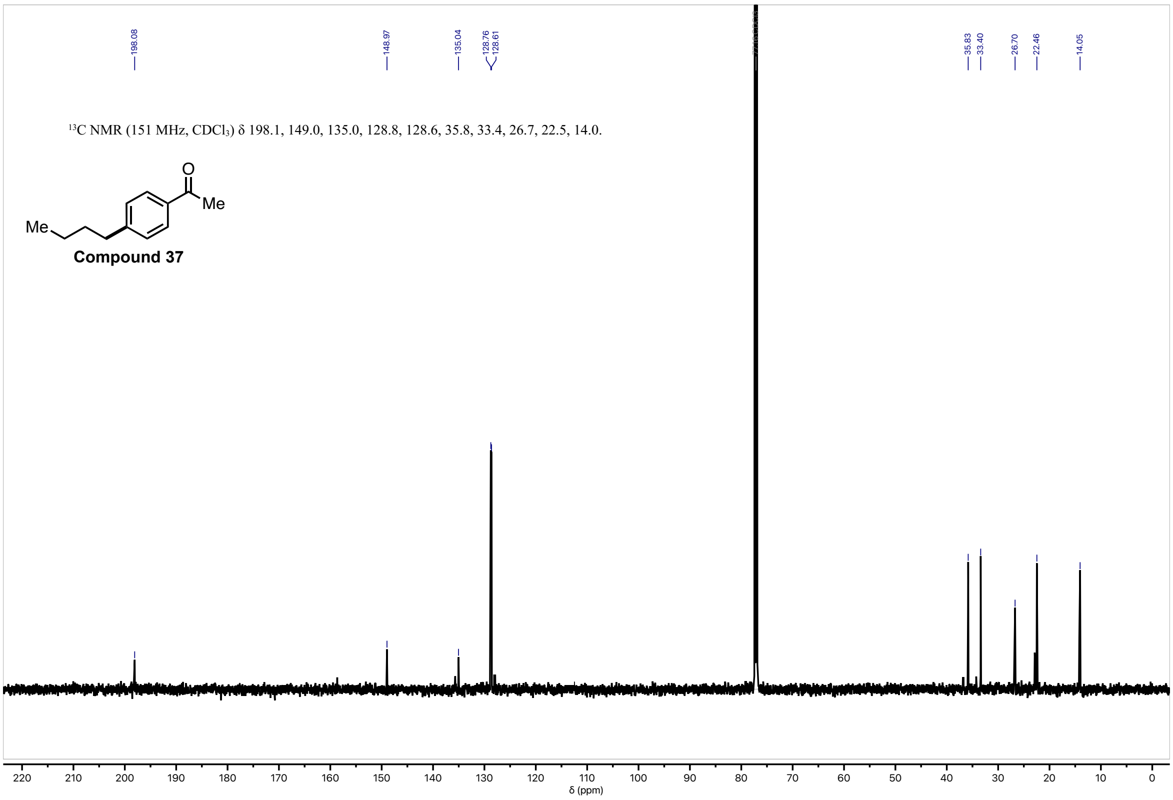
^{13}C NMR (151 MHz, CDCl$_3$) δ 198.1, 149.0, 135.0, 128.8, 128.6, 35.8, 33.4, 26.7, 22.5, 14.0.
O
Me
Me
Compound 37
δ (ppm)

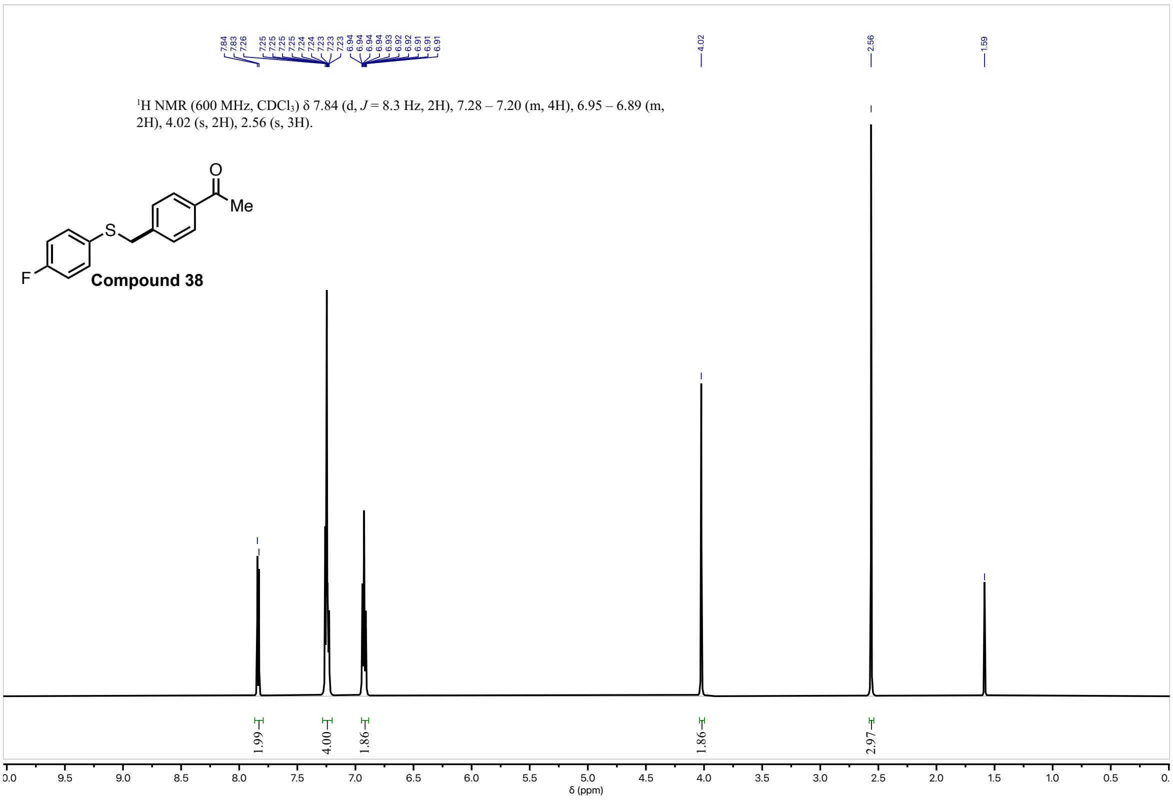
^{1}H NMR (600 MHz, CDCl$_3$) δ 7.84 (d, J = 8.3 Hz, 2H), 7.28 – 7.20 (m, 4H), 6.95 – 6.89 (m, 2H), 4.02 (s, 2H), 2.56 (s, 3H).
O
Me
S
F
Compound 38
δ (ppm)

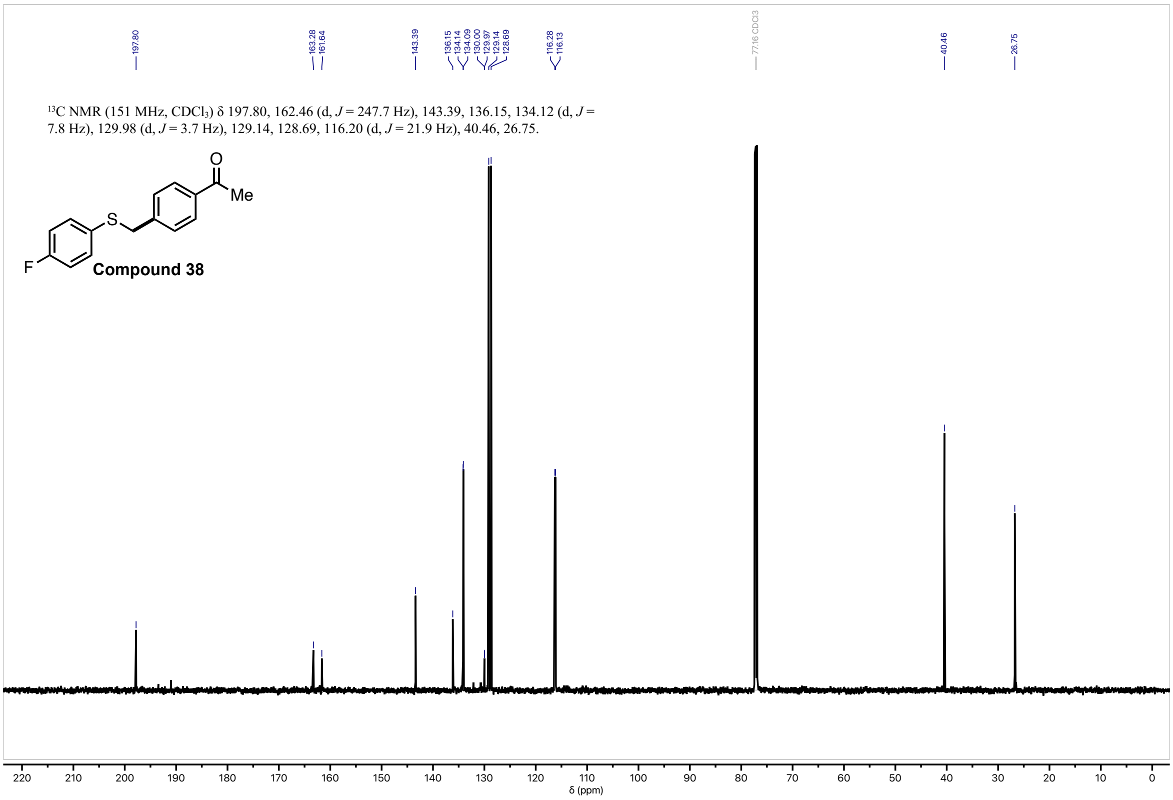

^{13}C NMR (151 MHz, $CDCl_3$) δ 197.80, 162.46 (d, J = 247.7 Hz), 143.39, 136.15, 134.12 (d, J = 7.8 Hz), 129.98 (d, J = 3.7 Hz), 129.14, 128.69, 116.20 (d, J = 21.9 Hz), 40.46, 26.75.

Compound 38

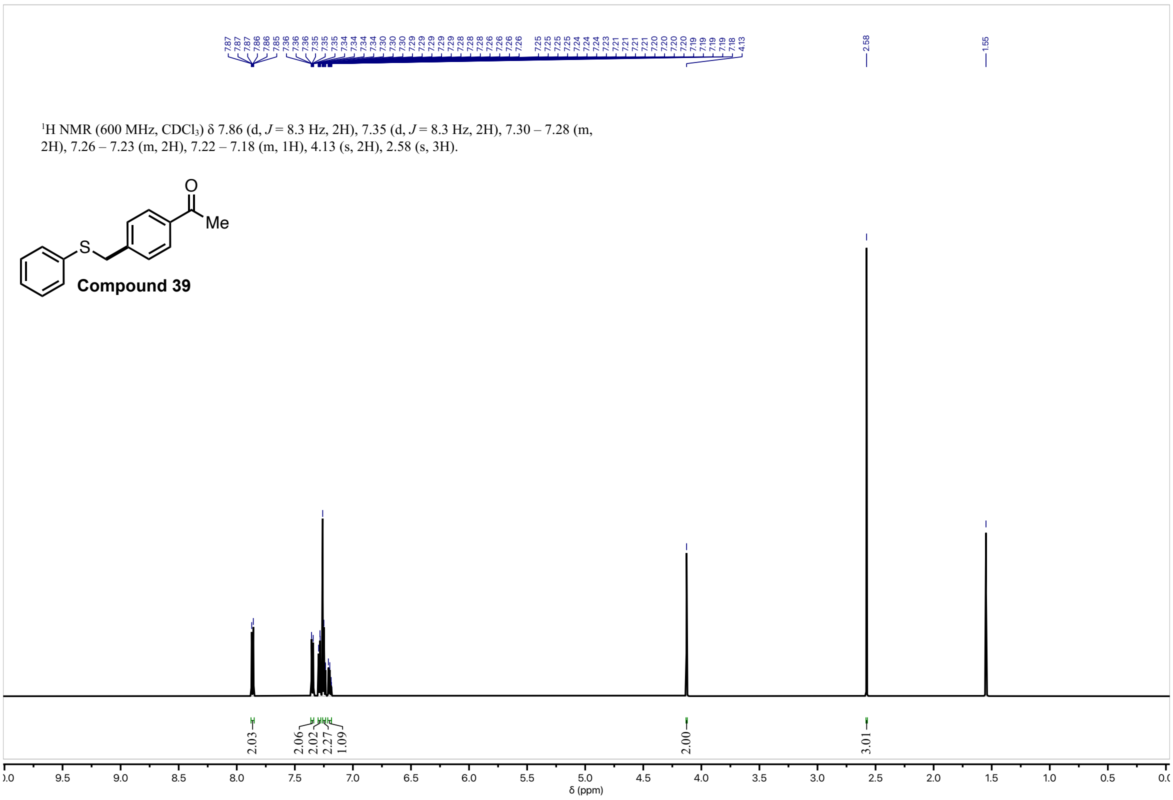

^{1}H NMR (600 MHz, $CDCl_3$) δ 7.86 (d, J = 8.3 Hz, 2H), 7.35 (d, J = 8.3 Hz, 2H), 7.30 – 7.28 (m, 2H), 7.26 – 7.23 (m, 2H), 7.22 – 7.18 (m, 1H), 4.13 (s, 2H), 2.58 (s, 3H).

Compound 39

^{13}C NMR (151 MHz, $CDCl_3$) δ 197.8, 143.4, 136.2, 135.5, 130.6, 129.1, 129.1, 128.7, 127.0, 39.2, 26.8.

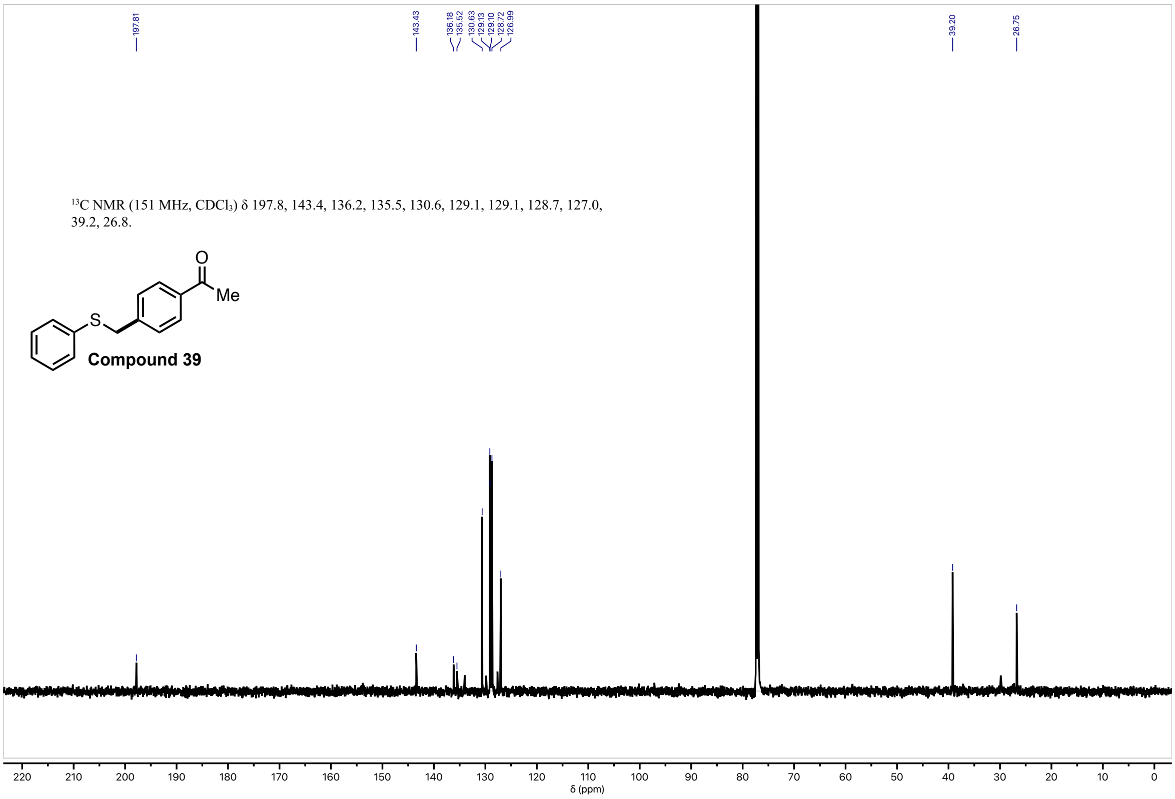

^{1}H NMR (600 MHz, $CDCl_3$) δ 8.11 – 8.07 (m, 2H), 8.00 – 7.96 (m, 2H), 7.60 – 7.56 (m, 1H), 7.56 – 7.52 (m, 2H), 7.48 – 7.44 (m, 2H), 5.42 (s, 2H), 2.61 (s, 3H).

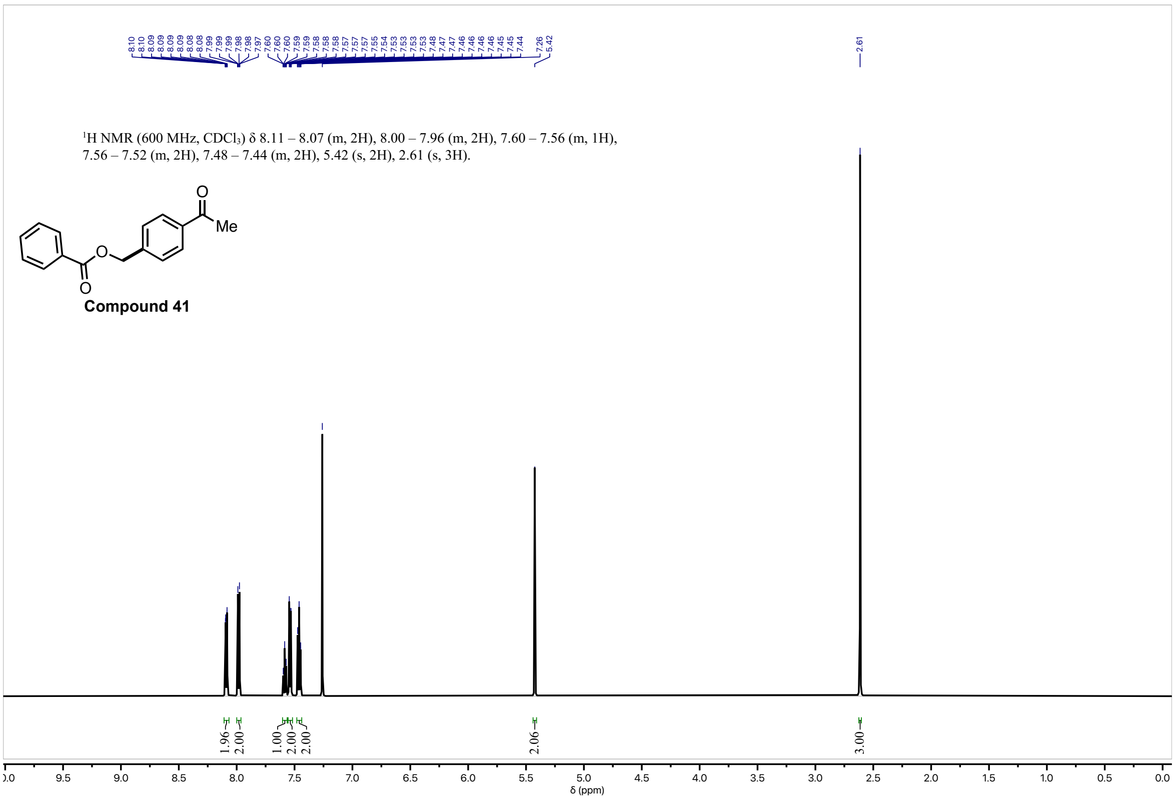

^{13}C NMR (151 MHz, $CDCl_3$) δ 197.8, 166.4, 141.5, 137.0, 133.4, 130.7, 129.9, 128.8, 128.6, 128.0, 66.0, 26.8.

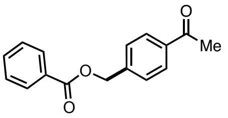

Compound 41

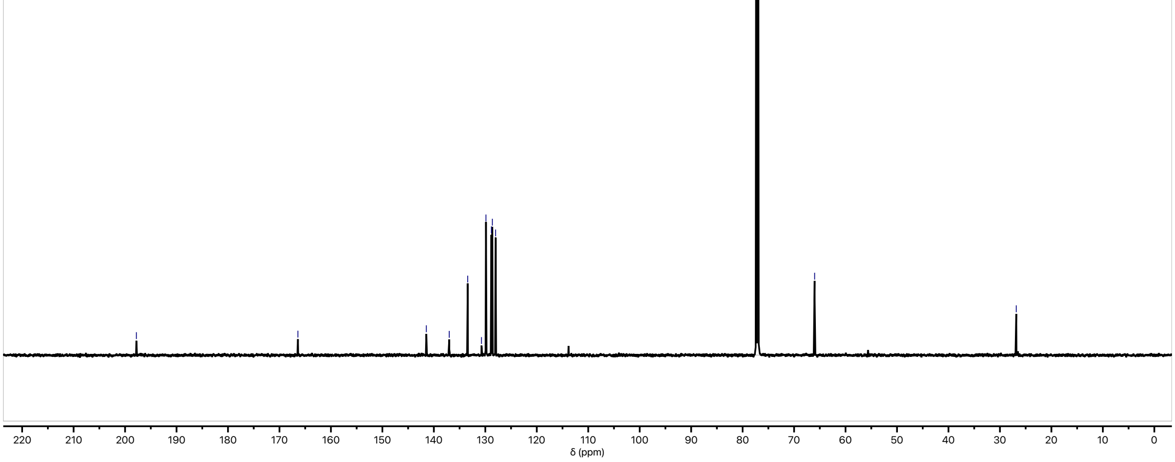

^{1}H NMR (600 MHz, $CDCl_3$) δ 8.00 – 7.96 (m, 2H), 7.85 (dd, J = 3.8, 1.3 Hz, 1H), 7.59 (dd, J = 5.0, 1.3 Hz, 1H), 7.54 – 7.50 (m, 2H), 7.12 (dd, J = 4.9, 3.7 Hz, 1H), 5.39 (s, 2H), 2.61 (s, 3H).

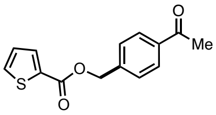

Compound 42

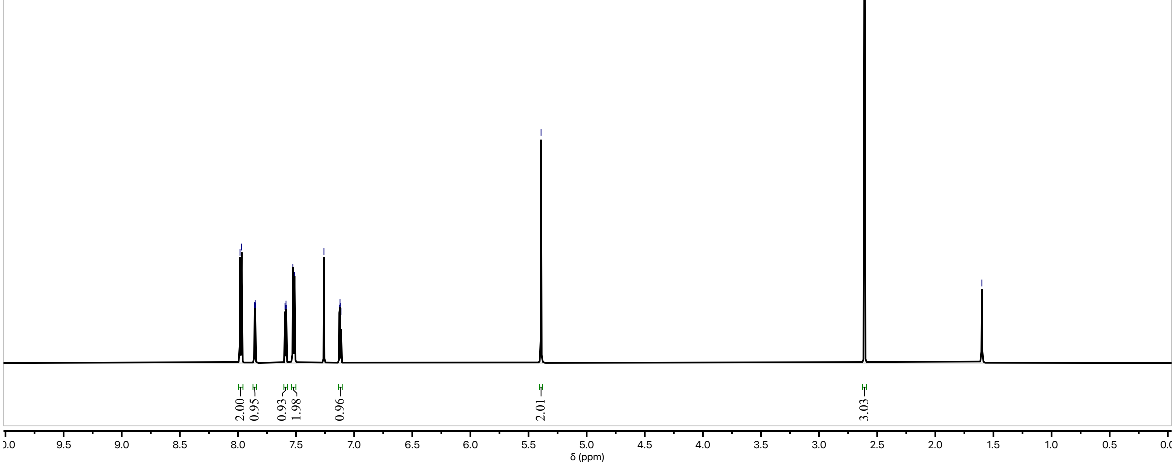

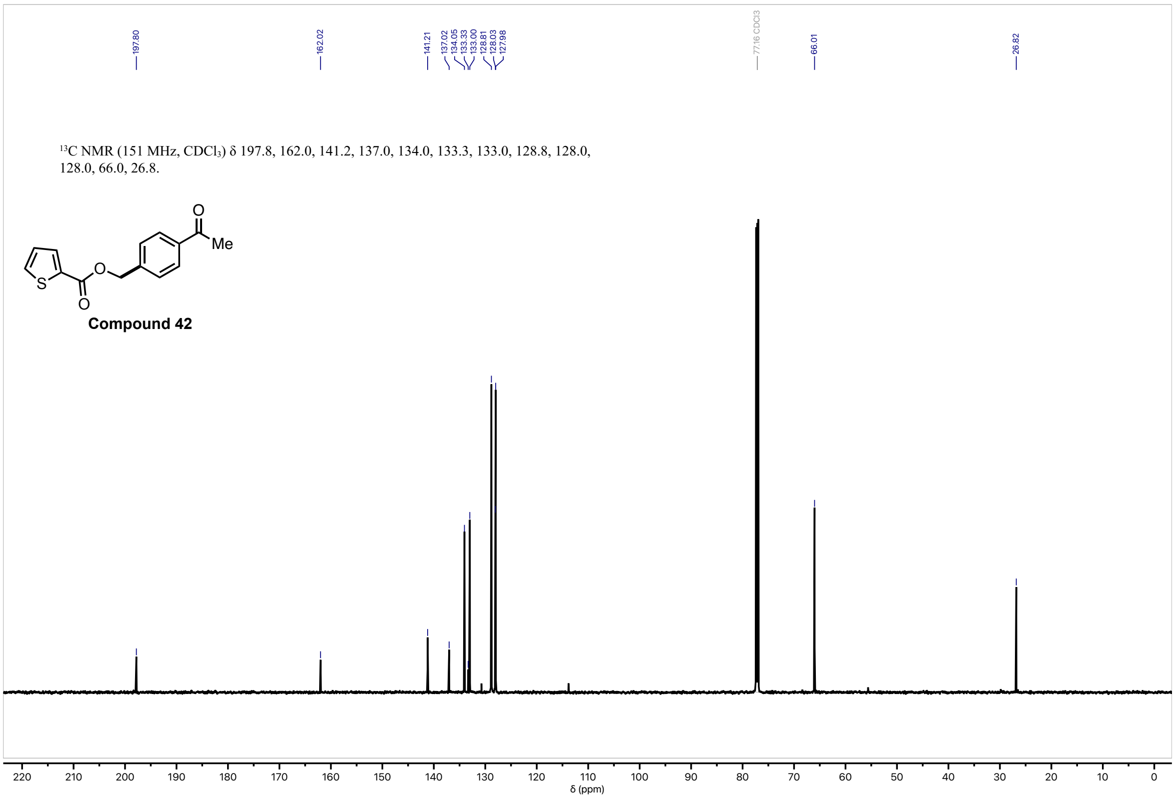
^{13}C NMR (151 MHz, CDCl$_3$) δ 197.8, 162.0, 141.2, 137.0, 134.0, 133.3, 133.0, 128.8, 128.0, 128.0, 66.0, 26.8.
Me
S
O
Compound 42
δ (ppm)

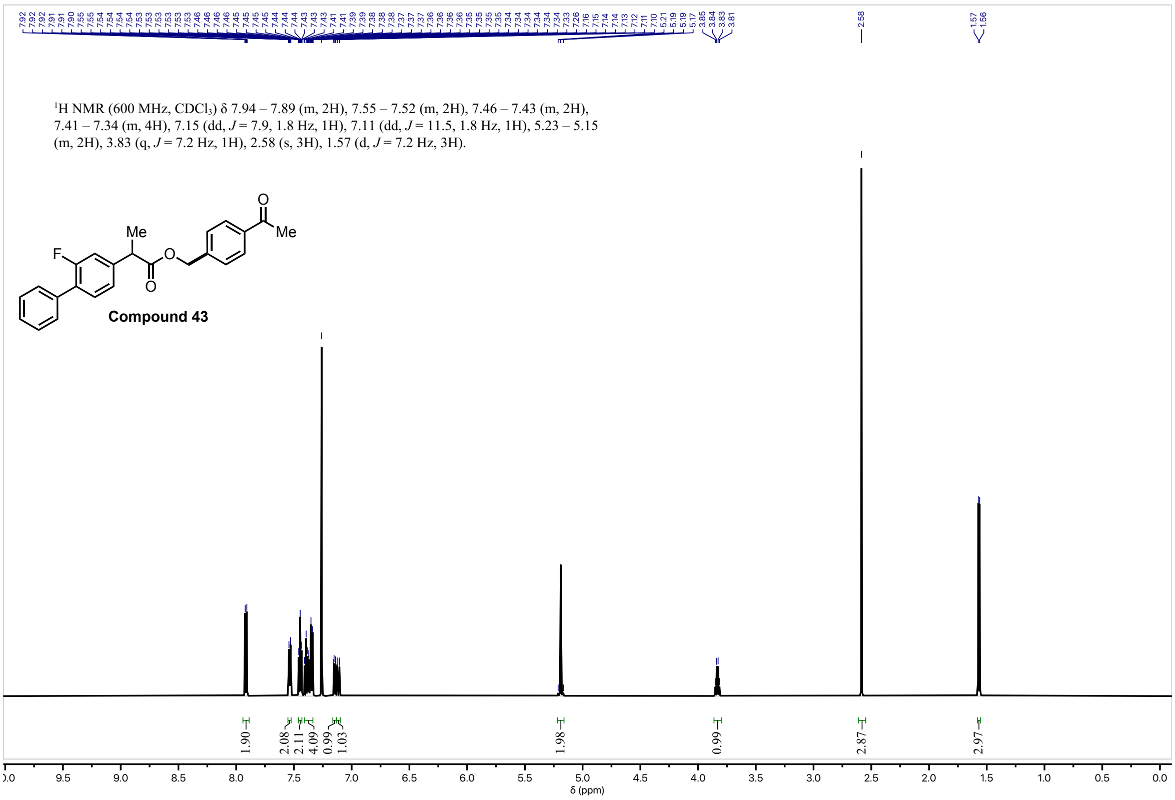
^{1}H NMR (600 MHz, CDCl$_3$) δ 7.94 – 7.89 (m, 2H), 7.55 – 7.52 (m, 2H), 7.46 – 7.43 (m, 2H), 7.41 – 7.34 (m, 4H), 7.15 (dd, J = 7.9, 1.8 Hz, 1H), 7.11 (dd, J = 11.5, 1.8 Hz, 1H), 5.23 – 5.15 (m, 2H), 3.83 (q, J = 7.2 Hz, 1H), 2.58 (s, 3H), 1.57 (d, J = 7.2 Hz, 3H).
F
Me
O
Compound 43
δ (ppm)

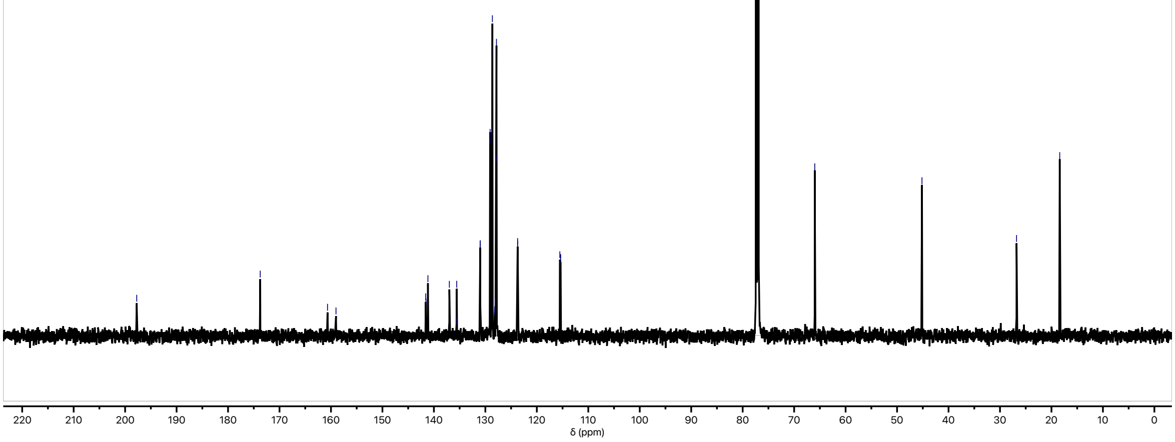

^{13}C NMR (151 MHz, $CDCl_3$) δ 197.7, 173.7, 159.8 (d, J = 248.2 Hz), 141.6 (d, J = 7.3 Hz), 141.2, 137.0, 135.6 (d, J = 7.4 Hz), 131.0 (d, J = 4.0 Hz), 129.1 (d, J = 2.9 Hz), 128.7, 128.6, 128.1 (d, J = 13.6 Hz), 127.9, 127.8, 123.7 (d, J = 3.5 Hz), 115.4 (d, J = 23.7 Hz), 66.0, 45.2, 26.8, 18.4.

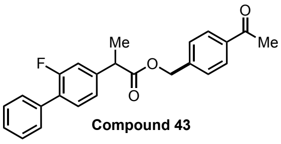

Compound 43

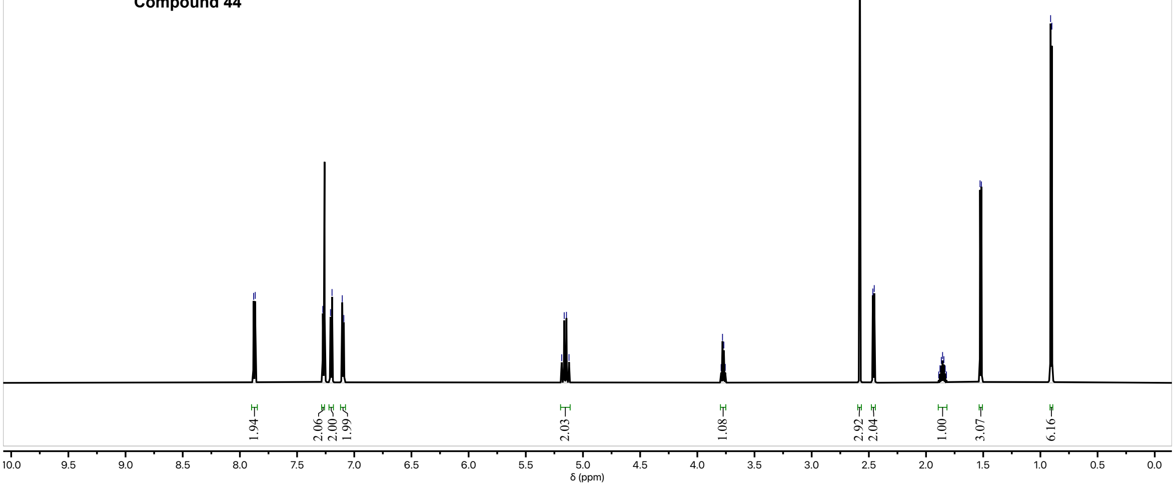

^{1}H NMR (600 MHz, $CDCl_3$) δ 7.90 – 7.85 (m, 2H), 7.29 – 7.26 (m, 2H), 7.22 – 7.18 (m, 2H), 7.12 – 7.08 (m, 2H), 5.20 – 5.11 (m, 2H), 3.77 (q, J = 7.2 Hz, 1H), 2.58 (s, 3H), 2.46 (d, J = 7.2 Hz, 2H), 1.86 (hept, J = 6.7 Hz, 1H), 1.52 (d, J = 7.1 Hz, 3H), 0.91 (d, J = 6.6 Hz, 6H).

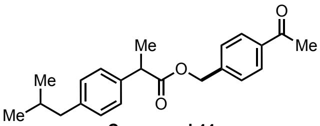

Compound 44

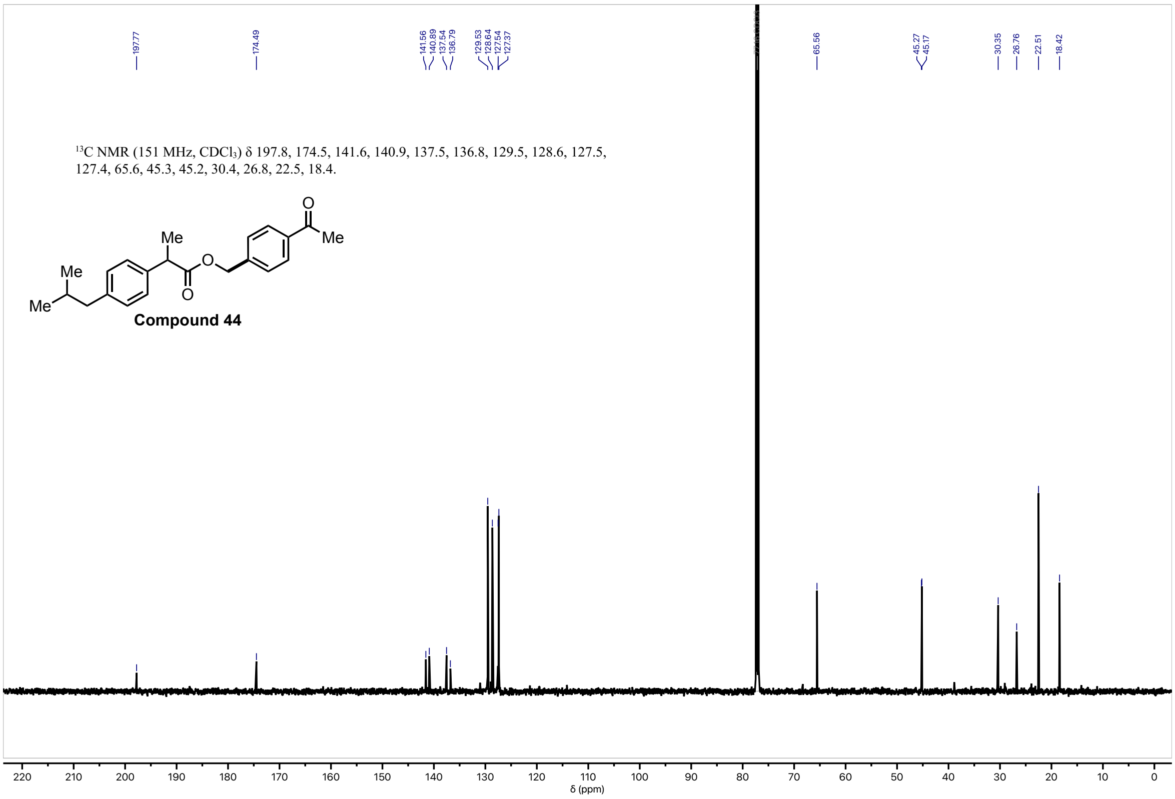

^{13}C NMR (151 MHz, $CDCl_3$) δ 197.8, 174.5, 141.6, 140.9, 137.5, 136.8, 129.5, 128.6, 127.5, 127.4, 65.6, 45.3, 45.2, 30.4, 26.8, 22.5, 18.4.

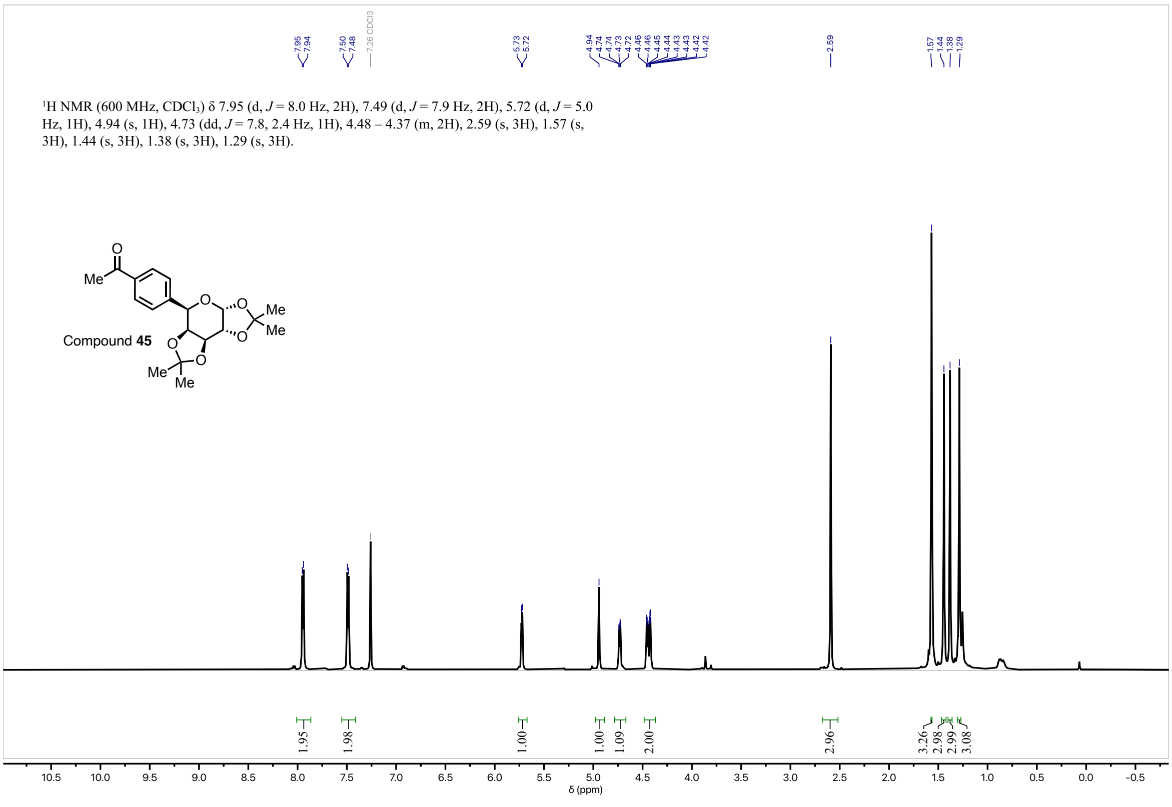

^{1}H NMR (600 MHz, $CDCl_3$) δ 7.95 (d, J = 8.0 Hz, 2H), 7.49 (d, J = 7.9 Hz, 2H), 5.72 (d, J = 5.0 Hz, 1H), 4.94 (s, 1H), 4.73 (dd, J = 7.8, 2.4 Hz, 1H), 4.48 – 4.37 (m, 2H), 2.59 (s, 3H), 1.57 (s, 3H), 1.44 (s, 3H), 1.38 (s, 3H), 1.29 (s, 3H).

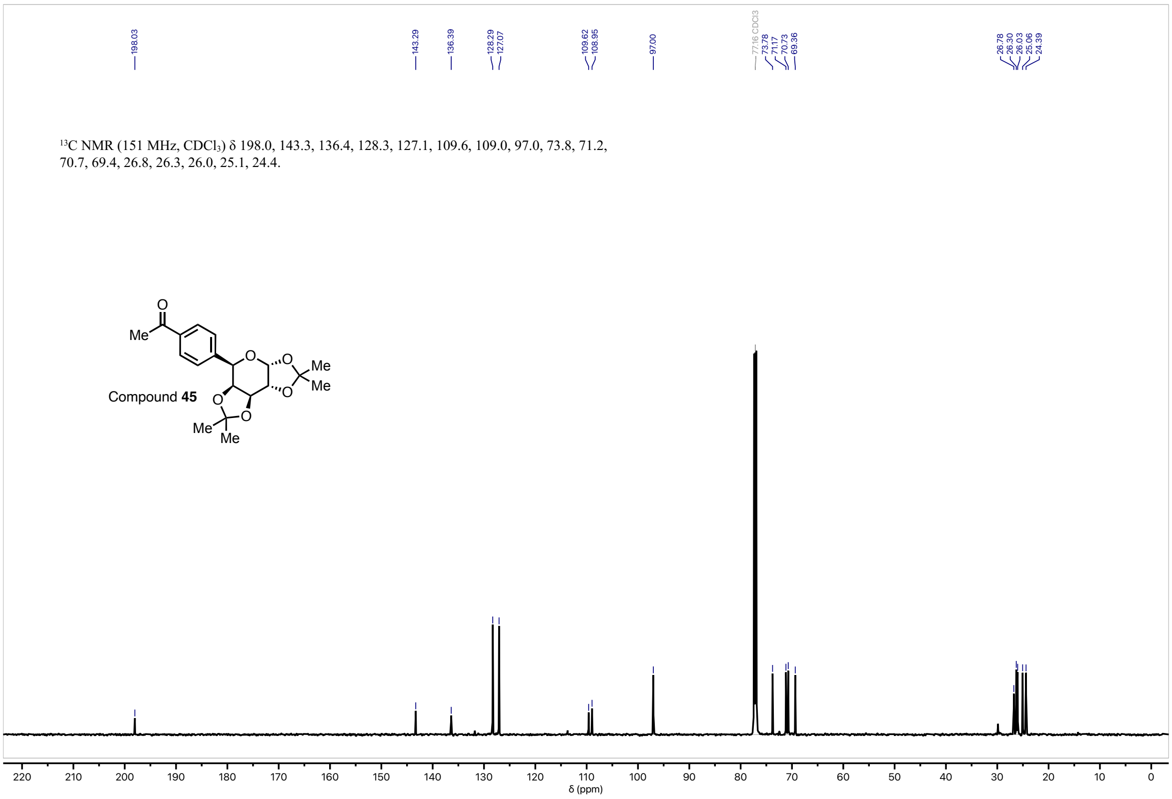

^{13}C NMR (151 MHz, $CDCl_3$) δ 198.0, 143.3, 136.4, 128.3, 127.1, 109.6, 109.0, 97.0, 73.8, 71.2, 70.7, 69.4, 26.8, 26.3, 26.0, 25.1, 24.4.

Compound **45**

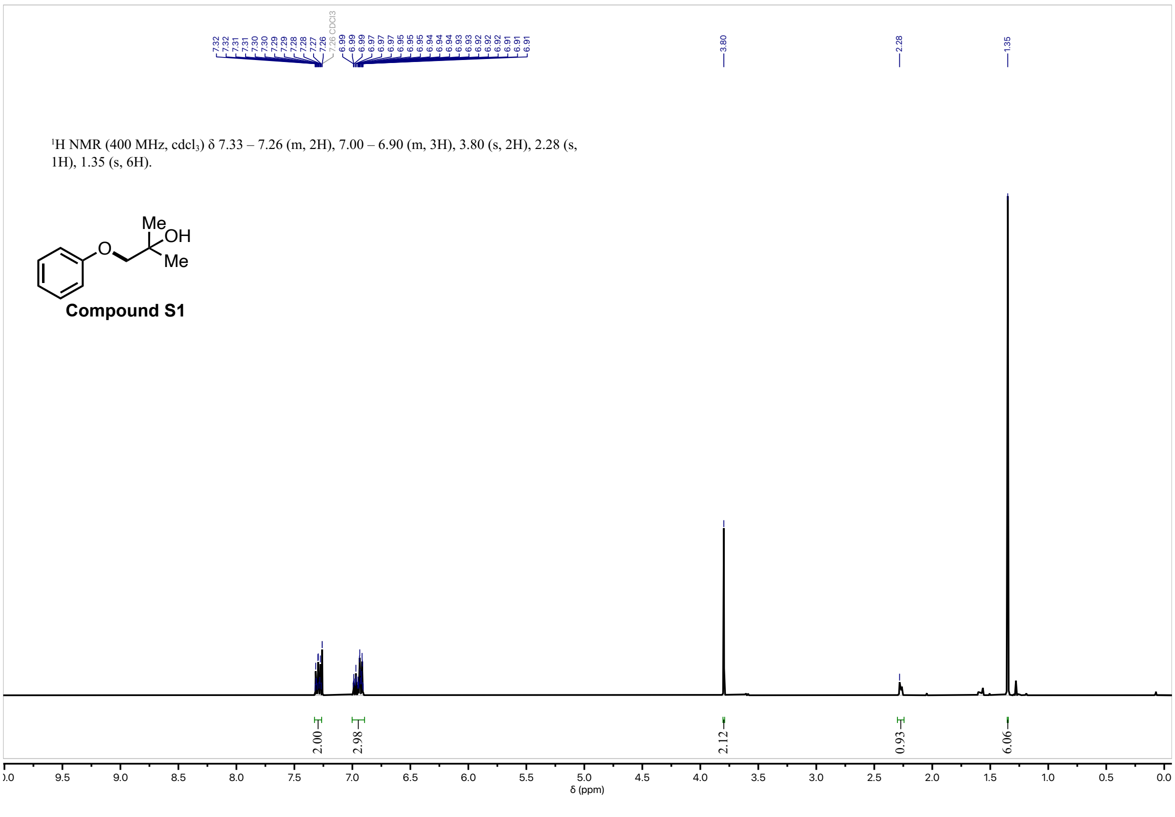

^{1}H NMR (400 MHz, cdcl$_3$) δ 7.33 – 7.26 (m, 2H), 7.00 – 6.90 (m, 3H), 3.80 (s, 2H), 2.28 (s, 1H), 1.35 (s, 6H).

Compound S1

^{1}H NMR (600 MHz, $CDCl_3$) δ 7.29 (d, *J* = 8.8 Hz, 2H), 6.77 (d, *J* = 8.9 Hz, 2H), 3.74 (s, 3H), 1.51 (s, 3H), 1.50 (s, 1H), 0.85 (s, 9H).

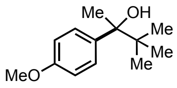

Compound S2

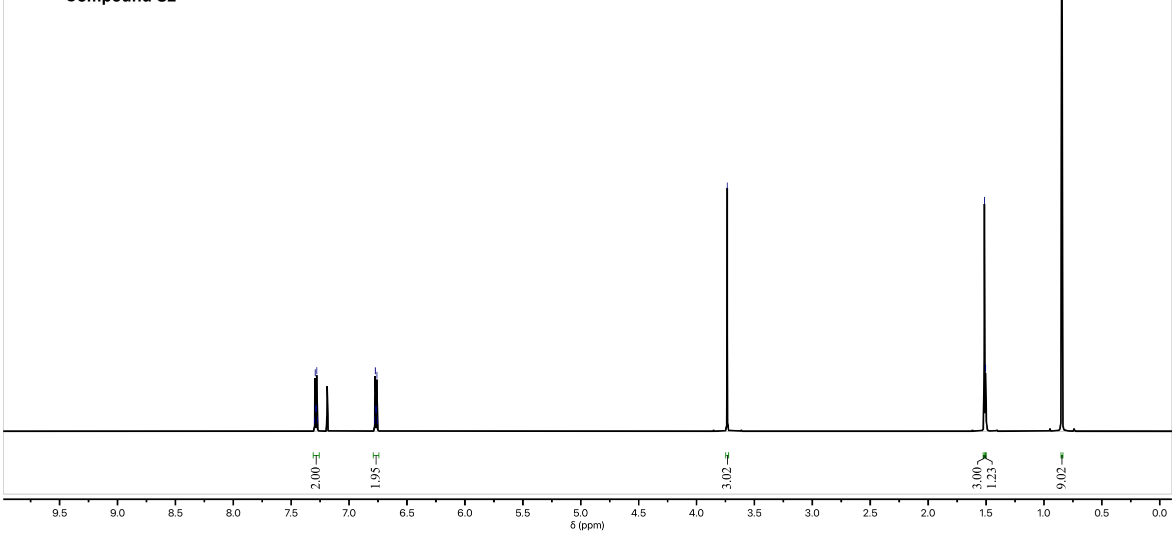

^{1}H NMR (600 MHz, $CDCl_3$) δ 7.00 – 6.95 (m, 2H), 6.88 – 6.84 (m, 2H), 3.75 (s, 2H), 2.22 (s, 1H), 1.34 (s, 6H).

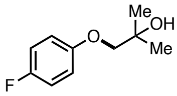

Compound S3

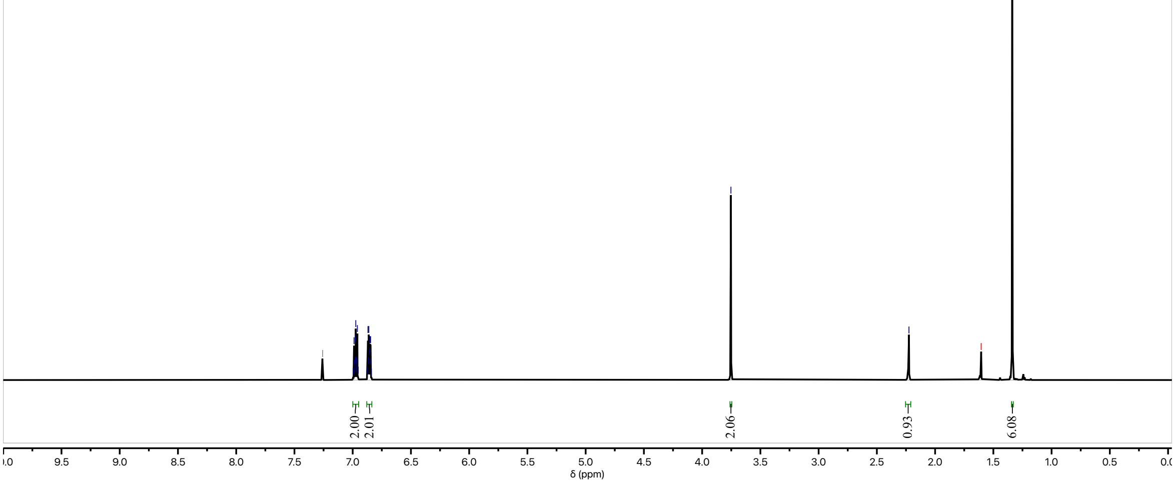

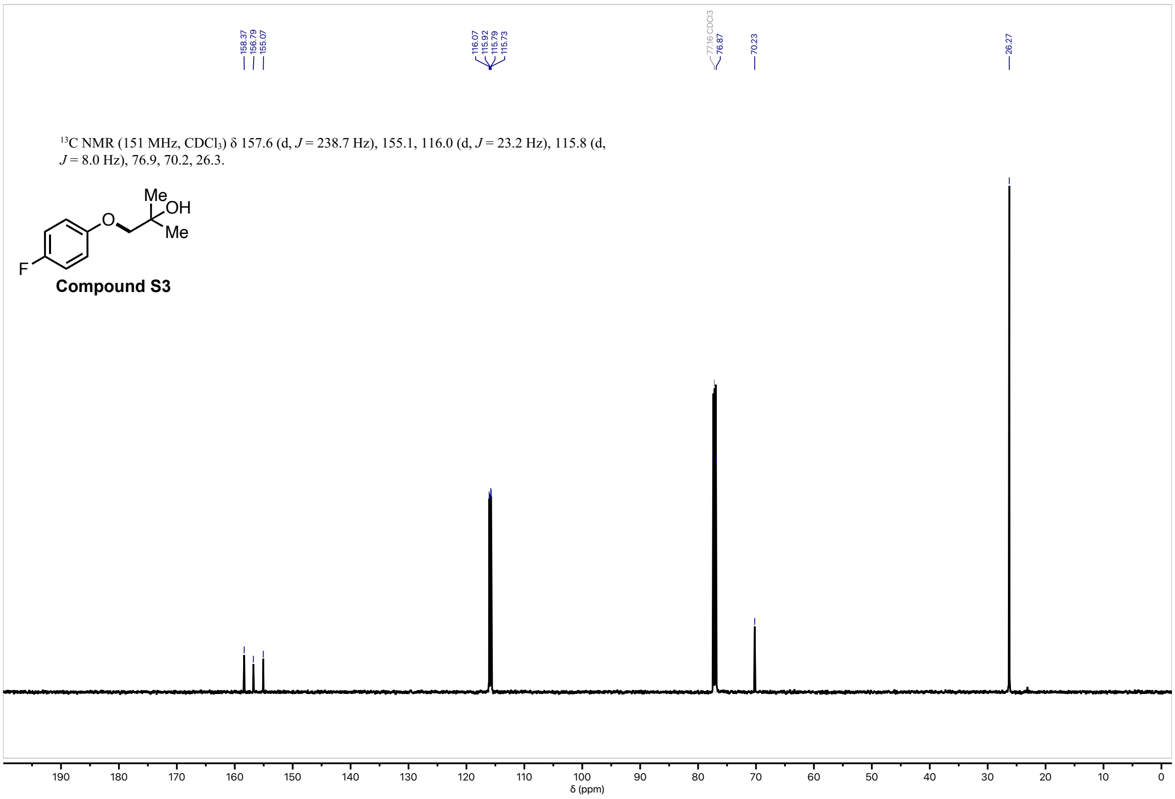
^{13}C NMR (151 MHz, CDCl$_3$) δ 157.6 (d, J = 238.7 Hz), 155.1, 116.0 (d, J = 23.2 Hz), 115.8 (d, J = 8.0 Hz), 76.9, 70.2, 26.3.
Compound S3
δ (ppm)

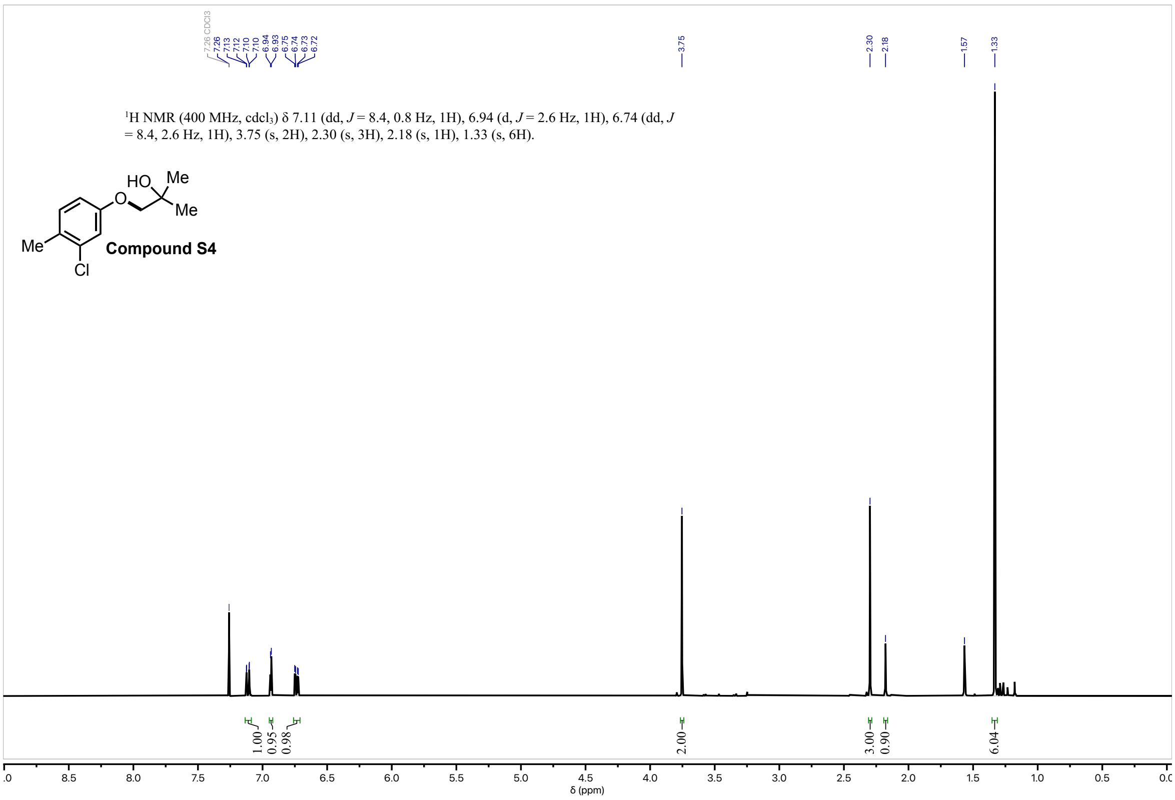
^{1}H NMR (400 MHz, cdcl$_3$) δ 7.11 (dd, J = 8.4, 0.8 Hz, 1H), 6.94 (d, J = 2.6 Hz, 1H), 6.74 (dd, J = 8.4, 2.6 Hz, 1H), 3.75 (s, 2H), 2.30 (s, 3H), 2.18 (s, 1H), 1.33 (s, 6H).
Compound S4
δ (ppm)

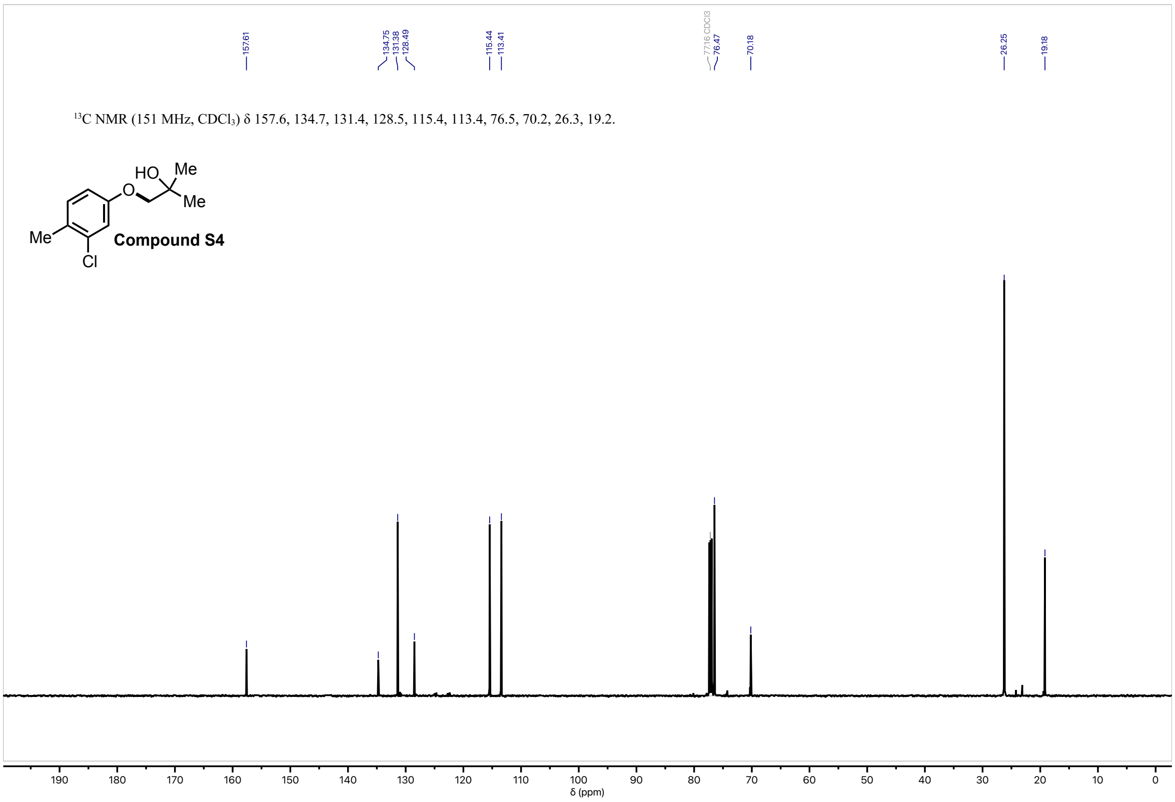

^{13}C NMR (151 MHz, $CDCl_3$) δ 157.6, 134.7, 131.4, 128.5, 115.4, 113.4, 76.5, 70.2, 26.3, 19.2.
Compound S4
δ (ppm)

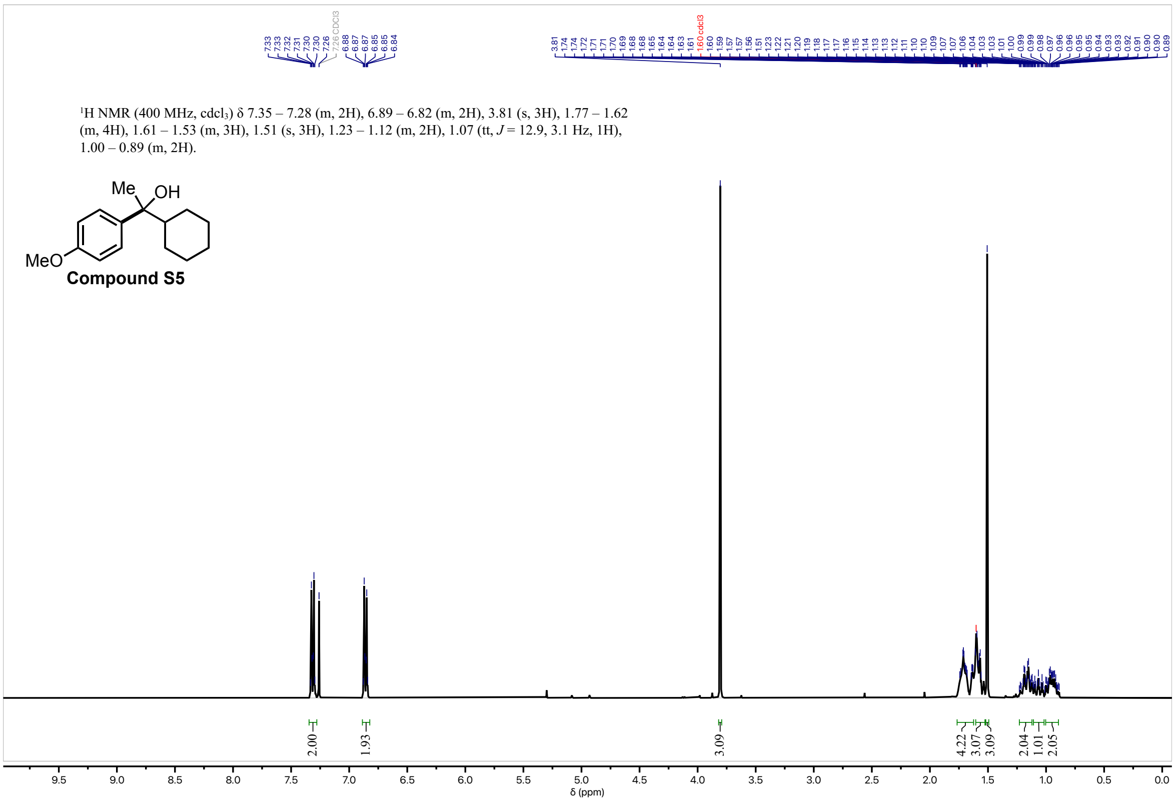

^1H NMR (400 MHz, cdcl$_3$) δ 7.35 – 7.28 (m, 2H), 6.89 – 6.82 (m, 2H), 3.81 (s, 3H), 1.77 – 1.62 (m, 4H), 1.61 – 1.53 (m, 3H), 1.51 (s, 3H), 1.23 – 1.12 (m, 2H), 1.07 (tt, J = 12.9, 3.1 Hz, 1H), 1.00 – 0.89 (m, 2H).
Compound S5
δ (ppm)

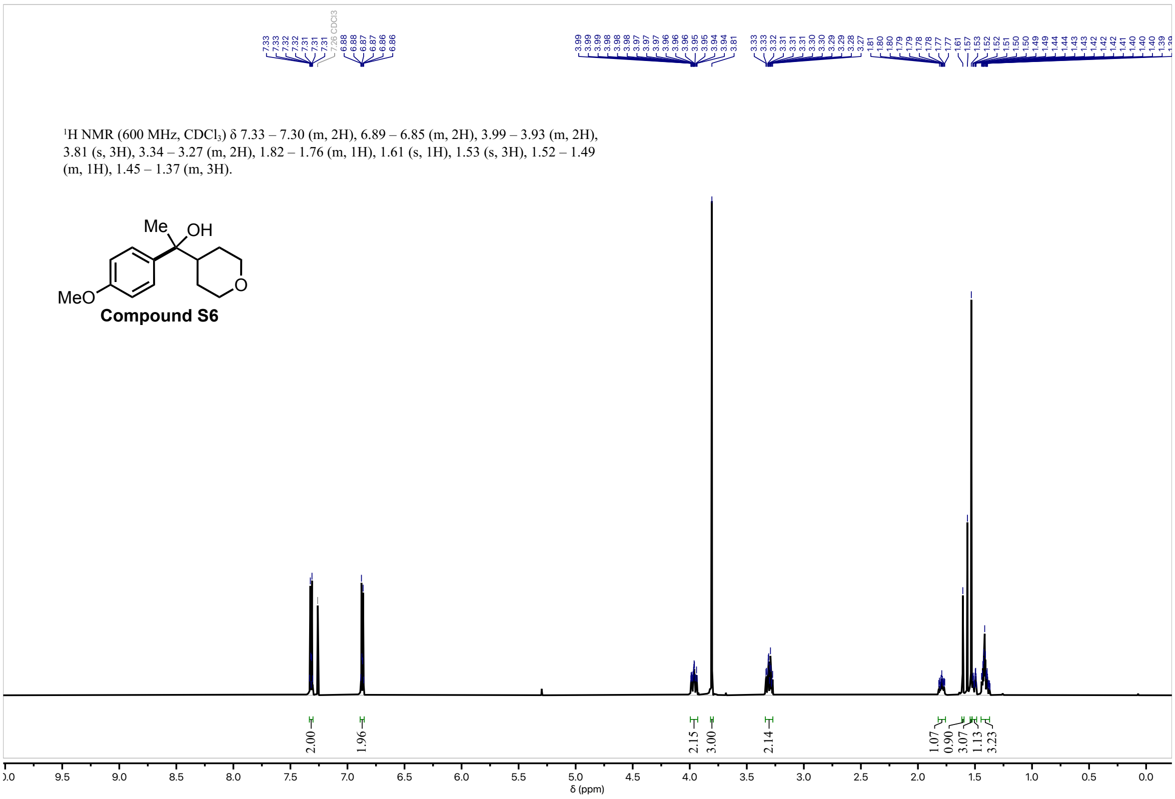

^{1}H NMR (600 MHz, $CDCl_3$) δ 7.33 – 7.30 (m, 2H), 6.89 – 6.85 (m, 2H), 3.99 – 3.93 (m, 2H), 3.81 (s, 3H), 3.34 – 3.27 (m, 2H), 1.82 – 1.76 (m, 1H), 1.61 (s, 1H), 1.53 (s, 3H), 1.52 – 1.49 (m, 1H), 1.45 – 1.37 (m, 3H).

Compound S6

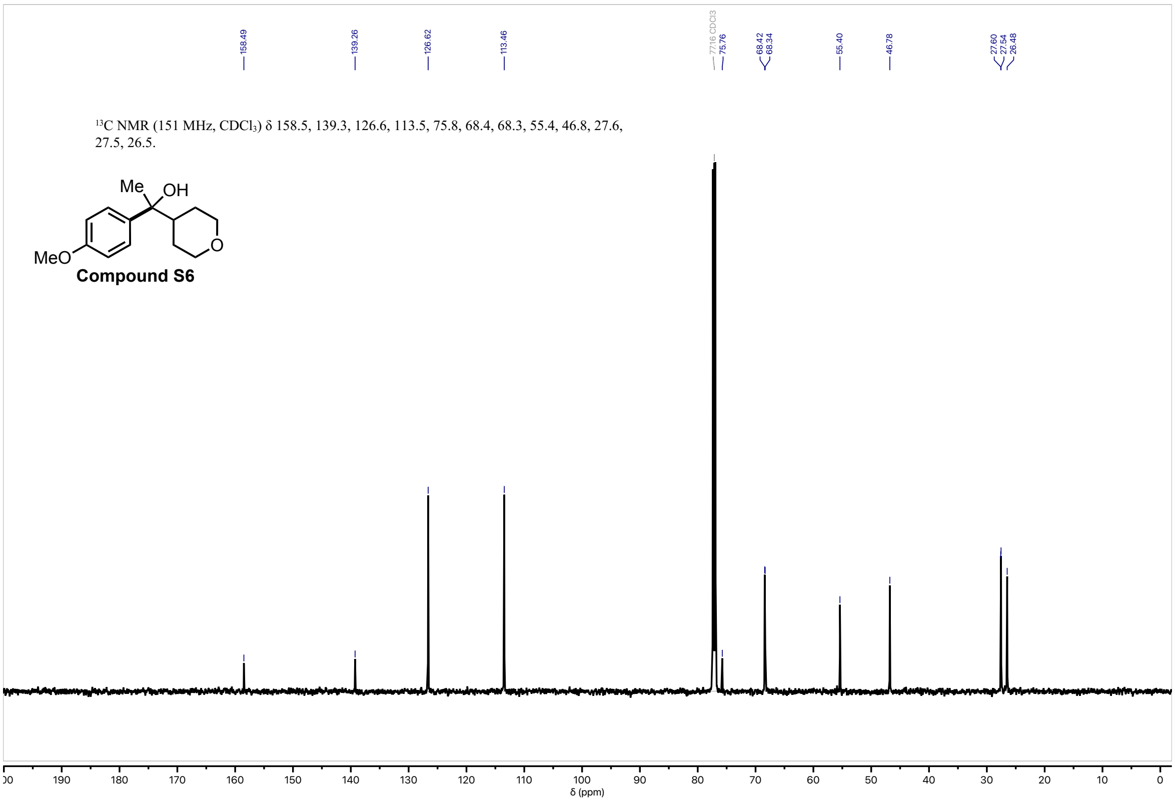

^{13}C NMR (151 MHz, $CDCl_3$) δ 158.5, 139.3, 126.6, 113.5, 75.8, 68.4, 68.3, 55.4, 46.8, 27.6, 27.5, 26.5.

Compound S6

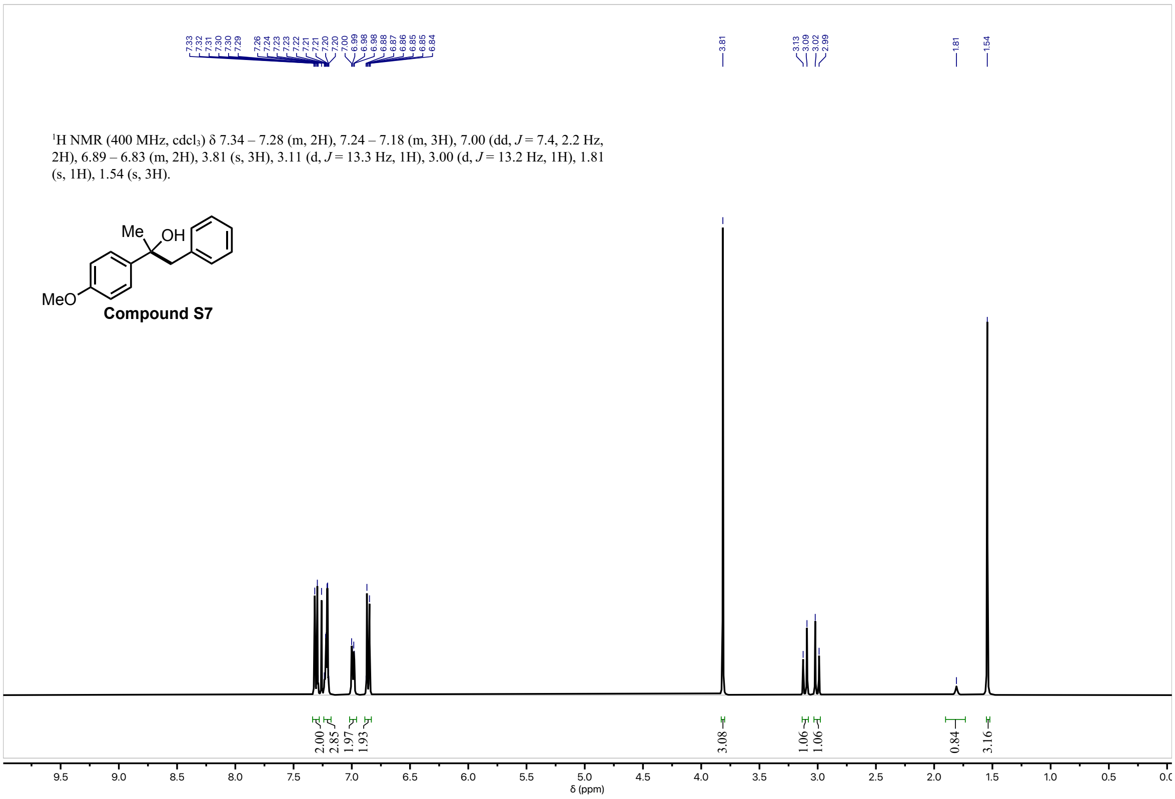

^{1}H NMR (400 MHz, cdcl$_3$) δ 7.34 – 7.28 (m, 2H), 7.24 – 7.18 (m, 3H), 7.00 (dd, J = 7.4, 2.2 Hz, 2H), 6.89 – 6.83 (m, 2H), 3.81 (s, 3H), 3.11 (d, J = 13.3 Hz, 1H), 3.00 (d, J = 13.2 Hz, 1H), 1.81 (s, 1H), 1.54 (s, 3H).
Me
OH
MeO
Compound S7
δ (ppm)

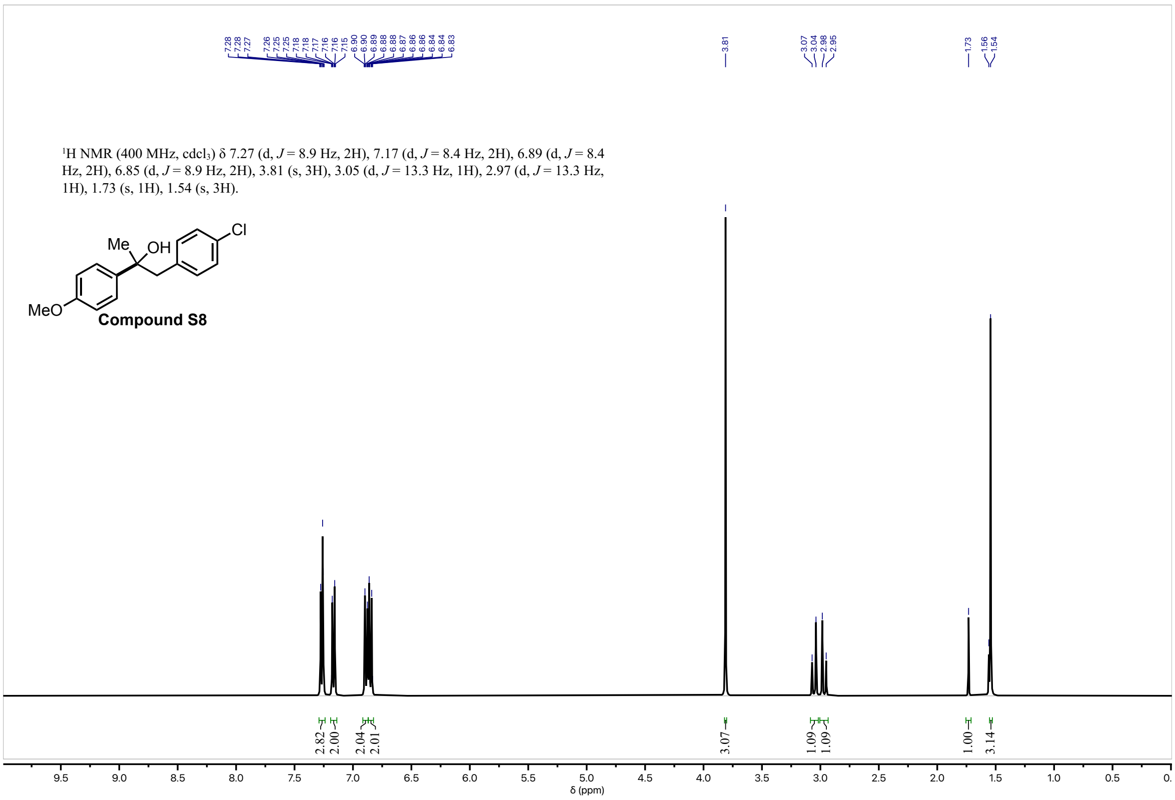

^{1}H NMR (400 MHz, cdcl$_3$) δ 7.27 (d, J = 8.9 Hz, 2H), 7.17 (d, J = 8.4 Hz, 2H), 6.89 (d, J = 8.4 Hz, 2H), 6.85 (d, J = 8.9 Hz, 2H), 3.81 (s, 3H), 3.05 (d, J = 13.3 Hz, 1H), 2.97 (d, J = 13.3 Hz, 1H), 1.73 (s, 1H), 1.54 (s, 3H).
Me
OH
Cl
MeO
Compound S8
δ (ppm)

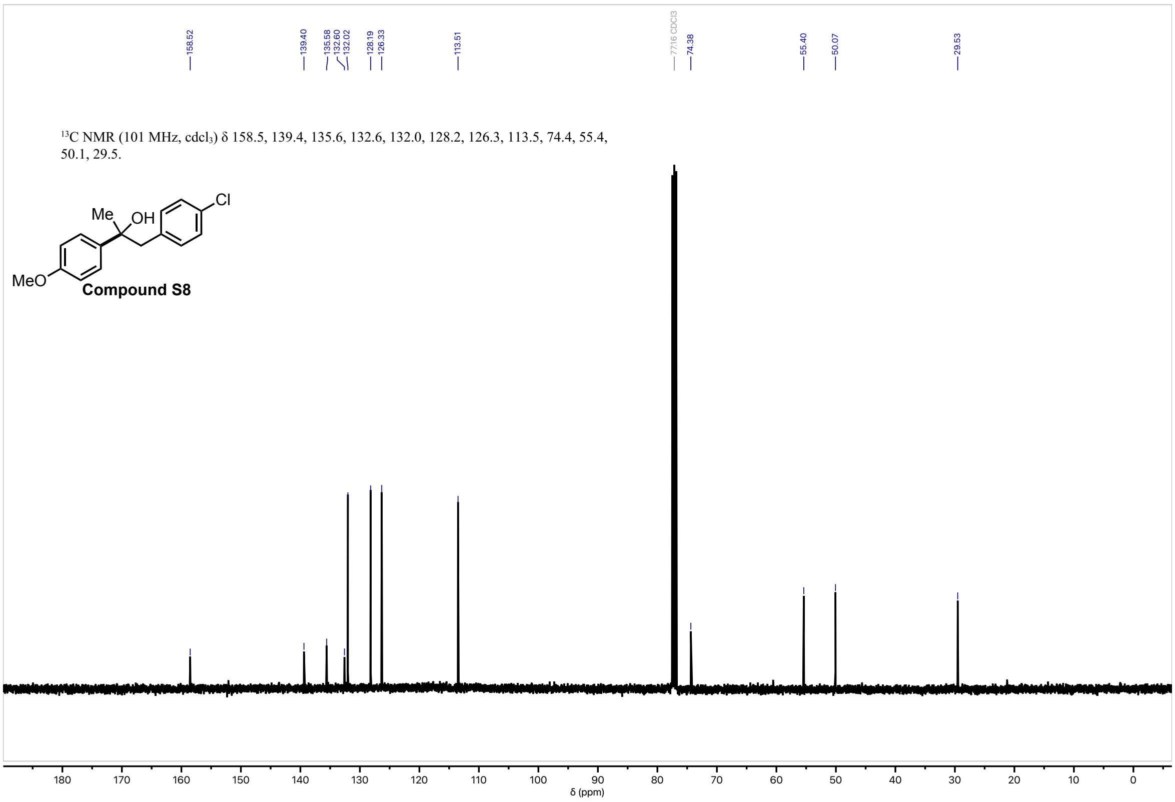

¹³C NMR (101 MHz, cdcl₃) δ 158.5, 139.4, 135.6, 132.6, 132.0, 128.2, 126.3, 113.5, 74.4, 55.4, 50.1, 29.5.
Me
OH
Cl
MeO
Compound S8
δ (ppm)

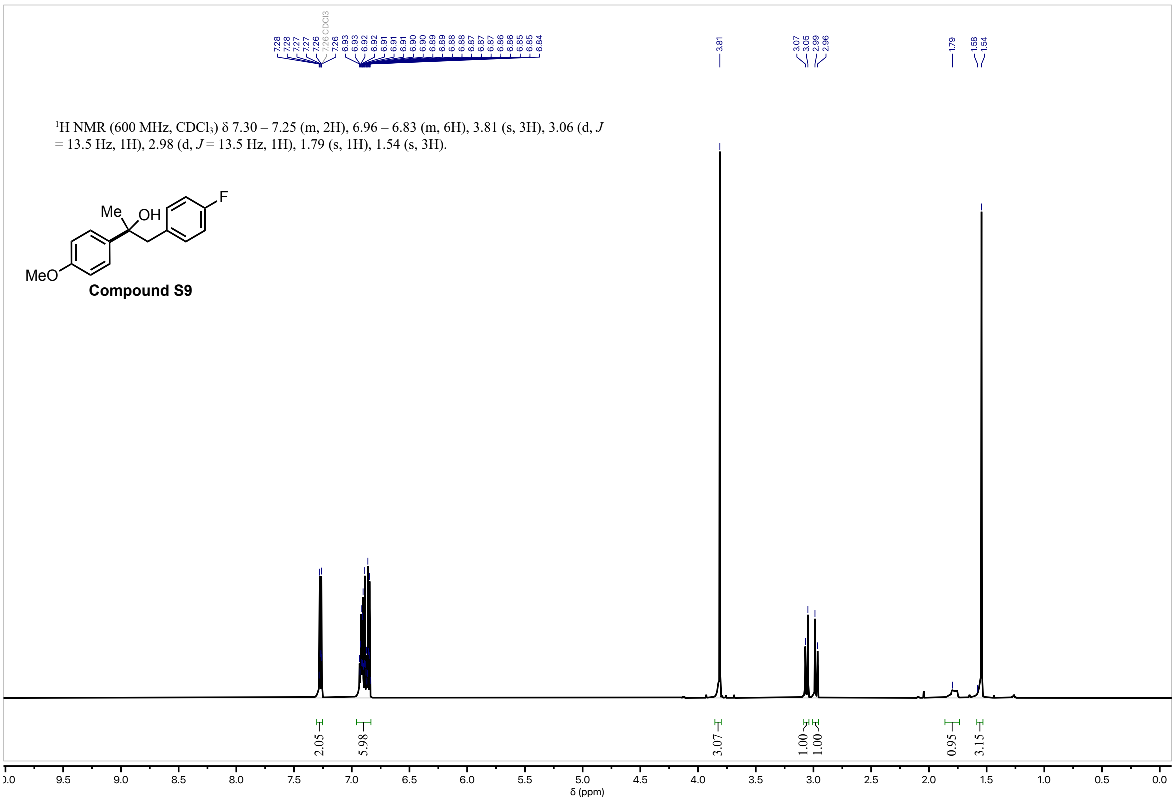

¹H NMR (600 MHz, CDCl₃) δ 7.30 – 7.25 (m, 2H), 6.96 – 6.83 (m, 6H), 3.81 (s, 3H), 3.06 (d, J = 13.5 Hz, 1H), 2.98 (d, J = 13.5 Hz, 1H), 1.79 (s, 1H), 1.54 (s, 3H).
Me
OH
F
MeO
Compound S9
δ (ppm)

^{13}C NMR (151 MHz, $CDCl_3$) δ 161.9 (d, J = 244.6 Hz), 158.5, 139.6, 132.8, 132.1 (d, J = 7.8 Hz), 126.3, 114.9 (d, J = 21.0 Hz), 113.5, 74.4, 55.4, 49.9, 29.5.

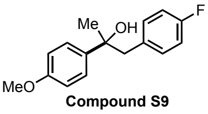

Compound S9

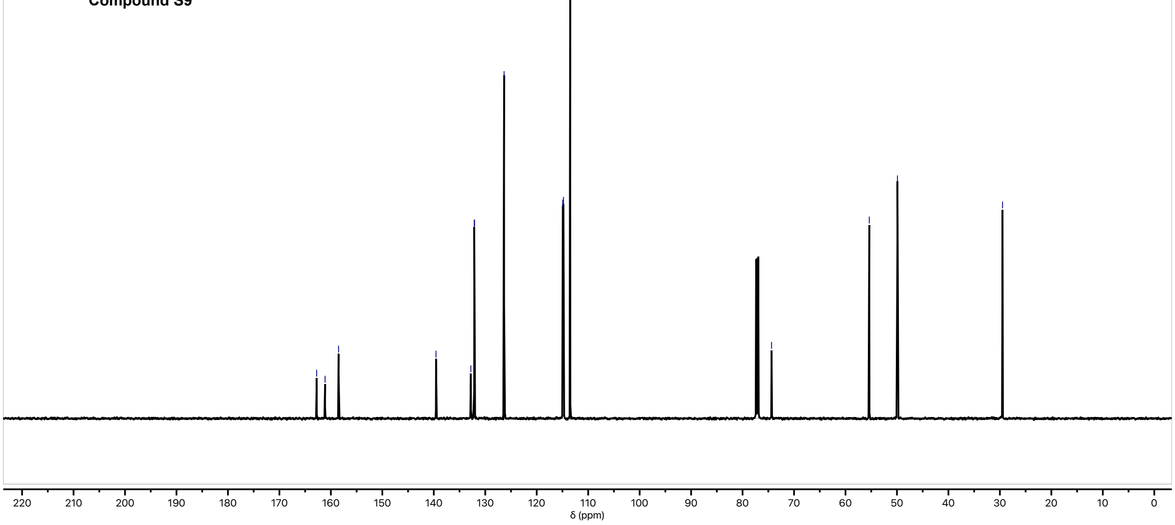

^{1}H NMR (600 MHz, $CDCl_3$) δ 7.33 – 7.29 (m, 2H), 7.05 – 7.01 (m, 2H), 6.90 – 6.84 (m, 4H), 3.81 (s, 3H), 3.07 (d, J = 13.3 Hz, 1H), 2.96 (d, J = 13.3 Hz, 1H), 2.30 (s, 3H), 1.81 (s, 1H), 1.53 (s, 3H).

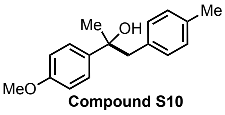

Compound S10

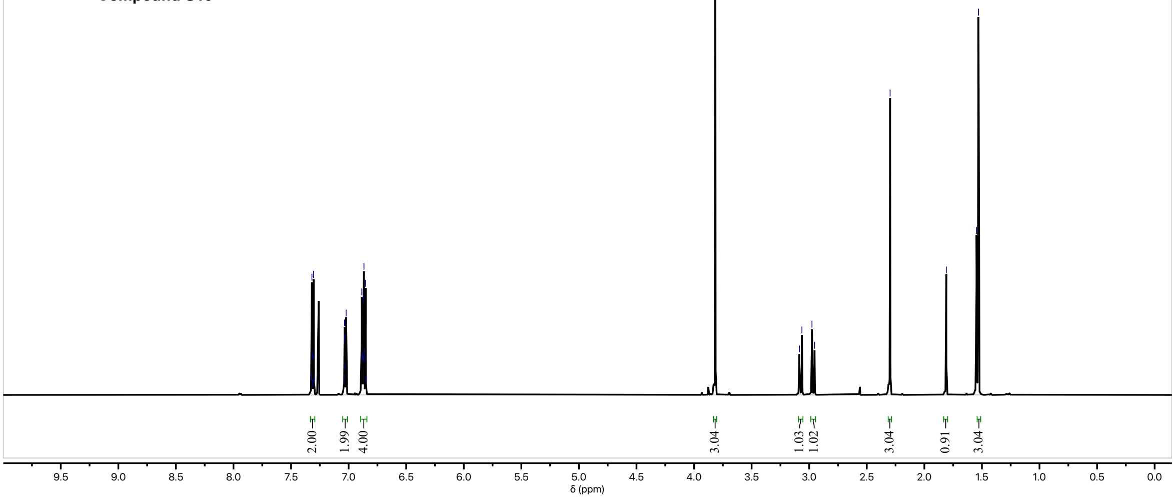

^{13}C NMR (151 MHz, $CDCl_3$) δ 158.4, 140.1, 136.3, 133.8, 130.6, 128.9, 126.3, 113.5, 74.3, 55.4, 50.3, 29.6, 21.2.

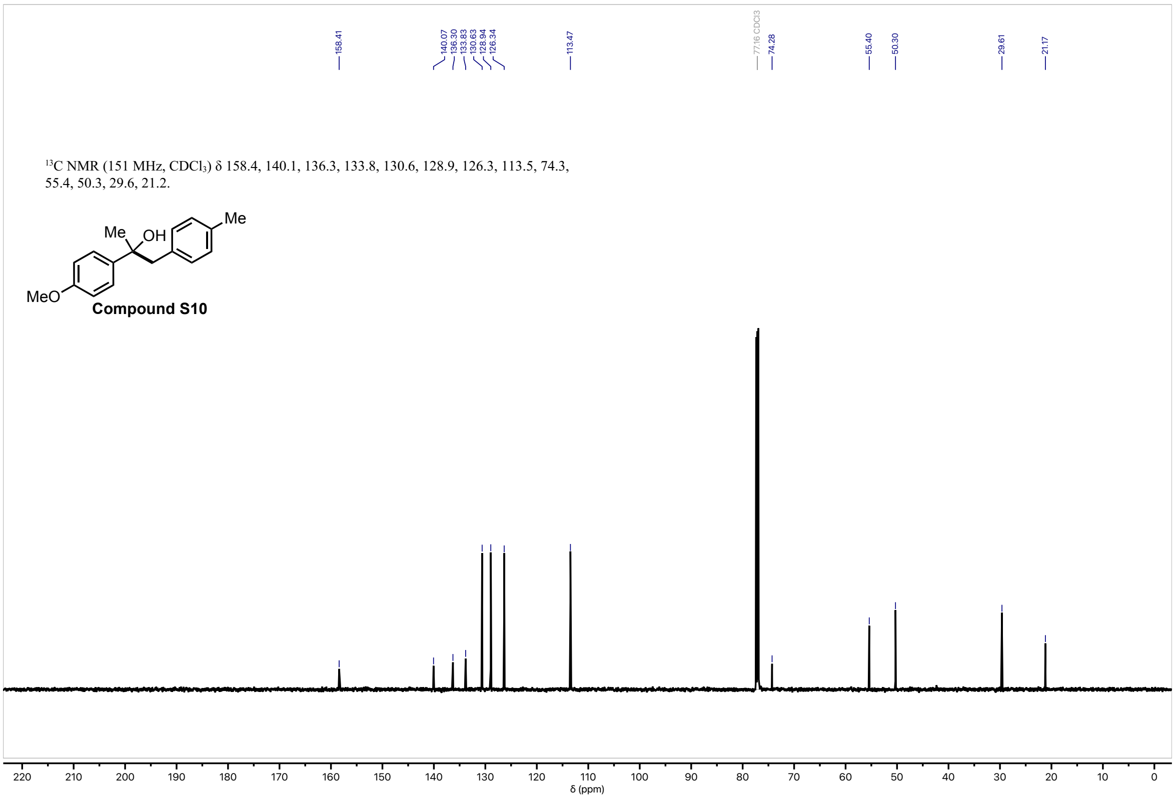

^{1}H NMR (400 MHz, cdcl$_3$) δ 7.81 – 7.72 (m, 2H), 7.66 (d, *J* = 8.4 Hz, 1H), 7.54 – 7.50 (m, 1H), 7.48 – 7.40 (m, 2H), 7.36 – 7.30 (m, 2H), 7.04 (dd, *J* = 8.4, 1.7 Hz, 1H), 6.89 – 6.84 (m, 2H), 3.82 (s, 3H), 3.27 (d, *J* = 13.2 Hz, 1H), 3.17 (d, *J* = 13.3 Hz, 1H), 1.88 (s, 1H), 1.55 (s, 3H).

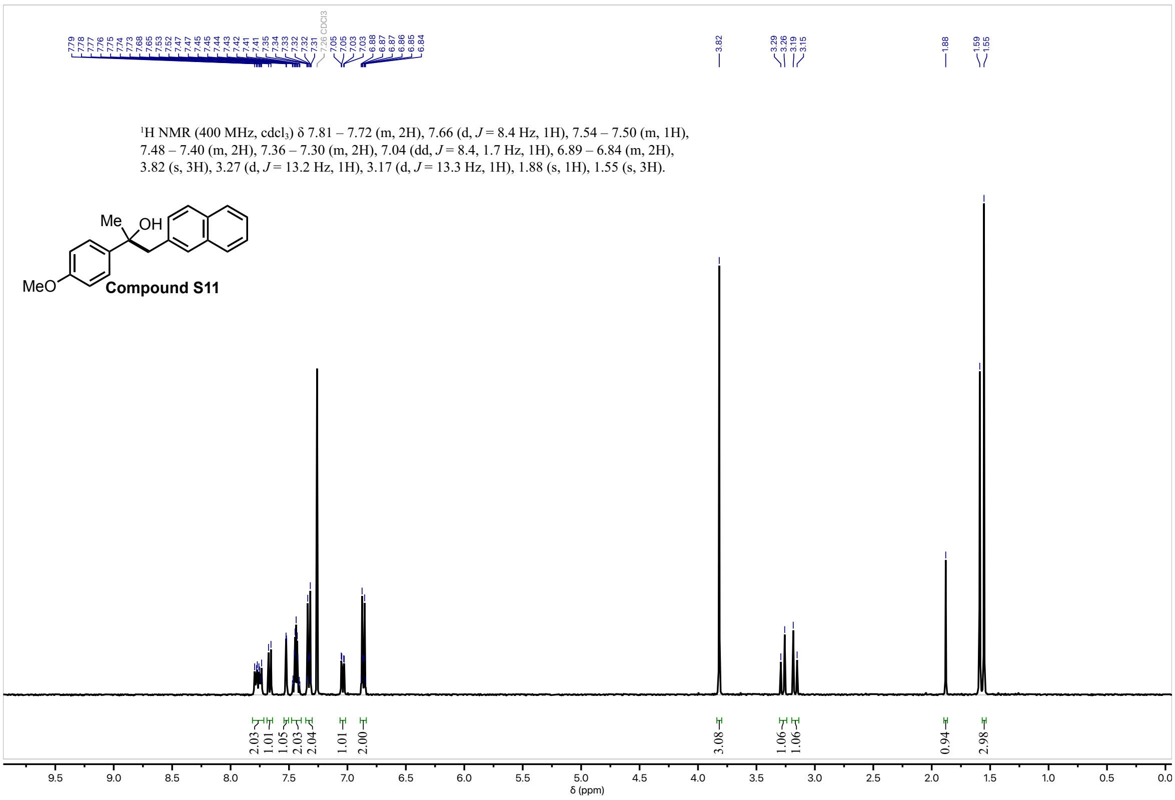

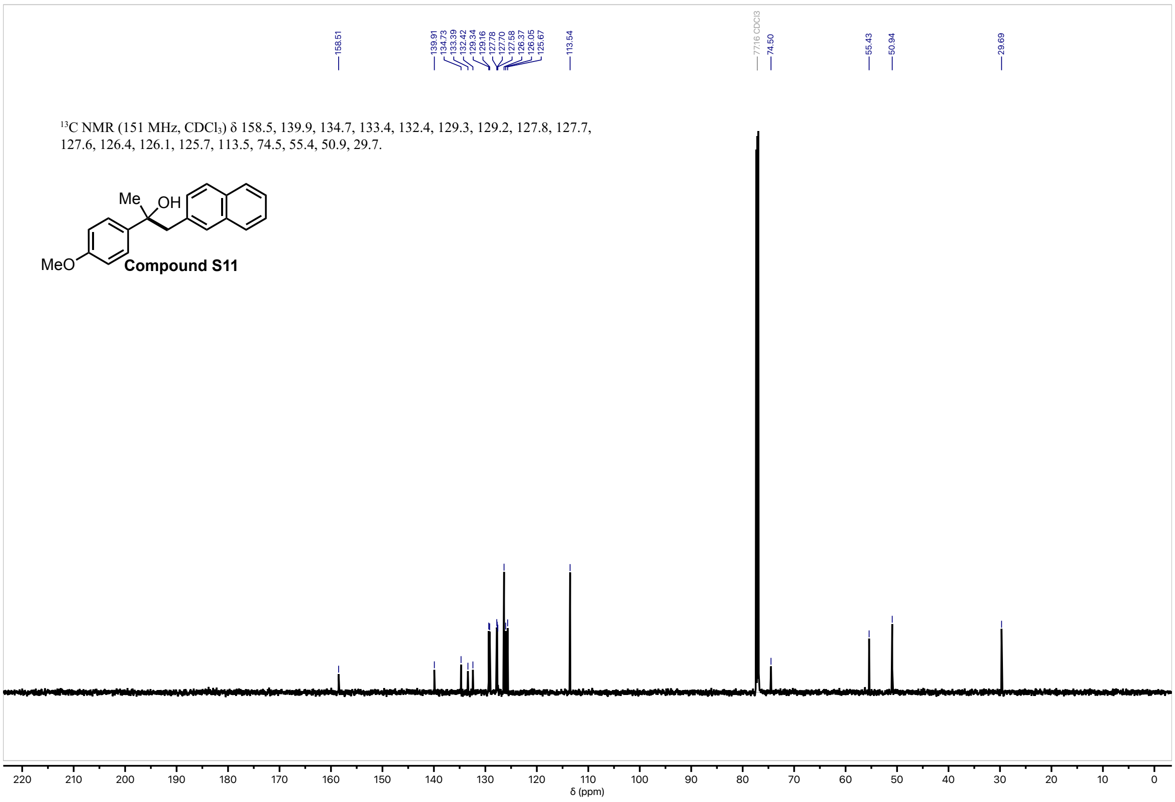

^{13}C NMR (151 MHz, $CDCl_3$) δ 158.5, 139.9, 134.7, 133.4, 132.4, 129.3, 129.2, 127.8, 127.7, 127.6, 126.4, 126.1, 125.7, 113.5, 74.5, 55.4, 50.9, 29.7.

Compound S11

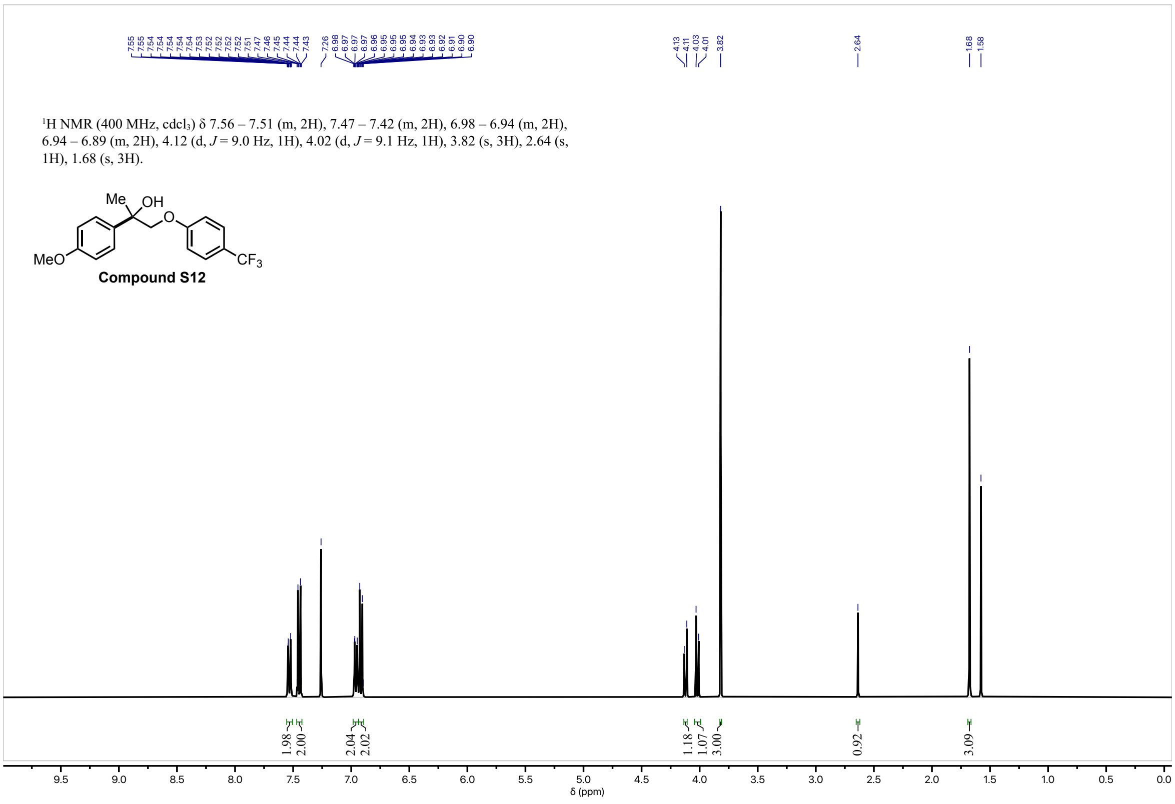

^{1}H NMR (400 MHz, cdcl$_3$) δ 7.56 – 7.51 (m, 2H), 7.47 – 7.42 (m, 2H), 6.98 – 6.94 (m, 2H), 6.94 – 6.89 (m, 2H), 4.12 (d, J = 9.0 Hz, 1H), 4.02 (d, J = 9.1 Hz, 1H), 3.82 (s, 3H), 2.64 (s, 1H), 1.68 (s, 3H).

Compound S12

^{13}C NMR (151 MHz, $CDCl_3$) δ 161.2, 159.1, 136.5, 127.1 (q, J = 4.0 Hz), 126.5, 124.5 (q, J = 271.0 Hz), 123.6 (q, J = 32.8 Hz), 114.8, 113.9, 76.1, 73.5, 55.4, 26.6.

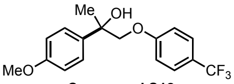

Compound S12

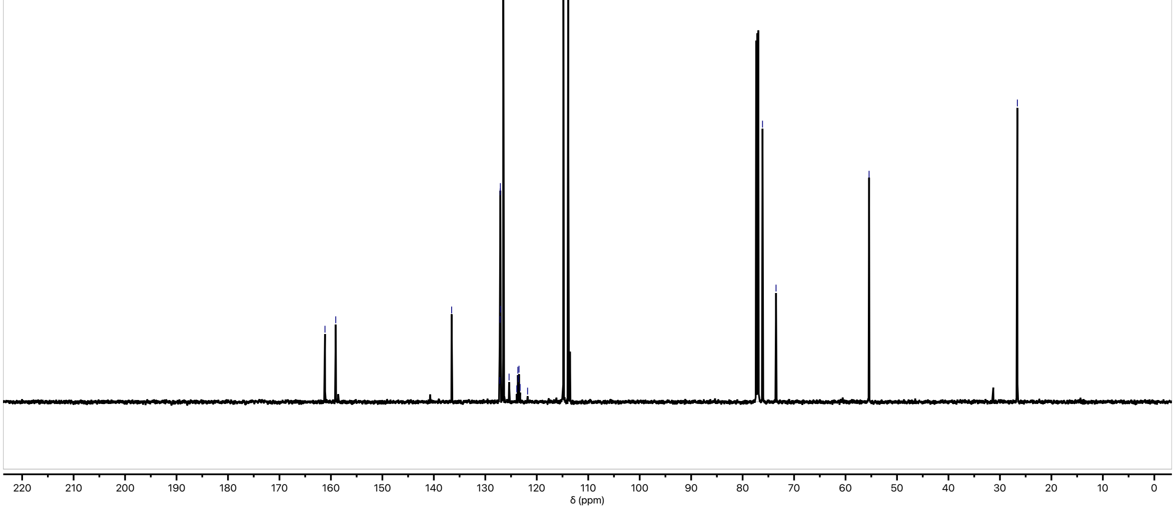

^{1}H NMR (400 MHz, $cdcl_3$) δ 7.47 – 7.42 (m, 2H), 7.09 – 7.03 (m, 2H), 6.92 – 6.88 (m, 2H), 6.82 – 6.77 (m, 2H), 4.06 (d, J = 9.1 Hz, 1H), 3.96 (d, J = 9.0 Hz, 1H), 3.81 (s, 3H), 2.77 (s, 1H), 2.28 (s, 3H), 1.64 (s, 3H).

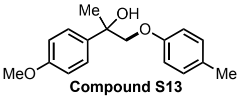

Compound S13

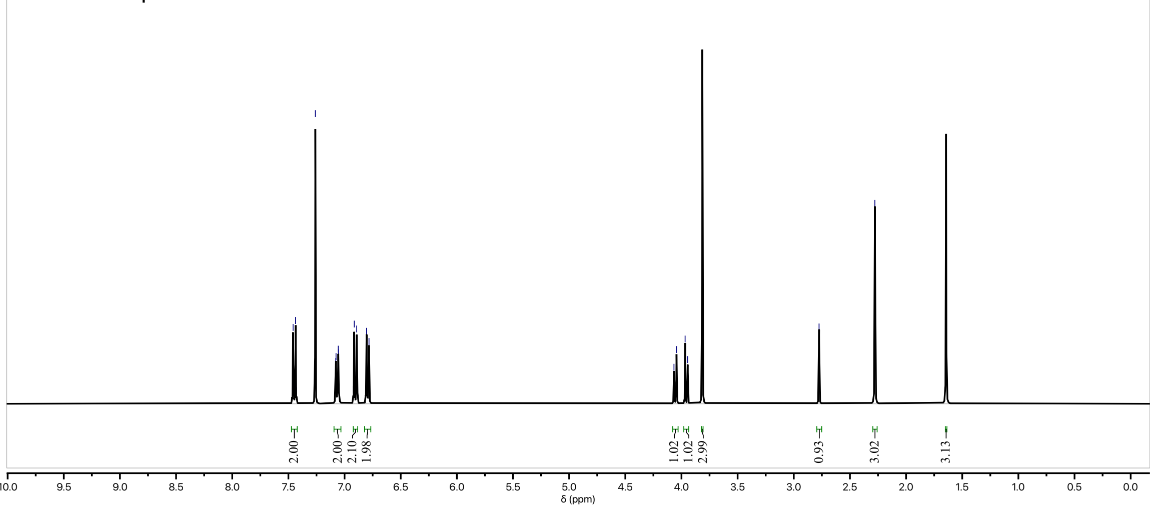

^{13}C NMR (151 MHz, CDCl$_3$) δ 158.9, 156.7, 137.1, 130.6, 130.1, 126.5, 114.7, 113.8, 76.2, 73.6, 55.4, 26.7, 20.6.

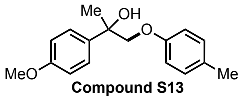

Compound S13

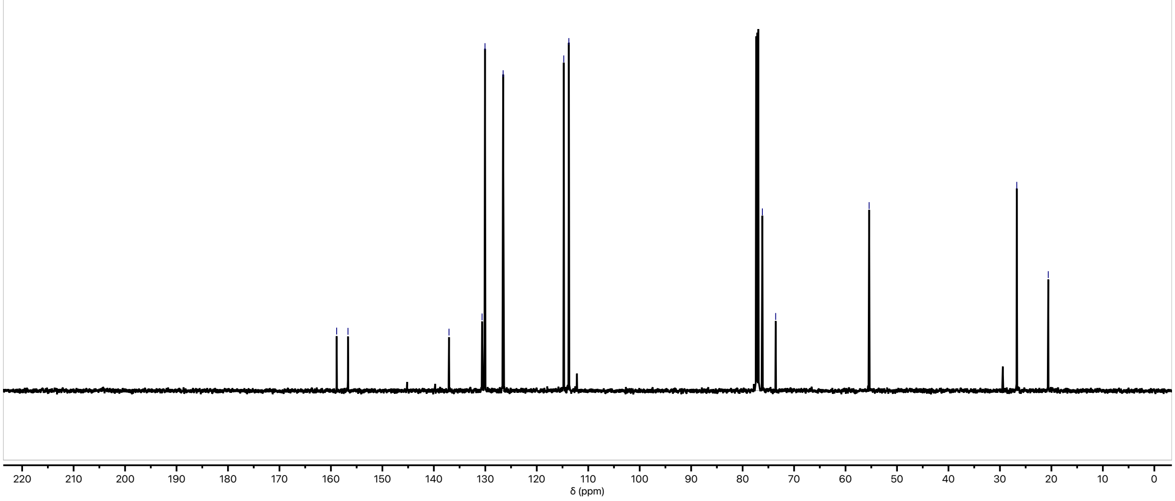

^{1}H NMR (600 MHz, CDCl$_3$) δ 7.47 – 7.41 (m, 2H), 7.25 – 7.20 (m, 2H), 6.93 – 6.89 (m, 2H), 6.84 – 6.80 (m, 2H), 4.05 (d, J = 9.0 Hz, 1H), 3.96 (d, J = 9.0 Hz, 1H), 3.82 (s, 3H), 2.66 (s, 1H), 1.65 (s, 3H).

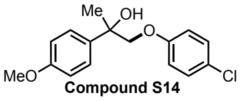

Compound S14

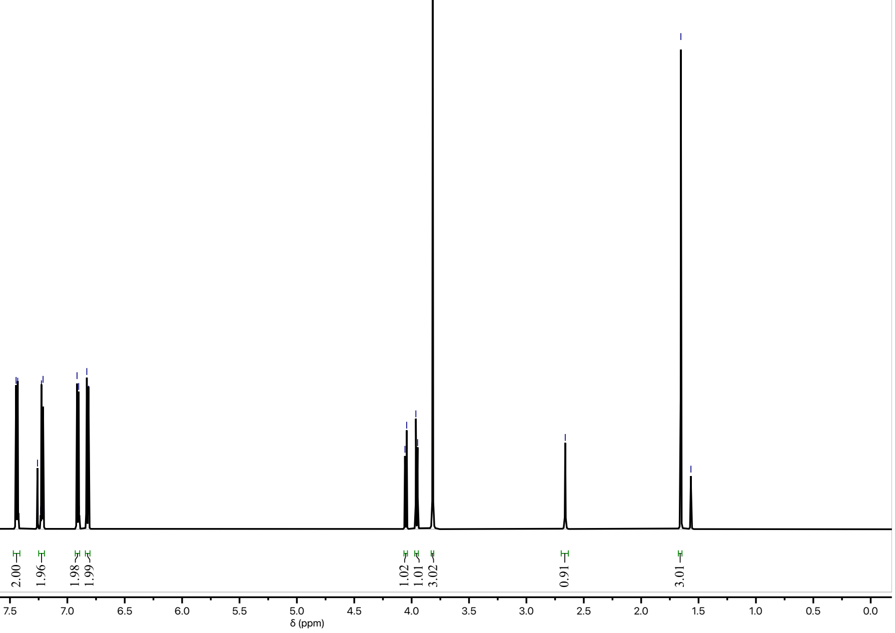

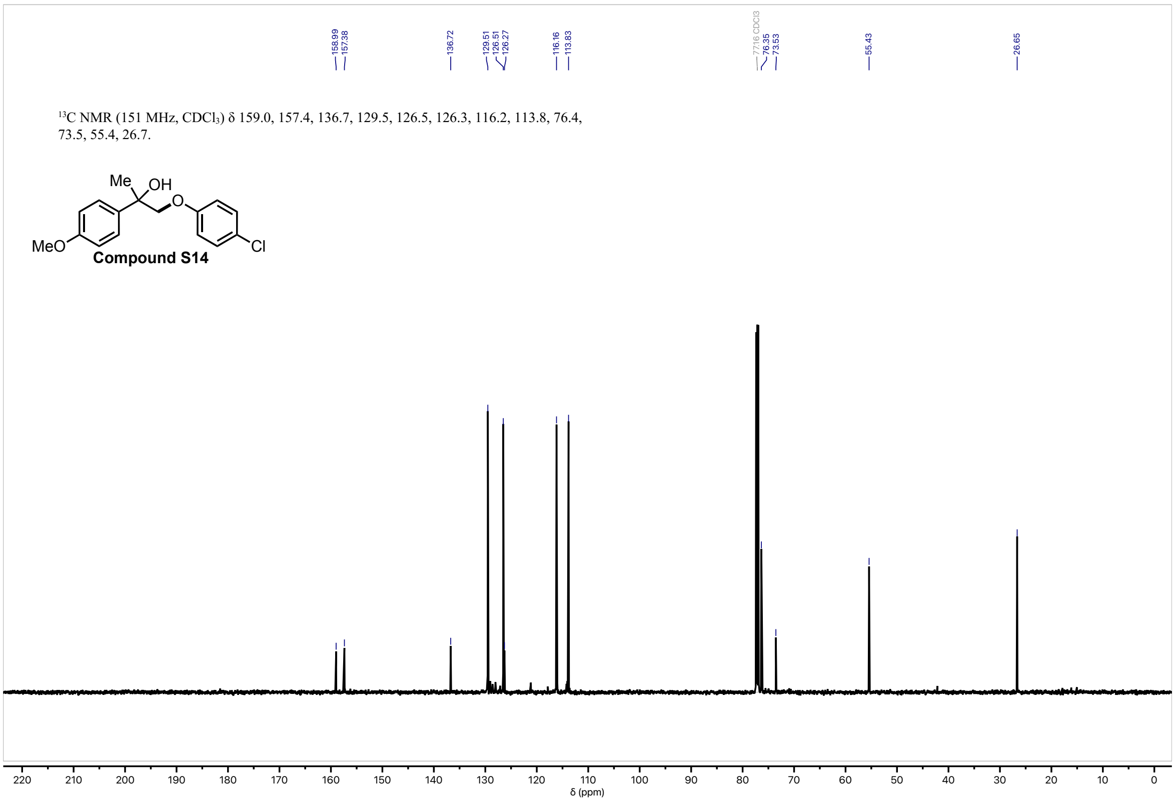

^{13}C NMR (151 MHz, $CDCl_3$) δ 159.0, 157.4, 136.7, 129.5, 126.5, 126.3, 116.2, 113.8, 76.4, 73.5, 55.4, 26.7.

Compound S14

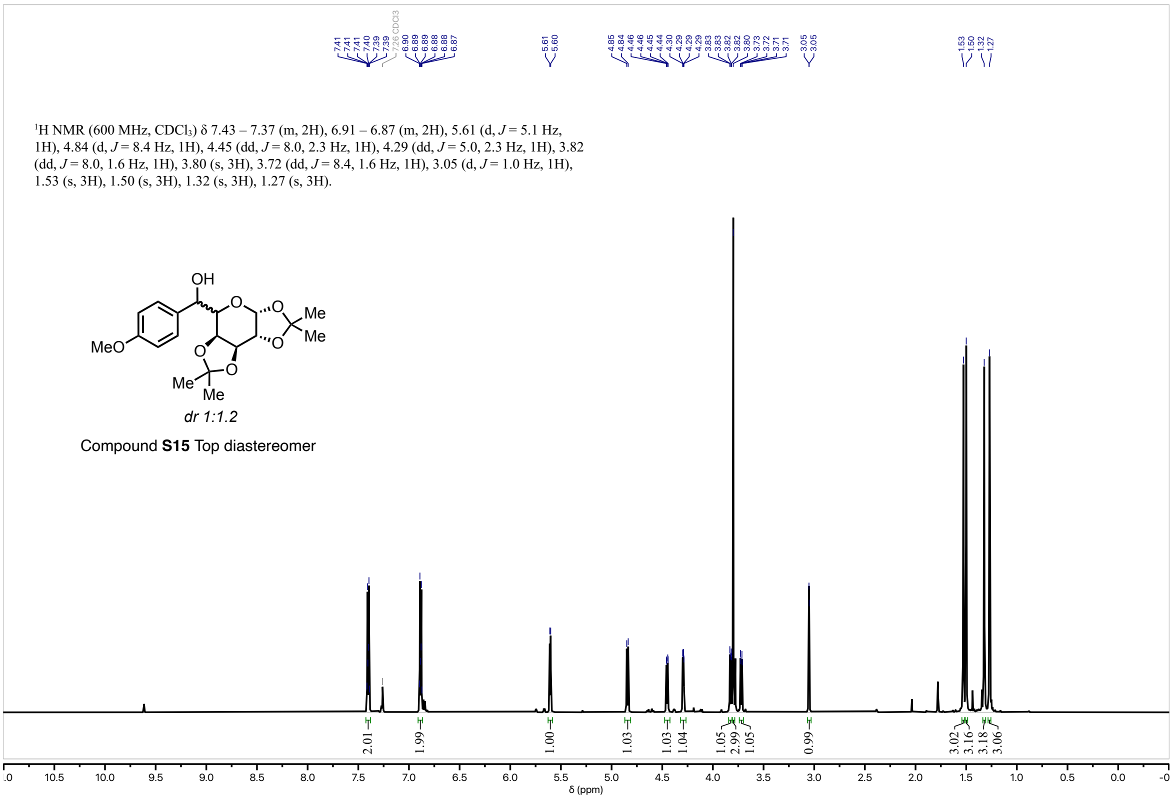

^{1}H NMR (600 MHz, $CDCl_3$) δ 7.43 – 7.37 (m, 2H), 6.91 – 6.87 (m, 2H), 5.61 (d, J = 5.1 Hz, 1H), 4.84 (d, J = 8.4 Hz, 1H), 4.45 (dd, J = 8.0, 2.3 Hz, 1H), 4.29 (dd, J = 5.0, 2.3 Hz, 1H), 3.82 (dd, J = 8.0, 1.6 Hz, 1H), 3.80 (s, 3H), 3.72 (dd, J = 8.4, 1.6 Hz, 1H), 3.05 (d, J = 1.0 Hz, 1H), 1.53 (s, 3H), 1.50 (s, 3H), 1.32 (s, 3H), 1.27 (s, 3H).

dr 1:1.2

Compound **S15** Top diastereomer

^{13}C NMR (151 MHz, $CDCl_3$) δ 159.5, 131.4, 128.6, 113.8, 109.4, 108.9, 96.6, 72.9, 72.3, 71.0, 70.9, 70.6, 55.3, 26.2, 26.1, 25.1, 24.3.

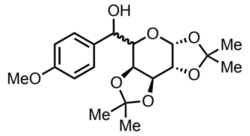

dr 1:1.2

Compound **S15** Top diastereomer

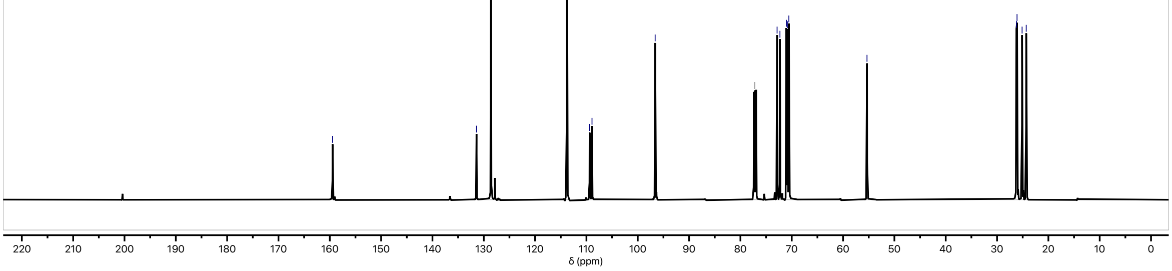

^{1}H NMR (600 MHz, $CDCl_3$) δ 7.35 – 7.30 (m, 2H), 6.90 – 6.85 (m, 2H), 5.51 (d, J = 5.0 Hz, 1H), 4.84 (t, J = 6.0 Hz, 1H), 4.57 (dd, J = 8.1, 2.4 Hz, 1H), 4.41 (dd, J = 8.1, 1.8 Hz, 1H), 4.27 (dd, J = 5.1, 2.4 Hz, 1H), 3.88 (dd, J = 7.1, 1.8 Hz, 1H), 3.78 (s, 3H), 3.23 (d, J = 5.6 Hz, 1H), 1.50 (s, 3H), 1.48 (s, 3H), 1.34 (s, 3H), 1.28 (s, 3H).

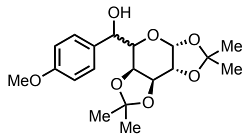

dr 1:1.2

Compound **S15** Low diastereomer

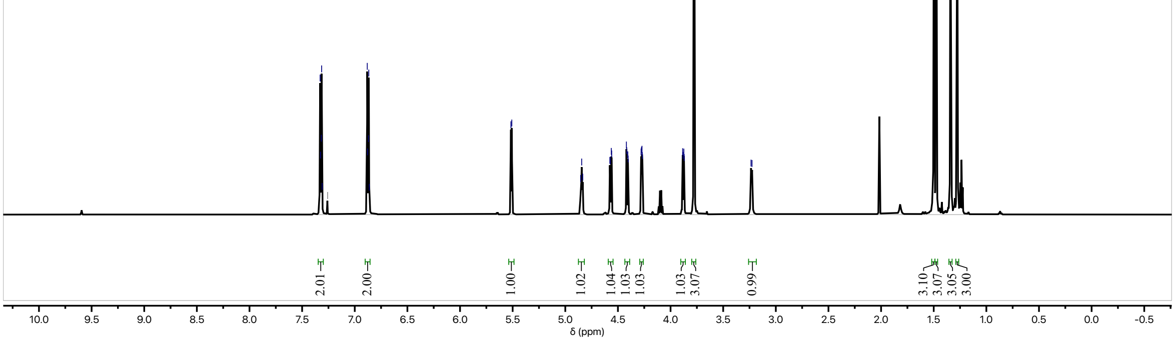

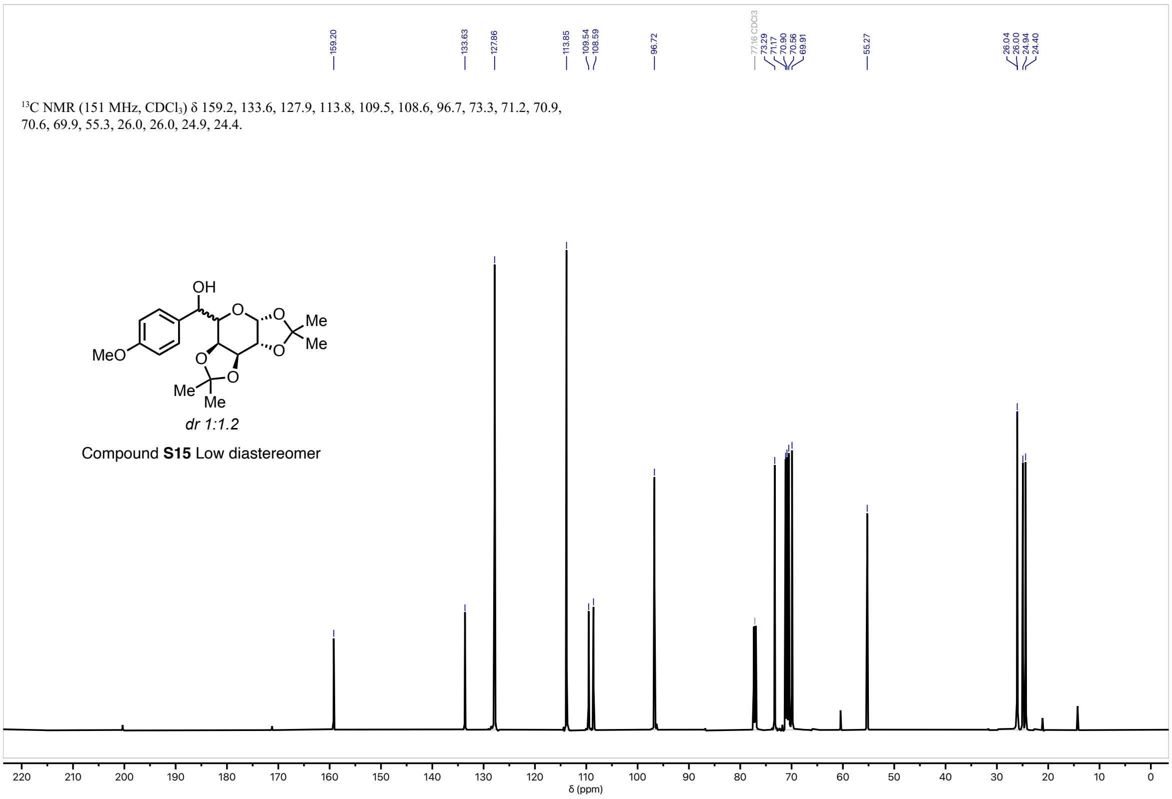
^{13}C NMR (151 MHz, $CDCl_3$) δ 159.2, 133.6, 127.9, 113.8, 109.5, 108.6, 96.7, 73.3, 71.2, 70.9, 70.6, 69.9, 55.3, 26.0, 26.0, 24.9, 24.4.
OH
MeO
Me
Me
Me
Me
dr 1:1.2
Compound **S15** Low diastereomer
δ (ppm)

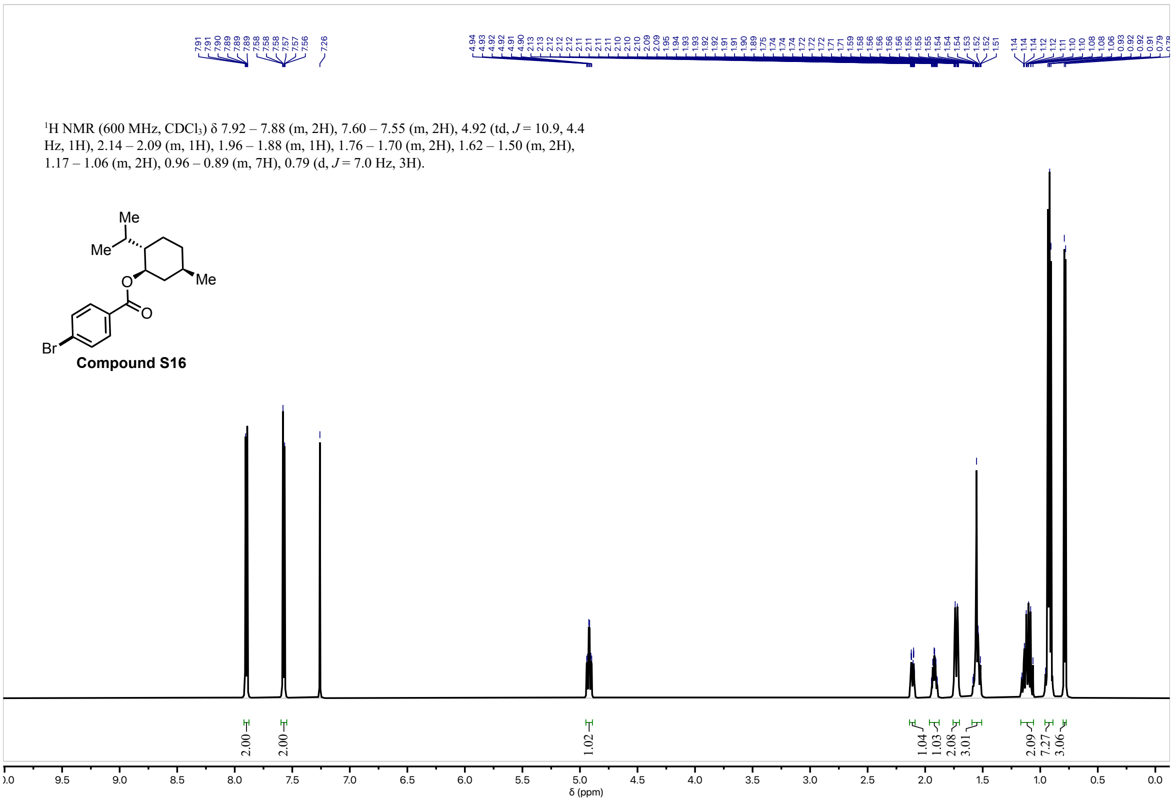
^{1}H NMR (600 MHz, $CDCl_3$) δ 7.92 – 7.88 (m, 2H), 7.60 – 7.55 (m, 2H), 4.92 (td, J = 10.9, 4.4 Hz, 1H), 2.14 – 2.09 (m, 1H), 1.96 – 1.88 (m, 1H), 1.76 – 1.70 (m, 2H), 1.62 – 1.50 (m, 2H), 1.17 – 1.06 (m, 2H), 0.96 – 0.89 (m, 7H), 0.79 (d, J = 7.0 Hz, 3H).
Me
Me
Me
O
Br
Compound S16
2.00
2.00
1.02
1.04
1.03
2.08
3.01
2.09
7.27
3.06
δ (ppm)

^{13}C NMR (151 MHz, $CDCl_3$) δ 165.5, 131.8, 131.3, 129.9, 127.9, 75.4, 47.4, 41.1, 34.4, 31.6, 26.7, 23.8, 22.2, 20.9, 16.7.

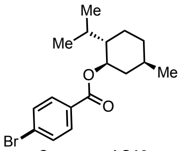

Compound S16

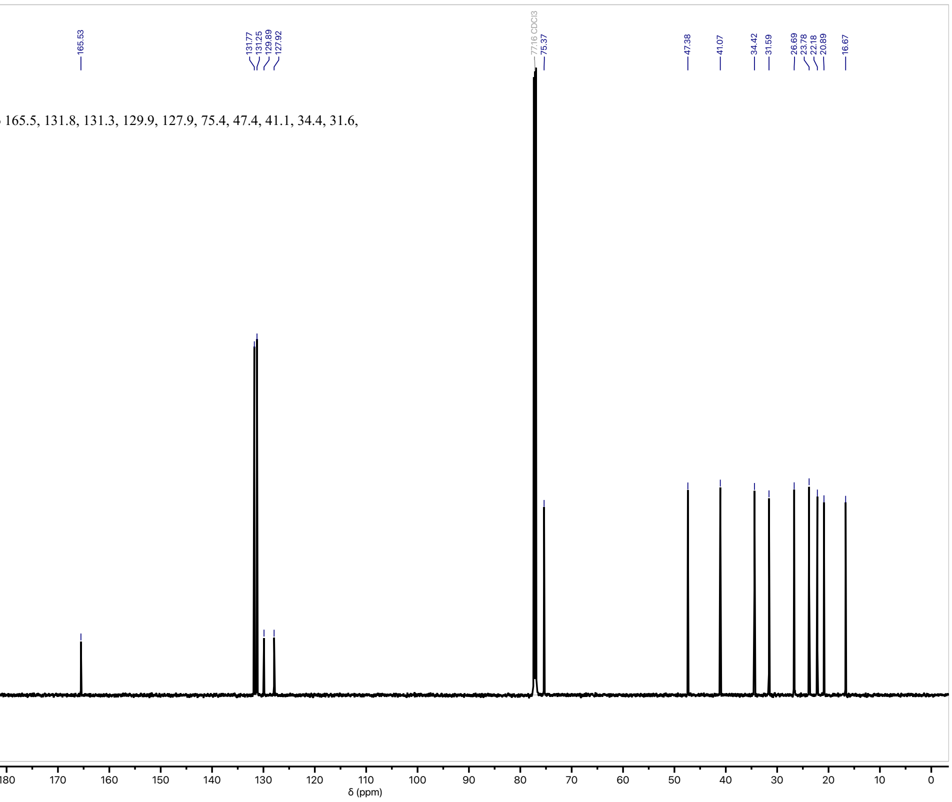

^{1}H NMR (600 MHz, $CDCl_3$) δ 7.38 – 7.34 (m, 2H), 6.88 – 6.84 (m, 2H), 3.80 (s, 3H), 2.33 – 2.26 (m, 1H), 1.72 – 1.66 (m, 1H), 1.60 – 1.56 (m, 2H), 1.54 – 1.44 (m, 4H), 1.52 (s, 3H), 1.41 – 1.35 (m, 1H), 1.29 – 1.23 (m, 1H).

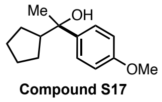

Compound S17

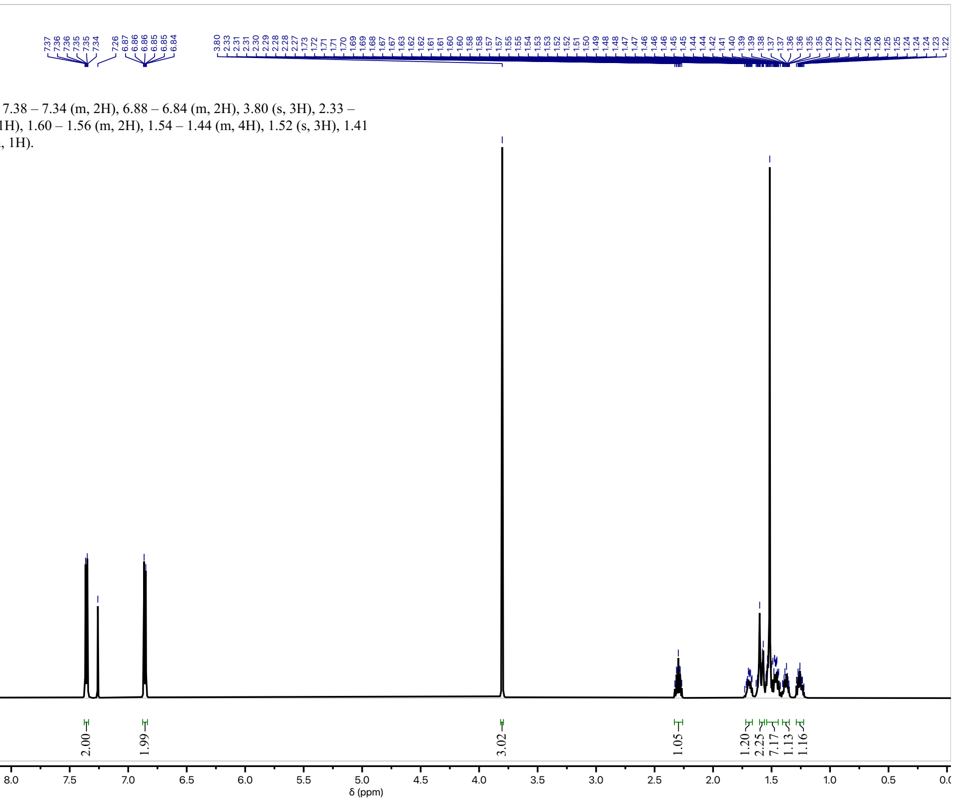

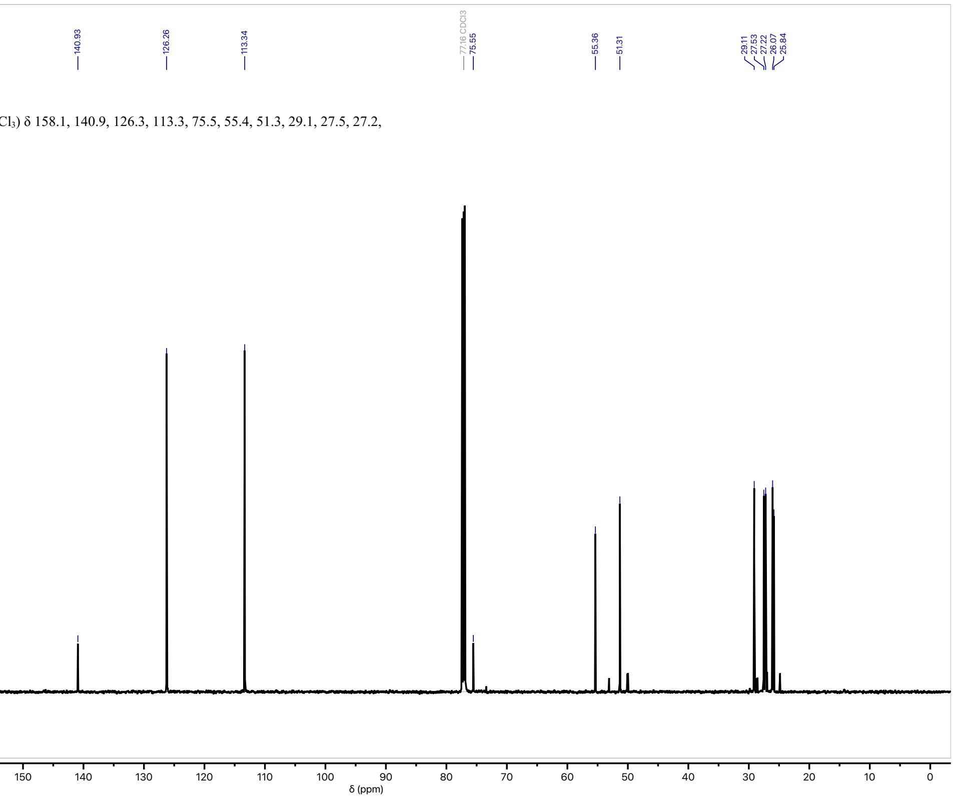

^{13}C NMR (151 MHz, $CDCl_3$) δ 158.1, 140.9, 126.3, 113.3, 75.5, 55.4, 51.3, 29.1, 27.5, 27.2, 26.1, 25.8.

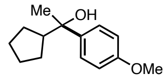

Compound S17

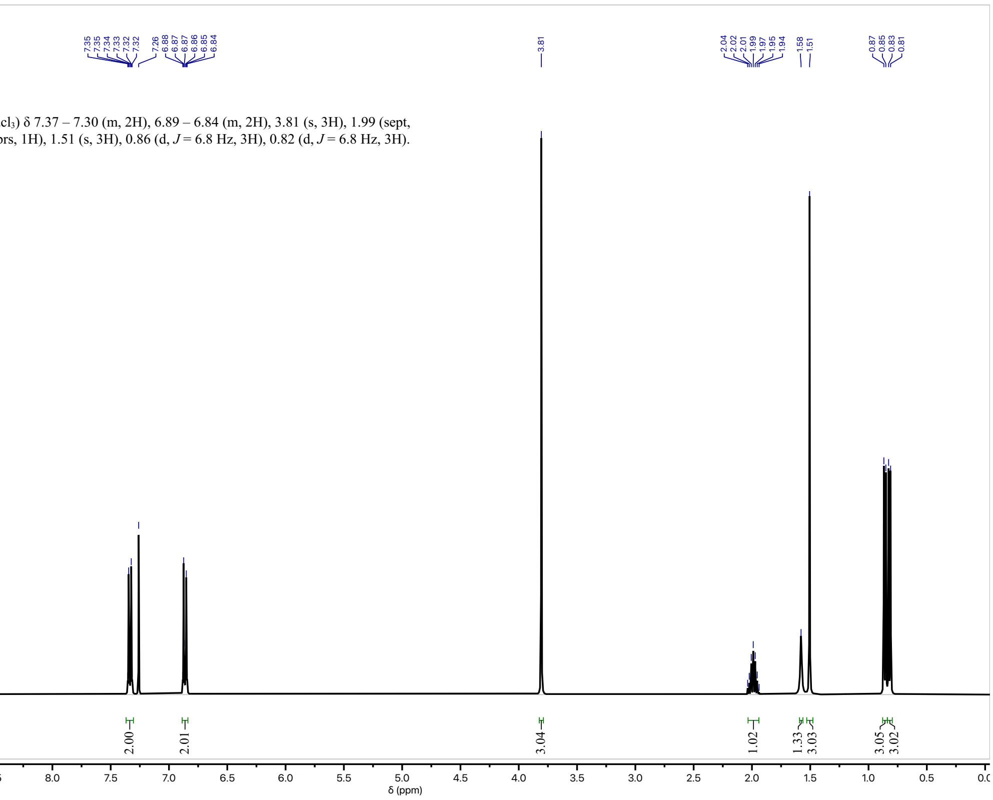

^{1}H NMR (400 MHz, cdcl$_3$) δ 7.37 – 7.30 (m, 2H), 6.89 – 6.84 (m, 2H), 3.81 (s, 3H), 1.99 (sept, J = 6.8 Hz, 1H), 1.58 (brs, 1H), 1.51 (s, 3H), 0.86 (d, J = 6.8 Hz, 3H), 0.82 (d, J = 6.8 Hz, 3H).

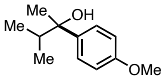

Compound S18

^{1}H NMR (600 MHz, $CDCl_3$) δ 7.27 (d, *J* = 8.6 Hz, 2H), 6.88 (d, *J* = 8.6 Hz, 2H), 4.61 (t, *J* = 6.8 Hz, 1H), 3.81 (s, 3H), 1.84 – 1.77 (m, 1H), 1.74 (s, 1H), 1.72 – 1.65 (m, 1H), 1.41 – 1.30 (m, 3H), 1.26 – 1.19 (m, 1H), 0.88 (t, *J* = 7.2 Hz, 3H).

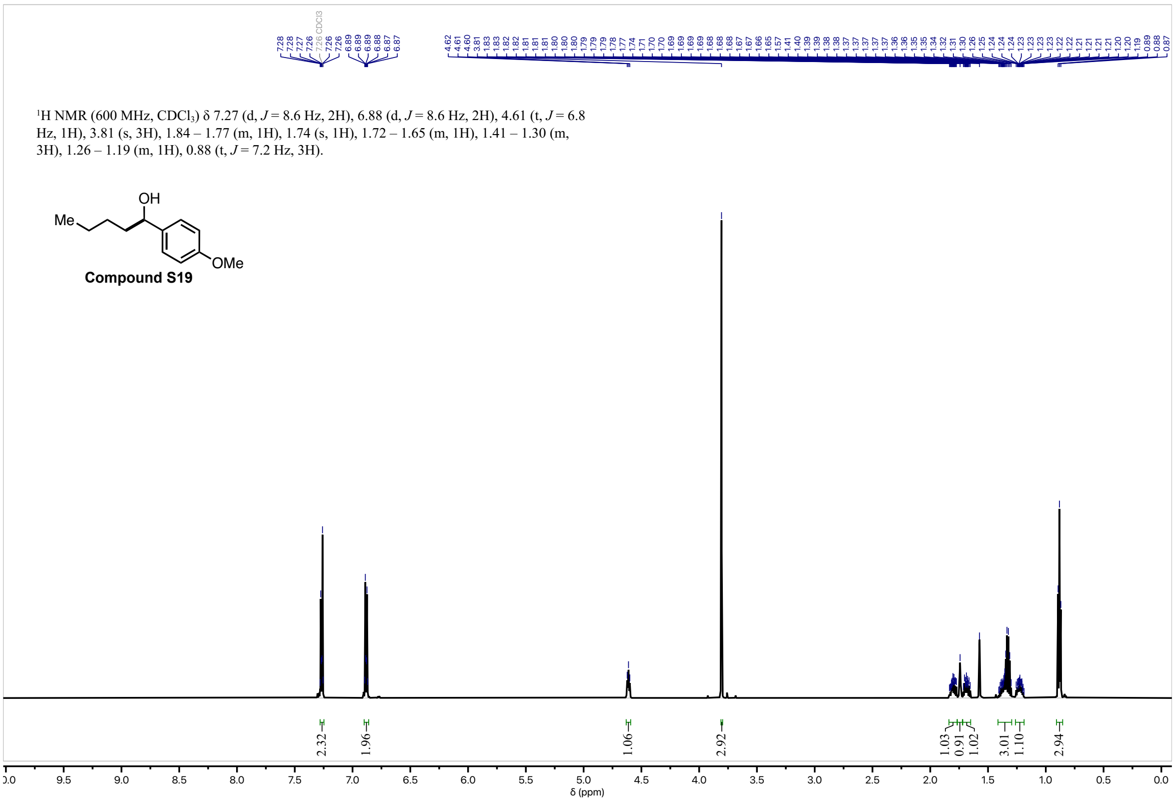

^{13}C NMR (151 MHz, $CDCl_3$) δ 159.1, 137.2, 127.3, 113.9, 74.5, 55.4, 38.8, 28.2, 22.8, 14.2.

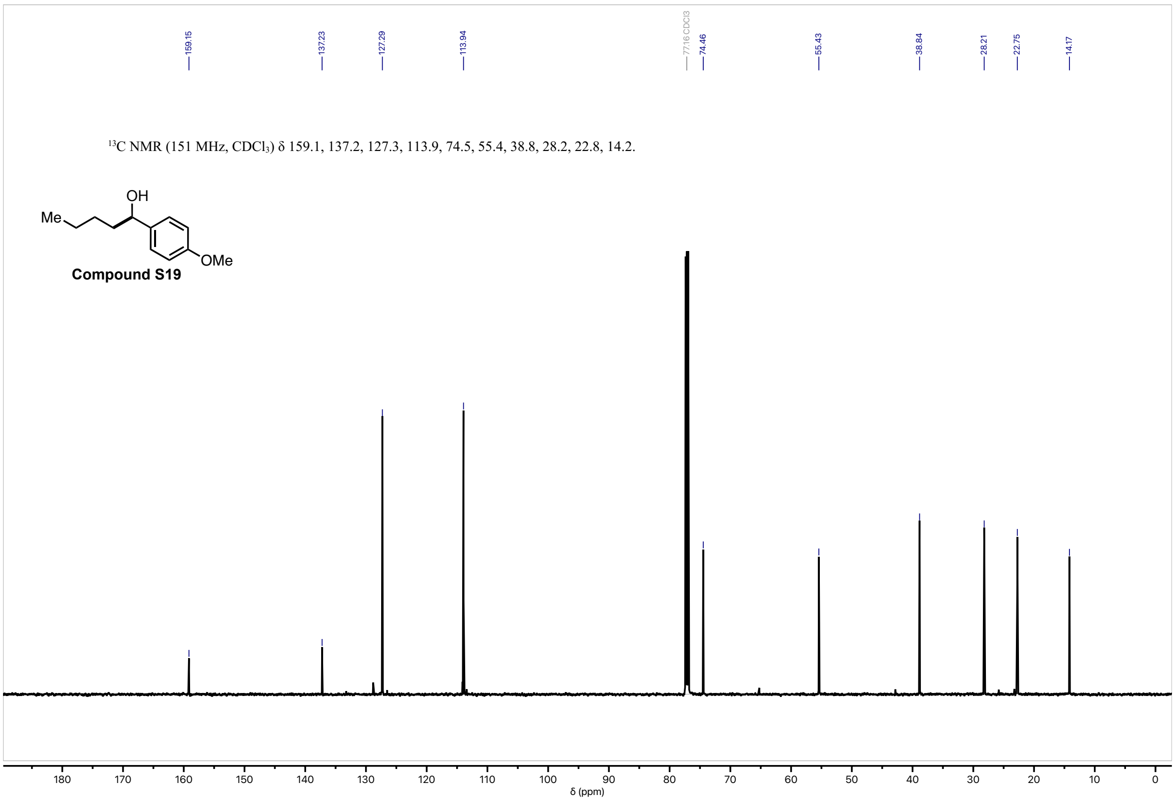

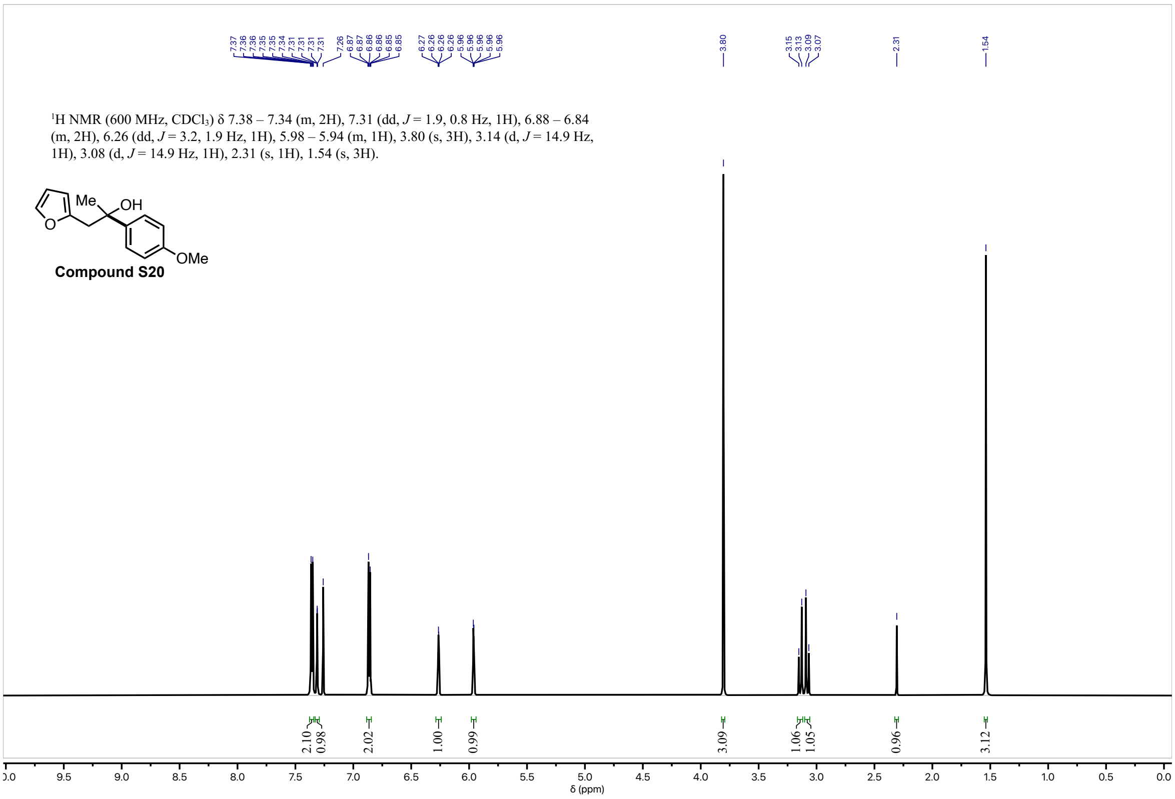

^{1}H NMR (600 MHz, $CDCl_3$) δ 7.38 – 7.34 (m, 2H), 7.31 (dd, J = 1.9, 0.8 Hz, 1H), 6.88 – 6.84 (m, 2H), 6.26 (dd, J = 3.2, 1.9 Hz, 1H), 5.98 – 5.94 (m, 1H), 3.80 (s, 3H), 3.14 (d, J = 14.9 Hz, 1H), 3.08 (d, J = 14.9 Hz, 1H), 2.31 (s, 1H), 1.54 (s, 3H).

Compound S20

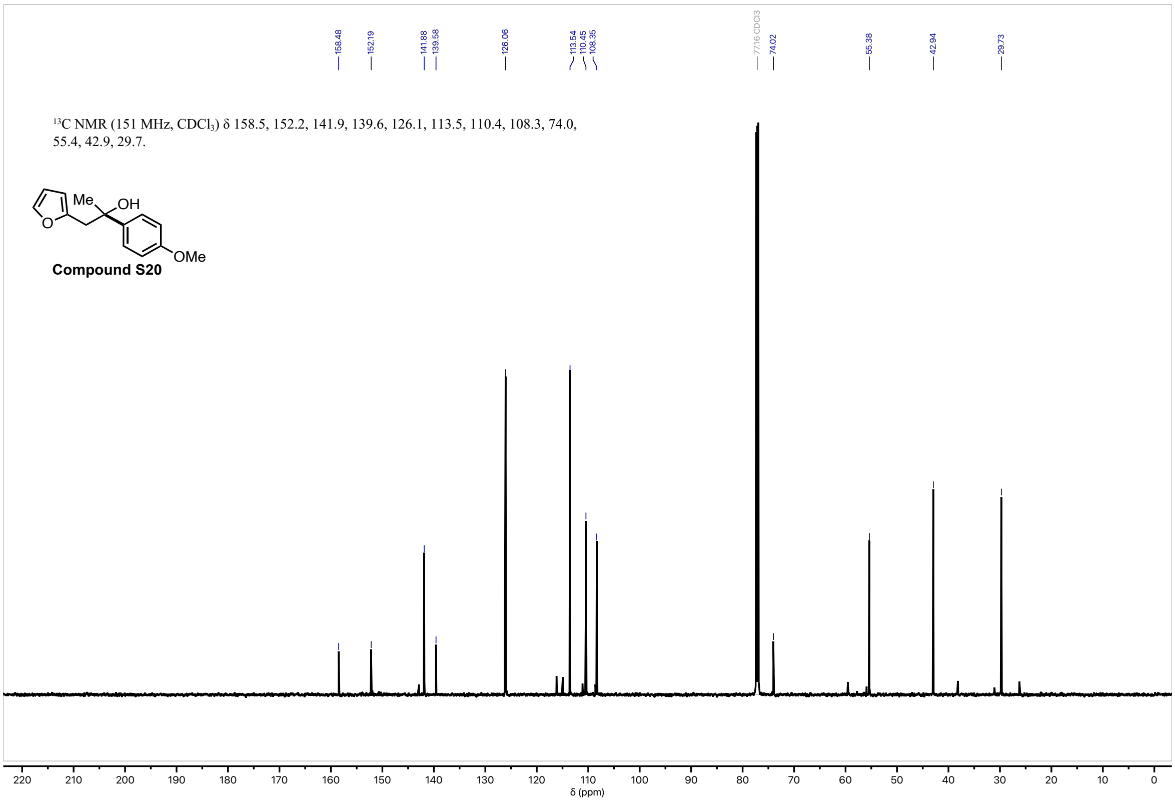

^{13}C NMR (151 MHz, $CDCl_3$) δ 158.5, 152.2, 141.9, 139.6, 126.1, 113.5, 110.4, 108.3, 74.0, 55.4, 42.9, 29.7.

Compound S20

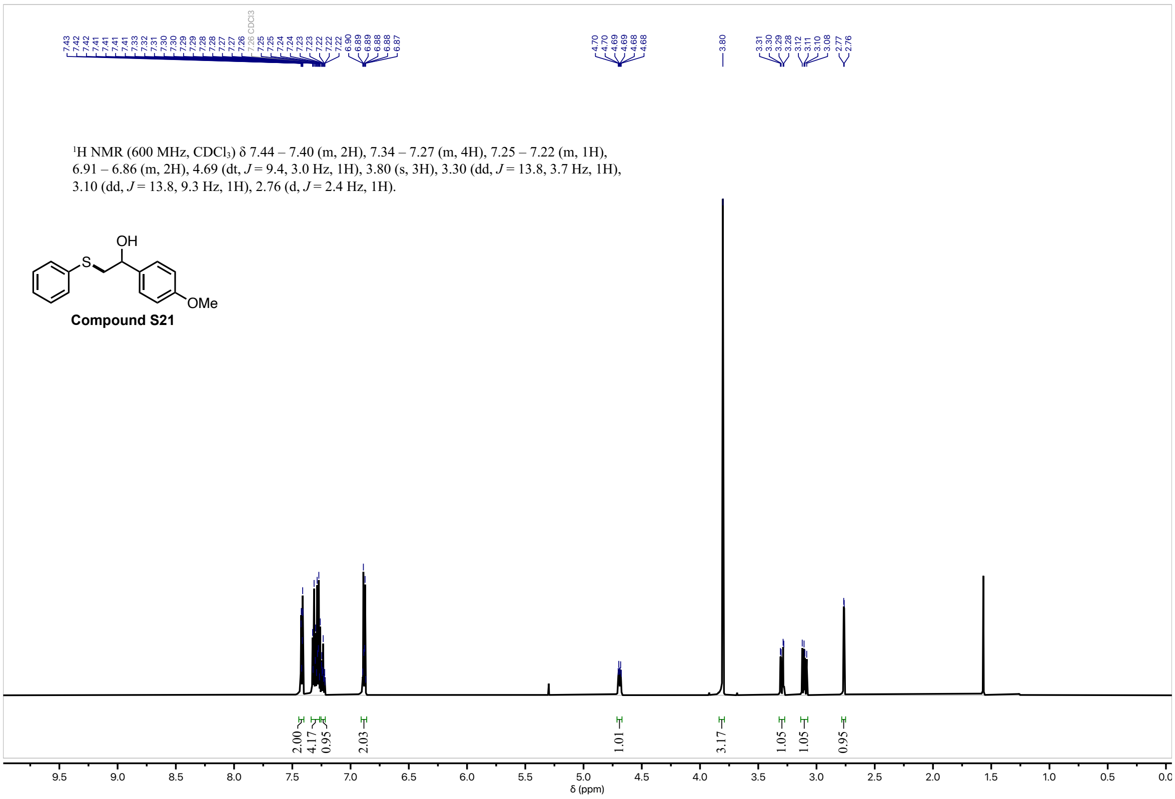

^{1}H NMR (600 MHz, $CDCl_3$) δ 7.44 – 7.40 (m, 2H), 7.34 – 7.27 (m, 4H), 7.25 – 7.22 (m, 1H), 6.91 – 6.86 (m, 2H), 4.69 (dt, *J* = 9.4, 3.0 Hz, 1H), 3.80 (s, 3H), 3.30 (dd, *J* = 13.8, 3.7 Hz, 1H), 3.10 (dd, *J* = 13.8, 9.3 Hz, 1H), 2.76 (d, *J* = 2.4 Hz, 1H).

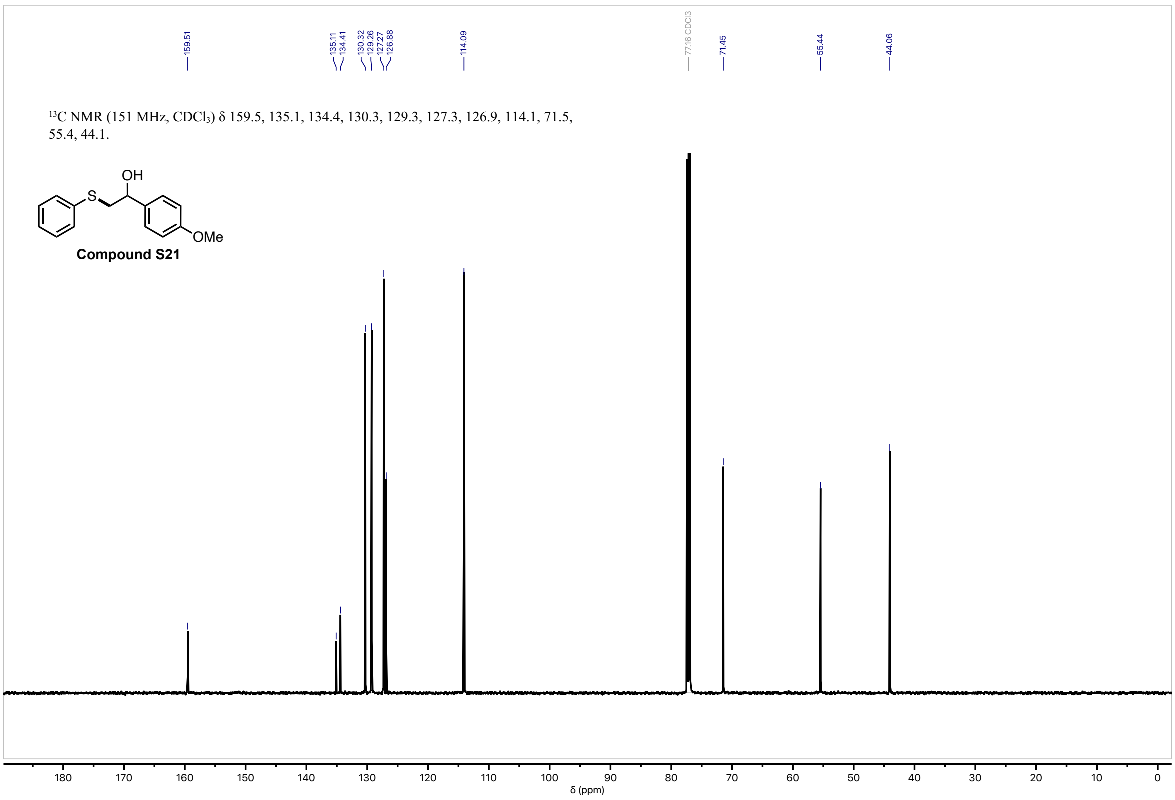

^{13}C NMR (151 MHz, $CDCl_3$) δ 159.5, 135.1, 134.4, 130.3, 129.3, 127.3, 126.9, 114.1, 71.5, 55.4, 44.1.

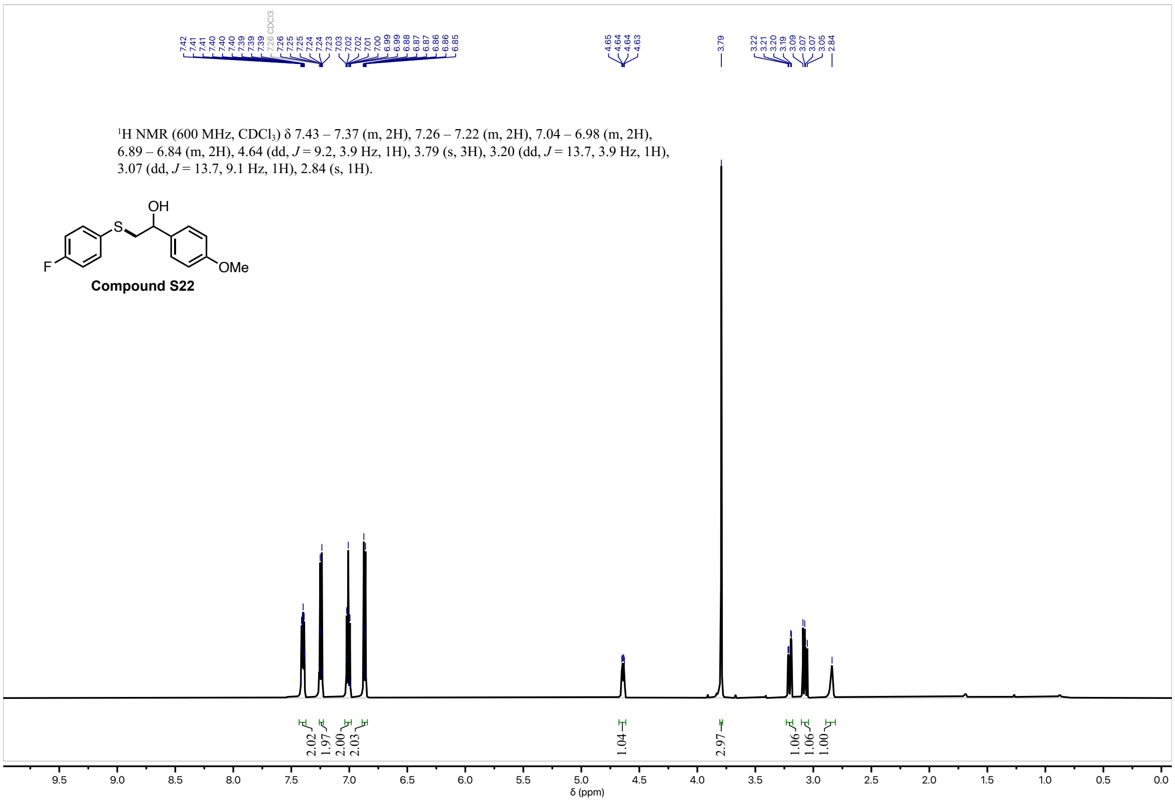

^{1}H NMR (600 MHz, CDCl3) δ 7.43 – 7.37 (m, 2H), 7.26 – 7.22 (m, 2H), 7.04 – 6.98 (m, 2H), 6.89 – 6.84 (m, 2H), 4.64 (dd, J = 9.2, 3.9 Hz, 1H), 3.79 (s, 3H), 3.20 (dd, J = 13.7, 3.9 Hz, 1H), 3.07 (dd, J = 13.7, 9.1 Hz, 1H), 2.84 (s, 1H).
OH
S
F
OMe
Compound S22
2.02
1.97
2.00
2.03
1.04
2.97
1.06
1.06
1.00
δ (ppm)

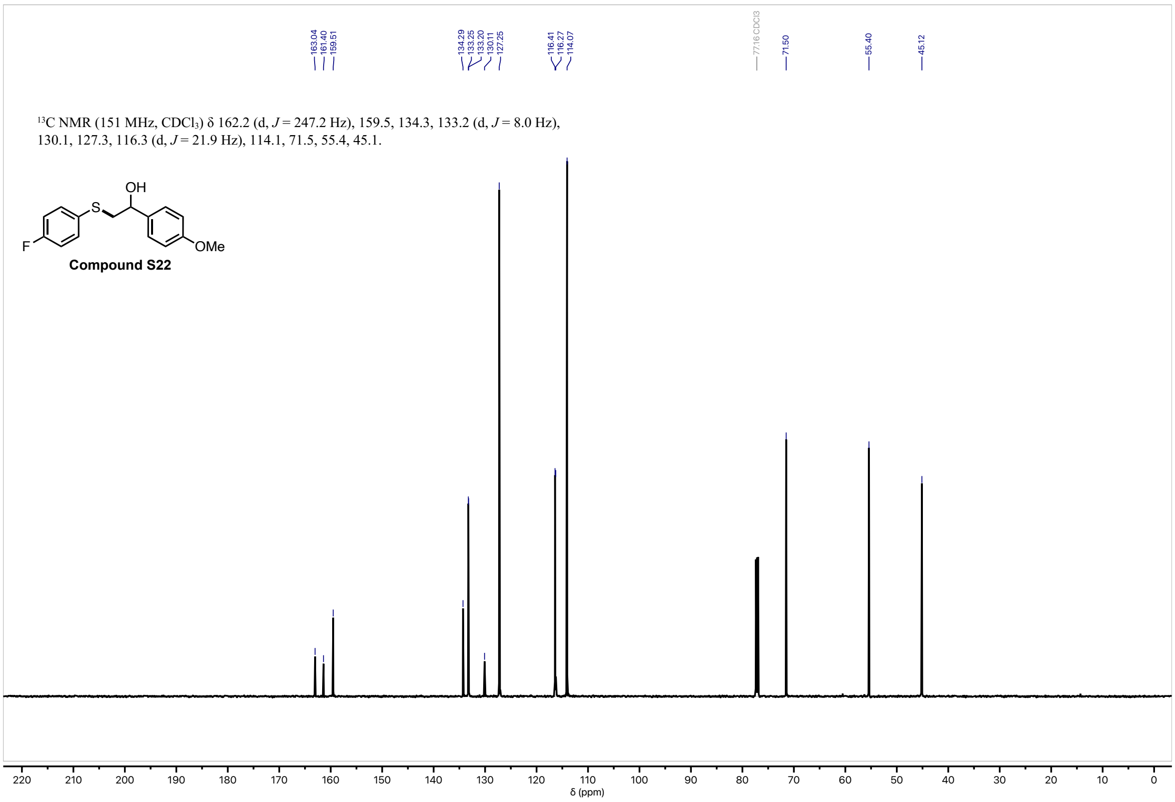

^{13}C NMR (151 MHz, CDCl3) δ 162.2 (d, J = 247.2 Hz), 159.5, 134.3, 133.2 (d, J = 8.0 Hz), 130.1, 127.3, 116.3 (d, J = 21.9 Hz), 114.1, 71.5, 55.4, 45.1.
OH
S
F
OMe
Compound S22
77.16 CDCl3
δ (ppm)

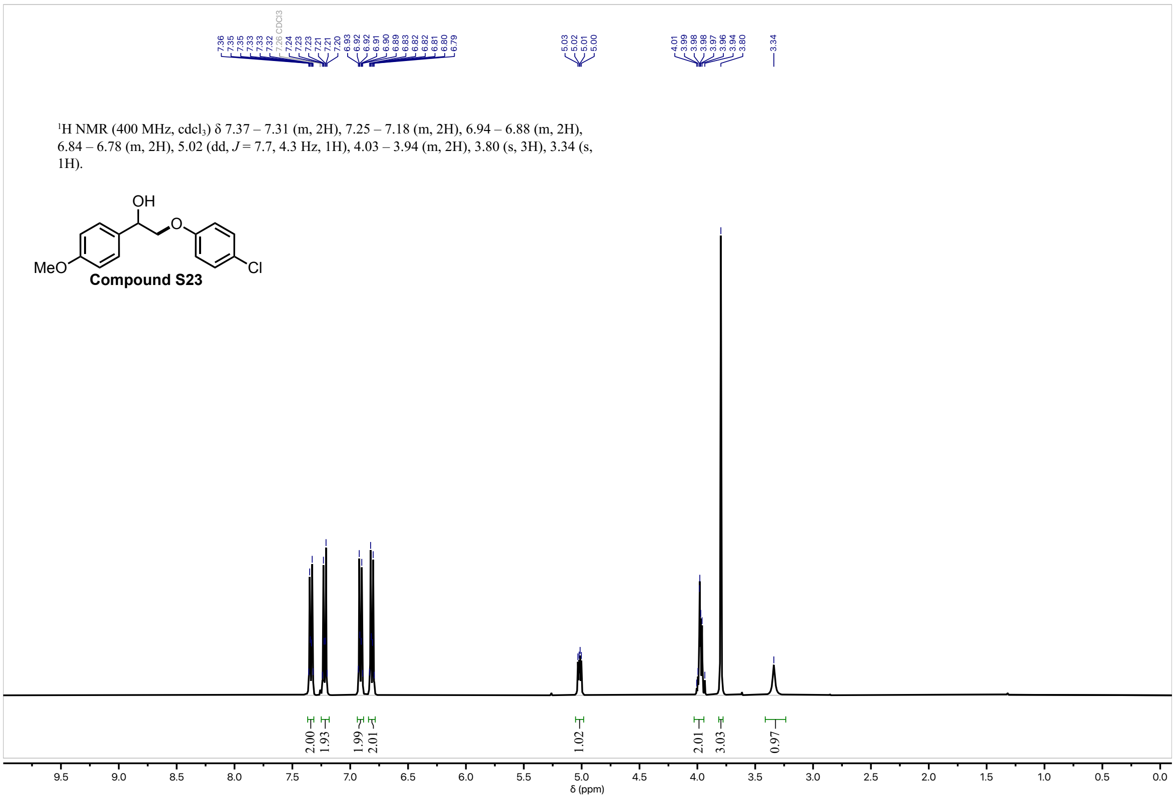
1H NMR (400 MHz, cdcl3) δ 7.37 – 7.31 (m, 2H), 7.25 – 7.18 (m, 2H), 6.94 – 6.88 (m, 2H), 6.84 – 6.78 (m, 2H), 5.02 (dd, J = 7.7, 4.3 Hz, 1H), 4.03 – 3.94 (m, 2H), 3.80 (s, 3H), 3.34 (s, 1H).
OH
O
MeO
Cl
Compound S23
δ (ppm)

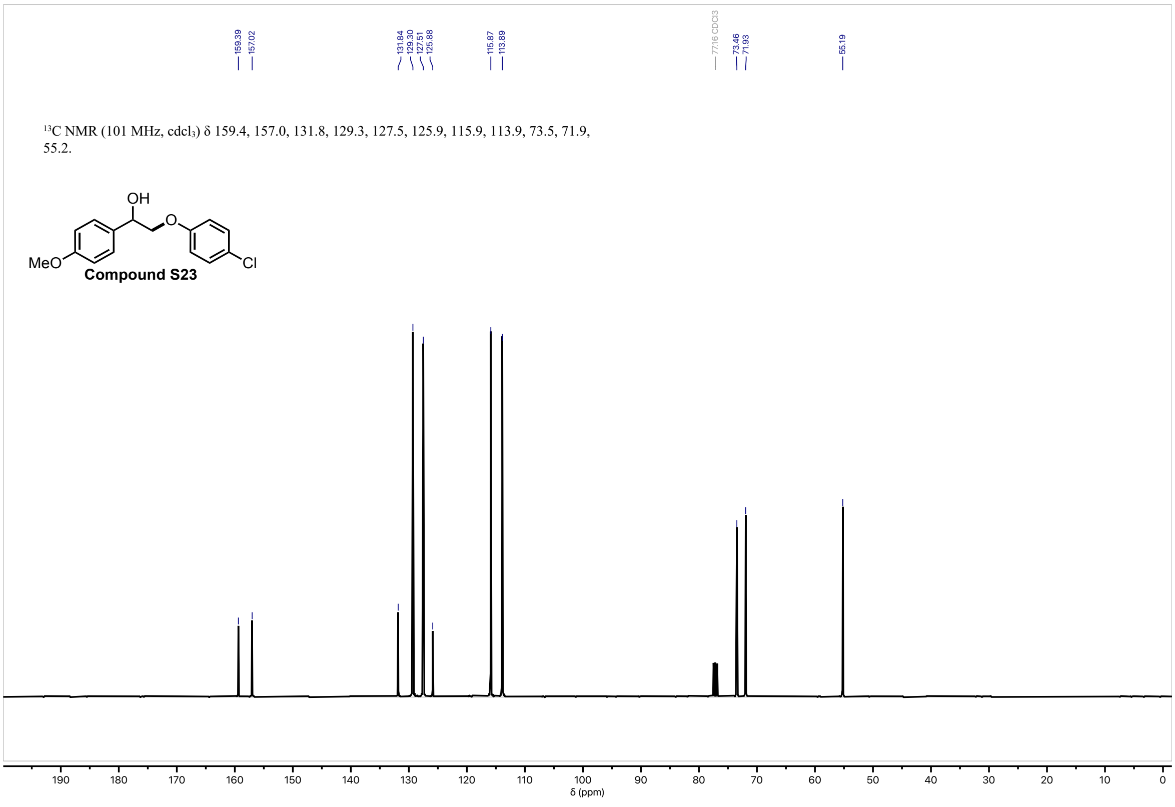
13C NMR (101 MHz, cdcl3) δ 159.4, 157.0, 131.8, 129.3, 127.5, 125.9, 115.9, 113.9, 73.5, 71.9, 55.2.
OH
O
MeO
Cl
Compound S23
δ (ppm)

^{1}H NMR (600 MHz, $CDCl_3$) δ 7.37 (d, J = 8.6 Hz, 2H), 7.08 (d, J = 8.6 Hz, 2H), 6.92 (d, J = 8.7 Hz, 2H), 6.82 (d, J = 8.6 Hz, 2H), 5.06 (dt, J = 9.0, 2.8 Hz, 1H), 4.04 (dd, J = 9.6, 3.2 Hz, 1H), 3.96 (t, J = 9.3 Hz, 1H), 3.82 (s, 3H), 2.73 (d, J = 2.4 Hz, 1H), 2.29 (s, 3H).

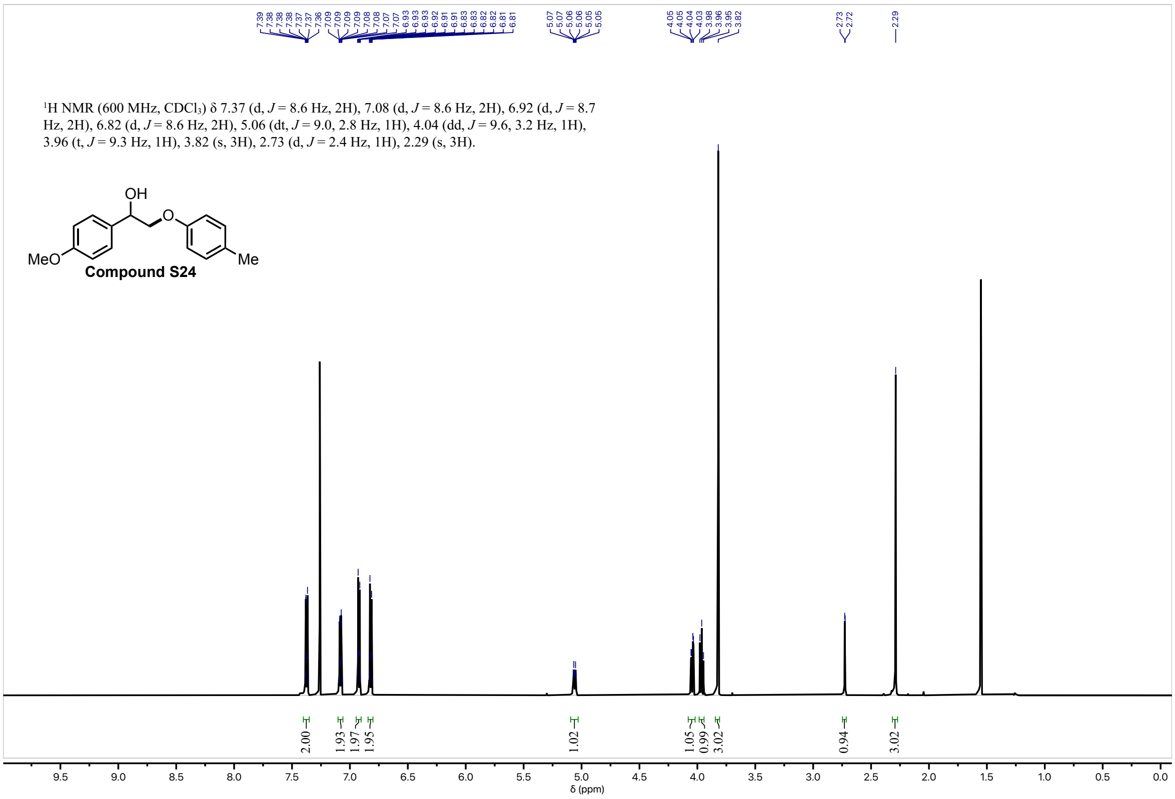

^{13}C NMR (151 MHz, $CDCl_3$) δ 159.7, 156.4, 131.9, 130.7, 130.1, 127.7, 114.6, 114.1, 73.6, 72.4, 55.5, 20.6.

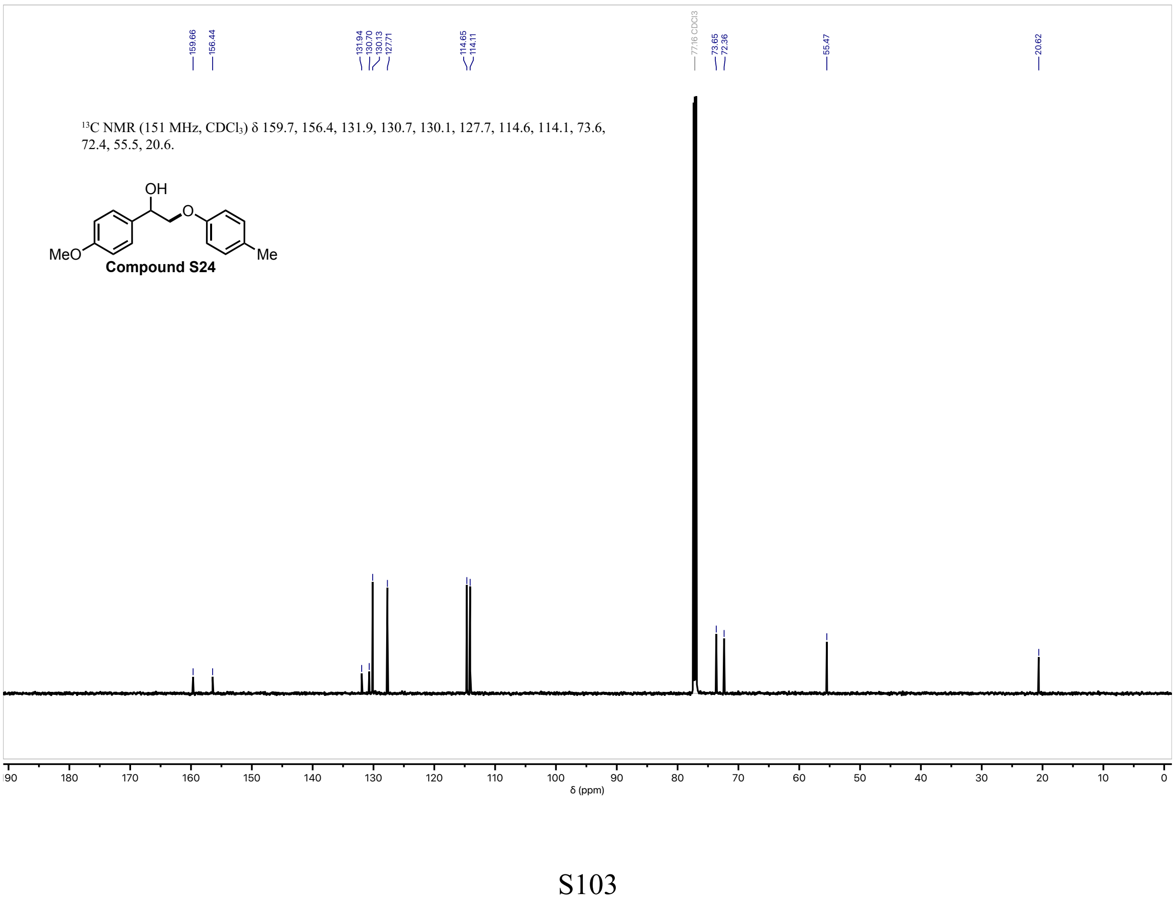

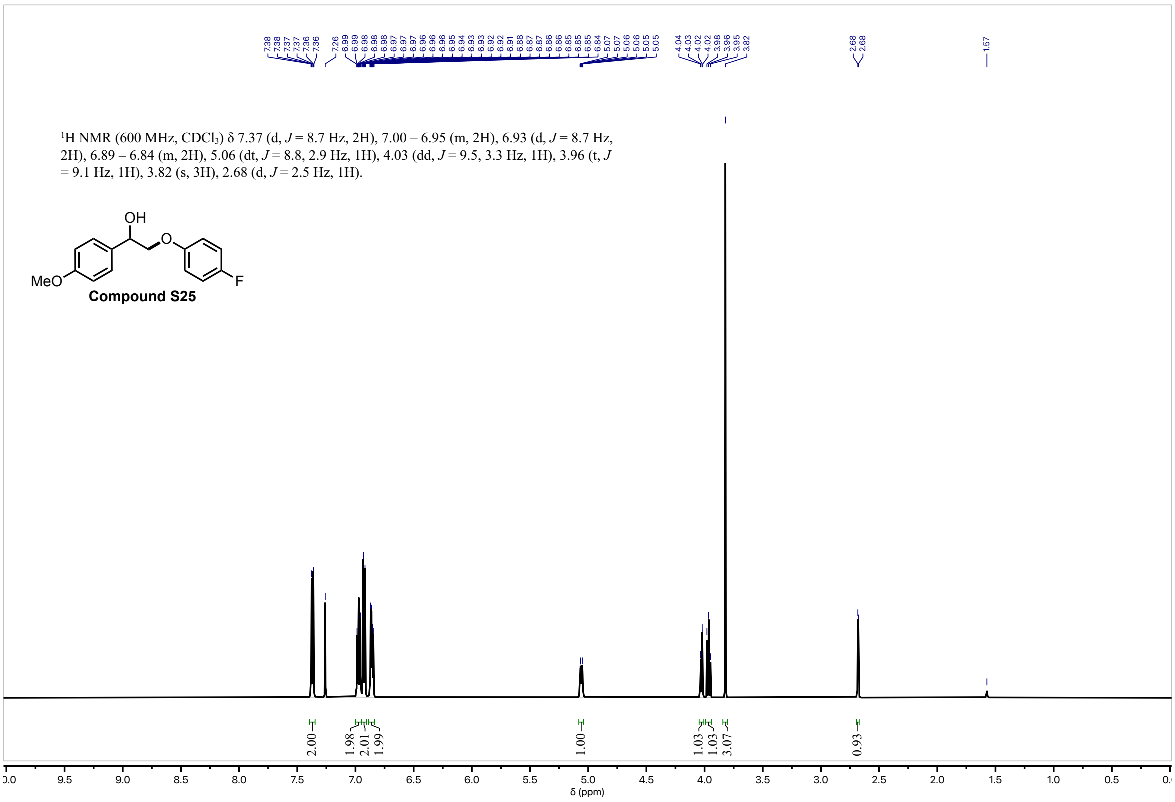
¹H NMR (600 MHz, CDCl$_3$) δ 7.37 (d, *J* = 8.7 Hz, 2H), 7.00 – 6.95 (m, 2H), 6.93 (d, *J* = 8.7 Hz, 2H), 6.89 – 6.84 (m, 2H), 5.06 (dt, *J* = 8.8, 2.9 Hz, 1H), 4.03 (dd, *J* = 9.5, 3.3 Hz, 1H), 3.96 (t, *J* = 9.1 Hz, 1H), 3.82 (s, 3H), 2.68 (d, *J* = 2.5 Hz, 1H).
Compound S25
δ (ppm)

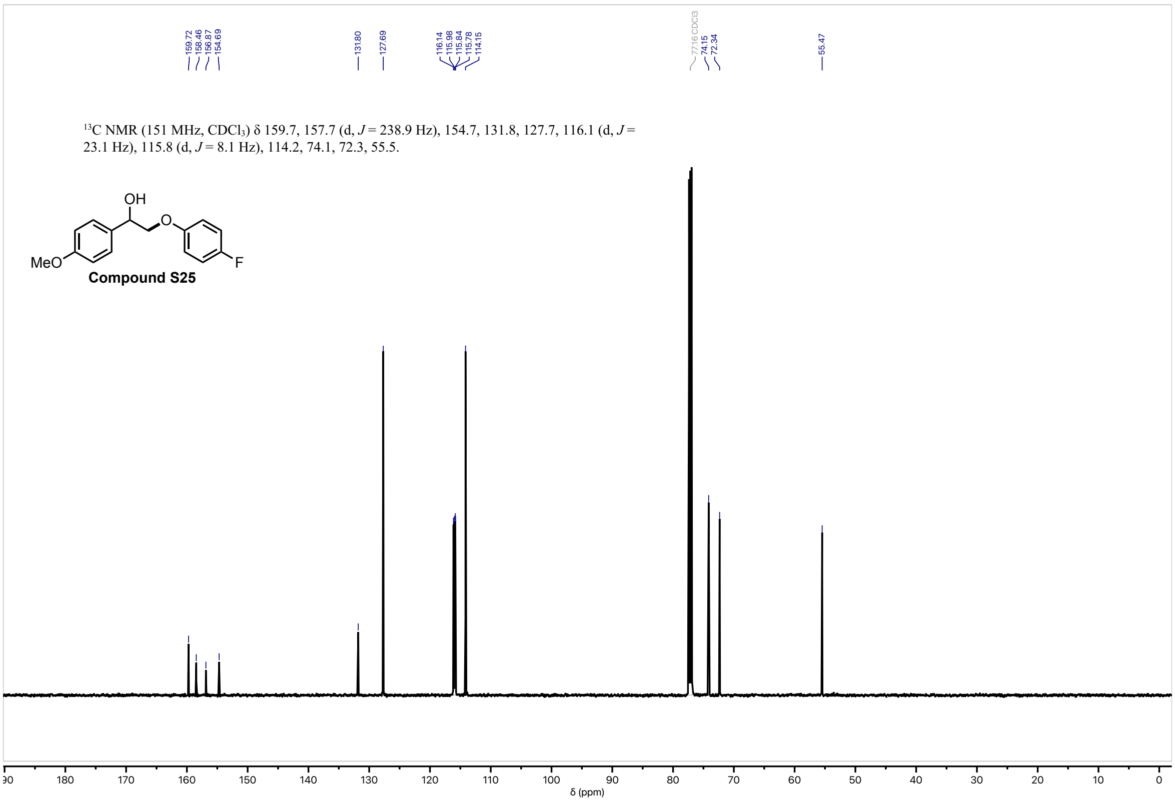
¹³C NMR (151 MHz, CDCl$_3$) δ 159.7, 157.7 (d, *J* = 238.9 Hz), 154.7, 131.8, 127.7, 116.1 (d, *J* = 23.1 Hz), 115.8 (d, *J* = 8.1 Hz), 114.2, 74.1, 72.3, 55.5.
Compound S25
δ (ppm)

^{1}H NMR (600 MHz, $CDCl_3$) δ 7.37 – 7.33 (m, 2H), 7.30 – 7.24 (m, 2H), 6.94 – 6.90 (m, 2H), 6.88 – 6.83 (m, 2H), 6.81 – 6.76 (m, 1H), 4.96 (ddd, J = 8.8, 4.2, 2.0 Hz, 1H), 3.82 (s, 3H), 3.51 (dd, J = 14.7, 9.0 Hz, 1H), 3.40 (dd, J = 14.7, 4.2 Hz, 1H), 2.94 (s, 3H), 2.46 (d, J = 2.1 Hz, 1H).

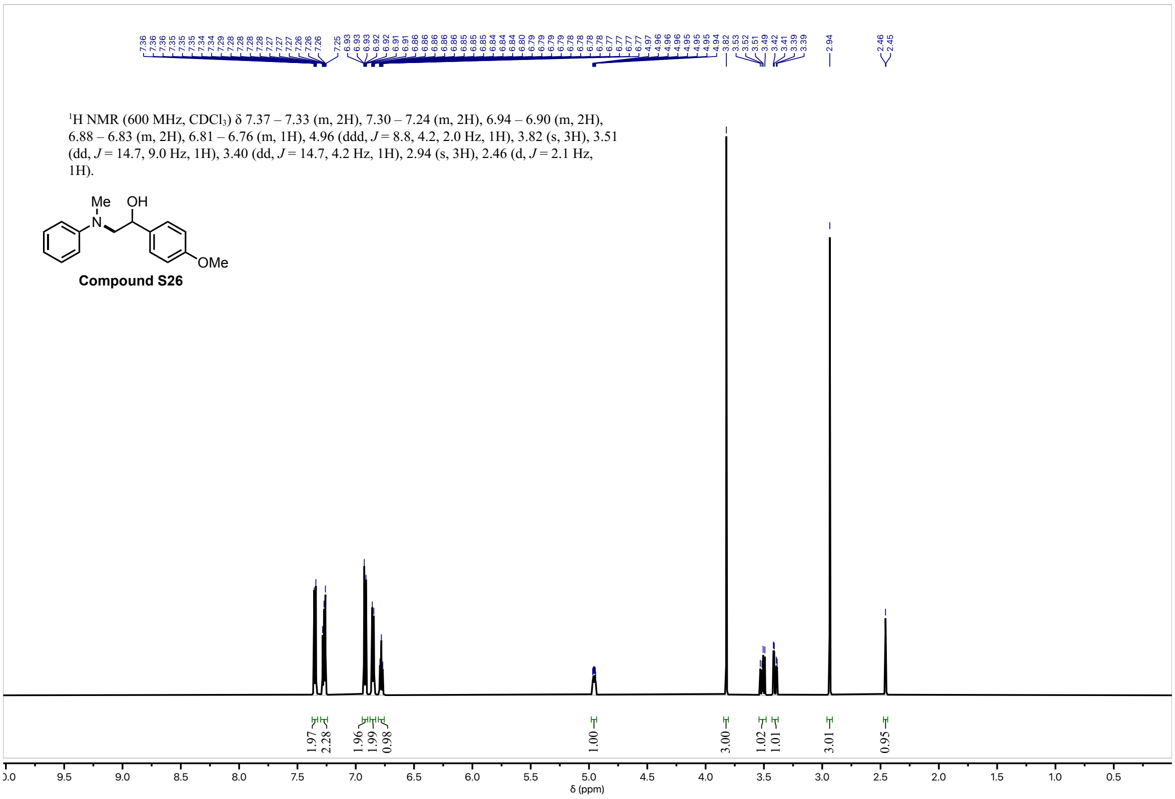

^{13}C NMR (151 MHz, $CDCl_3$) δ 159.4, 150.1, 134.2, 129.4, 127.3, 117.6, 114.1, 113.4, 71.5, 62.1, 55.5, 39.5.

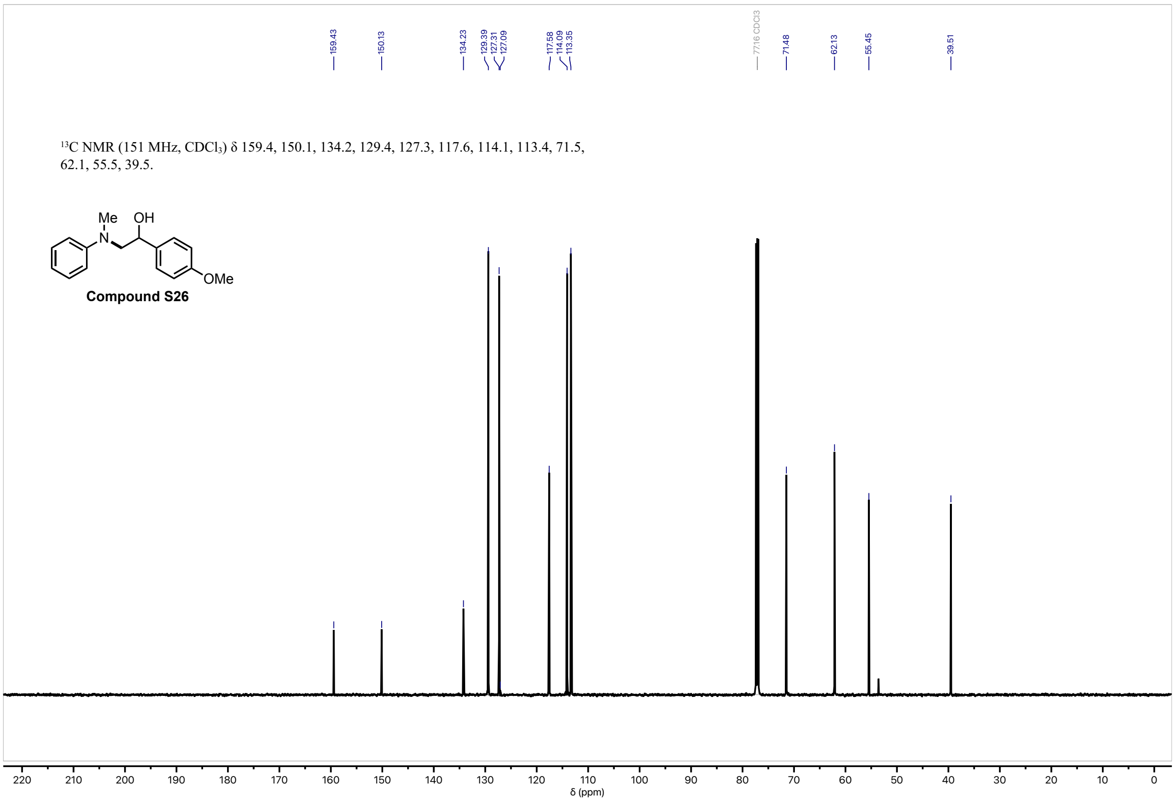

^{1}H NMR (400 MHz, cdcl$_3$) δ 7.22 (d, *J* = 8.6 Hz, 2H), 6.87 (d, *J* = 8.6 Hz, 2H), 4.31 (dd, *J* = 7.5, 3.0 Hz, 1H), 3.81 (s, 3H), 2.05 – 1.96 (m, 1H), 1.81 – 1.75 (m, 1H), 1.74 (d, *J* = 3.1 Hz, 1H), 1.70 – 1.57 (m, 3H), 1.39 – 1.31 (m, 1H), 1.30 – 1.08 (m, 3H), 1.08 – 0.97 (m, 1H), 0.94 – 0.83 (m, 1H).

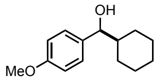

Compound S27

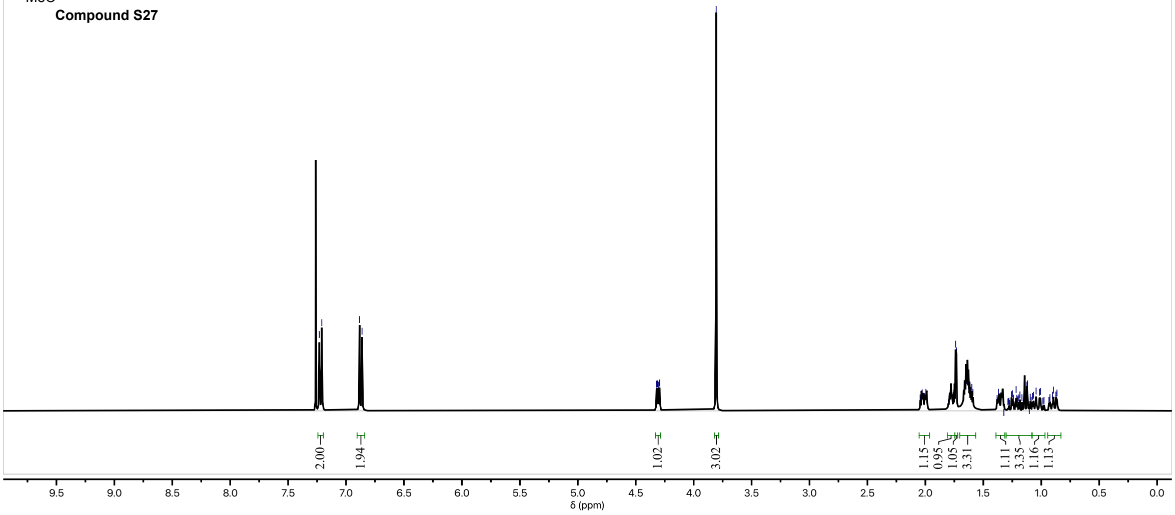

^{1}H NMR (600 MHz, CDCl$_3$) δ 8.06 (d, *J* = 7.9 Hz, 2H), 7.58 (t, *J* = 7.5 Hz, 1H), 7.45 (t, *J* = 7.7 Hz, 2H), 7.38 (d, *J* = 8.7 Hz, 2H), 6.92 (d, *J* = 8.7 Hz, 2H), 5.07 (dt, *J* = 8.2, 3.2 Hz, 1H), 4.49 (dd, *J* = 11.5, 3.5 Hz, 1H), 4.42 (dd, *J* = 11.5, 8.3 Hz, 1H), 3.82 (s, 3H), 2.52 (d, *J* = 3.2 Hz, 1H).

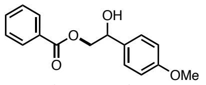

Compound S28

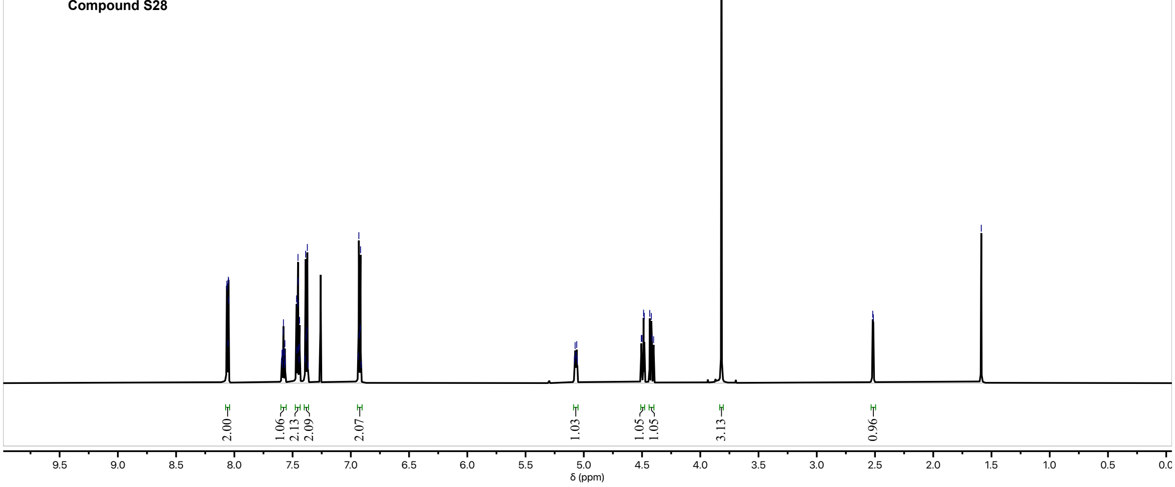

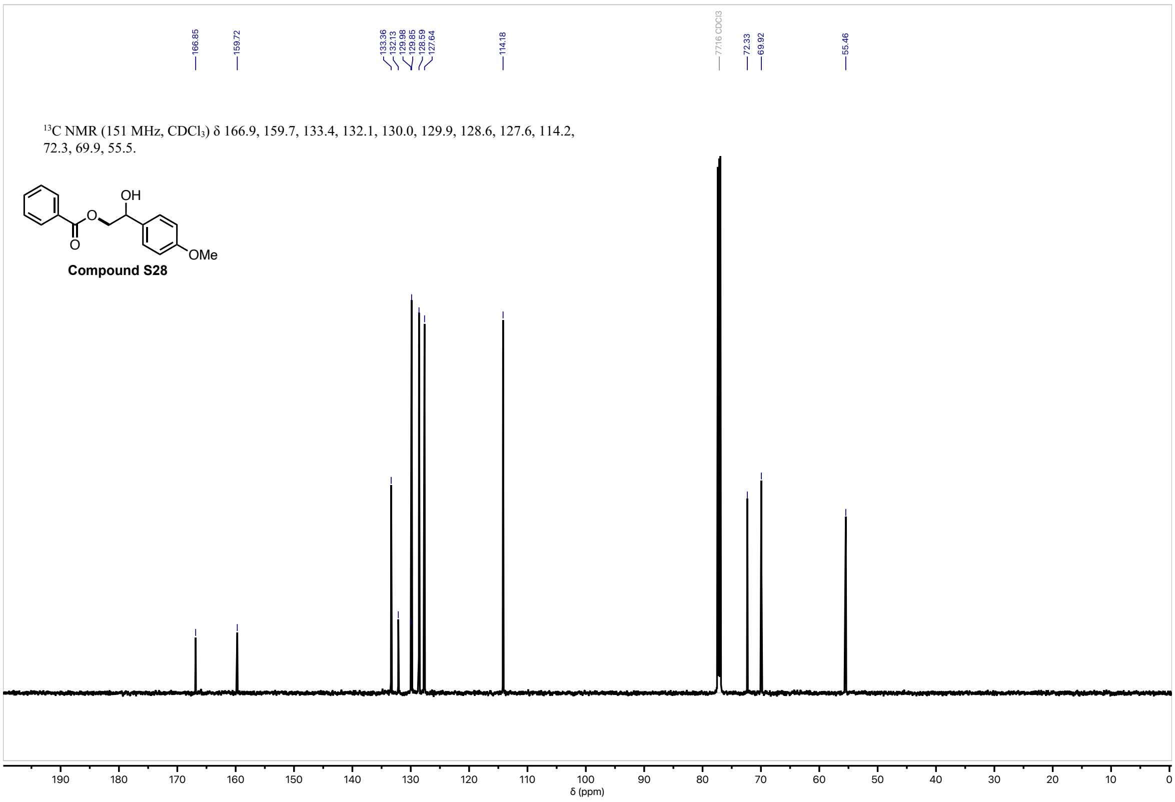

^{13}C NMR (151 MHz, $CDCl_3$) δ 166.9, 159.7, 133.4, 132.1, 130.0, 129.9, 128.6, 127.6, 114.2, 72.3, 69.9, 55.5.

Compound S28

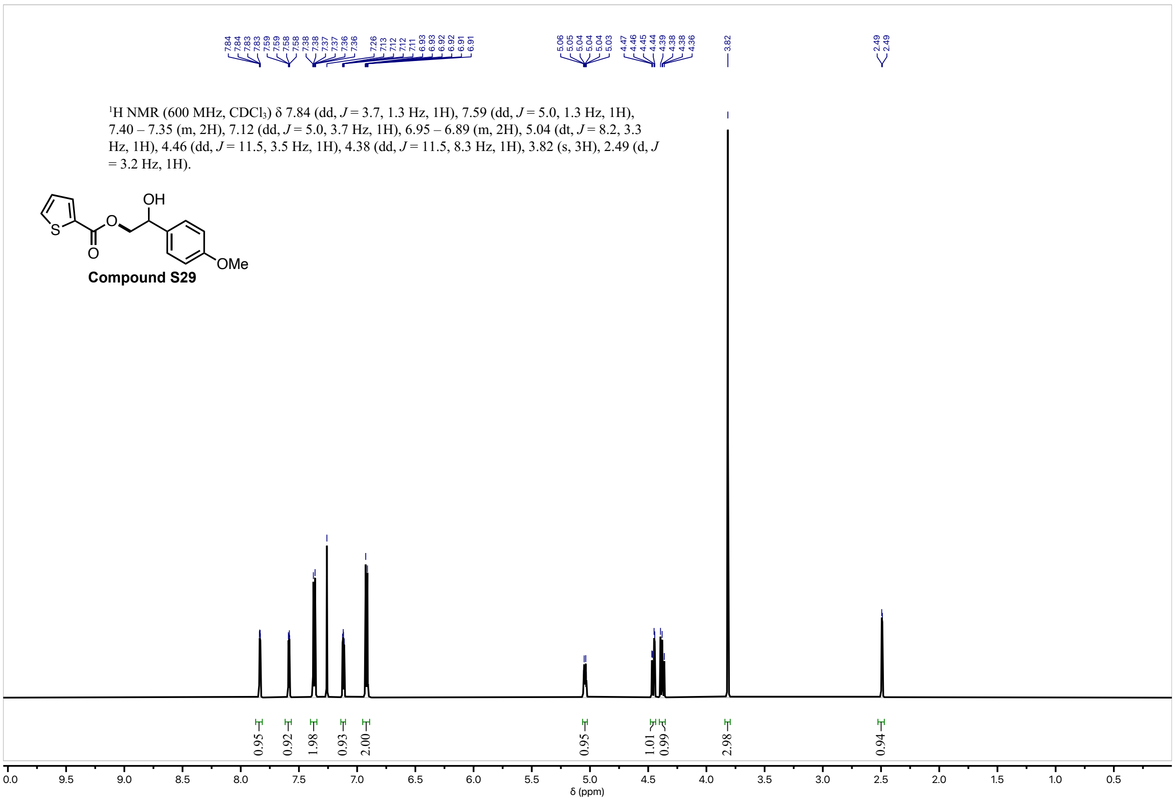

^{1}H NMR (600 MHz, $CDCl_3$) δ 7.84 (dd, J = 3.7, 1.3 Hz, 1H), 7.59 (dd, J = 5.0, 1.3 Hz, 1H), 7.40 – 7.35 (m, 2H), 7.12 (dd, J = 5.0, 3.7 Hz, 1H), 6.95 – 6.89 (m, 2H), 5.04 (dt, J = 8.2, 3.3 Hz, 1H), 4.46 (dd, J = 11.5, 3.5 Hz, 1H), 4.38 (dd, J = 11.5, 8.3 Hz, 1H), 3.82 (s, 3H), 2.49 (d, J = 3.2 Hz, 1H).

Compound S29

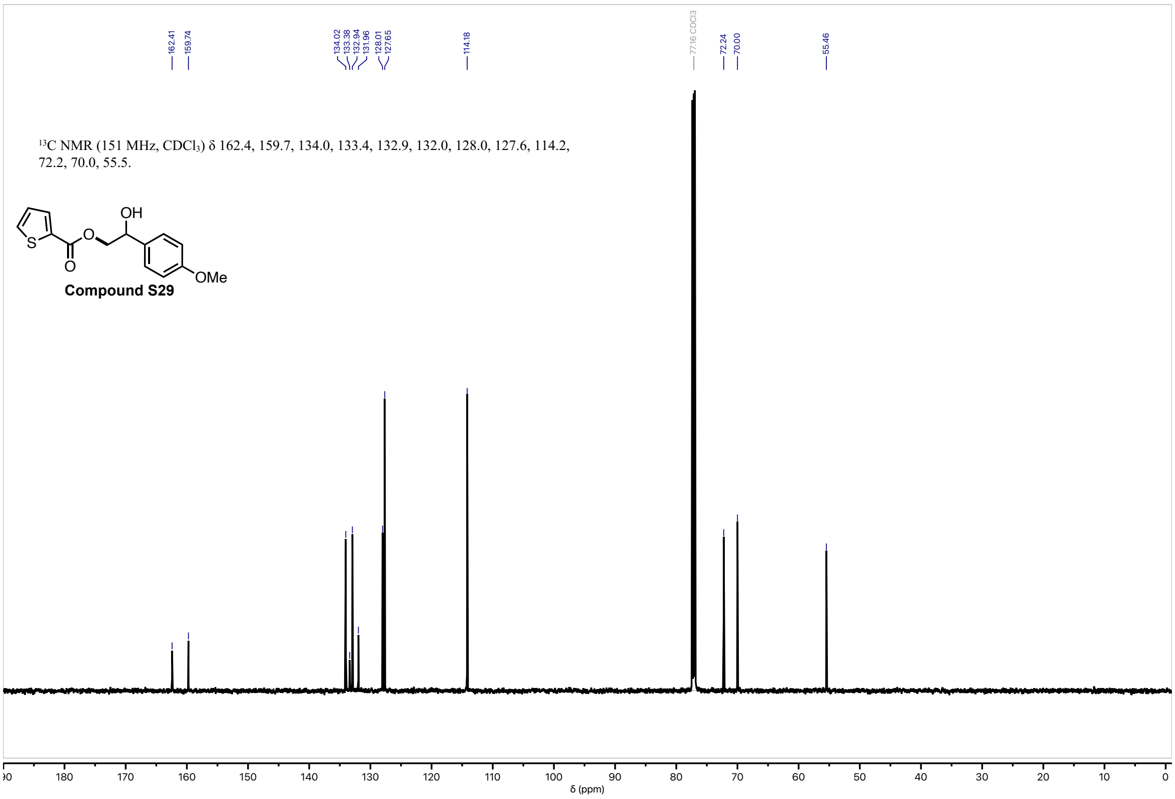

^{13}C NMR (151 MHz, $CDCl_3$) δ 162.4, 159.7, 134.0, 133.4, 132.9, 132.0, 128.0, 127.6, 114.2, 72.2, 70.0, 55.5.

Compound S29

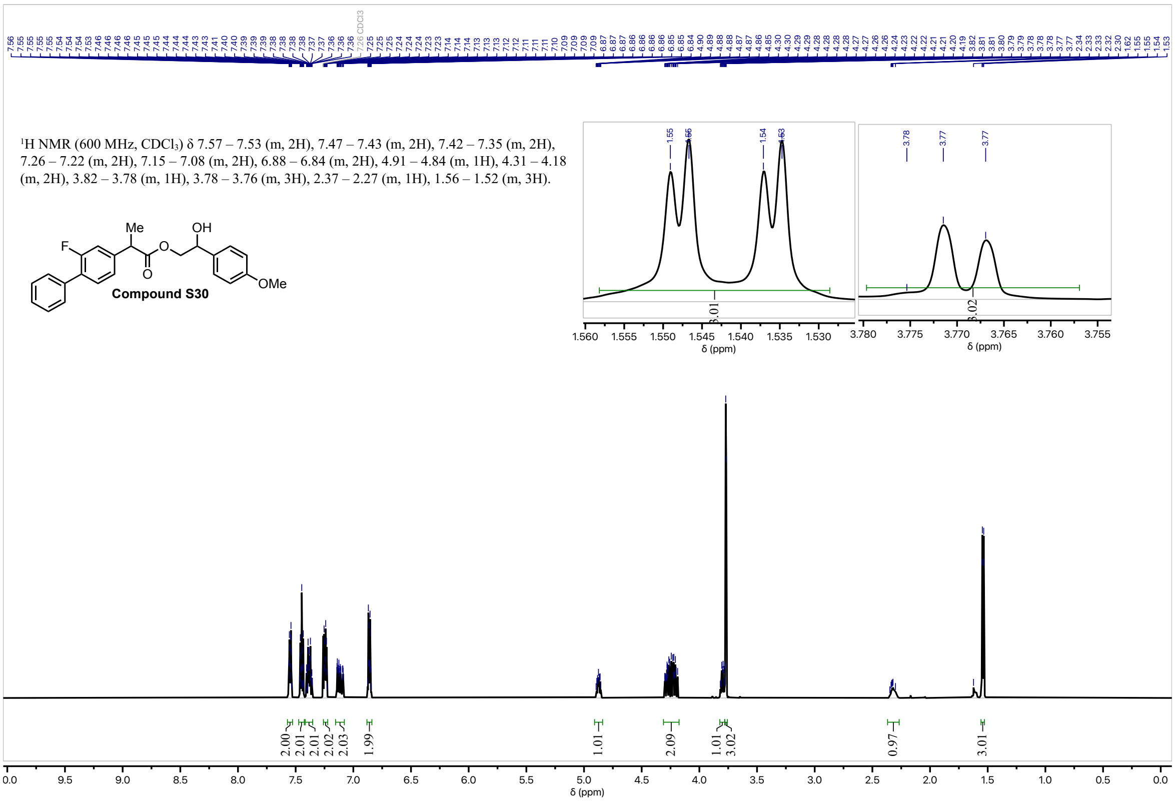

^{1}H NMR (600 MHz, $CDCl_3$) δ 7.57 – 7.53 (m, 2H), 7.47 – 7.43 (m, 2H), 7.42 – 7.35 (m, 2H), 7.26 – 7.22 (m, 2H), 7.15 – 7.08 (m, 2H), 6.88 – 6.84 (m, 2H), 4.91 – 4.84 (m, 1H), 4.31 – 4.18 (m, 2H), 3.82 – 3.78 (m, 1H), 3.78 – 3.76 (m, 3H), 2.37 – 2.27 (m, 1H), 1.56 – 1.52 (m, 3H).

Compound S30

^{13}C NMR (151 MHz, $CDCl_3$) δ 174.2, 174.1, 159.8 (d, J = 248.5 Hz), 159.7, 141.7 (d, J = 7.4 Hz), 141.7 (d, J = 7.5 Hz), 135.6, 131.9 (d, J = 4.8 Hz), 131.0 (d, J = 3.8 Hz), 129.1 (d, J = 2.8 Hz), 128.6, 128.0 (d, J = 13.5 Hz), 127.8, 127.5, 123.7, 123.6, 123.6, 115.4 (d, J = 23.7 Hz), 114.1, 72.0, 69.6, 69.6, 55.4, 45.1, 45.1, 18.5.

Compound S30

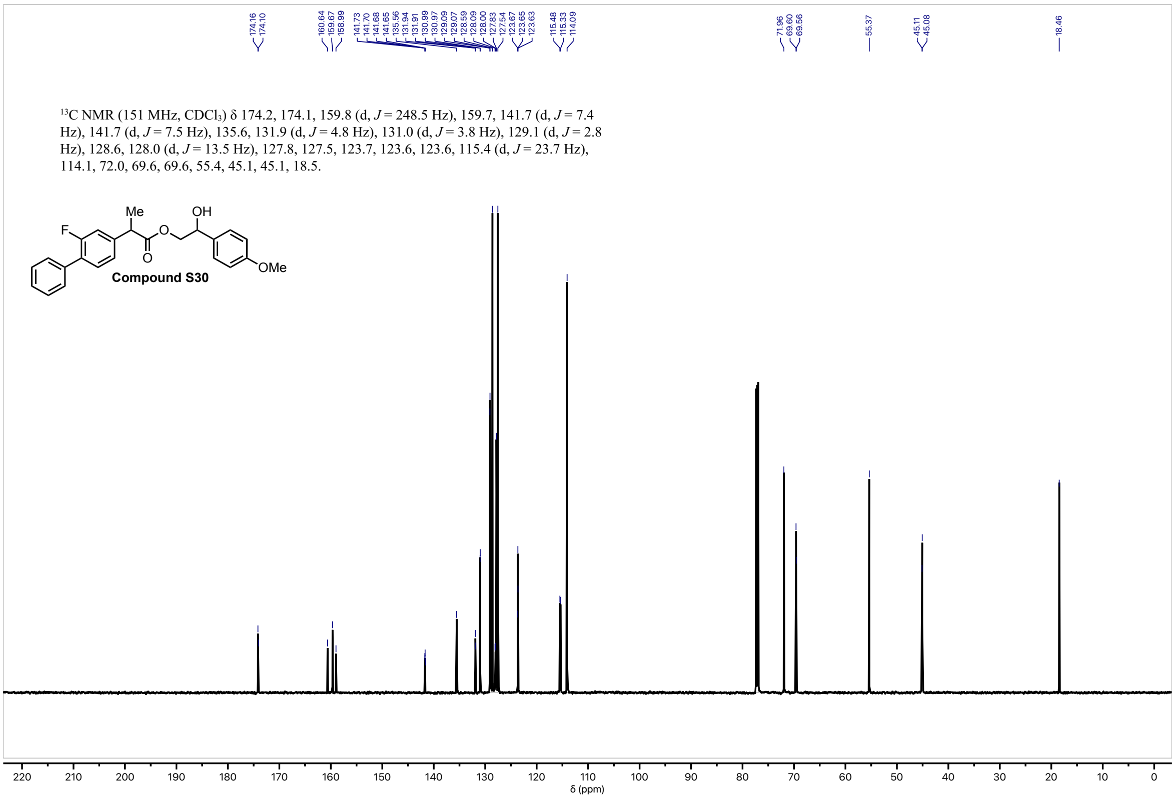

^{1}H NMR (600 MHz, $CDCl_3$) δ 7.25 – 7.21 (m, 2H), 7.21 – 7.17 (m, 2H), 7.14 – 7.08 (m, 2H), 6.90 – 6.82 (m, 2H), 4.86 – 4.78 (m, 1H), 4.28 – 4.09 (m, 2H), 3.80 (s, 3H), 3.77 – 3.72 (m, 1H), 2.46 (d, J = 7.2 Hz, 2H), 2.38 – 2.27 (m, 1H), 1.91 – 1.81 (m, 1H), 1.50 (dd, J = 7.2, 0.9 Hz, 3H), 0.91 (d, J = 6.6 Hz, 6H).

Compound S31

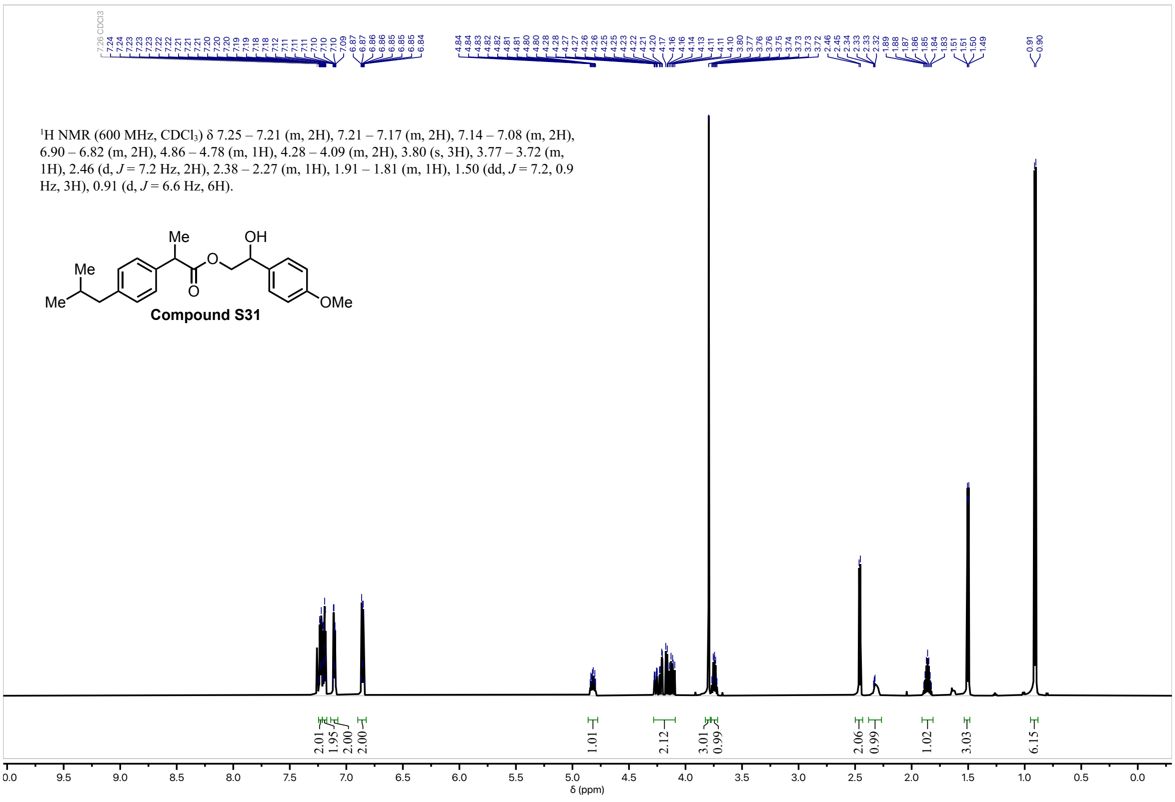

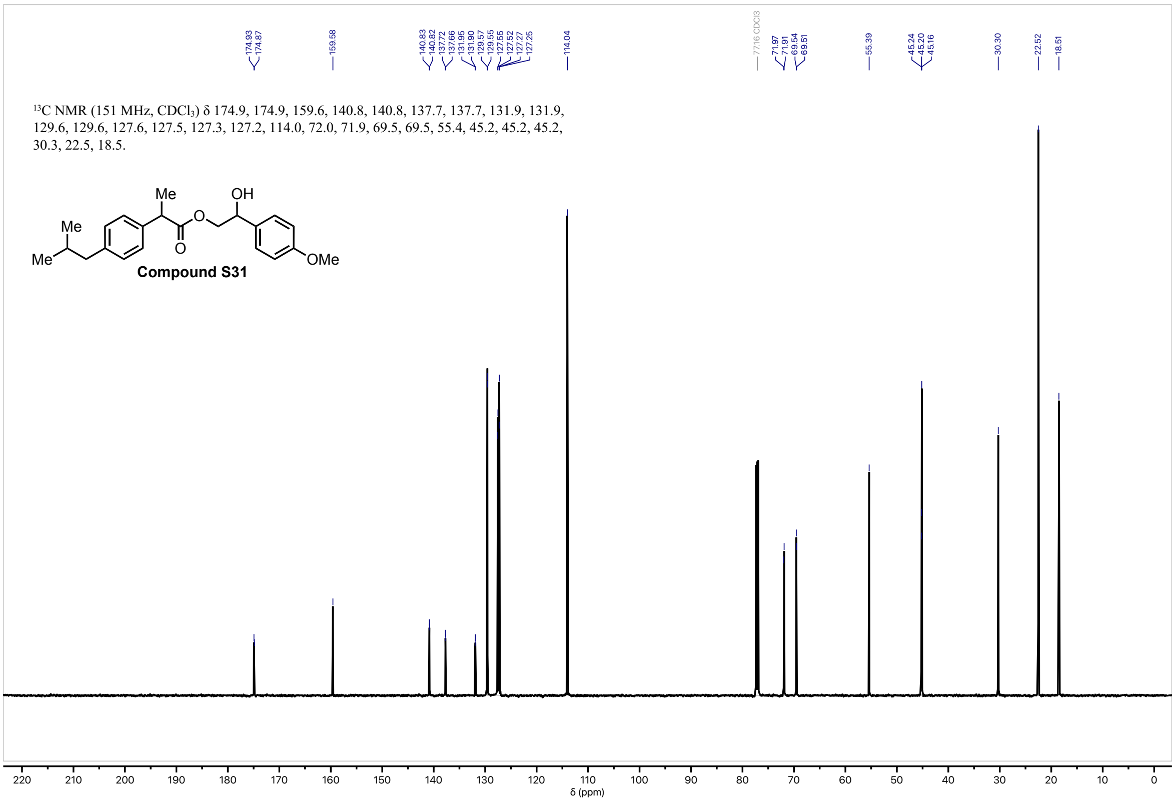

^{13}C NMR (151 MHz, $CDCl_3$) δ 174.9, 174.9, 159.6, 140.8, 140.8, 137.7, 137.7, 131.9, 131.9, 129.6, 129.6, 127.6, 127.5, 127.3, 127.2, 114.0, 72.0, 71.9, 69.5, 69.5, 55.4, 45.2, 45.2, 45.2, 30.3, 22.5, 18.5.

Compound S31

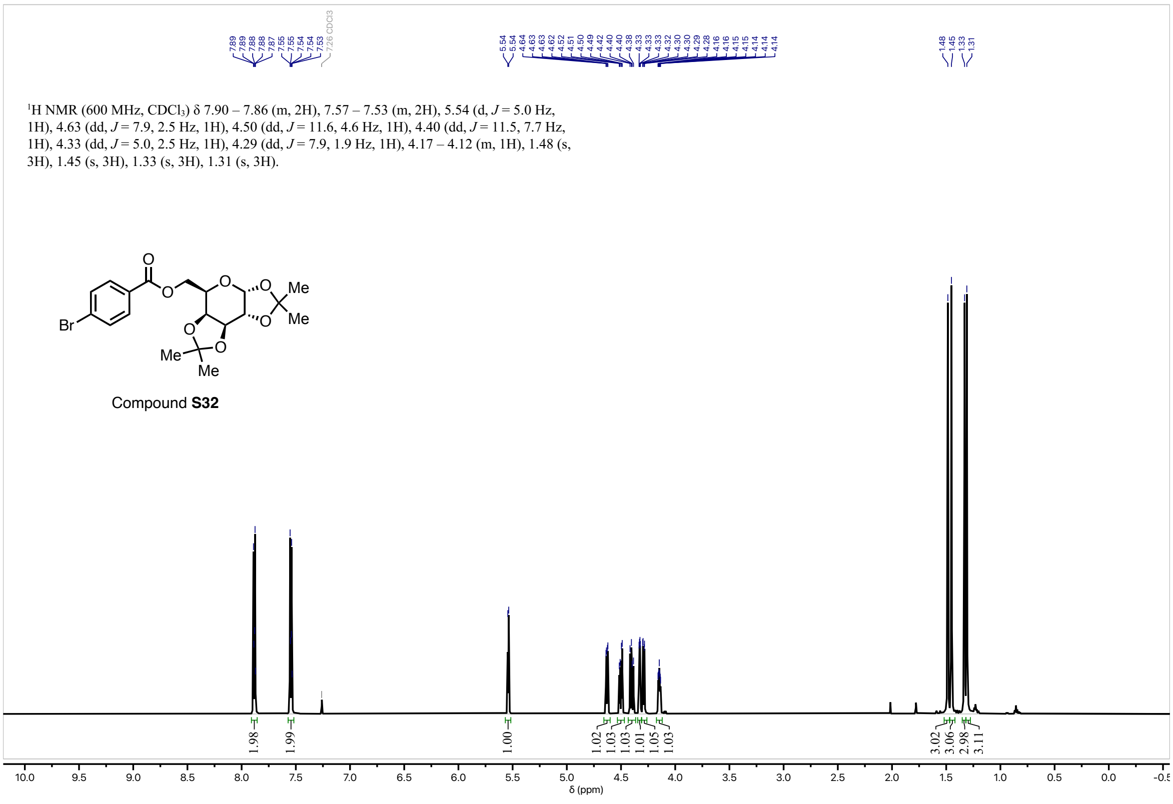

^{1}H NMR (600 MHz, $CDCl_3$) δ 7.90 – 7.86 (m, 2H), 7.57 – 7.53 (m, 2H), 5.54 (d, J = 5.0 Hz, 1H), 4.63 (dd, J = 7.9, 2.5 Hz, 1H), 4.50 (dd, J = 11.6, 4.6 Hz, 1H), 4.40 (dd, J = 11.5, 7.7 Hz, 1H), 4.33 (dd, J = 5.0, 2.5 Hz, 1H), 4.29 (dd, J = 7.9, 1.9 Hz, 1H), 4.17 – 4.12 (m, 1H), 1.48 (s, 3H), 1.45 (s, 3H), 1.33 (s, 3H), 1.31 (s, 3H).

Compound **S32**

^{13}C NMR (151 MHz, $CDCl_3$) δ 165.8, 131.8, 131.3, 129.1, 128.2, 109.8, 108.9, 96.4, 71.2, 70.8, 70.6, 66.2, 64.3, 26.1, 26.1, 25.1, 24.6.

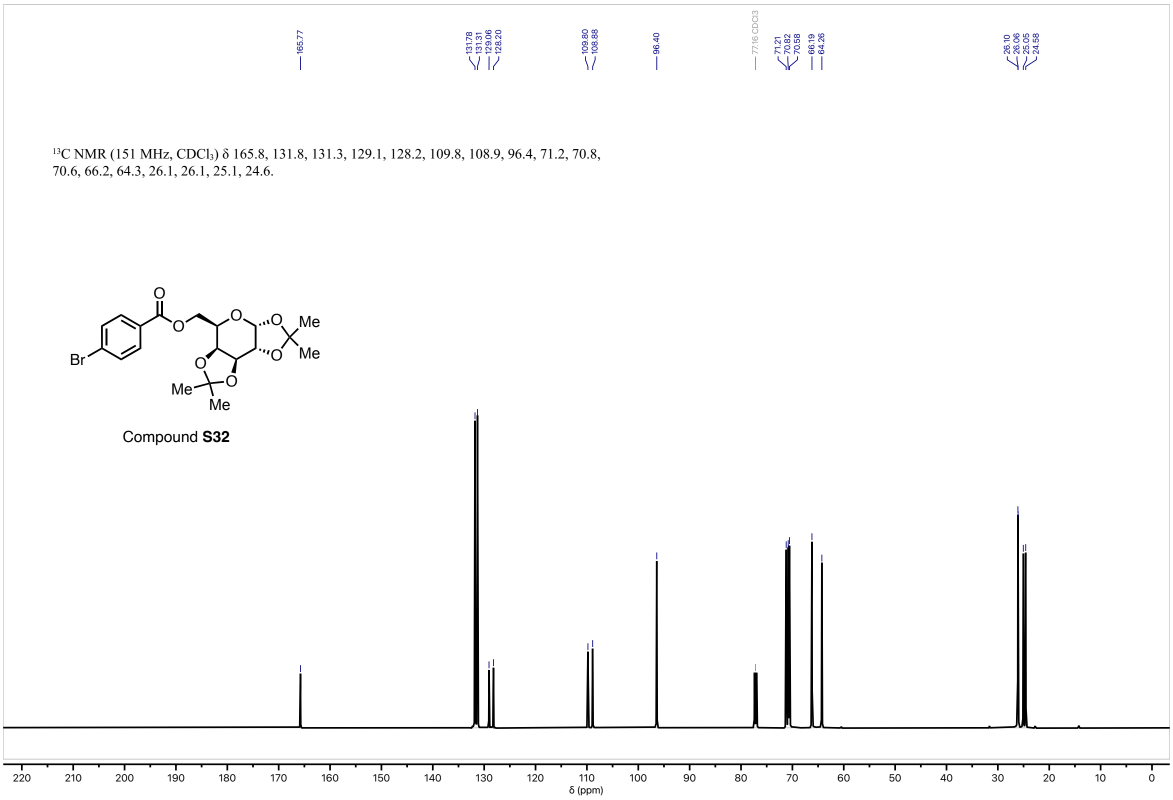

^{1}H NMR (600 MHz, $CDCl_3$) δ 7.63 – 7.59 (m, 2H), 7.59 – 7.55 (m, 2H), 7.35 – 7.24 (m, 3H), 7.19 – 7.12 (m, 2H), 6.60 (d, J = 7.5 Hz, 1H), 5.06 (dt, J = 7.5, 5.7 Hz, 1H), 4.24 (q, J = 7.1 Hz, 2H), 3.35 – 3.19 (m, 2H), 1.31 (t, J = 7.2 Hz, 3H).

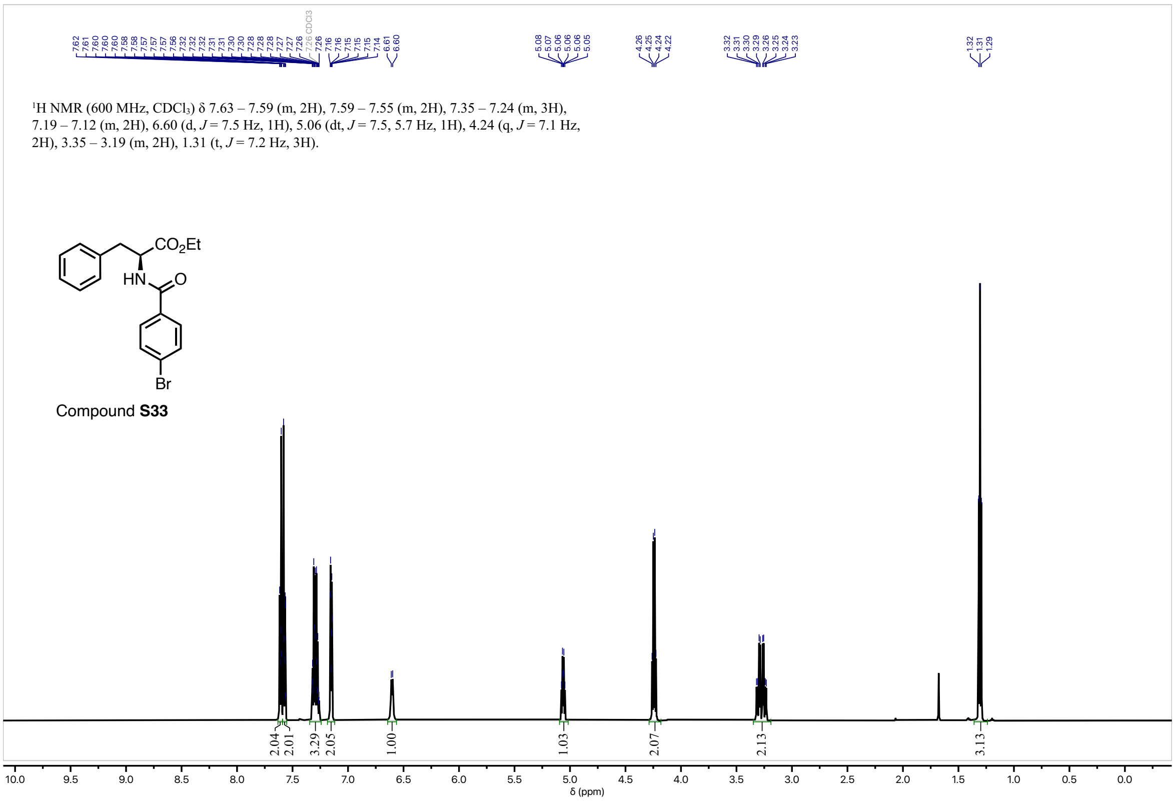

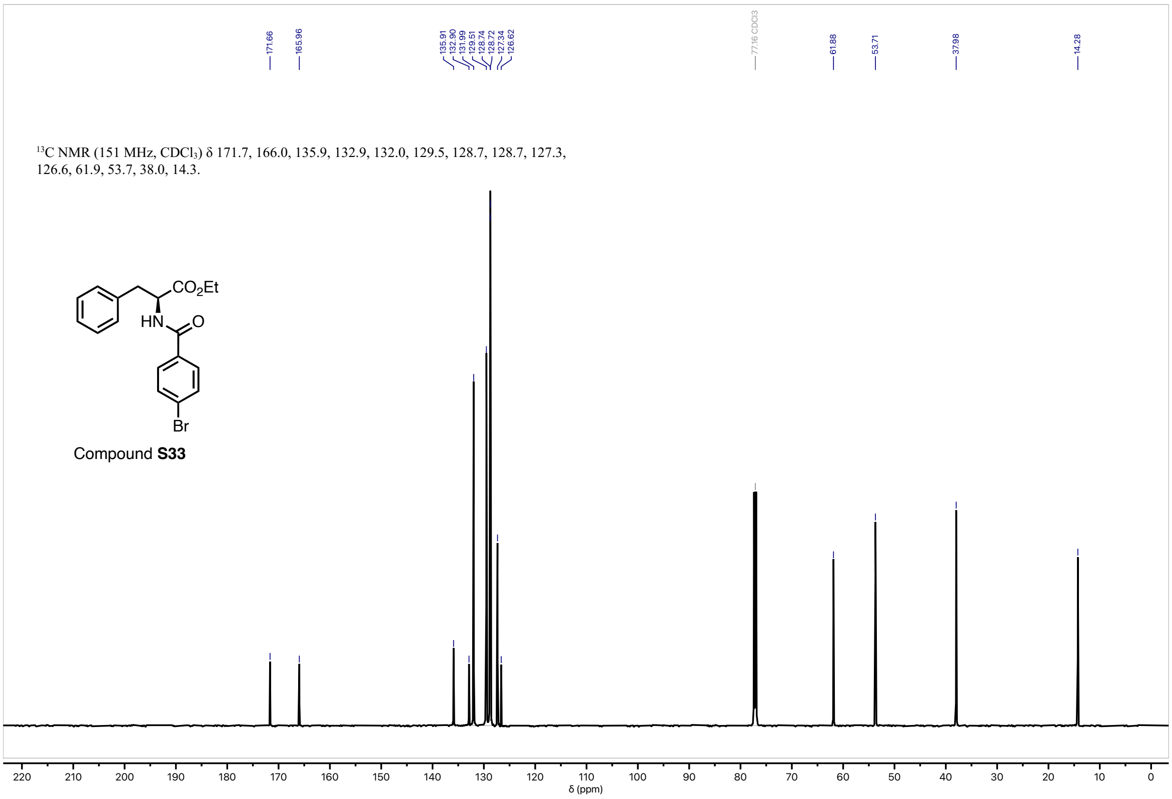
^{13}C NMR (151 MHz, CDCl$_3$) δ 171.7, 166.0, 135.9, 132.9, 132.0, 129.5, 128.7, 128.7, 127.3, 126.6, 61.9, 53.7, 38.0, 14.3.
Compound S33
δ (ppm)

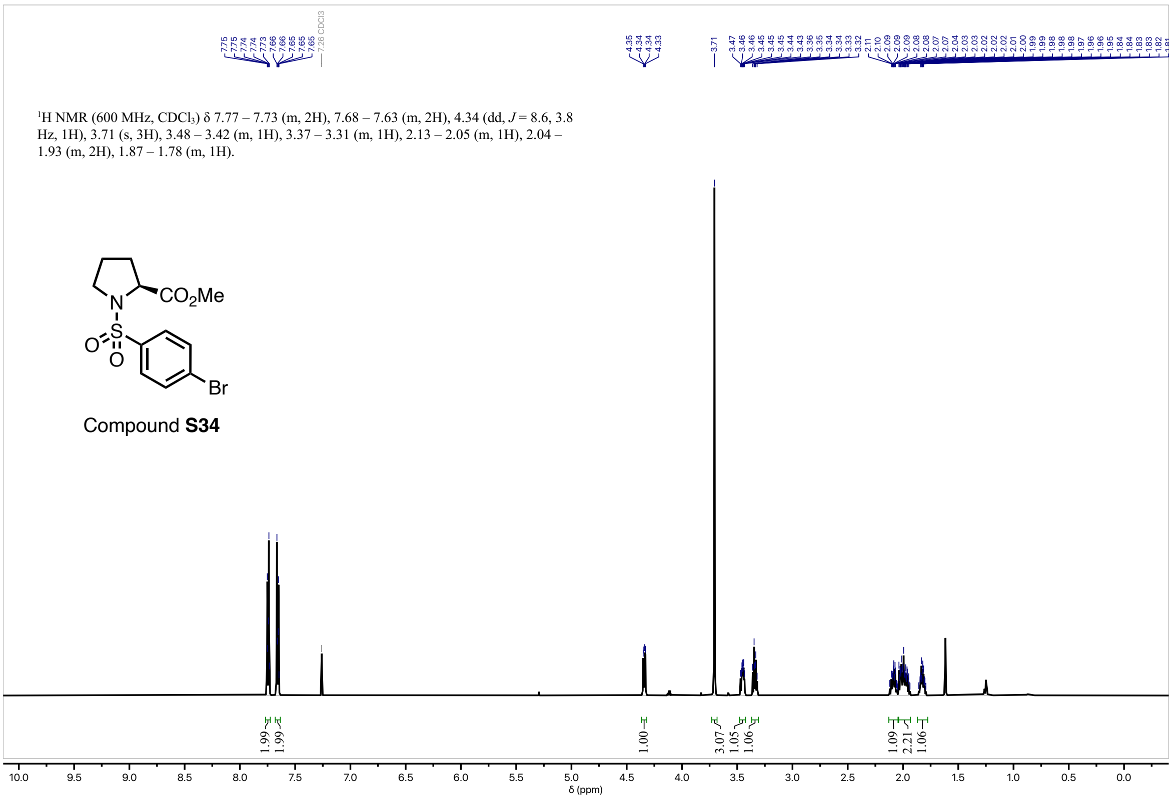
^{1}H NMR (600 MHz, CDCl$_3$) δ 7.77 – 7.73 (m, 2H), 7.68 – 7.63 (m, 2H), 4.34 (dd, J = 8.6, 3.8 Hz, 1H), 3.71 (s, 3H), 3.48 – 3.42 (m, 1H), 3.37 – 3.31 (m, 1H), 2.13 – 2.05 (m, 1H), 2.04 – 1.93 (m, 2H), 1.87 – 1.78 (m, 1H).
Compound S34
δ (ppm)

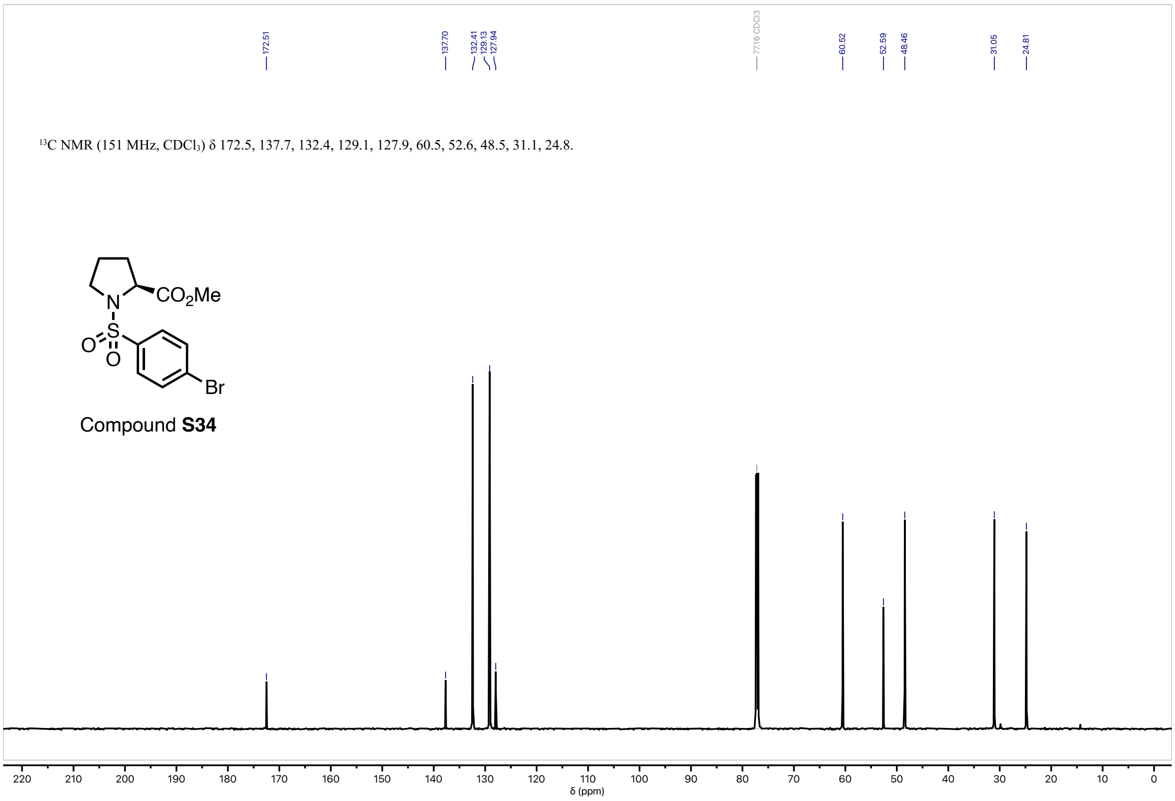

172.51
137.70
132.41
129.13
127.94
77.16 CDCl3
60.52
52.59
48.46
31.05
24.81
^{13}C NMR (151 MHz, $CDCl_3$) δ 172.5, 137.7, 132.4, 129.1, 127.9, 60.5, 52.6, 48.5, 31.1, 24.8.
N
CO_2Me
S
O
O
Br
Compound **S34**
220 210 200 190 180 170 160 150 140 130 120 110 100 90 80 70 60 50 40 30 20 10 0
δ (ppm)
